# Supplementary material for: Statistical methods applied for the assessment of the HIV cascade and continuum of care: a systematic scoping review
Source: BMJ Open. 2023 Nov 23;13(11):e071392. doi: 10.1136/bmjopen-2022-071392 (PMC10668296; doi:10.1136/bmjopen-2022-071392)
Supplement: Supplementary data [file bmjopen-2022-071392supp001.pdf]

Supplementary material

Table S1: Database search queries

Database	Query number	Query	Number of articles
PubMed (Searched on 05-04-2022)	1	((HIV) AND ((Care cascade) OR (cascade of care) OR (UNAIDS 90-90-90) OR (continuum of care[Title/Abstract]))) **OR** (((HIV) AND ((Care cascade) OR (UNAIDS 90-90-90) OR (cascade of care) OR (continuum of care[Title/Abstract]))) AND ((Multistate models[Title/Abstract]) OR (longitudinal[Title/Abstract]) OR (Markov models[Title/Abstract]) OR (Multi-state models[Title/Abstract])))	1,608
CINAHL Complete (04-07-2022)	S1	MH human immunodeficiency virus or hiv	32,691
	S2	MH hiv infection	945
	S3	TI (HIV infection or Human immunodeficiency virus or HIV) OR AB (HIV infection or Human immunodeficiency virus or HIV)	104,219
	S4	S1 OR S2 OR S3	106,982
	S5	MH care cascade	1,284
	S6	MH continuum of care	7,104
	S7	MH 90-90-90	17,568
	S8	TI (care cascade or cascade of care or continuum of care or "care cascade progress" or UNAIDS 90-90-90 progress" or UNAIDS 90-90-90 or "cross-sectional care cascade") OR AB (care cascade or cascade of care or continuum of care or "care cascade progress" or UNAIDS 90-90-90 progress" or UNAIDS 90-90-90 or "cross-sectional care cascade")	5,497
	S9	S5 OR S6 OR S7 OR S8	6,288
	S10	S4 AND S9	916
	S11	MH Multistate models	293
	S12	MH Multi-state models	1,763
	S13	MH Markov models	2,000
	S14	TI (Multistate models or Multi-state models or Markov models or "longitudinal care cascade") OR AB (Multistate models or Multi-state models or Markov models or "longitudinal care cascade")	3,925
	S15	S11 OR S12 OR S13 OR S14	3,992
	S16	S10 AND S15	5
	S17	S10 OR S16	916
EMBASE (Search date 15-07-2022)	#1	'human immunodeficiency virus'/exp OR 'human immunodeficiency virus infection'/exp OR 'human immunodeficiency virus':ti,ab,kw OR 'human immunodeficiency virus infection':ti,ab,kw OR hiv:ti,ab,kw OR 'hiv infection':ti,ab,kw	601495
	#2	'cascade of care'/exp OR 'continuum of care'/exp OR 'care continuum':ti,ab,kw OR '90 90 90':ti,ab,kw OR 'care cascade':ti,ab,kw OR 'cascade of care':ti,ab,kw OR 'continuum of care':ti,ab,kw OR 'care cascade progress':ti,ab,kw OR 'unaids 90-90-90 progress':ti,ab,kw	9220

Kalinjuma AV, *et al. BMJ Open* 2023; 13:e071392. doi: 10.1136/bmjopen-2022-071392

		OR 'unaids 90-90-90':ti,ab,kw OR 'cross-sectional care cascade':ti,ab,kw	
	#3	#1 AND #2	3145
	#4	'multistate model'/exp OR 'multistate model' OR 'multi state model'/exp OR 'multi state model' OR 'markov chain'/exp OR 'markov chain' OR 'multistate models':ti,ab,kw OR 'multi state models':ti,ab,kw OR 'markov models':ti,ab,kw OR 'longitudinal':ti,ab,kw	429711
	#5	#3 AND #4	171
	#6	#3 OR #5	3145
Other strategic searches	PubMed and Google scholar search using individual key works such as “HIV care cascade” or “HIV care cascade and multistate models” or “HIV care cascade and multi-state models” or “HIV care cascade and Markov models”. Bibliographic list of the articles.		136

Table S2: Data extraction tool for the scoping review

Information extracted from articles	Possible responses
Paper ID number	
Authors	
Study site in terms of country	
The specific study site of the research article	
The level of study representation	
The study design	Cross-sectional, Case-Control, Longitudinal, Cohort, Prospective cohort, Retrospective cohort, Clinical trial, Not stated in the article, Other
Other study design not listed above	
Data sources for the studies reviewed	Cohort data, Laboratory test data (including CD4 and viral load tests), Medical records from the hospital, Survey data, Simulated data (mathematical modeling), Surveillances, Surveillances, Others
Specify other data sources not listed in the list above	
Number of patients/participants included in the final analysis of the study	
The actual age of participants included in the study	
Categories of the age of participants included in the study	All (children and adults) PLHIV, Adults (≥18 years), Adults (≥15 years), Adults (≥16 years), Adolescents and adults (>=13 years), Adolescents and adults (>=12 years), Adolescents (10-19 years), Neonates
Type of participants included in a research article	All (mixture) PLHIV, Children only, Adolescents, Adults, Key populations, Migrants, Prisoners, Veterans, Refugees, Military
Key population, please specify	Men having sex with men, Female sex workers, Injected drug users, Transgender women, Transgender men
Cascade size	Full cascade stages, More than full cascade (> 6 stages), Medium cascade stages, Small to very small cascade stages, Other
Specify other study outcomes	
Number of the cascade stages	
Actual stage name	
stage 1	
stage 2	
stage 3	
stage 4	
stage 5	
stage 6	
stage 7	
stage 8	
stage 9	
Absorbing state	Death, LTFU, Transfer, Other
Specify other absorbing states	

Kalinjuma AV, *et al. BMJ Open* 2023; 13:e071392. doi: 10.1136/bmjopen-2022-071392

**Information extracted from articles**	**Possible responses**
The type of multistate model used	Non-Parametric multistate model, Parametric, continuous-time multistate Markov model, Time-homogeneous Markov models, Discrete-time multistate Markov model, General multistate model, Other
Specify other types of multistate models used	
Multistate model assumptions	
Assessment starting point (baseline)	Enrolment/recruitment date, ART start date
Reason for censoring	
Descriptions of statistics used	Prevalence, Incidence rates, Proportion for each stage of HIV care cascade, Chi-square test of independence, Fisher Exact test (small sample size), t-test, Wilcoxon rank test, Time spent in each cascade stage, Transition probabilities, Mean, Standard deviation, Median, Interquartile range
Type of regression models used	Logistics regression, Cox PH regression, Poisson regression, competing risk regression, Generalized linear regression model (GLM), Weighted linear regression, Mathematical back-calculation, Log-Binomial regression, Generalized linear mixed models (GLMM), Multinomial regression model, Other
Other regression models, specify	
The method used for handling missing data	Weighted analysis, Multiple imputations, Missing data indicator, Not addressed
Methods used for assessing the goodness of fit of the model	Pearson-type Chi-Square test, Visual comparison of observed and fitted value plots, Not assessed, Other
Other methods for assessing the goodness of fit of the model	
Broad categories of approaches used in research articles	Cross-sectional, Longitudinal
Specification of the cascade staging method	Yes, no
Cascade staging method	Dependent, independent, and both dependent and independent

Kalinjuma AV, *et al. BMJ Open* 2023; 13:e071392. doi: 10.1136/bmjopen-2022-071392

Table S3: List of articles included

Paper Number	Authors	Reference
1	Lippman et al 2016	Lippman, S. A., Shade, S. B., El Ayadi, A. M., Gilvydis, J. M., Grignon, J. S., Liegler, T., Morris, J., Naidoo, E., Prach, L. M., Puren, A., & Barnhart, S. (2016). Attrition and Opportunities Along the HIV Care Continuum: Findings From a Population-Based Sample, North West Province, South Africa. Journal of acquired immune deficiency syndromes (1999), 73(1), 91–99. https://doi.org/10.1097/QAI.0000000000001026
2	Supervie et al 2016	Supervie, V., Marty, L., Lacombe, J. M., Dray-Spira, R., Costagliola, D., & FHDH-ANRS CO4 study group (2016). Looking Beyond the Cascade of HIV Care to End the AIDS Epidemic: Estimation of the Time Interval From HIV Infection to Viral Suppression. Journal of acquired immune deficiency syndromes (1999), 73(3), 348–355. https://doi.org/10.1097/QAI.0000000000001120
3	CDC 2011	Centers for Disease Control and Prevention (CDC) (2011). Vital signs: HIV prevention through care and treatment--United States. MMWR. Morbidity and mortality weekly report, 60(47), 1618–1623.
4	Nosyk et al 2014	Nosyk, B., Montaner, J. S. G., Colley, G., Lima, V. D., Chan, K., Heath, K., Yip, B., Samji, H., Gilbert, M., Barrios, R., Gustafson, R., Hogg, R. S., & STOP HIV/AIDS Study Group (2014). The cascade of HIV care in British Columbia, Canada, 1996-2011: a population-based retrospective cohort study. The Lancet. Infectious diseases, 14(1), 40–49. https://doi.org/10.1016/S1473-3099(13)70254-8
5	Kohler et al 2015	Kohler, P., Schmidt, A. J., Cavassini, M., Furrer, H., Calmy, A., Battegay, M., Bernasconi, E., Ledergerber, B., Vernazza, P., & Swiss HIV Cohort Study (2015). The HIV care cascade in Switzerland: reaching the UNAIDS/WHO targets for patients diagnosed with HIV. AIDS (London, England), 29(18), 2509–2515. https://doi.org/10.1097/QAD.0000000000000878
6	Maman et al 2015	Maman, D., Zeh, C., Mukui, I., Kirubi, B., Masson, S., Opolo, V., Szumilin, E., Riche, B., & Etard, J. F. (2015). Cascade of HIV care and population viral suppression in a high-burden region of Kenya. AIDS (London, England), 29(12), 1557–1565. https://doi.org/10.1097/QAD.0000000000000741
7	Takuva et al 2017	Takuva, S., Brown, A. E., Pillay, Y., Delpech, V., & Puren, A. J. (2017). The continuum of HIV care in South Africa: implications for achieving the second and third UNAIDS 90-90-90 targets. AIDS (London, England), 31(4), 545–552. https://doi.org/10.1097/QAD.0000000000001340
8	Mangal et al 2014	Mangal, J. P., Rimland, D., & Marconi, V. C. (2014). The continuum of HIV care in a Veterans' Affairs clinic. AIDS research and human retroviruses, 30(5), 409–415. https://doi.org/10.1089/aid.2013.0232

Paper Number	Authors	Reference
9	Wilton et al 2019	Wilton, J., Liu, J., Sullivan, A., Rachlis, B., Marchand-Austin, A., Giles, M., Light, L., Rank, C., Burchell, A. N., Gardner, S., Sider, D., Gilbert, M., Kroch, A. E., & Ontario HIV Epidemiology and Surveillance Initiative (2019). Trends in HIV care cascade engagement among diagnosed people living with HIV in Ontario, Canada: A retrospective, population-based cohort study. PloS one, 14(1), e0210096. https://doi.org/10.1371/journal.pone.0210096
10	Kerkerian et al 2018	Kerkerian, G., Kestler, M., Carter, A., Wang, L., Kronfli, N., Sereda, P., Roth, E., Milloy, M. J., Pick, N., Money, D., Webster, K., Hogg, R. S., de Pokomandy, A., Loutfy, M., & Kaida, A. (2018). Attrition Across the HIV Cascade of Care Among a Diverse Cohort of Women Living With HIV in Canada. Journal of acquired immune deficiency syndromes (1999), 79(2), 226–236. https://doi.org/10.1097/QAI.0000000000001775
11	Lourenço et al 2014	Lourenço, L., Colley, G., Nosyk, B., Shopin, D., Montaner, J. S., Lima, V. D., & STOP HIV/AIDS Study Group (2014). High levels of heterogeneity in the HIV cascade of care across different population subgroups in British Columbia, Canada. PloS one, 9(12), e115277. https://doi.org/10.1371/journal.pone.0115277
12	Marsh et al 2019	Marsh, K., Eaton, J. W., Mahy, M., Sabin, K., Autenrieth, C. S., Wanyeki, I., Daher, J., & Ghys, P. D. (2019). Global, regional and country-level 90-90-90 estimates for 2018: assessing progress towards the 2020 target. AIDS (London, England), 33 Suppl 3(Suppl 3), S213–S226. https://doi.org/10.1097/QAD.0000000000002355
13	Muddu et al 2019	Muddu, M., Tusubira, A. K., Sharma, S. K., Akiteng, A. R., Ssinabulya, I., & Schwartz, J. I. (2019). Integrated Hypertension and HIV Care Cascades in an HIV Treatment Program in Eastern Uganda: A Retrospective Cohort Study. Journal of acquired immune deficiency syndromes (1999), 81(5), 552–561. https://doi.org/10.1097/QAI.0000000000002067
14	Nom et al 2020	Nom, N. A. M., Kyaw, K. W. Y., Kumar, A. M. V., Hone, S., Thida, T., Nwe, T. W., Soan, P., Htun, T., & Oo, H. N. (2020). HIV Care Cascade among Prisoners of the Mandalay Central Prison in Myanmar: 2011-2018. Tropical medicine and infectious disease, 5(1), 4. https://doi.org/10.3390/tropicalmed5010004
15	Stevenson et al 2020	Stevenson, K. A., Podewils, L. J., Zishiri, V. K., Castro, K. G., & Charalambous, S. (2020). HIV prevalence and the cascade of care in five South African correctional facilities. PloS one, 15(7), e0235178. https://doi.org/10.1371/journal.pone.0235178
16	McClarty et al 2021	McClarty, L. M., Kasper, K., Ireland, L., Loeppky, C., Blanchard, J. F., & Becker, M. L. (2021). The HIV care cascade in Manitoba, Canada: Methods, measures, and estimates to meet local needs. Journal of clinical

Paper Number	Authors	Reference
		epidemiology, 132, 26–33. https://doi.org/10.1016/j.jclinepi.2020.11.026
17	Shanaube et al 2020	Shanaube, K., Macleod, D., Chaila, M. J., Mackworth-Young, C., Hoddinott, G., Schaap, A., Floyd, S., Bock, P., Hayes, R., Fidler, S., & Ayles, H. (2021). HIV Care Cascade Among Adolescents in a "Test and Treat" Community-Based Intervention: HPTN 071 (PopART) for Youth Study. The Journal of adolescent health : official publication of the Society for Adolescent Medicine, 68(4), 719–727. https://doi.org/10.1016/j.jadohealth.2020.07.029
18	Burgos-soto et al 2020	Burgos-Soto, J., Ben Farhat, J., Alley, I., Ojuka, P., Mulogo, E., Kise-Sete, T., Bouhenia, M., Salumu, L., Mathela, R., Langendorf, C., Cohuet, S., & Huerga, H. (2020). HIV epidemic and cascade of care in 12 east African rural fishing communities: results from a population-based survey in Uganda. BMC public health, 20(1), 970. https://doi.org/10.1186/s12889-020-09121-6
19	Rwema et al 2020	Twahirwa Rwema, J. O., Lyons, C. E., Herbst, S., Liestman, B., Nyombayire, J., Ketende, S., Mazzei, A., Olawore, O., Nsanzimana, S., Mugwaneza, P., Kagaba, A., Sullivan, P. S., Allen, S., Karita, E., & Baral, S. D. (2020). HIV infection and engagement in HIV care cascade among men who have sex with men and transgender women in Kigali, Rwanda: a cross-sectional study. Journal of the International AIDS Society, 23 Suppl 6(Suppl 6), e25604. https://doi.org/10.1002/jia2.25604
20	Fern'andez-Luis et al 2020	Fernández-Luis, S., Nhampossa, T., Fuente-Soro, L., Augusto, O., Casellas, A., Bernardo, E., Ruperez, M., Gonzalez, R., Maculuve, S., Saura-Lázaro, A., Menendez, C., Naniche, D., & Lopez-Varela, E. (2020). Pediatric HIV Care Cascade in Southern Mozambique: Missed Opportunities for Early ART and Re-engagement in Care. The Pediatric infectious disease journal, 39(5), 429–434. https://doi.org/10.1097/INF.0000000000002612
21	Marukutira et al 2020	Marukutira, T., Gray, R. T., Douglass, C., El-Hayek, C., Moreira, C., Asselin, J., Donovan, B., Vickers, T., Spelman, T., Crowe, S., Guy, R., Stoove, M., & Hellard, M. (2020). Gaps in the HIV diagnosis and care cascade for migrants in Australia, 2013-2017: A cross-sectional study. PLoS medicine, 17(3), e1003044. https://doi.org/10.1371/journal.pmed.1003044
22	Conan et al 2021	Conan, N., Paye, C. P., Ortuno, R., Chijuwa, A., Chiwandira, B., Goemaere, E., Belen Garone, D., Coulborn, R. M., Chihana, M., & Maman, D. (2021). What gaps remain in the HIV cascade of care? Results of a population-based survey in Nsanje District, Malawi. PloS one, 16(4), e0248410. https://doi.org/10.1371/journal.pone.0248410

Paper Number	Authors	Reference
23	Elgalib et al 2021	Elgalib, A., Shah, S., Al-Wahaibi, A., Al-Habsi, Z., Al-Fouri, M., Lau, R., Al-Kindi, H., Al-Rawahi, B., & Al-Abri, S. (2021). Disparities between HIV patient subgroups in Oman: An analysis of the 2019 cascade of care. PloS one, 16(7), e0254474. https://doi.org/10.1371/journal.pone.0254474
24	Januraga et al 2018	Januraga, P. P., Reekie, J., Mulyani, T., Lestari, B. W., Iskandar, S., Wisaksana, R., Kusmayanti, N. A., Subronto, Y. W., Widyanthini, D. N., Wirawan, D. N., Wongso, L. V., Sudewo, A. G., Sukmaningrum, E., Nisa, T., Prabowo, B. R., Law, M., Cooper, D. A., & Kaldor, J. M. (2018). The cascade of HIV care among key populations in Indonesia: a prospective cohort study. The lancet. HIV, 5(10), e560–e568. https://doi.org/10.1016/S2352-3018(18)30148-6
26	Hawk et al 2019	Hawk, M., Maulsby, C., Enobun, B., Kinsky, S., & AIDS United Retention in Care Intervention Team (2019). HIV Treatment Cascade by Housing Status at Enrollment: Results from a Retention in Care Cohort. AIDS and behavior, 23(3), 765–775. https://doi.org/10.1007/s10461-018-2295-y
27	McAllister et al 2020	McAllister, S., van Asten, H., Anglemyer, A., Crengle, S., Zeng, J., Raymond, N., Handy, R., Giola, M., Dickson, N., & Priest, P. (2021). Cascade of care of people diagnosed with HIV in New Zealand between 2006 and 2017. HIV medicine, 22(2), 122–130. https://doi.org/10.1111/hiv.12983
28	Volger et al 2018	Vogler, I. H., Alfieri, D. F., Gianjacomo, H. D. B., Almeida, E. R. D., & Reiche, E. M. V. (2018). Cascade of care for people living with HIV infection in Southern Brazil: results from a public health network. Cadernos de saude publica, 34(12), e00009718. https://doi.org/10.1590/0102-311X00009718
29	Sazonova et al 2020	Sazonova, Y., Kulchynska, R., Sereda, Y., Azarskova, M., Novak, Y., Saliuk, T., Kornilova, M., Liulchuk, M., Vitek, C., & Dumchev, K. (2020). HIV treatment cascade among people who inject drugs in Ukraine. PloS one, 15(12), e0244572. https://doi.org/10.1371/journal.pone.0244572
30	Puryear et al 2020	Puryear, S. B., Balzer, L. B., Ayieko, J., Kwarisiima, D., Hahn, J. A., Charlebois, E. D., Clark, T. D., Cohen, C. R., Bukusi, E. A., Kamya, M. R., Petersen, M. L., Havlir, D. V., & Chamie, G. (2020). Associations between alcohol use and HIV care cascade outcomes among adults undergoing population-based HIV testing in East Africa. AIDS (London, England), 34(3), 405–413. https://doi.org/10.1097/QAD.0000000000002427
31	Ho et al 2019	Ho, Z. J. M., Huang, F., Wong, C. S., Chua, L., Ma, S., Chen, M. I., & Lee, V. J. (2019). Using a HIV registry to develop accurate estimates for the HIV care cascade - the Singapore experience. Journal of the International AIDS Society, 22(7), e25356. https://doi.org/10.1002/jia2.25356

Paper Number	Authors	Reference
32	Manne-Goehler et al 2019	Manne-Goehler, J., Siedner, M. J., Montana, L., Harling, G., Geldsetzer, P., Rohr, J., Gómez-Olivé, F. X., Goehler, A., Wade, A., Gaziano, T., Kahn, K., Davies, J. I., Tollman, S., & Bärnighausen, T. W. (2019). Hypertension and diabetes control along the HIV care cascade in rural South Africa. Journal of the International AIDS Society, 22(3), e25213. https://doi.org/10.1002/jia2.25213
33	Fuente-Soro et al 2019	Fuente-Soro, L., Iniesta, C., López-Varela, E., Cuna, M., Guilaze, R., Maixenchs, M., Bernardo, E. L., Augusto, O., Gonzalez, R., Couto, A., Munguambe, K., & Naniche, D. (2019). Tipping the balance towards long-term retention in the HIV care cascade: A mixed methods study in southern Mozambique. PloS one, 14(9), e0222028. https://doi.org/10.1371/journal.pone.0222028
34	Edun et al 2016	Edun, B., Iyer, M., Albrecht, H., & Weissman, S. (2017). The South Carolina rural-urban HIV continuum of care. AIDS care, 29(7), 817–822. https://doi.org/10.1080/09540121.2016.1270397
35	O'laughlin et al 2017	O'Laughlin, K. N., Kasozi, J., Rabideau, D. J., Parker, R. A., Mulogo, E., Faustin, Z. M., Greenwald, K. E., Doraiswamy, S., Walensky, R. P., & Bassett, I. V. (2017). The cascade of HIV care among refugees and nationals in Nakivale Refugee Settlement in Uganda. HIV medicine, 18(7), 513–518. https://doi.org/10.1111/hiv.12476
37	Barrow et al 2019	Barrow, G. J., & Brandeau, M. L. (2019). A modified HIV continuum of care: A six-year evaluation of a viral load cascade at a hospital-based clinic in Kingston, Jamaica. International journal of STD & AIDS, 30(8), 748–755. https://doi.org/10.1177/0956462419839514
38	Ikeda et al 2018	Ikeda, D. J., Hollander, L., Weigl, S., Sawicki, S. V., Belanger, D. R., West, N. Y., Brey Magnani, N., Wells, C. G., Gordon, P., Morne, J., & Agins, B. D. (2018). The Facility-Level HIV Treatment Cascade: Using a Population Health Tool in Health Care Facilities to End the Epidemic in New York State. Open forum infectious diseases, 5(10), ofy254. https://doi.org/10.1093/ofid/ofy254
39	Keen et al 2018	Keen, P., Gray, R. T., Telfer, B., Guy, R., Schmidt, H. M., Whittaker, B., Holden, J., Holt, M., Kelleher, A., Wilson, D., Callander, D., Cooper, D. A., Prestage, G., Selvey, C., Grulich, A. E., & NSW HIV Prevention Partnership Project (2018). The 2016 HIV diagnosis and care cascade in New South Wales, Australia: meeting the UNAIDS 90-90-90 targets. Journal of the International AIDS Society, 21(4), e25109. https://doi.org/10.1002/jia2.25109
40	Kowalska et al 2018	Kowalska, J. D., Ankiersztejn-Bartczak, M., Shepherd, L., & Mocroft, A. (2018). Cascade of care and factors associated with virological suppression among HIV-positive persons linked to care in the Test and Keep in Care (TAK) project. Infection, 46(4), 533–540. https://doi.org/10.1007/s15010-018-1154-0

Paper Number	Authors	Reference
41	Haghighat et al 2021	Haghighat, R., Toska, E., Bungane, N., & Cluver, L. (2021). The HIV care cascade for adolescents initiated on antiretroviral therapy in a health district of South Africa: a retrospective cohort study. BMC infectious diseases, 21(1), 60. https://doi.org/10.1186/s12879-020-05742-9
42	Zaller et al 2017	Zaller, N., Gordon, M., Bazerman, L., Kuo, I., & Beckwith, C. (2017). The HIV Care Cascade Among Individuals Under Community Supervision in Baltimore, Maryland. Journal of correctional health care : the official journal of the National Commission on Correctional Health Care, 23(3), 305–312. https://doi.org/10.1177/1078345817709285
43	Larmarange et al 2018	Larmarange, J., Diallo, M. H., McGrath, N., Iwuji, C., Plazy, M., Thiébaut, R., Tanser, F., Bärnighausen, T., Pillay, D., Dabis, F., Orne-Gliemann, J., & ANRS 12249 TasP Study Group (2018). The impact of population dynamics on the population HIV care cascade: results from the ANRS 12249 Treatment as Prevention trial in rural KwaZulu-Natal (South Africa). Journal of the International AIDS Society, 21 Suppl 4(Suppl Suppl 4), e25128. https://doi.org/10.1002/jia2.25128
44	Idrisov et al 2017	Idrisov, B., Lunze, K., Cheng, D. M., Blokhina, E., Gnatienko, N., Quinn, E., Bridden, C., Walley, A. Y., Bryant, K. J., Lioznov, D., Krupitsky, E., & Samet, J. H. (2017). Role of substance use in HIV care cascade outcomes among people who inject drugs in Russia. Addiction science & clinical practice, 12(1), 30. https://doi.org/10.1186/s13722-017-0098-5
45	Engelhard et al 2016	Engelhard, E. A., Smit, C., Van Sighem, A., Reiss, P., Nieuwkerk, P. T., Kroon, F. P., Brinkman, K., & Geerlings, S. E. (2016). Impact of HIV care facility characteristics on the cascade of care in HIV-infected patients in the Netherlands. AIDS (London, England), 30(2), 301–310. https://doi.org/10.1097/QAD.0000000000000938
46	Campbell et al 2015	Campbell, C. N., Ambrosioni, J., Miro, J. M., Esteve, A., Casabona, J., Navarro, G., García, I., Ferrer, E., Force, L., & Tural, C. (2015). The continuum of HIV care in Catalonia. AIDS care, 27(12), 1449–1454. https://doi.org/10.1080/09540121.2015.1109584
47	Rocha et al 2020	Rocha, A. B. M. D., Barros, C., Generoso, I. P., Bastos, F. I., & Veras, M. A. (2020). HIV continuum of care among trans women and travestis living in São Paulo, Brazil. Revista de saude publica, 54, 118. https://doi.org/10.11606/s1518-8787.2020054002374
48	Goldenberg et al 2019	Goldenberg, S. M., Muzaaya, G., Akello, M., Braschel, M., Birungi, J., & Shannon, K. (2019). High burden of previously undiagnosed HIV infections and gaps in HIV care cascade for conflict-affected female sex workers in northern Uganda. International journal of STD & AIDS, 30(3), 275–283. https://doi.org/10.1177/0956462418804658

Paper Number	Authors	Reference
49	Raymond et al 2016	Raymond, N., Bargh, K., Aung, K. L., & Rice, J. (2016). Cascade of care for people living with HIV infection in the Wellington region. The New Zealand medical journal, 129(1432), 41–51.
50	McNairy et al 2015	McNairy, M. L., Lamb, M. R., Abrams, E. J., Elul, B., Sahabo, R., Hawken, M. P., Mussa, A., Zwede, A., Justman, J., El-Sadr, W. M., & Identifying Optimal Models of HIV Care and Treatment in Sub-Saharan Africa Study (2015). Use of a Comprehensive HIV Care Cascade for Evaluating HIV Program Performance: Findings From 4 Sub-Saharan African Countries. Journal of acquired immune deficiency syndromes (1999), 70(2), e44–e51. https://doi.org/10.1097/QAI.0000000000000745
51	Lippman et al 2019	Lippman, S. A., El Ayadi, A. M., Grignon, J. S., Puren, A., Liegler, T., Venter, W. D. F., Ratlhagana, M. J., Morris, J. L., Naidoo, E., Agnew, E., Barnhart, S., & Shade, S. B. (2019). Improvements in the South African HIV care cascade: findings on 90-90-90 targets from successive population-representative surveys in North West Province. Journal of the International AIDS Society, 22(6), e25295. https://doi.org/10.1002/jia2.25295
52	McGettrick et al 2017	McGettrick, P., Ghavami-Kia, B., Tinago, W., Macken, A., O'Halloran, J., Lambert, J. S., Sheehan, G., & Mallon, P. W. G. (2017). The HIV Care Cascade and sub-analysis of those linked to but not retained in care: the experience from a tertiary HIV referral service in Dublin Ireland. HIV clinical trials, 18(3), 93–99. https://doi.org/10.1080/15284336.2017.1298317
53	Castro et al 2016	Castro, R., Ribeiro-Alves, M., Corrêa, R. G., Derrico, M., Lemos, K., Grangeiro, J. R., Jesus, B.d, Pires, D., Veloso, V. G., & Grinsztejn, B. (2016). The Men Who Have Sex with Men HIV Care Cascade in Rio de Janeiro, Brazil. PloS one, 11(6), e0157309. https://doi.org/10.1371/journal.pone.0157309
54	Cowan et al 2017	Cowan, F. M., Davey, C. B., Fearon, E., Mushati, P., Dirawo, J., Cambiano, V., Napierala Mavedzenge, S., Hanisch, D., Wong-Gruenwald, R., Chemhuru, M., Masuka, N., Hatzold, K., Mugurungi, O., Busza, J., Philips, A. N., & Hargreaves, J. R. (2017). The HIV Care Cascade Among Female Sex Workers in Zimbabwe: Results of a Population-Based Survey From the Sisters Antiretroviral Therapy Programme for Prevention of HIV, an Integrated Response (SAPPH-IRe) Trial. Journal of acquired immune deficiency syndromes (1999), 74(4), 375–382. https://doi.org/10.1097/QAI.0000000000001255
57	Schwartz et al 2016	Schwartz, S., Lambert, A., Phaswana-Mafuya, N., Kose, Z., Mcingana, M., Holland, C., Ketende, S., Yah, C., Sweitzer, S., Hausler, H., & Baral, S. (2017). Engagement in the HIV care cascade and barriers to antiretroviral therapy uptake among female sex workers in Port Elizabeth, South Africa: findings from a respondent-driven sampling study. Sexually transmitted infections,

Paper Number	Authors	Reference
		93(4), 290–296. https://doi.org/10.1136/sextrans-2016-052773
58	Tarigan et al 2020	Tarigan, Y. N., Woodman, R. J., Miller, E. R., Wisaksana, R., Wignall, F. S., & Ward, P. R. (2020). Changes in the HIV continuum of care following expanded access to HIV testing and treatment in Indonesia: A retrospective population-based cohort study. PloS one, 15(9), e0239041. https://doi.org/10.1371/journal.pone.0239041
59	Laisaae et al 2016	Laisaar, K. T., Raag, M., Lutsar, I., & Uusküla, A. (2016). People living with HIV in Estonia: engagement in HIV care in 2013. Euro surveillance : bulletin Europeen sur les maladies transmissibles = European communicable disease bulletin, 21(43), 30380. https://doi.org/10.2807/1560-7917.ES.2016.21.43.30380
60	Ruadze et al 2017	Ruadze, E., Chkhartishvili, N., Chokoshvili, O., & Tsertsvadze, T. (2017). Cascade of care among HIV patients diagnosed in 2013 in Georgia: Risk factors for late diagnosis and attrition from HIV care. SAGE open medicine, 5, 2050312117731977. https://doi.org/10.1177/2050312117731977
61	Elgalib et al 2020	Elgalib, A., Shah, S., Al-Habsi, Z., Al-Fouri, M., Lau, R., Al-Kindi, H., Al-Rawahi, B., & Al-Abri, S. (2020). The cascade of HIV care in Oman, 2015-2018: A population-based study from the Middle East. International journal of infectious diseases : IJID : official publication of the International Society for Infectious Diseases, 90, 28–34. https://doi.org/10.1016/j.ijid.2019.09.017
65	Auld et al 2017	Auld, A. F., Valerie Pelletier, Robin, E. G., Shiraishi, R. W., Dee, J., Antoine, M., Desir, Y., Desforges, G., Delcher, C., Duval, N., Joseph, N., Francois, K., Griswold, M., Domercant, J. W., Patrice Joseph, Y. A., Van Onacker, J. D., Deyde, V., Lowrance, D. W., & And The Groupe d'Analyses Salvh (2017). Retention Throughout the HIV Care and Treatment Cascade: From Diagnosis to Antiretroviral Treatment of Adults and Children Living with HIV-Haiti, 1985-2015. The American journal of tropical medicine and hygiene, 97(4_Suppl), 57–70. https://doi.org/10.4269/ajtmh.17-0116
66	Lurie et al 2020	Lurie, M. N., Kirwa, K., Callaway, J., Cornell, M., Boulle, A., Bengtson, A. M., Smith, M., Leon, N., & Colvin, C. (2020). Quantifying the HIV treatment cascade in a South African health sub-district by gender: retrospective cohort study. Tropical medicine & international health : TM & IH, 25(2), 186–192. https://doi.org/10.1111/tmi.13334

Paper Number	Authors	Reference
68	Chung et al 2017	Chung, N. C., Bolton-Moore, C., Chilengi, R., Kasaro, M. P., Stringer, J. S., & Chi, B. H. (2017). Patient engagement in HIV care and treatment in Zambia, 2004-2014. Tropical medicine & international health : TM & IH, 22(3), 332–339. https://doi.org/10.1111/tmi.12832
69	Drew et al 2017	Drew, R. S., Rice, B., Rüütel, K., Delpech, V., Attawell, K. A., Hales, D. K., Velasco, C., Amato-Gauci, A. J., Pharris, A., Tavoschi, L., & Noori, T. (2017). HIV continuum of care in Europe and Central Asia. HIV medicine, 18(7), 490–499. https://doi.org/10.1111/hiv.12480
71	Conan et al 2020	Conan, N., Coulborn, R. M., Simons, E., Mapfumo, A., Apollo, T., Garone, D. B., Casas, E. C., Puren, A. J., Chihana, M. L., & Maman, D. (2020). Successes and gaps in the HIV cascade of care of a high HIV prevalence setting in Zimbabwe: a population-based survey. Journal of the International AIDS Society, 23(9), e25613. https://doi.org/10.1002/jia2.25613
72	Ostermann et al 2015	Ostermann, J., Pence, B., Whetten, K., Yao, J., Itemba, D., Maro, V., Reddy, E., & Thielman, N. (2015). HIV serostatus disclosure in the treatment cascade: evidence from Northern Tanzania. AIDS care, 27 Suppl 1(sup1), 59–64. https://doi.org/10.1080/09540121.2015.1090534
73	Ghalehkhani et al 2019	Ghalehkhani, N., Farhoudi, B., Gouya, M. M., Sharifi, H., SeyedAlinaghi, S., Kamali, K., Fahimfar, N., Rajabpour, Z., Doosti-Irani, A., Sedaghat, A., & Mirzazadeh, A. (2019). The HIV treatment cascade in people living with HIV in Iran in 2014: Mixed-method study to measure losses and reasons. International journal of STD & AIDS, 30(13), 1257–1264. https://doi.org/10.1177/0956462419867573
75	Chkhartishvili et al 2015	Chkhartishvili, N., Sharavdze, L., Chokoshvili, O., DeHovitz, J. A., del Rio, C., & Tsertsvadze, T. (2015). The cascade of care in the Eastern European country of Georgia. HIV medicine, 16(1), 62–66. https://doi.org/10.1111/hiv.12172
76	Forbes et al 2016	Forbes, N., Johnson, G., Mortimer, A., Martin, I., Frankson, M., Johnston, K., Thompson, T., & Weissman, S. (2016). The HIV continuum of care in the Bahamas in 2014. Revista panamericana de salud publica = Pan American journal of public health, 40(6), 443–447.
77	Horberg et al 2015	Horberg, M. A., Hurley, L. B., Klein, D. B., Towner, W. J., Kadlecik, P., Antoniskis, D., Mogyoros, M., Brachman, P. S., Remmers, C. L., Gambatese, R. C., Blank, J., Ellis, C. G., & Silverberg, M. J. (2015). The HIV Care Cascade Measured Over Time and by Age, Sex, and Race in a Large National Integrated Care System. AIDS patient care and STDs, 29(11), 582–590. https://doi.org/10.1089/apc.2015.0139

Paper Number	Authors	Reference
78	Edwards et al 2019	Edwards, J. K., Arimi, P., Ssengooba, F., Mulholland, G., Markiewicz, M., Bukusi, E. A., Orikiiriza, J. T., Virkud, A., & Weir, S. (2019). The HIV care continuum among resident and non-resident populations found in venues in East Africa cross-border areas. Journal of the International AIDS Society, 22(1), e25226. https://doi.org/10.1002/jia2.25226
79	Alvarez-Uria et al 2013	Alvarez-Uria, G., Pakam, R., Midde, M., & Naik, P. K. (2013). Entry, Retention, and Virological Suppression in an HIV Cohort Study in India: Description of the Cascade of Care and Implications for Reducing HIV-Related Mortality in Low- and Middle-Income Countries. Interdisciplinary perspectives on infectious diseases, 2013, 384805. https://doi.org/10.1155/2013/384805
80	Napierala et al 2018	Napierala, S., Chabata, S. T., Fearon, E., Davey, C., Hargreaves, J., Busza, J., Mushati, P., Mtetwa, S., Chiyaka, T., Mugurungi, O., Hanisch, D., Hatzold, K., Phillips, A., & Cowan, F. M. (2018). Engagement in HIV Care Among Young Female Sex Workers in Zimbabwe. Journal of acquired immune deficiency syndromes (1999), 79(3), 358–366. https://doi.org/10.1097/QAI.0000000000001815
81	Wester et al 2016	Wester, C., Rebeiro, P. F., Shavor, T. J., Shepherd, B. E., McGoy, S. L., Daley, B., Morrison, M., Vermund, S. H., & Pettit, A. C. (2016). The 2013 HIV Continuum of Care in Tennessee: Progress Made, but Disparities Persist. Public health reports (Washington, D.C. : 1974), 131(5), 695–703. https://doi.org/10.1177/0033354916660082
82	Heimer et al 2017	Heimer, R., Usacheva, N., Barbour, R., Niccolai, L. M., Uusküla, A., & Levina, O. S. (2017). Engagement in HIV care and its correlates among people who inject drugs in St Petersburg, Russian Federation and Kohtla-Järve, Estonia. Addiction (Abingdon, England), 112(8), 1421–1431. https://doi.org/10.1111/add.13798
83	Gebreegziabher et al 2020	Gebreegziabher, E. A., McCoy, S. I., Ycasas, J. C., & Murgai, N. (2020). The Role of Neighborhood Poverty in the Association between Foreign-Born status and HIV Care Continuum Outcomes in Alameda County, California. Journal of immigrant and minority health, 22(5), 1023–1030. https://doi.org/10.1007/s10903-020-01002-9
84	Davoglio et al 2021	Davoglio, R. S., Gandin, H., & Mocellin, L. P. (2021). HIV/AIDS epidemic in a western border municipality of Rio Grande do Sul, Brazil: evolution, HIV cascade of care and lethality. Revista brasileira de epidemiologia = Brazilian journal of epidemiology, 24(suppl 1), e210018. https://doi.org/10.1590/1980-549720210018.supl.1

Paper Number	Authors	Reference
85	Muth et al 2017	Muth, S., Len, A., Evans, J. L., Phou, M., Chhit, S., Neak, Y., Ngak, S., Stein, E. S., Carrico, A. W., Maher, L., & Page, K. (2017). HIV treatment cascade among female entertainment and sex workers in Cambodia: impact of amphetamine use and an HIV prevention program. Addiction science & clinical practice, 12(1), 20. https://doi.org/10.1186/s13722-017-0085-x
86	Boothe et al 2021	Boothe, M. A. S., Sathane, I., Baltazar, C. S., Chicuecue, N., Horth, R., Fazito, E., & Raymond, H. F. (2021). Low engagement in HIV services and progress through the treatment cascade among key populations living with HIV in Mozambique: alarming gaps in knowledge of status. BMC public health, 21(1), 146. https://doi.org/10.1186/s12889-020-10039-2
87	Jalil et al 2017	Jalil, E. M., Wilson, E. C., Luz, P. M., Velasque, L., Moreira, R. I., Castro, C. V., Monteiro, L., Garcia, A. C. F., Cardoso, S. W., Coelho, L. E., McFarland, W., Liu, A. Y., Veloso, V. G., Buchbinder, S., & Grinsztejn, B. (2017). HIV testing and the care continuum among transgender women: population estimates from Rio de Janeiro, Brazil. Journal of the International AIDS Society, 20(1), 21873. https://doi.org/10.7448/IAS.20.1.21873
88	Billioux et al 2017	Billioux, V. G., Chang, L. W., Reynolds, S. J., Nakigozi, G., Ssekasanvu, J., Grabowski, M. K., Ssekubugu, R., Nalugoda, F., Kigozi, G., Kagaayi, J., Serwadda, D., Gray, R. H., & Wawer, M. J. (2017). Human immunodeficiency virus care cascade among sub-populations in Rakai, Uganda: an observational study. Journal of the International AIDS Society, 20(1), 21590. https://doi.org/10.7448/IAS.20.1.21590
90	Chowers et al 2020	Chowers, M., Chemtob, D., Mor, O., Levy, I., Elbirt, D., Elinav, H., Rizenberg, K., Lorber, M., Istomin, V., Nemet, S., Shahak, G., & Turner, D. (2020). Continuum of HIV care of newly diagnosed individuals in Israel, 2011-2015: a population-based cohort study. International journal of STD & AIDS, 31(4), 326–334. https://doi.org/10.1177/0956462419891023
91	De Boni et al 2018	De Boni, R. B., Peratikos, M. B., Shepherd, B. E., Grinsztejn, B., Cortés, C., Padgett, D., Gotuzzo, E., Belaunzarán-Zamudio, P. F., Rebeiro, P. F., Duda, S. N., McGowan, C. C., & for CCASAnet (2018). Is substance use associated with HIV cascade outcomes in Latin America?. PloS one, 13(3), e0194228. https://doi.org/10.1371/journal.pone.0194228
92	Ndudzo et al 2019	Ndudzo, C., Tripathy, J. P., Tauro, F., Sibanda, C., Chiramba, M., Shamu, A., Masinire, K., Muchengwa, T., & Kumar, A. M. (2019). HIV care among patients with presumptive tuberculosis in Masvingo district of Zimbabwe, 2017: how well are we doing?. The Pan African medical journal, 33, 158. https://doi.org/10.11604/pamj.2019.33.158.15847

Paper Number	Authors	Reference
93	Koirala et al 2017	Koirala, S., Deuba, K., Nampaisan, O., Marrone, G., Ekström, A. M., & CAT-S group (2017). Facilitators and barriers for retention in HIV care between testing and treatment in Asia-A study in Bangladesh, Indonesia, Lao, Nepal, Pakistan, Philippines and Vietnam. PloS one, 12(5), e0176914. https://doi.org/10.1371/journal.pone.0176914
94	Alvarez-Uria et al 2014	Alvarez-Uria G. (2014). Description of the cascade of care and factors associated with attrition before and after initiating antiretroviral therapy of HIV infected children in a cohort study in India. PeerJ, 2, e304. https://doi.org/10.7717/peerj.304
95	Wolff et al 2018	Wolff, M. J., Cortes, C. P., Mejìa, F. A., Padgett, D., Belaunzarán-Zamudio, P., Grinsztejn, B., Giganti, M. J., McGowan, C. C., Rebeiro, P. F., & Caribbean, Central and South America network for HIV epidemiology (CCASAnet) (2018). Evaluating the care cascade after antiretroviral therapy initiation in Latin America. International journal of STD & AIDS, 29(1), 4–12. https://doi.org/10.1177/0956462417714094
96	Reyes-uruena et al 2017	Reyes-Urueña, J., Campbell, C., Hernando, C., Vives, N., Folch, C., Ferrer, L., Fernández-López, L., Esteve, A., & Casabona, J. (2017). Differences between migrants and Spanish-born population through the HIV care cascade, Catalonia: an analysis using multiple data sources. Epidemiology and infection, 145(8), 1670–1681. https://doi.org/10.1017/S0950268817000437
97	Santos et al 2014	Santos, G. M., Wilson, E. C., Rapues, J., Macias, O., Packer, T., & Raymond, H. F. (2014). HIV treatment cascade among transgender women in a San Francisco respondent driven sampling study. Sexually transmitted infections, 90(5), 430–433. https://doi.org/10.1136/sextrans-2013-051342
98	Wechsberg et al 2017	Wechsberg, W. M., van der Horst, C., Ndirangu, J., Doherty, I. A., Kline, T., Browne, F. A., Belus, J. M., Nance, R., & Zule, W. A. (2017). Seek, test, treat: substance-using women in the HIV treatment cascade in South Africa. Addiction science & clinical practice, 12(1), 12. https://doi.org/10.1186/s13722-017-0077-x
99	Jaries et al 2017	Jaries, R., Vantilcke, V., Clevenbergh, P., Adoissi, J., Boukhari, R., Misslin, C., Nacher, M., Vreden, S., & Jolivet, A. (2017). Population movements and the HIV cascade in recently diagnosed patients at the French Guiana -Suriname border. AIDS care, 29(11), 1448–1452. https://doi.org/10.1080/09540121.2017.1291899
100	Kelly et al 2016	Kelly, J. D., Schlough, G. W., Conteh, S., Barrie, M. B., Kargbo, B., & Giordano, T. P. (2016). The Majority of the Pre-Antiretroviral Population Who Were Lost to Follow-Up Stopped Their Care in Freetown, Sierra Leone: A 12-Month Prospective Cohort Study Starting with HIV Diagnosis. PloS one, 11(2), e0149584. https://doi.org/10.1371/journal.pone.0149584

Paper Number	Authors	Reference
101	Medland et al 2017	Medland, N. A., Chow, E. P. F., McMahon, J. H., Elliott, J. H., Hoy, J. F., & Fairley, C. K. (2017). Time from HIV diagnosis to commencement of antiretroviral therapy as an indicator to supplement the HIV cascade: Dramatic fall from 2011 to 2015. PloS one, 12(5), e0177634. https://doi.org/10.1371/journal.pone.0177634
102	Kalawan et al 2020	Kalawan, V., Naidoo, K., & Archary, M. (2020). Impact of routine birth early infant diagnosis on neonatal HIV treatment cascade in eThekwini district, South Africa. Southern African journal of HIV medicine, 21(1), 1084. https://doi.org/10.4102/sajhivmed.v21i1.1084
103	Gissl'en et al 2017	Gisslén, M., Svedhem, V., Lindborg, L., Flamholc, L., Norrgren, H., Wendahl, S., Axelsson, M., & Sönnerborg, A. (2017). Sweden, the first country to achieve the Joint United Nations Programme on HIV/AIDS (UNAIDS)/World Health Organization (WHO) 90-90-90 continuum of HIV care targets. HIV medicine, 18(4), 305–307. https://doi.org/10.1111/hiv.12431
104	Maman et al 2016	Maman, D., Chilima, B., Masiku, C., Ayouba, A., Masson, S., Szumilin, E., Peeters, M., Ford, N., Heinzelmann, A., Riche, B., & Etard, J. F. (2016). Closer to 90-90-90. The cascade of care after 10 years of ART scale-up in rural Malawi: a population study. Journal of the International AIDS Society, 19(1), 20673. https://doi.org/10.7448/IAS.19.1.20673
106	Jin et al 2018	Jin, H., Restar, A., Biello, K., Kuhns, L., Reisner, S., Garofalo, R., & Mimiaga, M. J. (2019). Burden of HIV among young transgender women: factors associated with HIV infection and HIV treatment engagement. AIDS care, 31(1), 125–130. https://doi.org/10.1080/09540121.2018.1539213
107	Krentz et al 2015	Krentz, H. B., MacDonald, J., & Gill, M. J. (2015). The impact of transfer patients on the local cascade of HIV care continuum. Journal of acquired immune deficiency syndromes (1999), 68(2), 236–240. https://doi.org/10.1097/QAI.0000000000000430
108	Marukutira et al 2019	Marukutira, T., Yin, D., Cressman, L., Kariuki, R., Malone, B., Spelman, T., Mawandia, S., Ledikwe, J. H., Semo, B. W., Crowe, S., Stoove, M., Hellard, M., & Dickinson, D. (2019). Clinical outcomes of a cohort of migrants and citizens living with human immunodeficiency virus in Botswana: implications for Joint United Nation Program on HIV and AIDS 90-90-90 targets. Medicine, 98(23), e15994. https://doi.org/10.1097/MD.0000000000015994
109	Govender et al 2020	Govender, K., Durevall, D., Cowden, R. G., Beckett, S., Kharsany, A. B., Lewis, L., George, G., Cawood, C., & Khanyile, D. (2022). Depression symptoms, HIV testing, linkage to ART, and viral suppression among women in a high HIV burden district in KwaZulu-Natal, South Africa: A cross-sectional household study. Journal of health

Paper Number	Authors	Reference
		psychology, 27(4), 936–945. https://doi.org/10.1177/1359105320982042
111	Rhead et al 2018	Rhead, R., Elmes, J., Otobo, E., Nhongo, K., Takaruza, A., White, P. J., Nyamukapa, C. A., & Gregson, S. (2018). Do female sex workers have lower uptake of HIV treatment services than non-sex workers? A cross-sectional study from east Zimbabwe. BMJ open, 8(2), e018751. https://doi.org/10.1136/bmjopen-2017-018751
112	Khumalo et al 2020	Khumalo, P. N., Sacks, E., Chouraya, C., Tsabedze, B., Masuku, T., Nyoni, G., Zikalala, T., Nhlabatsi, B., Mthethwa, N., & Cohn, J. (2020). The Cascade of Care From Routine Point-of-Care HIV Testing at Birth: Results From an 18-Months Pilot Program in Eswatini. Journal of acquired immune deficiency syndromes (1999), 84 Suppl 1(1), S22–S27. https://doi.org/10.1097/QAI.0000000000002380
113	Moore et al 2021	Moore, D. M., Cui, Z., Skakoon-Sparling, S., Sang, J., Barath, J., Wang, L., Lachowsky, N., Cox, J., Lambert, G., Noor, S. W., Grace, D., Jollimore, J., Apelian, H., Lal, A., Parlette, A., & Hart, T. A. (2021). Characteristics of the HIV cascade of care and unsuppressed viral load among gay, bisexual and other men who have sex with men living with HIV across Canada's three largest cities. Journal of the International AIDS Society, 24(4), e25699. https://doi.org/10.1002/jia2.25699
114	Fearon et al 2020	Fearon, E., Tenza, S., Mokoena, C., Moodley, K., Smith, A. D., Bourne, A., Weatherburn, P., & Palanee-Phillips, T. (2020). HIV testing, care and viral suppression among men who have sex with men and transgender individuals in Johannesburg, South Africa. PloS one, 15(6), e0234384. https://doi.org/10.1371/journal.pone.0234384
115	Rossi et al 2020	Rossi, A. D. M., Albanese, S. P. R., Vogler, I. H., Pieri, F. M., Lentine, E. C., Birolim, M. M., & Dessunti, E. M. (2020). HIV Care Continuum from diagnosis in a Counseling and Testing Center. Revista brasileira de enfermagem, 73(6), e20190680. https://doi.org/10.1590/0034-7167-2019-0680
116	Sirirungsi et al 2016	Sirirungsi, W., Khamduang, W., Collins, I. J., Pusamang, A., Leechanachai, P., Chaivooth, S., Ngo-Giang-Huong, N., & Samleerat, T. (2016). Early infant HIV diagnosis and entry to HIV care cascade in Thailand: an observational study. The lancet. HIV, 3(6), e259–e265. https://doi.org/10.1016/S2352-3018(16)00045-X

Paper Number	Authors	Reference
118	Bachanas et al 2021	Bachanas, P., Alwano, M. G., Lebelonyane, R., Block, L., Behel, S., Raizes, E., Ussery, G., Wang, H., Ussery, F., Pretorius Holme, M., Sexton, C., Pals, S., Lasry, A., Del Castillo, L., Hader, S., Lockman, S., Bock, N., & Moore, J. (2021). Finding, treating and retaining persons with HIV in a high HIV prevalence and high treatment coverage country: Results from the Botswana Combination Prevention Project. PloS one, 16(4), e0250211. https://doi.org/10.1371/journal.pone.0250211
119	Huerga et al 2018	Huerga, H., Van Cutsem, G., Ben Farhat, J., Puren, A., Bouhenia, M., Wiesner, L., Dlamini, L., Maman, D., Ellman, T., & Etard, J. F. (2018). Progress towards the UNAIDS 90-90-90 goals by age and gender in a rural area of KwaZulu-Natal, South Africa: a household-based community cross-sectional survey. BMC public health, 18(1), 303. https://doi.org/10.1186/s12889-018-5208-0
120	Vetrova et al 2018	Vetrova, M. V., Aleksandrova, O. V., Paschenko, A. E., Toropov, S. E., Rassokhin, V. V., Abyshev, R. A., Levina, O. S., Niccolai, L. M., & Heimer, R. (2018). Early stages of HIV treatment cascade in people living with HIV in Saint-Petersburg, Russia. AIDS care, 30(7), 857–862. https://doi.org/10.1080/09540121.2017.1417536
122	Bijker et al 2020	Bijker, R., Kumarasamy, N., Kiertiburanakul, S., Pujari, S., Ng, O. T., Sun, L. P., Merati, T. P., Van Nguyen, K., Lee, M. P., Cuong, D. D., Chan, Y. J., Choi, J. Y., Ross, J., Law, M., & IeDEA Asia-Pacific (2020). An expanded HIV care cascade: ART uptake, viral load suppression and comorbidity monitoring among adults living with HIV in Asia. Antiviral therapy, 25(5), 275–285. https://doi.org/10.3851/IMP3379
123	Jean et al 2016	Jean, K., Puren, A., Cutler, E., Singh, B., Bouscaillou, J., Rain-Taljaard, R., Taljaard, D., Gouws, E., Lissouba, P., Lewis, D. A., Peytavin, G., & Auvert, B. (2016). Level of viral suppression and the cascade of HIV care in a South African semi-urban setting in 2012. AIDS (London, England), 30(13), 2107–2116. https://doi.org/10.1097/QAD.0000000000001155
124	Anglemyer et al 2020	Anglemyer, A., Haber, N., Noiman, A., Rutherford, G., Ganesan, A., Blaylock, J., Okulicz, J., Maves, R. C., Lalani, T., Schofield, C., Mancuso, J., Agan, B. K., & Infectious Disease Clinical Research Program HIV Working Group (2020). HIV Care Continuum and Meeting 90-90-90 Targets: Cascade of Care Analyses of a U.S. Military Cohort. Military medicine, 185(7-8), e1147–e1154. https://doi.org/10.1093/milmed/usaa021
136	Lally et al 2018	Lally, M. A., van den Berg, J. J., Westfall, A. O., Rudy, B. J., Hosek, S. G., Fortenberry, J. D., Monte, D., Tanney, M. R., McFarland, E. J., Xu, J., Kapogiannis, B. G., Wilson, C. M., & Adolescent Medicine Trials Network for HIV/AIDS Interventions (ATN) (2018). HIV Continuum of Care for Youth in the United States. Journal of acquired

Paper Number	Authors	Reference
		immune deficiency syndromes (1999), 77(1), 110–117. https://doi.org/10.1097/QAI.0000000000001563
137	Colombe et al 2019	Colombe, S., Machemba, R., Mtenga, B., Lutonja, P., Safari, W., Beard, J., Downs, J. A., Urassa, M., Todd, J., & Changalucha, J. (2020). Cascade of care for HIV-seroconverters in rural Tanzania: a longitudinal study. AIDS care, 32(5), 666–671. https://doi.org/10.1080/09540121.2019.1640842
139	Gesesew et al 2020	Gesesew, H. A., Ward, P., Woldemichael, K., & Mwanri, L. (2020). HIV Care continuum Outcomes: Can Ethiopia Meet the UNAIDS 90-90-90 Targets?. Ethiopian journal of health sciences, 30(2), 179–188. https://doi.org/10.4314/ejhs.v30i2.5
140	Stephens et al 2021	Jacqueline H. Stephens, Richard T Gray, Rebecca Guy, Tobias Vickers & James Ward (2021): A HIV diagnosis and treatment cascade for Aboriginal and Torres Strait Islander peoples of Australia, AIDS Care, DOI: 10.1080/09540121.2021.2001416
141	Vourli et al 2018	Vourli, G., Nikolopoulos, G., Paparizos, V., Skoutelis, A., Metallidis, S., Gargalianos, P., Papadopoulos, A., Chini, M., Sipsas, N. V., Psychogiou, M., Chrysos, G., Sambatakou, H., Gogos, C., Katsarou, O., Paraskeva, D., Dedes, N., Touloumi, G., & Greek HIV Prevention Group (2018). HIV cascade of care in Greece: Useful insights from additional stages. PloS one, 13(11), e0207355. https://doi.org/10.1371/journal.pone.0207355
142	Nijhawan et al 2020	Nijhawan, A. E., Bhattatiry, M., Chansard, M., Zhang, S., & Halm, E. A. (2020). HIV care cascade before and after hospitalization: impact of a multidisciplinary inpatient team in the US South. AIDS care, 32(11), 1343–1352. https://doi.org/10.1080/09540121.2019.1698704
144	Brown et al 2018	Brown, A. E., Attawell, K., Hales, D., Rice, B. D., Pharris, A., Supervie, V., Van Beckhoven, D., Delpech, V. C., An der Heiden, M., Marcus, U., Maly, M., & Noori, T. (2018). Monitoring the HIV continuum of care in key populations across Europe and Central Asia. HIV medicine, 10.1111/hiv.12603. Advance online publication. https://doi.org/10.1111/hiv.12603
145	Johansson et al 2021	Johansson, M., Penno, C., Winqvist, N., Tesfaye, F., & Björkman, P. (2021). How does HIV testing modality impact the cascade of care among persons diagnosed with HIV in Ethiopia?. Global health action, 14(1), 1933788. https://doi.org/10.1080/16549716.2021.1933788

Paper Number	Authors	Reference
146	Meloni et al 2020	Meloni, S. T., Agaba, P., Chang, C. A., Yiltok, E., Oguche, S., Ejeliogu, E., Agbaji, O., Okonkwo, P., & Kanki, P. J. (2020). Longitudinal evaluation of adherence, retention, and transition patterns of adolescents living with HIV in Nigeria. PloS one, 15(7), e0236801. https://doi.org/10.1371/journal.pone.0236801
147	Guaraldi et al 2021	Guaraldi, G., Borghi, V., Milic, J., Carli, F., Cuomo, G., Menozzi, M., Santoro, A., Orlando, G., Puzzolante, C., Meschiari, M., Franceschini, E., Bedini, A., Ferrari, F., Gennari, W., Sarti, M., & Mussini, C. (2021). The Impact of COVID-19 on UNAIDS 90-90-90 Targets: Calls for New HIV Care Models. Open forum infectious diseases, 8(7), ofab283. https://doi.org/10.1093/ofid/ofab283
148	Wang et al 2020	Wang, G., Lu, C., Qin, S., Wei, W., Lai, J., Jiang, J., Liang, B., Zhou, O., Han, J., Yang, Y., Ye, L., Liang, H., & Ning, C. (2020). 90-90-90 cascade analysis on reported CLHIV infected by mother-to-child transmission in Guangxi, China: a modeling study. Scientific reports, 10(1), 5295. https://doi.org/10.1038/s41598-020-62281-8
150	Lacombe-Duncan et al 2019	Lacombe-Duncan, A., Bauer, G. R., Logie, C. H., Newman, P. A., Shokoohi, M., Kay, E. S., Persad, Y., O'Brien, N., Kaida, A., de Pokomandy, A., & Loutfy, M. (2019). The HIV Care Cascade Among Transgender Women with HIV in Canada: A Mixed-Methods Study. AIDS patient care and STDs, 33(7), 308–322. https://doi.org/10.1089/apc.2019.0013
151	Boerma et al 2019	Boerma, R., Schellekens, O., Rinke de Wit, T. F., Wit, F. W., van der Borght, S., Rijckborst, H., Chukwumah, P., & Schilthuis, H. (2019). Reaching 90-90-90: outcomes of a 15-year multi-country HIV workplace programme in sub-Saharan Africa. Antiviral therapy, 24(5), 363–370. https://doi.org/10.3851/IMP3311
152	Van Santen et al 2021	van Santen, D. K., Asselin, J., Haber, N. A., Traeger, M. W., Callander, D., Donovan, B., El-Hayek, C., McMahon, J. H., Petoumenos, K., McManus, H., Hoy, J. F., Hellard, M., Guy, R., Stoové, M., & TAIPAN investigators (2021). Improvements in transition times through the HIV cascade of care among gay and bisexual men with a new HIV diagnosis in New South Wales and Victoria, Australia (2012-19): a longitudinal cohort study. The lancet. HIV, 8(10), e623–e632. https://doi.org/10.1016/S2352-3018(21)00155-7
153	Comelli et al 2019	Comelli, A., Izzo, I., Donato, F., Celotti, A., Focà, E., Pezzoli, C., Castelli, F., & Quiros-Roldan, E. (2019). Disengagement and reengagement of HIV continuum of care in a single center cohort in northern Italy. HIV research & clinical practice, 20(1), 1–11. https://doi.org/10.1080/15284336.2019.1595887

Paper Number	Authors	Reference
154	Rohr et al 2019	Rohr, J. K., Manne-Goehler, J., Gómez-Olivé, F. X., Wagner, R. G., Rosenberg, M., Geldsetzer, P., Kabudula, C., Kahn, K., Tollman, S., Bärnighausen, T., & Salomon, J. A. (2020). HIV treatment cascade for older adults in rural South Africa. Sexually transmitted infections, 96(4), 271–276. https://doi.org/10.1136/sextrans-2018-053925
156	Iwamoto et al 2017	Iwamoto, A., Taira, R., Yokomaku, Y., Koibuchi, T., Rahman, M., Izumi, Y., & Tadokoro, K. (2017). The HIV care cascade: Japanese perspectives. PloS one, 12(3), e0174360. https://doi.org/10.1371/journal.pone.0174360
157	Chappell et al 2019	Chappell, E., Lyall, H., Riordan, A., Thorne, C., Foster, C., Butler, K., Prime, K., Bamford, A., Peters, H., Judd, A., Collins, I. J., & Collaborative HIV Paediatric Study Steering Committee (2019). The cascade of care for children and adolescents with HIV in the UK and Ireland, 2010 to 2016. Journal of the International AIDS Society, 22(9), e25379. https://doi.org/10.1002/jia2.25379
159	Touloumi et al 2022	Touloumi, G., Thomadakis, C., Pantazis, N., Papastamopoulos, V., Paparizos, V., Metallidis, S., Adamis, G., Chini, M., Psichogiou, M., Chrysos, G., Sambatakou, H., Barbunakis, E., Vourli, G., Antoniadou, A., & AMACS (2022). HIV continuum of care: bridging cross-sectional and longitudinal analyses. AIDS (London, England), 36(4), 583–591. https://doi.org/10.1097/QAD.0000000000003131
161	Agolory et al 2018	Agolory, S., de Klerk, M., Baughman, A. L., Sawadogo, S., Mutenda, N., Pentikainen, N., Shoopala, N., Wolkon, A., Taffa, N., Mutandi, G., Jonas, A., Mengistu, A. T., Dzinotyiweyi, E., Prybylski, D., Hamunime, N., & Medley, A. (2018). Low Case Finding Among Men and Poor Viral Load Suppression Among Adolescents Are Impeding Namibia's Ability to Achieve UNAIDS 90-90-90 Targets. Open forum infectious diseases, 5(9), ofy200. https://doi.org/10.1093/ofid/ofy200
162	Lopez-Varela et al 2021	Lopez-Varela, E., Augusto, O., Fuente-Soro, L., Sacoor, C., Nhacolo, A., Casavant, I., Karajeanes, E., Vaz, P., & Naniche, D. (2021). Quantifying the gender gap in the HIV care cascade in southern Mozambique: We are missing the men. PloS one, 16(2), e0245461. https://doi.org/10.1371/journal.pone.0245461
163	Kalichman et al 2017	Kalichman, S. C., Hernandez, D., Finneran, S., Price, D., & Driver, R. (2017). Transgender women and HIV-related health disparities: falling off the HIV treatment cascade. Sexual health, 14(5), 469–476. https://doi.org/10.1071/SH17015
164	Lopez-Varela et al 2018	Lopez-Varela, E., Fuente-Soro, L., Augusto, O. J., Sacoor, C., Nhacolo, A., Karajeanes, E., Vaz, P., & Naniche, D. (2018). Continuum of HIV Care in Rural Mozambique: The Implications of HIV Testing Modality on Linkage and Retention. Journal of acquired immune deficiency syndromes (1999), 78(5), 527–535. https://doi.org/10.1097/QAI.0000000000001720

Paper Number	Authors	Reference
165	Schiaroli et al 2020	Schiaroli, E., De Socio, G. V., Gabrielli, C., Papalini, C., Nofri, M., Baldelli, F., & Francisci, D. (2020). Partial Achievement of the 90-90-90 UNAIDS Target in a Cohort of HIV Infected Patients from Central Italy. Mediterranean journal of hematology and infectious diseases, 12(1), e2020017. https://doi.org/10.4084/MJHID.2020.017
166	Tarigan et al 2021	Tarigan, Y. N., Woodman, R. J., Miller, E. R., Wisaksana, R., & Ward, P. R. (2021). Impact of strategic use of antiretroviral therapy intervention to the HIV continuum of care in 13 cities in Indonesia: an interrupted time series analysis. AIDS research and therapy, 18(1), 22. https://doi.org/10.1186/s12981-021-00340-4
167	Edun et al 2015	Edun, B., Iyer, M., Albrecht, H., & Weissman, S. (2015). The South Carolina HIV Cascade of Care. Southern medical journal, 108(11), 670–674. https://doi.org/10.14423/SMJ.0000000000000368
168	Ssekalembe et al 2020	Ssekalembe, G., Isfandiari, M. A., & Suprianto, H. (2020). Current Status Towards 90-90-90 UNAIDS Target and Factors Associated with HIV Viral Load Suppression in Kediri City, Indonesia. HIV/AIDS (Auckland, N.Z.), 12, 47–57. https://doi.org/10.2147/HIV.S231173
169	Whittaker et al 2020	Whittaker, R., Case, K. K., Nilsen, Ø., Blystad, H., Cowan, S., Kløvstad, H., & van Sighem, A. (2020). Monitoring progress towards the first UNAIDS 90-90-90 target in key populations living with HIV in Norway. BMC infectious diseases, 20(1), 451. https://doi.org/10.1186/s12879-020-05178-1
170	Porter et al 2018	Porter, K., Gourlay, A., Attawell, K., Hales, D., Supervie, V., Touloumi, G., Rosinska, M., Vourli, G., van Sighem, A., Pharris, A., Noori, T., & ECDC Dublin Declaration Monitoring Network (2018). Substantial Heterogeneity in Progress Toward Reaching the 90-90-90 HIV Target in the WHO European Region. Journal of acquired immune deficiency syndromes (1999), 79(1), 28–37. https://doi.org/10.1097/QAI.0000000000001761
171	Ma et al 2018	Ma, Y., Dou, Z., Guo, W., Mao, Y., Zhang, F., McGoogan, J. M., Zhao, Y., Zhao, D., Wu, Y., Liu, Z., & Wu, Z. (2018). The Human Immunodeficiency Virus Care Continuum in China: 1985-2015. Clinical infectious diseases : an official publication of the Infectious Diseases Society of America, 66(6), 833–839. https://doi.org/10.1093/cid/cix911

Paper Number	Authors	Reference
172	Vourli et al 2020	Vourli, G., Noori, T., Pharris, A., Porter, K., Axelsson, M., Begovac, J., Cazein, F., Costagliola, D., Cowan, S., Croxford, S., d'Arminio Monforte, A., Delpech, V., Díaz, A., Girardi, E., Gunsenheimer-Bartmeyer, B., Hernando, V., Leierer, G., Lot, F., Nunez, O., Obel, N., … European HIV Continuum of Care Working Group (2020). Human Immunodeficiency Virus Continuum of Care in 11 European Union Countries at the End of 2016 Overall and by Key Population: Have We Made Progress?. Clinical infectious diseases : an official publication of the Infectious Diseases Society of America, 71(11), 2905–2916. https://doi.org/10.1093/cid/ciaa696
174	Marinda et al 2020	Marinda, E., Simbayi, L., Zuma, K., Zungu, N., Moyo, S., Kondlo, L., Jooste, S., Nadol, P., Igumbor, E., Dietrich, C., & Briggs-Hagen, M. (2020). Towards achieving the 90-90-90 HIV targets: results from the south African 2017 national HIV survey. BMC public health, 20(1), 1375. https://doi.org/10.1186/s12889-020-09457-z
176	Cesar et al 2016	Cesar, C., Blugerman, G., Valiente, J. A., Rebeiro, P., Sued, O., Fink, V., Soto, M. R., Cillis, R., Yamamoto, C., Falistocco, C., Cahn, P., & Pérez, H. (2016). The HIV care cascade in Buenos Aires, Argentina: results in a tertiary referral hospital. Revista panamericana de salud publica = Pan American journal of public health, 40(6), 448–454.
177	Poteat at al 2020	Poteat, T., Hanna, D. B., Rebeiro, P. F., Klein, M., Silverberg, M. J., Eron, J. J., Horberg, M. A., Kitahata, M. M., Mathews, W. C., Mattocks, K., Mayor, A., Rich, A. J., Reisner, S., Thorne, J., Moore, R. D., Jing, Y., & Althoff, K. N. (2020). Characterizing the Human Immunodeficiency Virus Care Continuum Among Transgender Women and Cisgender Women and Men in Clinical Care: A Retrospective Time-series Analysis. Clinical infectious diseases : an official publication of the Infectious Diseases Society of America, 70(6), 1131–1138. https://doi.org/10.1093/cid/ciz322
178	Bowman et al 2015	Bowman, A. S., Lerebours, L., Amesty, S., de la Rosa, M., Gil, E., Halpern, M., Nicholas, S., & Lamb, M. R. (2016). Evaluation of patient care cascade for HIV-positive patients diagnosed in La Romana, Dominican Republic in 2011: a retrospective cohort study. International journal of STD & AIDS, 27(5), 394–401. https://doi.org/10.1177/0956462415584487
179	Chkhartishvili et al 2016	Chkhartishvili, N., Chokoshvili, O., Dvali, N., Abutidze, A., Sharvadze, L., & Tsertsvadze, T. (2016). Significant Improvements Are Needed in HIV Care Continuum to Meet 90-90-90 Targets in Georgia. Journal of the International Association of Providers of AIDS Care, 15(6), 451–454. https://doi.org/10.1177/2325957416667487

Paper Number	Authors	Reference
180	Grobler et al 2017	Grobler, A., Cawood, C., Khanyile, D., Puren, A., & Kharsany, A. B. M. (2017). Progress of UNAIDS 90-90-90 targets in a district in KwaZulu-Natal, South Africa, with high HIV burden, in the HIPSS study: a household-based complex multilevel community survey. The lancet. HIV, 4(11), e505–e513. https://doi.org/10.1016/S2352-3018(17)30122-4
181	Meteliuk et al 2020	Meteliuk, A., Prokhorova, T., Filippovych, S., Ompad, D. C., & Zaller, N. (2020). The role of access to integrated services at opioid agonist treatment sites in reaching 90-90-90 cascade in people who inject drugs in Ukraine: Country-level data. Drug and alcohol dependence, 216, 108216. https://doi.org/10.1016/j.drugalcdep.2020.108216
182	Mazhnaya et al 2018	Mazhnaya, A., Marcus, R., Bojko, M. J., Zelenev, A., Makarenko, I., Pykalo, I., Filippovych, S., Dvoriak, S., & Altice, F. L. (2018). Opioid Agonist Treatment and Improved Outcomes at Each Stage of the HIV Treatment Cascade in People Who Inject Drugs in Ukraine. Journal of acquired immune deficiency syndromes (1999), 79(3), 288–295. https://doi.org/10.1097/QAI.0000000000001827
184	Chkhartishvili et al 2017	Chkhartishvili, N., Chokoshvili, O., Abutidze, A., Dvali, N., Del Rio, C., & Tsertsvadze, T. (2017). Progress Toward Achieving the UNAIDS 90-90-90 Goals in HIV Care From Diagnosis to Durable Viral Suppression in the Country of Georgia. AIDS research and human retroviruses, 33(10), 999–1003. https://doi.org/10.1089/AID.2016.0103
185	Psichogiou et al 2019	Psichogiou, M., Giallouros, G., Pantavou, K., Pavlitina, E., Papadopoulou, M., Williams, L. D., Hadjikou, A., Kakalou, E., Skoutelis, A., Protopapas, K., Antoniadou, A., Boulmetis, G., Paraskevis, D., Hatzakis, A., Friedman, S. R., & Nikolopoulos, G. K. (2019). Identifying, linking, and treating people who inject drugs and were recently infected with HIV in the context of a network-based intervention. AIDS care, 31(11), 1376–1383. https://doi.org/10.1080/09540121.2019.1601671
186	Bowman et al 2017	Bowman, A. S., Mehta, M., Lerebours Nadal, L., Halpern, M., Nicholas, S. W., & Amesty, S. (2017). Strengthening the HIV Care Continuum in the Dominican Republic: Application of a Triadic Implementation Framework to Meet the UNAIDS 90-90-90 Treatment Goal. AIDS patient care and STDs, 31(10), 407–412. https://doi.org/10.1089/apc.2017.0118
187	Ghiam et al 2017	Ghiam, M. K., Rebeiro, P. F., Turner, M., Rogers, W. B., Bebawy, S. S., Raffanti, S. P., Person, A. K., & Pettit, A. C. (2017). Trends in HIV Continuum of Care Outcomes over Ten Years of Follow-Up at a Large HIV Primary Medical Home in the Southeastern United States. AIDS research and human retroviruses, 33(10), 1027–1034. https://doi.org/10.1089/AID.2017.0016

Paper Number	Authors	Reference
188	Aboobaker et al 2022	Aboobaker, A., Zingela, Z., & Adeniyi, O. V. (2022). Correlates and cascade of HIV care in patients with psychiatric disorders in the Eastern Cape province, South Africa. The South African journal of psychiatry : SAJP : the journal of the Society of Psychiatrists of South Africa, 28, 1753. https://doi.org/10.4102/sajpsychiatry.v28i0.1753
190	Jaffer et al 2022	Jaffer, M., Christofides, N., Hlongwane, K., Otwombe, K., Milovanovic, M., Hopkins, K. L., Matuludi, M., Mbowane, V., Abdullah, F., Gray, G., Jewkes, R., & Coetzee, J. (2022). The HIV Cascade of Care and Service Utilisation at Sex Work Programmes Among Female Sex Workers in South Africa. AIDS and behavior, 26(9), 2907–2919. https://doi.org/10.1007/s10461-022-03616-6
191	Balayan et al 2019	Balayan, T., Oprea, C., Yurin, O., Jevtovic, D., Begovac, J., Lakatos, B., Sedlacek, D., Karpov, I., Horban, A., Kowalska, J. D., & Euro-guidelines in Central and Eastern Europe Network Group (2019). People who inject drugs remain hard-to-reach population across all HIV continuum stages in Central, Eastern and South Eastern Europe - data from Euro-guidelines in Central and Eastern Europe Network. Infectious diseases (London, England), 51(4), 277–286. https://doi.org/10.1080/23744235.2019.1565415
192	Colasanti et al 2016	Colasanti, J., Kelly, J., Pennisi, E., Hu, Y. J., Root, C., Hughes, D., Del Rio, C., & Armstrong, W. S. (2016). Continuous Retention and Viral Suppression Provide Further Insights Into the HIV Care Continuum Compared to the Cross-sectional HIV Care Cascade. Clinical infectious diseases : an official publication of the Infectious Diseases Society of America, 62(5), 648–654. https://doi.org/10.1093/cid/civ941
193	Raymond et al 2016	Raymond, H. F., Scheer, S., Santos, G. M., & McFarland, W. (2016). Examining progress toward the UNAIDS 90-90-90 framework among men who have sex with men, San Francisco, 2014. AIDS care, 28(9), 1177–1180. https://doi.org/10.1080/09540121.2016.1153593
194	Kelly et al 2014	Kelly, J. D., Hartman, C., Graham, J., Kallen, M. A., & Giordano, T. P. (2014). Social support as a predictor of early diagnosis, linkage, retention, and adherence to HIV care: results from the steps study. The Journal of the Association of Nurses in AIDS Care : JANAC, 25(5), 405–413. https://doi.org/10.1016/j.jana.2013.12.002
195	Hakim et al 2022	Hakim, A. J., Bolo, A., Coy, K. C., Achut, V., Katoro, J., Caesar, G., Lako, R., Taban, A. I., Sleeman, K., Wesson, J., & Okiria, A. G. (2022). Progress toward the UNAIDS 90-90-90 targets among female sex workers and sexually exploited female adolescents in Juba and Nimule, South Sudan. BMC public health, 22(1), 132. https://doi.org/10.1186/s12889-022-12533-1

Paper Number	Authors	Reference
196	Holland et al 2016	Holland, C. E., Kouanda, S., Lougué, M., Pitche, V. P., Schwartz, S., Anato, S., Ouedraogo, H. G., Tchalla, J., Yah, C. S., Kapesa, L., Ketende, S., Beyrer, C., & Baral, S. (2016). Using Population-Size Estimation and Cross-sectional Survey Methods to Evaluate HIV Service Coverage Among Key Populations in Burkina Faso and Togo. Public health reports (Washington, D.C. : 1974), 131(6), 773–782. https://doi.org/10.1177/0033354916677237
198	Krentz et al 2014	Krentz, H. B., MacDonald, J., & John Gill, M. (2014). High Mortality Among Human Immunodeficiency Virus (HIV)-Infected Individuals Before Accessing or Linking to HIV Care: A Missing Outcome in the Cascade of Care?. Open forum infectious diseases, 1(1), ofu011. https://doi.org/10.1093/ofid/ofu011
199	Lima et al 2017	Lima, V. D., St-Jean, M., Rozada, I., Shoveller, J. A., Nosyk, B., Hogg, R. S., Sereda, P., Barrios, R., & Montaner, J. S. G. (2017). Progress towards the United Nations 90-90-90 and 95-95-95 targets: the experience in British Columbia, Canada. Journal of the International AIDS Society, 20(3), e25011. https://doi.org/10.1002/jia2.25011
200	Doshi et al 2018	Doshi, R. H., Sande, E., Ogwal, M., Kiyingi, H., McIntyre, A., Kusiima, J., Musinguzi, G., Serwadda, D., & Hladik, W. (2018). Progress toward UNAIDS 90-90-90 targets: A respondent-driven survey among female sex workers in Kampala, Uganda. PloS one, 13(9), e0201352. https://doi.org/10.1371/journal.pone.0201352
201	Harooni et al 2022	Harooni, M. Z., Atarud, A. A., Ehsan, E., Alokozai, A., McFarland, W., & Mirzazadeh, A. (2022). Gaps in the continuum of care among people living with HIV in Afghanistan. International journal of STD & AIDS, 33(3), 282–288. https://doi.org/10.1177/09564624211055299
202	Harris et al 2022	Harris, T. G., Wu, Y., Parmley, L. E., Musuka, G., Mapingure, M. P., Chingombe, I., Mugurungi, O., Hakim, A., Gozhora, P., Miller, S. S., Lamb, M. R., Samba, C., & Rogers, J. H. (2022). HIV care cascade and associated factors among men who have sex with men, transgender women, and genderqueer individuals in Zimbabwe: findings from a biobehavioural survey using respondent-driven sampling. The lancet. HIV, 9(3), e182–e201. https://doi.org/10.1016/S2352-3018(21)00297-6
204	Wilson Dib et al 2020	Wilson Dib, R., Dandachi, D., Matar, M., Shayya, A., Davila, J. A., Giordano, T. P., & Mokhbat, J. E. (2020). HIV in Lebanon: Reasons for Testing, Engagement in Care, and Outcomes in Patients with Newly Diagnosed HIV Infections. AIDS and behavior, 24(8), 2290–2298. https://doi.org/10.1007/s10461-020-02788-3

Paper Number	Authors	Reference
205	Magro et al 2021	Magro, P., Cerini, C., da Gloria, A., Tembe, S., Castelli, F., & Tomasoni, L. R. (2021). The cascade of care of HIV after one year of follow-up in a cohort of HIV-positive adult patients in three health settings of Morrumbene in rural Mozambique. Tropical medicine & international health : TM & IH, 26(11), 1503–1511. https://doi.org/10.1111/tmi.13671
206	Mathews et al 2021	Mathews, C., Cheyip, M., Beauclair, R., Puren, A., Lombard, C., Jonas, K., Ayalew, K. A., Govindasamy, D., Kuo, C., Dietrich, J., Abdullah, F., & Gray, G. (2021). HIV care coverage among HIV-positive adolescent girls and young women in South Africa: Results from the HERStory Study. South African medical journal = Suid-Afrikaanse tydskrif vir geneeskunde, 111(5), 460–468. https://doi.org/10.7196/SAMJ.2021.v111i5.15351
208	Lacombe-Duncan et al 2022	Lacombe-Duncan, A., Shokoohi, M., Persad, Y., Underhill, A., Machouf, N., Côté, P., Wheatley, M., Gupta, M., Kyne, L. T., Besharati, A. A., Fung, R., Chan, L. Y. L., Arbess, G., Bourns, A., Nguyen, Q., & Loutfy, M. (2022). Short report: Characterizing HIV care among a clinical sample of transgender women living with HIV. HIV medicine, 23(4), 324–330. https://doi.org/10.1111/hiv.13261
210	Gaolathe et al 2016	Gaolathe, T., Wirth, K. E., Holme, M. P., Makhema, J., Moyo, S., Chakalisa, U., Yankinda, E. K., Lei, Q., Mmalane, M., Novitsky, V., Okui, L., van Widenfelt, E., Powis, K. M., Khan, N., Bennett, K., Bussmann, H., Dryden-Peterson, S., Lebelonyane, R., El-Halabi, S., Mills, L. A., … Botswana Combination Prevention Project study team (2016). Botswana's progress toward achieving the 2020 UNAIDS 90-90-90 antiretroviral therapy and virological suppression goals: a population-based survey. The lancet. HIV, 3(5), e221–e230. https://doi.org/10.1016/S2352-3018(16)00037-0
211	Tlhajoane et al 2018	Tlhajoane, M., Eaton, J. W., Takaruza, A., Rhead, R., Maswera, R., Schur, N., Sherr, L., Nyamukapa, C., & Gregson, S. (2018). Prevalence and Associations of Psychological Distress, HIV Infection and HIV Care Service Utilization in East Zimbabwe. AIDS and behavior, 22(5), 1485–1495. https://doi.org/10.1007/s10461-017-1705-x
212	Eberhart et al 2013	Eberhart, M. G., Yehia, B. R., Hillier, A., Voytek, C. D., Blank, M. B., Frank, I., Metzger, D. S., & Brady, K. A. (2013). Behind the cascade: analyzing spatial patterns along the HIV care continuum. Journal of acquired immune deficiency syndromes (1999), 64 Suppl 1(0 1), S42–S51. https://doi.org/10.1097/QAI.0b013e3182a90112

Paper Number	Authors	Reference
213	Maughan-Brown et al 2020	Maughan-Brown, B., Beckett, S., Kharsany, A. B. M., Cawood, C., Khanyile, D., Lewis, L., Venkataramani, A., & George, G. (2021). Poor rates of linkage to HIV care and uptake of treatment after home-based HIV testing among newly diagnosed 15-to-49 year-old men and women in a high HIV prevalence setting in South Africa. AIDS care, 33(1), 70–79. https://doi.org/10.1080/09540121.2020.1719025
214	Chihana et al 2021	Chihana, M. L., Conan, N., Ellman, T., Poulet, E., Garone, D. B., Ortuno, R., Wanjala, S., Masiku, C., Etard, J. F., Davies, M. A., & Maman, D. (2021). The HIV cascade of care among serodiscordant couples in four high HIV prevalence settings in sub-Saharan Africa. South African medical journal = Suid-Afrikaanse tydskrif vir geneeskunde, 111(8), 768–776. https://doi.org/10.7196/SAMJ.2021.v111i8.15489
215	Tsondai et al 2020	Tsondai, P. R., Sohn, A. H., Phiri, S., Sikombe, K., Sawry, S., Chimbetete, C., Fatti, G., Hobbins, M. A., Technau, K. G., Rabie, H., Bernheimer, J., Fox, M. P., Judd, A., Collins, I. J., Davies, M. A., & International epidemiology to Evaluate AIDS Southern Africa (IeDEA-SA) Collaboration (2020). Characterizing the double-sided cascade of care for adolescents living with HIV transitioning to adulthood across Southern Africa. Journal of the International AIDS Society, 23(1), e25447. https://doi.org/10.1002/jia2.25447
216	Gibbs et al 2022	Gibbs, A., Reddy, T., Closson, K., Cawood, C., Khanyile, D., & Hatcher, A. (2022). Intimate Partner Violence and the HIV Care and Treatment Cascade Among Adolescent Girls and Young Women in DREAMS, South Africa. Journal of acquired immune deficiency syndromes (1999), 89(2), 136–142. https://doi.org/10.1097/QAI.0000000000002843
217	Lambdin et al 2017	Lambdin, B. H., Kral, A. H., Comfort, M., Lopez, A. M., & Lorvick, J. (2017). Associations of criminal justice and substance use treatment involvement with HIV/HCV testing and the HIV treatment cascade among people who use drugs in Oakland, California. Addiction science & clinical practice, 12(1), 13. https://doi.org/10.1186/s13722-017-0078-9
218	Bacha et al 2022	Bacha, J. M., Dlamini, S., Anabwani, F., Gwimile, J., Kanywa, J. B., Farirai, J., Bvumbwe, M., Steffy, T., Nguyen, D., Wanless, R. S., & Haq, H. (2022). Achieving Antiretroviral Therapy Uptake and Viral Suppression Among Children and Adolescents Living With HIV in the UNAIDS 90-90-90 Era Across Six Countries in Eastern and Southern Africa-Lessons From the BIPAI Network. Journal of acquired immune deficiency syndromes (1999), 90(3), 300–308. https://doi.org/10.1097/QAI.0000000000002957

Paper Number	Authors	Reference
219	Williams et al 2018	Williams, E. C., McGinnis, K. A., Edelman, E. J., Matson, T. E., Gordon, A. J., Marshall, B. D. L., Bryant, K. J., Rubinsky, A. D., Lapham, G. T., Satre, D. D., Richards, J. E., Catz, S. L., Fiellin, D. A., Justice, A. C., & Bradley, K. A. (2019). Level of Alcohol Use Associated with HIV Care Continuum Targets in a National U.S. Sample of Persons Living with HIV Receiving Healthcare. AIDS and behavior, 23(1), 140–151. https://doi.org/10.1007/s10461-018-2210-6
220	Mulholland et al 2021	Mulholland, G. E., Markiewicz, M., Arimi, P., Ssengooba, F., Weir, S., & Edwards, J. K. (2022). HIV Prevalence and the HIV Treatment Cascade Among Female Sex Workers in Cross-Border Areas in East Africa. AIDS and behavior, 26(2), 556–568. https://doi.org/10.1007/s10461-021-03411-9
221	Kalichman et al 2020	Kalichman, S. C., Banas, E., Katner, H., Hill, M., & Kalichman, M. O. (2020). Individual Social Capital and the HIV Continuum of Care in a Rural Setting of the Southeast United States. Rural mental health, 44(2), 75–86. https://doi.org/10.1037/rmh0000134
222	Hayes et al 2017	Hayes, R., Floyd, S., Schaap, A., Shanaube, K., Bock, P., Sabapathy, K., Griffith, S., Donnell, D., Piwowar-Manning, E., El-Sadr, W., Beyers, N., Ayles, H., Fidler, S., & HPTN 071 (PopART) Study Team (2017). A universal testing and treatment intervention to improve HIV control: One-year results from intervention communities in Zambia in the HPTN 071 (PopART) cluster-randomised trial. PLoS medicine, 14(5), e1002292. https://doi.org/10.1371/journal.pmed.1002292
223	King et al 2020	King, C., Giang, L. M., Bart, G., Kunkel, L., & Korthuis, P. T. (2020). HIV care continuum characteristics among people with opioid use disorder and HIV in Vietnam: baseline results from the BRAVO study. BMC public health, 20(1), 421. https://doi.org/10.1186/s12889-020-08538-3
224	Pascom et al 2018	Pascom, A. R. P., Meireles, M. V., & Benzaken, A. S. (2018). Sociodemographic determinants of attrition in the HIV continuum of care in Brazil, in 2016. Medicine, 97(1S Suppl 1), S69–S74. https://doi.org/10.1097/MD.0000000000009857
225	Chandler et al 2019	Chandler, C. J., Sang, J. M., Bukowski, L. A., Andrade, E., Eaton, L. A., Stall, R. D., & Matthews, D. D. (2019). Characterizing the HIV care continuum among a community sample of black men who have sex with men in the United States. AIDS care, 31(7), 816–820. https://doi.org/10.1080/09540121.2018.1549724
226	Gray et al 2014	Gray, K. M., Cohen, S. M., Hu, X., Li, J., Mermin, J., & Hall, H. I. (2014). Jurisdiction level differences in HIV diagnosis, retention in care, and viral suppression in the United States. Journal of acquired immune deficiency syndromes (1999), 65(2), 129–132. https://doi.org/10.1097/QAI.0000000000000028

Paper Number	Authors	Reference
227	Gokengin et al 2018	Gokengin, D., Oprea, C., Begovac, J., Horban, A., Zeka, A. N., Sedlacek, D., Allabergan, B., Almamedova, E. A., Balayan, T., Banhegyi, D., Bukovinova, P., Chkhartishvili, N., Damira, A., Deva, E., Elenkov, I., Gashi, L., Gexha-Bunjaku, D., Hadciosmanovic, V., Harxhi, A., Holban, T., … Yurin, O. (2018). HIV care in Central and Eastern Europe: How close are we to the target?. International journal of infectious diseases : IJID : official publication of the International Society for Infectious Diseases, 70, 121–130. https://doi.org/10.1016/j.ijid.2018.03.007
228	Whitham et al 2017	Whitham, H. K., Sansom, S. L., Wejnert, C., Finlayson, T., Huang, Y. A., An, Q., Paz-Bailey, G., & NHBS Study Group (2017). Sex Practices by HIV Awareness and Engagement in the Continuum of Care Among MSM: A National HIV Behavioral Surveillance Analysis in 21 U.S. Cities. AIDS and behavior, 22(3), 840–847. https://doi.org/10.1007/s10461-017-1966-4
229	Halperin et al 2019	Halperin, J., Conner, K., Butler, I., Zeng, P., Myers, L., Clark, R., & Van Sickels, N. (2019). A Care Continuum of Immediate ART for Newly Diagnosed Patients and Patients Presenting Later to Care at a Federally Qualified Health Center in New Orleans. Open forum infectious diseases, 6(4), ofz161. https://doi.org/10.1093/ofid/ofz161
231	Okeke et al 2016	Okeke, N., McFarland, W., & Raymond, H. F. (2016). Closing the Gap? The HIV Continuum in Care for African-American Men Who Have Sex with Men, San Francisco, 2004-2014. AIDS and behavior, 21(6), 1741–1744. https://doi.org/10.1007/s10461-016-1472-0
233	Lagi et al 2018	Lagi, F., Kiros, S. T., Campolmi, I., Giachè, S., Rogasi, P. G., Mazzetti, M., Bartalesi, F., Trotta, M., Nizzoli, P., Bartoloni, A., & Sterrantino, G. (2018). Continuum of care among HIV-1 positive patients in a single center in Italy (2007-2017). Patient preference and adherence, 12, 2545–2551. https://doi.org/10.2147/PPA.S180736
234	Smith et al 2015	Smith, L. R., Patterson, T. L., Magis-Rodriguez, C., Ojeda, V. D., Burgos, J. L., Rojas, S. A., Zúñiga, M. L., & Strathdee, S. A. (2015). Engagement in the HIV Care Continuum among Key Populations in Tijuana, Mexico. AIDS and behavior, 20(5), 1017–1025. https://doi.org/10.1007/s10461-015-1186-8
235	Hightow-Weidman et al 2017	Hightow-Weidman, L., LeGrand, S., Choi, S. K., Egger, J., Hurt, C. B., & Muessig, K. E. (2017). Exploring the HIV continuum of care among young black MSM. PloS one, 12(6), e0179688. https://doi.org/10.1371/journal.pone.0179688

Paper Number	Authors	Reference
237	Gonsalves et al 2017	Gonsalves, G. S., Paltiel, A. D., Cleary, P. D., Gill, M. J., Kitahata, M. M., Rebeiro, P. F., Silverberg, M. J., Horberg, M., Abraham, A. G., Althoff, K. N., Moore, R., Bosch, R. J., Tang, T., Hall, H. I., & Kaplan, E. H. (2017). A Flow-Based Model of the HIV Care Continuum in the United States. Journal of acquired immune deficiency syndromes (1999), 75(5), 548–553. https://doi.org/10.1097/QAI.0000000000001429
238	Hellenberg et al 2013	Helleberg, M., Häggblom, A., Sönnerborg, A., & Obel, N. (2013). HIV care in the Swedish-Danish HIV cohort 1995-2010, closing the gaps. PloS one, 8(8), e72257. https://doi.org/10.1371/journal.pone.0072257
240	Zhang et al 2016	Zhang, N., Bussell, S., Wang, G., Zhu, X., Yang, X., Huang, T., Qian, Y., Tao, X., Kang, D., & Wang, N. (2016). Disparities in HIV Care Along the Path From Infection to Viral Suppression: A Cross-sectional Study of HIV/AIDS Patient Records in 2013, Shandong Province, China. Clinical infectious diseases : an official publication of the Infectious Diseases Society of America, 63(1), 115–121. https://doi.org/10.1093/cid/ciw190
242	Xia et al 2016	Xia, Q., Shah, D., Gill, B., Torian, L. V., & Braunstein, S. L. (2016). Continuum of Care Among People Living with Perinatally Acquired HIV Infection in New York City, 2014. Public health reports (Washington, D.C. : 1974), 131(4), 566–573. https://doi.org/10.1177/0033354916662215
243	Kapogiannis et al 2020	Kapogiannis, B. G., Koenig, L. J., Xu, J., Mayer, K. H., Loeb, J., Greenberg, L., Monte, D., Banks-Shields, M., Fortenberry, J. D., & Adolescent Medicine Trials Network for HIV/AIDS Interventions (2020). The HIV Continuum of Care for Adolescents and Young Adults Attending 13 Urban US HIV Care Centers of the NICHD-ATN-CDC-HRSA SMILE Collaborative. Journal of acquired immune deficiency syndromes (1999), 84(1), 92–100. https://doi.org/10.1097/QAI.0000000000002308
244	Rajabiun et al 2018	Rajabiun, S., Tryon, J., Feaster, M., Pan, A., McKeithan, L., Fortu, K., Cabral, H. J., Borne, D., & Altice, F. L. (2018). The Influence of Housing Status on the HIV Continuum of Care: Results From a Multisite Study of Patient Navigation Models to Build a Medical Home for People Living With HIV Experiencing Homelessness. American journal of public health, 108(S7), S539–S545. https://doi.org/10.2105/AJPH.2018.304736
246	Beckwith et al 2018	Beckwith, C. G., Kuo, I., Fredericksen, R. J., Brinkley-Rubinstein, L., Cunningham, W. E., Springer, S. A., Loeliger, K. B., Franks, J., Christopoulos, K., Lorvick, J., Kahana, S. Y., Young, R., Seal, D. W., Zawitz, C., Delaney, J. A., Crane, H. M., & Biggs, M. L. (2018). Risk behaviors and HIV care continuum outcomes among criminal justice-involved HIV-infected transgender women and cisgender men: Data from the Seek, Test, Treat, and Retain Harmonization Initiative. PloS one,

Paper Number	Authors	Reference
		13(5), e0197730. https://doi.org/10.1371/journal.pone.0197730
249	Xia et al 2016	Xia, Q., Lazar, R., Bernard, M. A., McNamee, P., Daskalakis, D. C., Torian, L. V., & Braunstein, S. L. (2016). New York City Achieves the UNAIDS 90-90-90 Targets for HIV-Infected Whites but Not Latinos/Hispanics and Blacks. Journal of acquired immune deficiency syndromes (1999), 73(3), e59–e62. https://doi.org/10.1097/QAI.0000000000001132
250	Skarbinski et al 2015	Skarbinski, J., Rosenberg, E., Paz-Bailey, G., Hall, H. I., Rose, C. E., Viall, A. H., Fagan, J. L., Lansky, A., & Mermin, J. H. (2015). Human immunodeficiency virus transmission at each step of the care continuum in the United States. JAMA internal medicine, 175(4), 588–596. https://doi.org/10.1001/jamainternmed.2014.8180
251	Vallecillo et al 2020	Vallecillo, G., Fonseca, F., Marín, G. et al. Reaching the 90–90–90 UNAIDS treatment target for people who inject drugs receiving integrated clinical care at a drug-use outpatient treatment facility. J Public Health (Berl.) 30, 481–486 (2022). https://doi.org/10.1007/s10389-020-01298-9
252	Tan et al 2016	Tan, J. Y., Pollack, L., Rebchook, G., Peterson, J., Huebner, D., Eke, A., Johnson, W., & Kegeles, S. (2016). The Role of the Primary Romantic Relationship in HIV Care Engagement Outcomes Among Young HIV-Positive Black Men Who Have Sex with Men. AIDS and behavior, 22(3), 774–790. https://doi.org/10.1007/s10461-016-1601-9
253	Anderson et al 2017	Anderson, E. A., Momplaisir, F. M., Corson, C., & Brady, K. A. (2017). Assessing the Impact of Perinatal HIV Case Management on Outcomes Along the HIV Care Continuum for Pregnant and Postpartum Women Living With HIV, Philadelphia 2005-2013. AIDS and behavior, 21(9), 2670–2681. https://doi.org/10.1007/s10461-017-1714-9
254	Wong et al 2017	Wong, N. S., Mao, J., Cheng, W., Tang, W., Cohen, M. S., Tucker, J. D., & Xu, H. (2018). HIV Linkage to Care and Retention in Care Rate Among MSM in Guangzhou, China. AIDS and behavior, 22(3), 701–710. https://doi.org/10.1007/s10461-017-1893-4
255	Terzian et al 2018	Terzian, A. S., Younes, N., Greenberg, A. E., Opoku, J., Hubbard, J., Happ, L. P., Kumar, P., Jones, R. R., Castel, A. D., & DC Cohort Executive Committee (2018). Identifying Spatial Variation Along the HIV Care Continuum: The Role of Distance to Care on Retention

Paper Number	Authors	Reference
		and Viral Suppression. AIDS and behavior, 22(9), 3009–3023. https://doi.org/10.1007/s10461-018-2103-8
256	Takada et al 2019	Takada, S., Ettner, S. L., Harawa, N. T., Garland, W. H., Shoptaw, S. J., & Cunningham, W. E. (2019). Life Chaos is Associated with Reduced HIV Testing, Engagement in Care, and ART Adherence Among Cisgender Men and Transgender Women upon Entry into Jail. AIDS and behavior, 24(2), 491–505. https://doi.org/10.1007/s10461-019-02570-0
257	Mimiaga et al 2019	Mimiaga, M. J., August Oddleifson, D., Meersman, S. C., Silvia, A., Hughto, J. M. W., Landers, S., Brown, E., & Loberti, P. (2019). Multilevel Barriers to Engagement in the HIV Care Continuum Among Residents of the State of Rhode Island Living with HIV. AIDS and behavior, 24(4), 1133–1150. https://doi.org/10.1007/s10461-019-02677-4
258	Abia et al 2020	Abia, A., Rothman, R. E., Mohareb, A. M., Lim, G. L. H., Patel, A. V., Bigelow, B., Klein, E. Y., Cole, G., Gebo, K. A., Moore, R. D., & Hsieh, Y. H. (2020). Resource utilization across the continuum of HIV care: An emergency department-based cohort study. The American journal of emergency medicine, 43, 164–169. https://doi.org/10.1016/j.ajem.2020.02.037
259	Hsieh et al 2015	Hsieh, Y. H., Kelen, G. D., Laeyendecker, O., Kraus, C. K., Quinn, T. C., & Rothman, R. E. (2015). HIV Care Continuum for HIV-Infected Emergency Department Patients in an Inner-City Academic Emergency Department. Annals of emergency medicine, 66(1), 69–78. https://doi.org/10.1016/j.annemergmed.2015.01.001
260	Jin et al 2018	Jin, H., Ogunbajo, A., Mimiaga, M. J., Duncan, D. T., Boyer, E., Chai, P., Dilworth, S. E., & Carrico, A. W. (2018). Over the influence: The HIV care continuum among methamphetamine-using men who have sex with men. Drug and alcohol dependence, 192, 125–128. https://doi.org/10.1016/j.drugalcdep.2018.07.038
261	Satre et al 2021	Satre, D. D., Levine-Hall, T., Sterling, S. A., Young-Wolff, K. C., Lam, J. O., Alexeeff, S., Hojilla, J. C., Williams, A., Justice, A. C., Sterne, J., Cavassini, M., Bryant, K. J., Williams, E. C., Horberg, M. A., Volberding, P., Weisner, C., & Silverberg, M. J. (2021). The relationship of smoking and unhealthy alcohol use to the HIV care continuum among people with HIV in an integrated health care system. Drug and alcohol dependence, 219, 108481. https://doi.org/10.1016/j.drugalcdep.2020.108481

Paper Number	Authors	Reference
262	Nwangu-ike et al 2015	Nwangwu-Ike, N., Hernandez, A. L., An, Q., Huang, T., & Hall, H. I. (2015). The Epidemiology of Human Immunodeficiency Virus Infection and Care among Adult and Adolescent Females in the United States, 2008-2012. Women's health issues : official publication of the Jacobs Institute of Women's Health, 25(6), 711–719. https://doi.org/10.1016/j.whi.2015.07.004
264	Parchure et al 2015	Parchure, R., Kulkarni, V., Kulkarni, S., & Gangakhedkar, R. (2015). Pattern of linkage and retention in HIV care continuum among patients attending referral HIV care clinic in private sector in India. AIDS care, 27(6), 716–722. https://doi.org/10.1080/09540121.2014.996518
265	Yoon et al 2017	Yoon, I. S., Downing, M. J., Jr, Teran, R., Chiasson, M. A., Houang, S. T., Parsons, J. T., & Hirshfield, S. (2017). Sexual risk taking and the HIV care continuum in an online sample of men who have sex with men. AIDS care, 30(7), 921–929. https://doi.org/10.1080/09540121.2017.1417535
266	Takada et al 2020	Takada, S., Gorbach, P., Brookmeyer, R., & Shoptaw, S. (2021). Associations of social capital resources and experiences of homophobia with HIV transmission risk behavior and HIV care continuum among men who have sex with men in Los Angeles. AIDS care, 33(5), 663–674. https://doi.org/10.1080/09540121.2020.1828798
267	Morgan et al 2016	Morgan, E., Khanna, A. S., Skaathun, B., Michaels, S., Young, L., Duvoisin, R., Chang, M., Voisin, D., Cornwell, B., Coombs, R. W., Friedman, S. R., & Schneider, J. A. (2016). Marijuana Use Among Young Black Men Who Have Sex With Men and the HIV Care Continuum: Findings From the uConnect Cohort. Substance use & misuse, 51(13), 1751–1759. https://doi.org/10.1080/10826084.2016.1197265
268	Rowan et al 2014	Rowan, S. E., Burman, W. J., Johnson, S. C., Connick, E., Reirden, D., Daniloff, E., & Gardner, E. M. (2014). Engagement-in-care during the first 5 years after HIV diagnosis: data from a cohort of newly HIV-diagnosed individuals in a large US city. AIDS patient care and STDs, 28(9), 475–482. https://doi.org/10.1089/apc.2013.0340
269	Almirol et al 2018	Almirol, E. A., McNulty, M. C., Schmitt, J., Eavou, R., Taylor, M., Tobin, A., Ramirez, K., Glick, N., Stamos, M., Schuette, S., Ridgway, J. P., & Pitrak, D. (2018). Gender Differences in HIV Testing, Diagnosis, and Linkage to Care in Healthcare Settings: Identifying African American Women with HIV in Chicago. AIDS patient care and STDs, 32(10), 399–407. https://doi.org/10.1089/apc.2018.0066
270	Parcesepe et al 2020	Parcesepe, A. M., Lahuerta, M., Lamb, M. R., Ahoua, L., Abacassamo, F., & Elul, B. (2020). Household Decision-Making and HIV Care Continuum Outcomes Among Women Living with HIV in Mozambique. AIDS patient

Paper Number	Authors	Reference
		care and STDs, 34(4), 173–183. https://doi.org/10.1089/apc.2019.0268
271	Desir et al 2018	Desir, F. A., Lesko, C. R., Moore, R. D., Horberg, M. A., Wong, C., Crane, H. M., Silverberg, M., Thorne, J. E., Rachlis, B., Rabkin, C., Mayor, A. M., Mathews, W. C., Althoff, K. N., & North American AIDS Cohort Collaboration on Research and Design (NA-ACCORD) Region of the International Epidemiologic Databases to Evaluate AIDS (IeDEA) Consortium (2018). One Size Fits (n)One: The Influence of Sex, Age, and Sexual Human Immunodeficiency Virus (HIV) Acquisition Risk on Racial/Ethnic Disparities in the HIV Care Continuum in the United States. Clinical infectious diseases : an official publication of the Infectious Diseases Society of America, 68(5), 795–802. https://doi.org/10.1093/cid/ciy556
272	Cuzin et al 2021	Cuzin, L., Allavena, C., Cotte, L., Delpierre, C., Huleux, T., Palich, R., Delobel, P., Raffi, F., Cabié, A., & Dat'AIDS Study Group (2021). No barrier to care, yet disparities in the HIV care continuum in France: a nationwide population study. The Journal of antimicrobial chemotherapy, 76(6), 1573–1579. https://doi.org/10.1093/jac/dkab061
273	Lesko et al 2016	Lesko, C. R., Edwards, J. K., Moore, R. D., & Lau, B. (2016). A longitudinal, HIV care continuum: 10-year restricted mean time in each care continuum stage after enrollment in care, by history of IDU. AIDS (London, England), 30(14), 2227–2234. https://doi.org/10.1097/QAD.0000000000001183
274	Schneider et al 2017	Schneider, J. A., Kozloski, M., Michaels, S., Skaathun, B., Voisin, D., Lancki, N., Morgan, E., Khanna, A., Green, K., Coombs, R. W., Friedman, S. R., Laumann, E., Schumm, P., & uConnectand BARS study teams (2017). Criminal justice involvement history is associated with better HIV care continuum metrics among a population-based sample of young black MSM. AIDS (London, England), 31(1), 159–165. https://doi.org/10.1097/QAD.0000000000001269
275	Matson et al 2018	Matson, T. E., McGinnis, K. A., Rubinsky, A. D., Frost, M. C., Czarnogorski, M., Bryant, K. J., Edelman, E. J., Satre, D. D., Catz, S. L., Bensley, K. M., Fiellin, D. A., Justice, A. C., & Williams, E. C. (2018). Gender and alcohol use: influences on HIV care continuum in a national cohort of patients with HIV. AIDS (London, England), 32(15), 2247–2253. https://doi.org/10.1097/QAD.0000000000001946

Paper Number	Authors	Reference
276	Chen et al 2019	Chen, J. S., Pence, B. W., Rahangdale, L., Patterson, K. B., Farel, C. E., Durr, A. L., Antono, A. C., Zakharova, O., Eron, J. J., & Napravnik, S. (2019). Postpartum HIV care continuum outcomes in the southeastern USA. AIDS (London, England), 33(4), 637–644. https://doi.org/10.1097/QAD.0000000000002094
277	Nelson et al 2016	Nelson, J. A., Kinder, A., Johnson, A. S., Hall, H. I., Hu, X., Sweet, D., Guido, A., Katner, H., Janelle, J., Gonzalez, M., Paz, N. M., Ledonne, C., Henry, J., Bramel, T., & Harris, J. (2018). Differences in Selected HIV Care Continuum Outcomes Among People Residing in Rural, Urban, and Metropolitan Areas-28 US Jurisdictions. The Journal of rural health : official journal of the American Rural Health Association and the National Rural Health Care Association, 34(1), 63–70. https://doi.org/10.1111/jrh.12208
278	Wirtz et al 2015	Wirtz, A. L., Zelaya, C. E., Latkin, C., Peryshkina, A., Galai, N., Mogilniy, V., Dzhigun, P., Kostetskaya, I., Mehta, S. H., & Beyrer, C. (2015). The HIV care continuum among men who have sex with men in Moscow, Russia: a cross-sectional study of infection awareness and engagement in care. Sexually transmitted infections, 92(2), 161–167. https://doi.org/10.1136/sextrans-2015-052076
279	Mitsch et al 2016	Mitsch, A., Surendera Babu, A., Seneca, D., Whiteside, Y. O., & Warne, D. (2016). HIV care and treatment of American Indians/Alaska natives with diagnosed HIV infection - 27 states and the District of Columbia, 2012. International journal of STD & AIDS, 28(10), 953–961. https://doi.org/10.1177/0956462416681183
280	Van Beckhoven et al 2015	Van Beckhoven, D., Florence, E., Ruelle, J., Deblonde, J., Verhofstede, C., Callens, S., Vancutsem, E., Lacor, P., Demeester, R., Goffard, J. C., Sasse, A., & BREACH Belgian Research on AIDS and HIV Consortium (2015). Good continuum of HIV care in Belgium despite weaknesses in retention and linkage to care among migrants. BMC infectious diseases, 15, 496. https://doi.org/10.1186/s12879-015-1230-3
282	Ntombela et al 2022	Ntombela, N. P., Kharsany, A. B. M., Soogun, A., Yende-Zuma, N., Baxter, C., Kohler, H. P., & McKinnon, L. R. (2022). Viral suppression among pregnant adolescents and women living with HIV in rural KwaZulu-Natal, South Africa: a cross sectional study to assess progress towards UNAIDS indicators and Implications for HIV Epidemic Control. Reproductive health, 19(1), 116. https://doi.org/10.1186/s12978-022-01419-5
283	Lindman et al 2020	Lindman, J., Djalo, M. A., Biai, A., Månsson, F., Esbjörnsson, J., Jansson, M., Medstrand, P., Norrgren, H., & SWEGUB CORE group (2020). The HIV care continuum and HIV-1 drug resistance among female sex workers: a key population in Guinea-Bissau. AIDS

Paper Number	Authors	Reference
		research and therapy, 17(1), 33. https://doi.org/10.1186/s12981-020-00290-3
284	Mcnairy et al 2017	McNairy, M. L., Lamb, M. R., Gachuhi, A. B., Nuwagaba-Biribonwoha, H., Burke, S., Mazibuko, S., Okello, V., Ehrenkranz, P., Sahabo, R., & El-Sadr, W. M. (2017). Effectiveness of a combination strategy for linkage and retention in adult HIV care in Swaziland: The Link4Health cluster randomized trial. PLoS medicine, 14(11), e1002420. https://doi.org/10.1371/journal.pmed.1002420
285	Shade et al 2021	Shade, S. B., Kirby, V. B., Stephens, S., Moran, L., Charlebois, E. D., Xavier, J., Cajina, A., Steward, W. T., & Myers, J. J. (2021). Outcomes and costs of publicly funded patient navigation interventions to enhance HIV care continuum outcomes in the United States: A before-and-after study. PLoS medicine, 18(5), e1003418. https://doi.org/10.1371/journal.pmed.1003418
286	Dailey et al 2017	Dailey, A. F., Johnson, A. S., & Wu, B. (2017). HIV Care Outcomes Among Blacks with Diagnosed HIV - United States, 2014. MMWR. Morbidity and mortality weekly report, 66(4), 97–103. https://doi.org/10.15585/mmwr.mm6604a2
287	Harxhi et al 2020	Harxhi, A., Vrapi, E., Gjataj, A., Meta, E., Simaku, A., Bani, R., ... & Youle, M. HIV care cascade in Albania: analysis of newly diagnosed cases in 2016. HIV & AIDS Review. International Journal of HIV-Related Problems, 19(4), 267-272.
288	Alvarezi - Uria et al 2013	Alvarez-Uria, G., Pakam, R., Midde, M., & Naik, P. K. (2013). Entry, Retention, and Virological Suppression in an HIV Cohort Study in India: Description of the Cascade of Care and Implications for Reducing HIV-Related Mortality in Low- and Middle-Income Countries. Interdisciplinary perspectives on infectious diseases, 2013, 384805. https://doi.org/10.1155/2013/384805
289	Gant et al 2014	Gant, Z., Bradley, H., Hu, X., Skarbinski, J., Hall, H. I., Lansky, A., & Centers for Disease Control and Prevention (CDC) (2014). Hispanics or Latinos living with diagnosed HIV: progress along the continuum of HIV care - United States, 2010. MMWR. Morbidity and mortality weekly report, 63(40), 886–890.
290	Shi et al 2021	Shi, L., Tang, W., Hu, H., Qiu, T., Marley, G., Liu, X., Chen, Y., Chen, Y., & Fu, G. (2021). The impact of COVID-19 pandemic on HIV care continuum in Jiangsu, China. BMC infectious diseases, 21(1), 768. https://doi.org/10.1186/s12879-021-06490-0

Paper Number	Authors	Reference
292	Greene et al 2018	Greene, R. E., Luong, A., Barton, S. C., Kapadia, F., & Halkitis, P. N. (2018). Assessing Gaps in the HIV Care Continuum in Young Men Who Have Sex With Men: The P18 Cohort Study. The Journal of the Association of Nurses in AIDS Care : JANAC, 29(3), 475–478. https://doi.org/10.1016/j.jana.2017.12.004
295	Kilcrease et al 2019	Kilcrease, C., Miller, M. M., Neely, S., & Liedtke, M. D. (2020). Pharmacist impact on the HIV Care Continuum: Decreasing time to care. Journal of the American College of Clinical Pharmacy, 3(3), 586-592.
296	Ndori-mharadze et al 2018	Ndori-Mharadze, T., Fearon, E., Busza, J., Dirawo, J., Musemburi, S., Davey, C., Acharya, X., Mtetwa, S., Hargreaves, J. R., & Cowan, F. (2018). Changes in engagement in HIV prevention and care services among female sex workers during intensified community mobilization in 3 sites in Zimbabwe, 2011 to 2015. Journal of the International AIDS Society, 21 Suppl 5(Suppl Suppl 5), e25138. https://doi.org/10.1002/jia2.25138
297	Baguso et al 2019	Baguso, G. N., Turner, C. M., Santos, G. M., Raymond, H. F., Dawson-Rose, C., Lin, J., & Wilson, E. C. (2019). Successes and final challenges along the HIV care continuum with transwomen in San Francisco. Journal of the International AIDS Society, 22(4), e25270. https://doi.org/10.1002/jia2.25270
298	Bhattacharjee et al 2020	Bhattacharjee, P., Isac, S., Musyoki, H., Emmanuel, F., Olango, K., Kuria, S., Ongaro, M. K., Walimbwa, J., Musimbi, J., Mugambi, M., Kaosa, S., Kioko, J., Njraini, M., Melon, M., Onyoni, J., Bartilol, K., Becker, M., Lorway, R., Pickles, M., Moses, S., … Mishra, S. (2020). HIV prevalence, testing and treatment among men who have sex with men through engagement in virtual sexual networks in Kenya: a cross-sectional bio-behavioural study. Journal of the International AIDS Society, 23 Suppl 2(Suppl 2), e25516. https://doi.org/10.1002/jia2.25516
299	Ramadhani et al 2020	Ramadhani, H. O., Crowell, T. A., Nowak, R. G., Ndembi, N., Kayode, B. O., Kokogho, A., Ononaku, U., Shoyemi, E., Ekeh, C., Adebajo, S., Baral, S. D., & Charurat, M. E. (2020). Association of age with healthcare needs and engagement among Nigerian men who have sex with men and transgender women: cross-sectional and longitudinal analyses from an observational cohort. Journal of the International AIDS Society, 23 Suppl 6(Suppl 6), e25599. https://doi.org/10.1002/jia2.25599
300	Yohannes et al 2021	Yohannes, N. T., Jenkins, C. A., Clouse, K., Cortés, C. P., Mejía Cordero, F., Padgett, D., Rouzier, V., Friedman, R. K., McGowan, C. C., Shepherd, B. E., & Rebeiro, P. F. (2021). Timing of HIV diagnosis relative to pregnancy and postpartum HIV care continuum outcomes among Latin American women, 2000 to 2017. Journal of the International AIDS Society, 24(5), e25740. https://doi.org/10.1002/jia2.25740

Paper Number	Authors	Reference
301	Lesko et al 2016	Lesko, C. R., Tong, W., Moore, R. D., & Lau, B. (2016). Retention, Antiretroviral Therapy Use and Viral Suppression by History of Injection Drug Use Among HIV-Infected Patients in an Urban HIV Clinical Cohort. AIDS and behavior, 21(4), 1016–1024. https://doi.org/10.1007/s10461-016-1585-5
302	Zhang et al 2017	Zhang, J., Xu, J. J., Song, W., Pan, S., Chu, Z. X., Hu, Q. H., Yu, H., Mao, X., Jiang, Y. J., Geng, W. Q., Shang, H., & Wang, N. (2018). HIV Incidence and Care Linkage among MSM First-Time-Testers in Shenyang, China 2012-2014. AIDS and behavior, 22(3), 711–721. https://doi.org/10.1007/s10461-017-1840-4
304	Norwood et al 2022	Norwood, J., Kheshti, A., Shepherd, B. E., Rebeiro, P. F., Ahonkhai, A., Kelly, S., & Wanjalla, C. (2022). The Impact of COVID-19 on the HIV Care Continuum in a Large Urban Southern Clinic. AIDS and behavior, 26(8), 2825–2829. https://doi.org/10.1007/s10461-022-03615-7
305	Brewer et al 2020	Brewer, R., Hood, K. B., Hotton, A., Moore, M., Spieldenner, A., Daunis, C., Mukherjee, S., Sprague, L., Schneider, J. A., Smith-Davis, M., Brown, G., & Bowen, B. (2022). Associations Between Experienced HIV Stigma, Resulting Consequences, and the HIV Care Continuum: Moderating Effects of Two Resilience Characteristics Among Persons Living with HIV (PLWH) in Louisiana. Journal of racial and ethnic health disparities, 9(1), 9–22. https://doi.org/10.1007/s40615-020-00925-1
306	Genberg et al 2015	Genberg, B. L., Naanyu, V., Wachira, J., Hogan, J. W., Sang, E., Nyambura, M., Odawa, M., Duefield, C., Ndege, S., & Braitstein, P. (2015). Linkage to and engagement in HIV care in western Kenya: an observational study using population-based estimates from home-based counselling and testing. The lancet. HIV, 2(1), e20–e26. https://doi.org/10.1016/S2352-3018(14)00034-4
307	Jose et al 2018	Jose, S., Delpech, V., Howarth, A., Burns, F., Hill, T., Porter, K., Sabin, C. A., & UK CHIC Study Steering Committee (2018). A continuum of HIV care describing mortality and loss to follow-up: a longitudinal cohort study. The lancet. HIV, 5(6), e301–e308. https://doi.org/10.1016/S2352-3018(18)30048-1
308	Steiner et al 2020	Steiner, C., MacKellar, D., Cham, H. J., Rwabiyago, O. E., Maruyama, H., Msumi, O., Pals, S., Weber, R., Kundi, G., Byrd, J., Kazaura, K., Madevu-Matson, C., Morales, F., Justman, J., Rutachunzibwa, T., & Rwebembera, A. (2020). Community-wide HIV testing, linkage case management, and defaulter tracing in Bukoba, Tanzania: pre-intervention and post-intervention, population-based survey evaluation. The lancet. HIV, 7(10), e699–e710. https://doi.org/10.1016/S2352-3018(20)30199-5

Paper Number	Authors	Reference
310	Kouyoumdjian et al 2019	Kouyoumdjian, F. G., Lamarche, L., McCormack, D., Rowe, J., Kiefer, L., Kroch, A., & Antoniou, T. (2020). 90-90-90 for everyone?: Access to HIV care and treatment for people with HIV who experience imprisonment in Ontario, Canada. AIDS care, 32(9), 1168–1176. https://doi.org/10.1080/09540121.2019.1679710
312	Ross et al 2017	Ross, J., Felsen, U. R., Cunningham, C. O., Patel, V. V., & Hanna, D. B. (2017). Outcomes Along the HIV Care Continuum Among Undocumented Immigrants in Clinical Care. AIDS research and human retroviruses, 33(10), 1038–1044. https://doi.org/10.1089/AID.2017.0015
313	Tymejczyk et al 2018	Tymejczyk, O., Jamison, K., Pathela, P., Braunstein, S., Schillinger, J. A., & Nash, D. (2018). HIV Care and Viral Load Suppression After Sexual Health Clinic Visits by Out-of-Care HIV-Positive Persons. AIDS patient care and STDs, 32(10), 390–398. https://doi.org/10.1089/apc.2018.0097
314	Raj et al 2018	Raj, A., Yore, J., Urada, L., Triplett, D. P., Vaida, F., & Smith, L. R. (2018). Multi-Site Evaluation of Community-Based Efforts to Improve Engagement in HIV Care Among Populations Disproportionately Affected by HIV in the United States. AIDS patient care and STDs, 32(11), 438–449. https://doi.org/10.1089/apc.2018.0128
315	Doshi et al 2014	Doshi, R. K., Milberg, J., Isenberg, D., Matthews, T., Malitz, F., Matosky, M., Trent-Adams, S., Parham Hopson, D., & Cheever, L. W. (2014). High rates of retention and viral suppression in the US HIV safety net system: HIV care continuum in the Ryan White HIV/AIDS Program, 2011. Clinical infectious diseases : an official publication of the Infectious Diseases Society of America, 60(1), 117–125. https://doi.org/10.1093/cid/ciu722
316	Mehta et al 2015	Mehta, S. H., Lucas, G. M., Solomon, S., Srikrishnan, A. K., McFall, A. M., Dhingra, N., Nandagopal, P., Kumar, M. S., Celentano, D. D., & Solomon, S. S. (2015). HIV care continuum among men who have sex with men and persons who inject drugs in India: barriers to successful engagement. Clinical infectious diseases : an official publication of the Infectious Diseases Society of America, 61(11), 1732–1741. https://doi.org/10.1093/cid/civ669
317	Adams et al 2015	Adams, J. W., Brady, K. A., Michael, Y. L., Yehia, B. R., & Momplaisir, F. M. (2015). Postpartum Engagement in HIV Care: An Important Predictor of Long-term Retention in Care and Viral Suppression. Clinical infectious diseases : an official publication of the Infectious Diseases Society of America, 61(12), 1880–1887. https://doi.org/10.1093/cid/civ678

Paper Number	Authors	Reference
318	Philbin et al 2019	Philbin, M. M., Feaster, D. J., Gooden, L., Duan, R., Das, M., Jacobs, P., Lucas, G. M., Batey, D. S., Nijhawan, A., Jacobson, J. M., Mandler, R., Daar, E., McMahon, D. K., Armstrong, W. S., Del Rio, C., & Metsch, L. R. (2019). The North-South Divide: Substance Use Risk, Care Engagement, and Viral Suppression Among Hospitalized Human Immunodeficiency Virus-Infected Patients in 11 US Cities. Clinical infectious diseases : an official publication of the Infectious Diseases Society of America, 68(1), 146–149. https://doi.org/10.1093/cid/ciy506
319	Summers et al 2021	Summers, N. A., Huynh, T. T., Dunn, R. C., Cross, S. L., & Fuchs, C. J. (2021). Effects of Gender-Affirming Hormone Therapy on Progression Along the HIV Care Continuum in Transgender Women. Open forum infectious diseases, 8(9), ofab404. https://doi.org/10.1093/ofid/ofab404
320	Schechter et al 2018	Schechter, M. C., Bizune, D., Kagei, M., Holland, D. P., Del Rio, C., Yamin, A., Mohamed, O., Oladele, A., Wang, Y. F., Rebolledo, P. A., Ray, S. M., & Kempker, R. R. (2018). Challenges Across the HIV Care Continuum for Patients With HIV/TB Co-infection in Atlanta, GA [corrected]. Open forum infectious diseases, 5(4), ofy063. https://doi.org/10.1093/ofid/ofy063
321	Haachambwa et al 2019	Haachambwa, L., Kandiwo, N., Zulu, P. M., Rutagwera, D., Geng, E., Holmes, C. B., Sinkala, E., Claassen, C. W., Mugavero, M. J., Wa Mwanza, M., Turan, J. M., & Vinikoor, M. J. (2019). Care Continuum and Postdischarge Outcomes Among HIV-Infected Adults Admitted to the Hospital in Zambia. Open forum infectious diseases, 6(10), ofz336. https://doi.org/10.1093/ofid/ofz336
322	Luo et al 2019	Luo, Y., Wu, H., Zhang, X. L., Li, X. T., Scott, S. R., Chen, J. F., & Wu, Z. Y. (2019). HIV care continuum among newly diagnosed student and non-student youths between 2012 and 2016 in Hangzhou, China. Chinese medical journal, 132(12), 1420–1428. https://doi.org/10.1097/CM9.0000000000000264
323	Katz et al 2019	Katz, I. T., Bogart, L. M., Dietrich, J. J., Leslie, H. H., Iyer, H. S., Leone, D., Magidson, J. F., Earnshaw, V. A., Courtney, I., Tshabalala, G., Fitzmaurice, G. M., Orrell, C., Gray, G., & Bangsberg, D. R. (2019). Understanding the role of resilience resources, antiretroviral therapy initiation, and HIV-1 RNA suppression among people living with HIV in South Africa: a prospective cohort study. AIDS (London, England), 33 Suppl 1(Suppl 1), S71–S79. https://doi.org/10.1097/QAD.0000000000002175
324	Dombrowski et al 2012	Dombrowski, J. C., Kent, J. B., Buskin, S. E., Stekler, J. D., & Golden, M. R. (2012). Population-based metrics for the timing of HIV diagnosis, engagement in HIV care, and virologic suppression. AIDS (London, England), 26(1), 77–86. https://doi.org/10.1097/QAD.0b013e32834dcee9

Paper Number	Authors	Reference
325	Dombrowski et al 2014	Dombrowski, J. C., Buskin, S. E., Bennett, A., Thiede, H., & Golden, M. R. (2014). Use of multiple data sources and individual case investigation to refine surveillance-based estimates of the HIV care continuum. Journal of acquired immune deficiency syndromes (1999), 67(3), 323–330. https://doi.org/10.1097/QAI.0000000000000302
326	Wiewel et al 2015	Wiewel, E. W., Braunstein, S. L., Xia, Q., Shepard, C. W., & Torian, L. V. (2015). Monitoring outcomes for newly diagnosed and prevalent HIV cases using a care continuum created with New York city surveillance data. Journal of acquired immune deficiency syndromes (1999), 68(2), 217–226. https://doi.org/10.1097/QAI.0000000000000424
327	Zulliger et al 2015	Zulliger, R., Barrington, C., Donastorg, Y., Perez, M., & Kerrigan, D. (2015). High Drop-off Along the HIV Care Continuum and ART Interruption Among Female Sex Workers in the Dominican Republic. Journal of acquired immune deficiency syndromes (1999), 69(2), 216–222. https://doi.org/10.1097/QAI.0000000000000590
328	Backus et al 2015	Backus, L., Czarnogorski, M., Yip, G., Thomas, B. P., Torres, M., Bell, T., & Ross, D. (2015). HIV Care Continuum Applied to the US Department of Veterans Affairs: HIV Virologic Outcomes in an Integrated Health Care System. Journal of acquired immune deficiency syndromes (1999), 69(4), 474–480. https://doi.org/10.1097/QAI.0000000000000615
330	Patrick et al 2017	Patrick, R., Greenberg, A., Magnus, M., Opoku, J., Kharfen, M., & Kuo, I. (2017). Development of an HIV Testing Dashboard to Complement the HIV Care Continuum Among MSM, PWID, and Heterosexuals in Washington, DC, 2007-2015. Journal of acquired immune deficiency syndromes (1999), 75 Suppl 3(Suppl 3), S397–S407. https://doi.org/10.1097/QAI.0000000000001417
331	Friedman et al 2018	Friedman, M. R., Sang, J. M., Bukowski, L. A., Matthews, D. D., Eaton, L. A., Raymond, H. F., & Stall, R. (2018). HIV Care Continuum Disparities Among Black Bisexual Men and the Mediating Effect of Psychosocial Comorbidities. Journal of acquired immune deficiency syndromes (1999), 77(5), 451–458. https://doi.org/10.1097/QAI.0000000000001631
332	Pathela et al 2018	Pathela, P., Jamison, K., Braunstein, S. L., Schillinger, J. A., Tymejczyk, O., & Nash, D. (2018). Gaps Along the HIV Care Continuum: Findings Among a Population Seeking Sexual Health Care Services in New York City. Journal of acquired immune deficiency syndromes (1999), 78(3), 314–321. https://doi.org/10.1097/QAI.0000000000001674

Paper Number	Authors	Reference
333	Ramadhani et al 2018	Ramadhani, H. O., Ndembi, N., Nowak, R. G., Ononaku, U., Gwamna, J., Orazulike, I., Adebajo, S., Crowell, T. A., Liu, H., Baral, S. D., Ake, J., Charurat, M. E., & TRUST/RV368 Study Group (2018). Individual and Network Factors Associated With HIV Care Continuum Outcomes Among Nigerian MSM Accessing Health Care Services. Journal of acquired immune deficiency syndromes (1999), 79(1), e7–e16. https://doi.org/10.1097/QAI.0000000000001756
334	Bukowski et al 2018	Bukowski, L. A., Chandler, C. J., Creasy, S. L., Matthews, D. D., Friedman, M. R., & Stall, R. D. (2018). Characterizing the HIV Care Continuum and Identifying Barriers and Facilitators to HIV Diagnosis and Viral Suppression Among Black Transgender Women in the United States. Journal of acquired immune deficiency syndromes (1999), 79(4), 413–420. https://doi.org/10.1097/QAI.0000000000001831
335	Hoover et al 2019	Hoover, K. W., Hu, X., Porter, S. E., Buchacz, K., Bond, M. D., Siddiqi, A. E., & Haynes, S. G. (2019). HIV Diagnoses and the HIV Care Continuum Among Women and Girls Aged ≥13 Years-39 States and the District of Columbia, 2015-2016. Journal of acquired immune deficiency syndromes (1999), 81(3), 251–256. https://doi.org/10.1097/QAI.0000000000002023
336	Kerrigan et al 2019	Kerrigan, D., Mbwambo, J., Likindikoki, S., Davis, W., Mantsios, A., Beckham, S. W., Leddy, A., Shembilu, C., Mwampashi, A., Aboud, S., & Galai, N. (2019). Project Shikamana: Community Empowerment-Based Combination HIV Prevention Significantly Impacts HIV Incidence and Care Continuum Outcomes Among Female Sex Workers in Iringa, Tanzania. Journal of acquired immune deficiency syndromes (1999), 82(2), 141–148. https://doi.org/10.1097/QAI.0000000000002123
337	Hakim et al 2021	Hakim, A. J., Tippett Barr, B. A., Kinchen, S., Musuka, G., Manjengwa, J., Munyati, S., Gwanzura, L., Mugurungi, O., Ncube, G., Saito, S., Parekh, B. S., Patel, H., Duong, Y. T., Gonese, E., Sleeman, K., Ruangtragool, L., Justman, J., Herman-Roloff, A., & Radin, E. (2021). Progress Toward the 90-90-90 HIV Targets in Zimbabwe and Identifying Those Left Behind. Journal of acquired immune deficiency syndromes (1999), 88(3), 272–281. https://doi.org/10.1097/QAI.0000000000002772
338	Dailey et al 2021	Dailey, A., Johnson, A. S., Hu, X., Gant, Z., Lyons, S. J., & Adih, W. (2021). Trends in HIV Care Outcomes Among Adults and Adolescents-33 Jurisdictions, United States, 2014-2018. Journal of acquired immune deficiency syndromes (1999), 88(4), 333–339. https://doi.org/10.1097/QAI.0000000000002778

Paper Number	Authors	Reference
339	Hakim et al 2019	Hakim, A. J., Badman, S. G., Weikum, D., Amos, A., Willie, B., Narokobi, R., Gabuzzi, J., Pekon, S., Kupul, M., Hou, P., Aeno, H., Neo Boli, R., Nembari, J., Ase, S., Kaldor, J. M., Vallely, A. J., Kelly-Hanku, A., & Kauntim mi tu Study Team (2019). Considerable distance to reach 90-90-90 targets among female sex workers, men who have sex with men and transgender women in Port Moresby, Papua New Guinea: findings from a cross-sectional respondent-driven sampling survey. Sexually transmitted infections, 96(2), 143–150. https://doi.org/10.1136/sextrans-2019-053961
340	Eamsakulrat et al 2022	Eamsakulrat, P., & Kiertiburanakul, S. (2022). The Impact of Timing of Antiretroviral Therapy Initiation on Retention in Care, Viral Load Suppression and Mortality in People Living with HIV: A Study in a University Hospital in Thailand. Journal of the International Association of Providers of AIDS Care, 21, 23259582221082607. https://doi.org/10.1177/23259582221082607
341	Cope et al 2015	Cope, A. B., Powers, K. A., Kuruc, J. D., Leone, P. A., Anderson, J. A., Ping, L. H., Kincer, L. P., Swanstrom, R., Mobley, V. L., Foust, E., Gay, C. L., Eron, J. J., Cohen, M. S., & Miller, W. C. (2015). Ongoing HIV Transmission and the HIV Care Continuum in North Carolina. PloS one, 10(6), e0127950. https://doi.org/10.1371/journal.pone.0127950
342	Lancaster et al 2016	Lancaster, K. E., Powers, K. A., Lungu, T., Mmodzi, P., Hosseinipour, M. C., Chadwick, K., Go, V. F., Pence, B. W., Hoffman, I. F., & Miller, W. C. (2016). The HIV Care Continuum among Female Sex Workers: A Key Population in Lilongwe, Malawi. PloS one, 11(1), e0147662. https://doi.org/10.1371/journal.pone.0147662
343	Poon et al 2018	Poon, C. M., Wong, N. S., Kwan, T. H., Wong, H. T. H., Chan, K. C. W., & Lee, S. S. (2018). Changes of sexual risk behaviors and sexual connections among HIV-positive men who have sex with men along their HIV care continuum. PloS one, 13(12), e0209008. https://doi.org/10.1371/journal.pone.0209008
344	Sandfort et al 2019	Sandfort, T. G. M., Dominguez, K., Kayange, N., Ogendo, A., Panchia, R., Chen, Y. Q., Chege, W., Cummings, V., Guo, X., Hamilton, E. L., Stirratt, M., & Eshleman, S. H. (2019). HIV testing and the HIV care continuum among sub-Saharan African men who have sex with men and transgender women screened for participation in HPTN 075. PloS one, 14(5), e0217501. https://doi.org/10.1371/journal.pone.0217501
345	Uuskula et al 2020	Uusküla, A., Vickerman, P., Raag, M., Walker, J., Paraskevis, D., Eritsyan, K., Sypsa, V., Lioznov, D., Avi, R., & Des Jarlais, D. (2020). Presenting a conceptual framework for an HIV prevention and care continuum and assessing the feasibility of empirical measurement in

Paper Number	Authors	Reference
		Estonia: A case study. PloS one, 15(10), e0240224. https://doi.org/10.1371/journal.pone.0240224
347	Karch et al 2016	Karch, D. L., Gray, K. M., Shi, J., & Hall, H. I. (2016). HIV Infection Care and Viral Suppression Among People Who Inject Drugs, 28 U.S. Jurisdictions, 2012-2013. The open AIDS journal, 10, 127–135. https://doi.org/10.2174/1874613601610010127
350	Robeiro et al 2016	Rebeiro, P. F., Cesar, C., Shepherd, B. E., De Boni, R. B., Cortés, C. P., Rodriguez, F., Belaunzarán-Zamudio, P., Pape, J. W., Padgett, D., Hoces, D., McGowan, C. C., & Cahn, P. (2016). Assessing the HIV Care Continuum in Latin America: progress in clinical retention, cART use and viral suppression. Journal of the International AIDS Society, 19(1), 20636. https://doi.org/10.7448/IAS.19.1.20636
351	Hussen et al 2017	Hussen, S. A., Chakraborty, R., Knezevic, A., Camacho-Gonzalez, A., Huang, E., Stephenson, R., & Del Rio, C. (2017). Transitioning young adults from paediatric to adult care and the HIV care continuum in Atlanta, Georgia, USA: a retrospective cohort study. Journal of the International AIDS Society, 20(1), 21848. https://doi.org/10.7448/IAS.20.1.21848
352	Tapera et al 2019	Tapera, T., Willis, N., Madzeke, K., Napei, T., Mawodzeke, M., Chamoko, S., Mutsinze, A., Zvirawa, T., Dupwa, B., Mangombe, A., Chimwaza, A., Makoni, T. M., Mandewo, W., Senkoro, M., Owiti, P., Tripathy, J. P., & Kumar, A. M. V. (2019). Effects of a Peer-Led Intervention on HIV Care Continuum Outcomes Among Contacts of Children, Adolescents, and Young Adults Living With HIV in Zimbabwe. Global health, science and practice, 7(4), 575–584. https://doi.org/10.9745/GHSP-D-19-00210
126	Mody et al 2020	Mody, A., Glidden, D. V., Eshun-Wilson, I., Sikombe, K., Simbeza, S., Mukamba, N., Somwe, P., Beres, L. K., Pry, J., Bolton-Moore, C., Padian, N., Holmes, C. B., Sikazwe, I., & Geng, E. H. (2020). Longitudinal Care Cascade Outcomes Among People Eligible for Antiretroviral Therapy Who Are Newly Linking to Care in Zambia: A Multistate Analysis. Clinical infectious diseases : an official publication of the Infectious Diseases Society of America, 71(10), e561–e570. https://doi.org/10.1093/cid/ciaa268
127	Wang et al 2019	Wang, L., Krebs, E., Min, J. E., Mathews, W. C., Nijhawan, A., Somboonwit, C., Aberg, J. A., Moore, R. D., Gebo, K. A., Nosyk, B., & HIV Research Network (2019). Combined estimation of disease progression and retention on antiretroviral therapy among treated individuals with HIV in the USA: a modelling study. The

Paper Number	Authors	Reference
		lancet. HIV, 6(8), e531–e539. https://doi.org/10.1016/S2352-3018(19)30148-1
129	Rahmalia et al 2019	Rahmalia, A., Price, M. H., Hartantri, Y., Alisjahbana, B., Wisaksana, R., van Crevel, R., & van der Ven, A. J. A. M. (2019). Are there differences in HIV retention in care between female and male patients in Indonesia? A multi-state analysis of a retrospective cohort study. PloS one, 14(6), e0218781. https://doi.org/10.1371/journal.pone.0218781
130	Gillis et al 2016	Gillis, J., Loutfy, M., Bayoumi, A. M., Antoniou, T., Burchell, A. N., Walmsley, S., Cooper, C., Klein, M. B., Machouf, N., Montaner, J. S., Rourke, S. B., Tsoukas, C., Hogg, R., Raboud, J., & CANOC Collaboration (2016). A Multi-State Model Examining Patterns of Transitioning Among States of Engagement in Care in HIV-Positive Individuals Initiating Combination Antiretroviral Therapy. Journal of acquired immune deficiency syndromes (1999), 73(5), 531–539. https://doi.org/10.1097/QAI.0000000000001109
131	Lee et al 2017	Lee, H., Hogan, J. W., Genberg, B. L., Wu, X. K., Musick, B. S., Mwangi, A., & Braitstein, P. (2017). A state transition framework for patient-level modeling of engagement and retention in HIV care using longitudinal cohort data. Statistics in medicine, 37(2), 302–319. https://doi.org/10.1002/sim.7502
132	Lee et al 2018	Lee, H., Wu, X. K., Genberg, B. L., Mugavero, M. J., Cole, S. R., Lau, B., Hogan, J. W., & Centers for AIDS Research Network of Integrated Clinical Systems (CNICS) Investigators (2018). Beyond binary retention in HIV care: predictors of the dynamic processes of patient engagement, disengagement, and re-entry into care in a US clinical cohort. AIDS (London, England), 32(15), 2217–2225. https://doi.org/10.1097/QAD.0000000000001936
134	Nsanzimana et al 2015	Nsanzimana, S., Kanters, S., Remera, E., Forrest, J. I., Binagwaho, A., Condo, J., & Mills, E. J. (2015). HIV care continuum in Rwanda: a cross-sectional analysis of the national programme. The lancet. HIV, 2(5), e208–e215. https://doi.org/10.1016/S2352-3018(15)00024-7
135	Haber et al 2017	Haber, N., Tanser, F., Bor, J., Naidu, K., Mutevedzi, T., Herbst, K., Porter, K., Pillay, D., & Bärnighausen, T. (2017). From HIV infection to therapeutic response: a population-based longitudinal HIV cascade-of-care study in KwaZulu-Natal, South Africa. The lancet. HIV, 4(5), e223–e230. https://doi.org/10.1016/S2352-3018(16)30224-7

Table S4: Cascades stages used in HIV cascade and continuum of care assessments in articles included in this review

**Cross-sectional**	**Longitudinal**
ALL (HIV negative)	PLHIV
PLHIV (incidence and prevalence)	Diagnosis
Diagnosis	Linkage to care or in HIV care
Linkage to care	Optimal care
ART eligibility	On ART (in care or transferred out or LTFU or re-engaged or death)
Clinical staging	No ART or off-ART (in care or transferred out or LTFU or death)
On ART (history of ART or ever on ART and current use of ART)	ART eligibility
ART adherence	Retention in care
Retention in care	LTFU or disengaged
Viral load suppression	Sub-optimal care
LTFU	Engagement in care
In care	Transferred out
Death	Death
CD4 testing	Viral load suppression or high CD4

Table S5: The reported study design and data analysis methods used to assess the HIV cascade and continuum of care among longitudinal studies (n=21)

Reported study design	Data analysis methods used	Number of articles
Longitudinal	Survival analysis: Competing risk methods	5
Longitudinal	Repeated cross-sectional	1
Longitudinal	Survival analysis	2
Longitudinal	Survival analysis and multistate models	6
Longitudinal	Cross-sectional analysis	5
Longitudinal	Repeated cross-sectional and time spent in the cascade stage	1
Longitudinal	Repeated measure analysis	1

Kalinjuma AV, *et al. BMJ Open* 2023; 13:e071392. doi: 10.1136/bmjopen-2022-071392

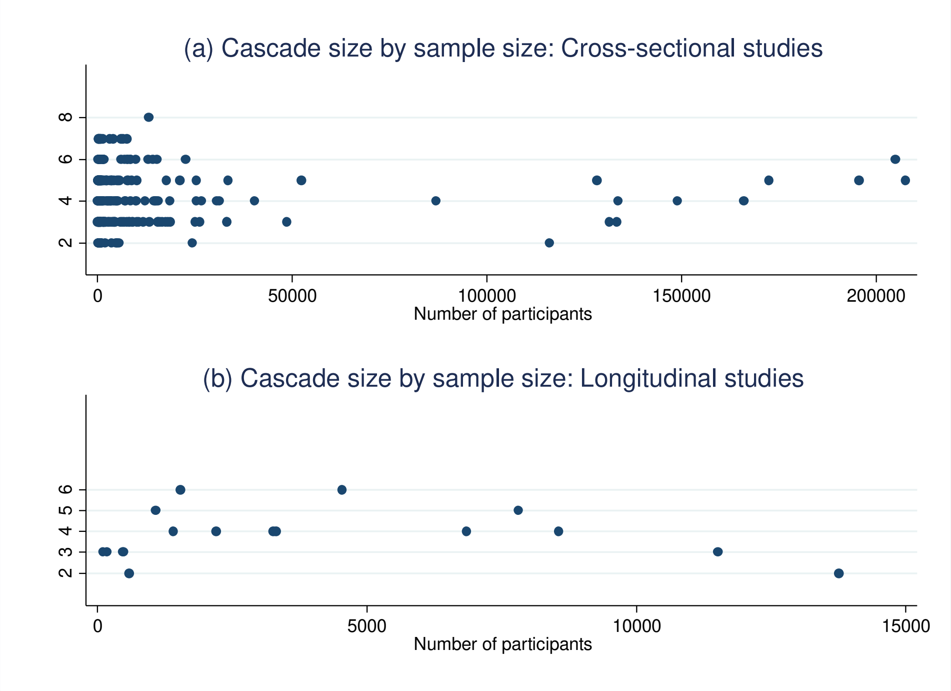

Figure S1: The distribution of cascade size by study number of participants included, in panel (a) sample size was truncated at 300,000 (excluded 12 studies), and in panel (b) sample size was truncated at 20,000 (excluded 4 studies) to allow better visualization of the distribution

(a) Mody et al 2020

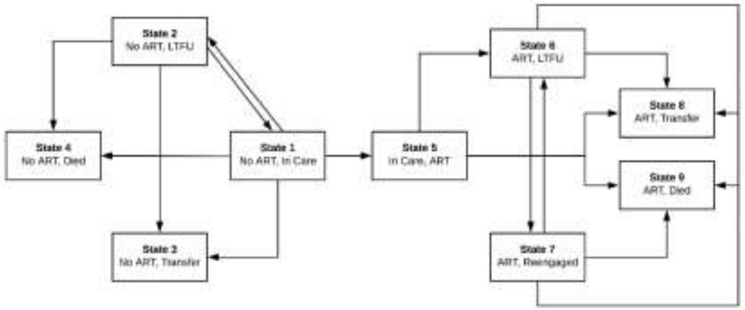

(b) Rahmalia et al 2019

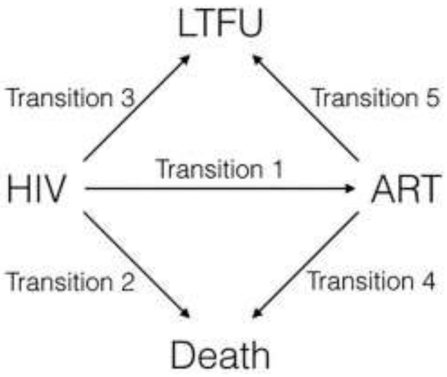

(c) Lee et al 2017

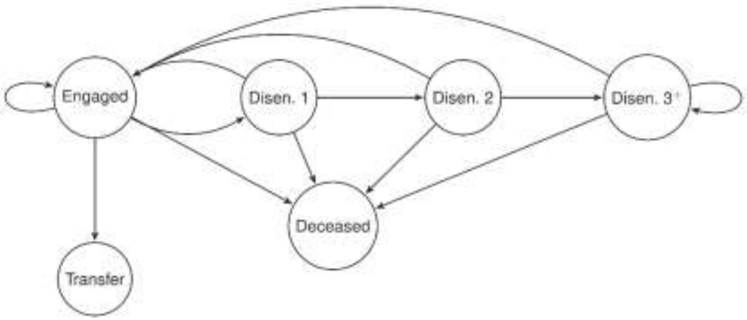

(d) Lee et al 2018

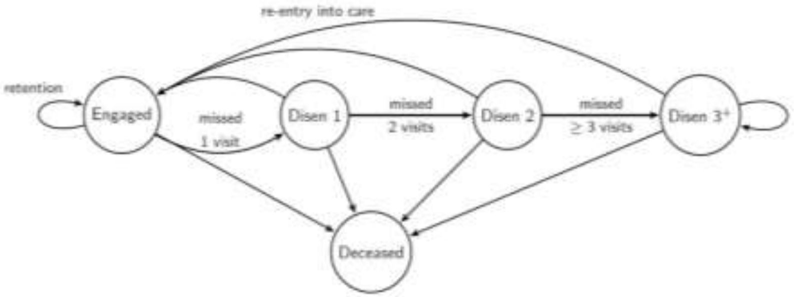

(E) Wang et al 2019

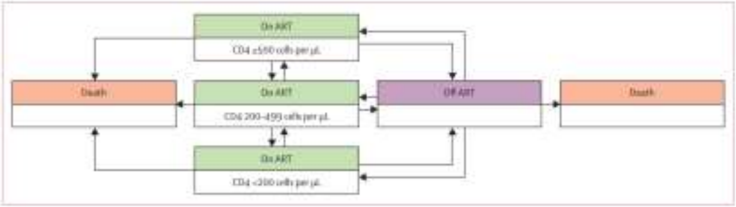	

Figure S2: Multistate frameworks of included articles

Kalinjuma AV, *et al. BMJ Open* 2023; 13:e071392. doi: 10.1136/bmjopen-2022-071392
