# Supplementary material for: Statistical methods applied for the assessment of the HIV cascade and continuum of care: a systematic scoping review
Source: BMJ Open. 2023 Nov 23;13(11):e071392. doi: 10.1136/bmjopen-2022-071392 (PMC10668296; doi:10.1136/bmjopen-2022-071392)
Supplement: Author's manuscript [file bmjopen-2022-071392.draft_revisions.pdf]

BMJ Open

BMJ Open is committed to open peer review. As part of this commitment we make the peer review history of every article we publish publicly available.

When an article is published we post the peer reviewers' comments and the authors' responses online. We also post the versions of the paper that were used during peer review. These are the versions that the peer review comments apply to.

The versions of the paper that follow are the versions that were submitted during the peer review process. They are not the versions of record or the final published versions. They should not be cited or distributed as the published version of this manuscript.

BMJ Open is an open access journal and the full, final, typeset and author-corrected version of record of the manuscript is available on our site with no access controls, subscription charges or pay-per-view fees (http://bmjopen.bmj.com).

If you have any questions on BMJ Open's open peer review process please email
info.bmjopen@bmj.com

BMJ Open

Statistical Methods Applied for the Assessment of the HIV Cascade and Continuum of Care: A Systematic Scoping Review

Journal:	*BMJ Open*
Manuscript ID	bmjopen-2022-071392
Article Type:	Original research
Date Submitted by the Author:	28-Dec-2022

Complete List of Authors:	Kalinjuma, Aneth Vedastus; Ifakara Health Institute; Faculty of Health Sciences University of the Witwatersrand<br>Glass, Tracy Renée; Swiss Tropical and Public Health Institute; University of Basel<br>Masanja, Honorati; Ifakara Health Institute, P.O Box 78373, Dar es Salaam, Tanzania, Health<br>Weisser, Maja; Ifakara Health Institute; University of Basel<br>Msengwa, Amina Suleiman; University of Dar es Salaam, Department of Statistics<br>Vanobberghen, Fiona; Swiss Tropical and Public Health Institute; University of Basel<br>Otwombe, Kennedy; University of the Witwatersrand Perinatal HIV Research Unit, Statistics and Data Management Centre; School of Public Health, Faculty of Health Sciences, University of the Witwatersrand, Johannesburg, South Africa

Keywords:	STATISTICS & RESEARCH METHODS, HIV & AIDS < INFECTIOUS DISEASES, Public health < INFECTIOUS DISEASES

SCHOLARONE™ Manuscripts

I, the Submitting Author has the right to grant and does grant on behalf of all authors of the Work (as defined in the below author licence), an exclusive licence and/or a non-exclusive licence for contributions from authors who are: i) UK Crown employees; ii) where BMJ has agreed a CC-BY licence shall apply, and/or iii) in accordance with the terms applicable for US Federal Government officers or employees acting as part of their official duties; on a worldwide, perpetual, irrevocable, royalty-free basis to BMJ Publishing Group Ltd ("BMJ") its licensees and where the relevant Journal is co-owned by BMJ to the co-owners of the Journal, to publish the Work in this journal and any other BMJ products and to exploit all rights, as set out in our licence.

The Submitting Author accepts and understands that any supply made under these terms is made by BMJ to the Submitting Author unless you are acting as an employee on behalf of your employer or a postgraduate student of an affiliated institution which is paying any applicable article publishing charge ("APC") for Open Access articles. Where the Submitting Author wishes to make the Work available on an Open Access basis (and intends to pay the relevant APC), the terms of reuse of such Open Access shall be governed by a Creative Commons licence – details of these licences and which Creative Commons licence will apply to this Work are set out in our licence referred to above.

Other than as permitted in any relevant BMJ Author's Self Archiving Policies, I confirm this Work has not been accepted for publication elsewhere, is not being considered for publication elsewhere and does not duplicate material already published. I confirm all authors consent to publication of this Work and authorise the granting of this licence.

For peer review only

Statistical Methods Applied for the Assessment of the HIV Cascade and Continuum of Care: A Systematic Scoping Review

Aneth Vedastus Kalinjuma[1,2*], Tracy Renée Glass[3,4], Honorati Masanja[1], Maja Weisser[1,3,4,5], Amina Suleiman Msengwa[6], Fiona Vanobberghen[3,4]†, and Kennedy Otwombe[2,7]†

†Contributed equally

Affiliations

[1]Ifakara Health Institute, Ifakara, Morogoro, Tanzania

[2]School of Public Health, Faculty of Health Sciences, University of the Witwatersrand, Johannesburg, South Africa

[3]Swiss Tropical and Public Health Institute, Basel, Switzerland

[4]University of Basel, Basel, Switzerland.

[5]Division of Infectious Diseases and Hospital Epidemiology, University Hospital Basel, University of Basel, Switzerland

[6]Department of Statistics, University of Dar es Salaam, Tanzania

[7]Perinatal HIV Research Unit, Chris Hani Baragwanath Academic Hospital, Faculty of Health Sciences, University of the Witwatersrand, Johannesburg, South Africa

***Corresponding Author**

Aneth Vedastus Kalinjuma, Ifakara Health Institute, P.O. Box 78383, Mikocheni, Dar es Salaam, Tanzania.

Email: avedastus@ihi.or.tz and Phone: +255 222 774 714

Word count: Abstract = 300 and Main text = 3974

For peer review only

For peer review only - http://bmjopen.bmj.com/site/about/guidelines.xhtml

Abstract

Objective: This scoping review aims to identify and synthesize existing statistical methods used to assess the progress of HIV treatment programs in terms of the HIV cascade and continuum of care among people living with HIV (PLHIV).

Inclusion Criteria: This scoping review included original articles in English that estimated and described the HIV cascade and continuum of care progress among PLHIV. The review considered quantitative articles evaluating either HIV care cascade progress in terms of UNAIDS targets or the dynamics of engagement in HIV care.

Methods: Articles published between April and July 2022 were retrieved from PubMed, CINAHL Complete, and EMBASE databases. The PRISMA-ScR and JBI manual for evidence synthesis (Scoping Review Chapter) guides were used for review protocol development, data handling, and reporting. The review findings were summarised using maps, bar graphs, and descriptive statistics.

Results: This review included 313 articles. Cross-sectional study design methods were most commonly used to assess the HIV care cascade (93%). In cross-sectional and longitudinal studies, the majority used proportions to describe individuals at each cascade stage (99% and 91%, respectively). In longitudinal studies, the time spent in cascade stages, transition probabilities, and cumulative incidence function were estimated. The logistic regression model was common in cross-sectional and longitudinal studies (36% and 35%, respectively). Of the articles that used a longitudinal design, six articles used multistate models, which included non-parametric multistate, parametric, continuous-time multistate, time-homogeneous Markov, and discrete-time multistate Markov models.

Conclusion: Most literature on the HIV cascade and continuum of care arises from cross-sectional study design methods. The use of a longitudinal study design in the HIV cascade is growing, and they provide additional information about the transition dynamics along the cascade. Therefore, a methodological guide for the application of different types of multistate models in this topic could be useful for future studies.

For peer review only - http://bmjopen.bmj.com/site/about/guidelines.xhtml

Keywords: Cross-sectional design; Longitudinal design; Multistate Modelling; HIV care and treatment programs

For peer review only

For peer review only - http://bmjopen.bmj.com/site/about/guidelines.xhtml

Strengths and limitations of this study

Strengths

- This review is among the largest systematic scoping reviews conducted on the methods used to assess the HIV cascade and continuum of care with wide coverage and diversity.
- The review also highlights areas with limited research in terms of methods and their applications to the HIV cascade and continuum of care.

Limitation

- This review was limited to articles written in English. The impact of including other languages in this review is unknown.

For peer review only

For peer review only - http://bmjopen.bmj.com/site/about/guidelines.xhtml

Introduction

The HIV care cascade is a framework that describes the stages of people living with HIV (PLHIV) transitioning from HIV infection, diagnosis, linkage to care, initiation of antiretroviral therapy (ART), retention, and suppression of the viral load (1,2). This framework characterizes and quantifies the proportion of PLHIV at each stage of the cascade (3,4). It is a useful tool for monitoring the uptake of HIV services and the progress of HIV treatment programs (5–7).

In literature, the terms 'HIV care cascade' and 'HIV continuum of care' are used interchangeably (4,5,8,9), but Kay et al. (10) distinguished the two concepts. They defined the 'HIV continuum of care' as a dynamic and bidirectional engagement in HIV care, whereas the 'HIV care cascade' was defined as a static and cross-sectional representation of HIV care stages at the population level. In recent years, many researchers have embraced these concepts, and the distinction between them has been recognised in the literature, particularly after the Joint United Nations Programme on HIV and AIDS (UNAIDS) announced the 90-90-90 targets for HIV for 2020 (11) and 95-95-95 targets for 2025 (12). The 95-95-95 target states that 95% of PLHIV know their HIV status, 95% of those diagnosed positive are initiated on ART, and 95% of those on ART are virally suppressed.

Currently, most HIV treatment program assessments and evaluations are performed following the HIV care cascade approach, which aims to report progress toward the UNAIDS targets for 2020 or 2025 (13–22). These assessments often apply descriptive statistics and they show progress at a particular time point, referred to as cross-sectional design. In recent years, assessments of the HIV cascade and continuum of care that use a longitudinal design have been increasingly featured in published literature. In these applications, methods, such as multistate models (3,5), competing risk methods (23), and the distribution time spent in each cascade stage (24), were used. The existence of various methods and their application in the HIV cascade and continuum of care evaluation calls for a review that identifies the statistical methods used in these assessments.

For peer review only

For peer review only - http://bmjopen.bmj.com/site/about/guidelines.xhtml

Different types of reviews exist on this topic, the majority of which focus on synthesising the estimates of each HIV care cascade stage. These reviews mainly focused on HIV testing, engagement in care and treatment (25,26), cascades among alcohol users (27), and cascades among injecting drug users on methadone (28). Others have conducted a review on cascade stages definitions (29), cascade experiences among Indigenous people (30), cascades among young children and adolescents (31), and cascades among children and adolescents (32).

Currently, few systematic reviews have synthesized the methods used to assess the HIV cascade and continuum of care progress. The first systematic review was conducted by Medland et al. (33) and published in 2015. At that time, few cascade articles were published, and most studies used descriptive statistics in a cross-sectional study design. During 2010–2016, Granich et al. (34) conducted a review of methods used for assessing the progress of the UNAIDS 90-90-90, and the review showed that different methods were used; however, in this review, the focus was mainly on articles that used a cross-sectional study design. In 2021, Vourli et al. (35) published a systematic review that showed the importance of expanding the scope of cascade assessments to include the time to the next cascade stage. In these reviews, the components of the statistical methods applied in the HIV cascade and continuum of care assessments were limited. Therefore, the aim of this systematic scoping review was to identify and summarise the statistical methods used to assess the progress of the HIV treatment program in terms of the HIV cascade and continuum of care among PLHIV using both cross-sectional and longitudinal study designs.

Methods

Review protocol

The scoping review protocol was developed from November to December 2021, following the Joanna Briggs Institute System for the Unified Management, Assessment, and Review of Information (JBI SUMARI) manual for evidence synthesis (Scoping Review Chapter) (36).

For peer review only - http://bmjopen.bmj.com/site/about/guidelines.xhtml

Eligibility criteria

This scoping review included original articles written in English that focused on estimating and describing the HIV cascade and continuum of care among PLHIV. Specifically, the review considered quantitative articles that either evaluated or assessed the HIV care cascade in terms of UNAIDS targets or the dynamics of engagement in HIV care and treatment clinics for the HIV continuum of care. Mixed method articles were included if the quantitative component was described well. The review excluded articles focusing on comparing the HIV care cascade and other cascades, such as the TB care cascade, the hepatitis cascade, and the Prevention of Mother-to-Child Transmission of HIV. The review further excluded review and meta-analysis articles; opinion, editorial and commentary articles; and non-peer-reviewed articles (because the methods sections were not well-described). Finally, the review did not include published conference abstracts or proceedings because the methods were typically not provided in sufficient detail.

Search strategy

The development of an electronic bibliographical database search strategy started with a pilot phase, whereby the concepts of the review were searched independently in PubMed and Google Scholar. The retrieved relevant titles and abstracts were screened to identify keywords to be included in the development of actual electronic database search queries. The database search query was first developed using the PubMed database and was adapted to the Cumulative Index to Nursing and Allied Health Literature (CINAHL) Complete and Excerpta Medica dataBASE (EMBASE) databases as needed (Table S1). The database search dates were from April to July 2022.

Source of the evidence selection process

For peer review only - http://bmjopen.bmj.com/site/about/guidelines.xhtml

Titles and abstracts were retrieved from all three databases, and duplicates were removed based on the digital object identifier and article content. Titles and abstracts were screened for eligibility by the lead author, and irrelevant articles were excluded. All potentially relevant full-text articles were downloaded for review. The lead author and two independent reviewers conducted a full-text review, during which the review criteria were applied. The article search and assessment of inclusion and exclusion criteria were summarised and presented using the Preferred Reporting Items for Systematic Reviews and Meta-Analyses extension for Scoping Reviews (PRISMA-ScR) flow diagram (37).

Data extraction and data entry process

The extracted information included the first author's name and publication year, type of participants, age, country, sample size, study design, study level of representation, and data sources. Other extracted information included cascade stages, cascade size, cascade staging methods, and statistical methods used in assessments of the HIV cascade and continuum of care (Table S2). All extracted information was first entered into an Excel spreadsheet. The data extraction tool was modified and revised whenever necessary, depending on the new information available in the articles and its relevance to this review. The leading author reviewed all extracted data. Disagreements between the reviewers were resolved through consensus. After finalising the data collection template, an electronic data capture system was developed using an Open Data Kit (ODK), and data entry was performed by the reviewers. The information entered in ODK was pooled in Excel with a comma-separated values file format using ODK briefcase.

Data items

The variables used in this review included publication year, which included three groups: 2011–2014, referring to the time between the first cascade report and before the announcement of the UNAIDS 90-90-90 targets; 2015–2019, following the UNAIDS 90-90-90 targets announcement and the implementation

For peer review only - http://bmjopen.bmj.com/site/about/guidelines.xhtml

time; 2020–2022 (by July), being the reporting time for the UNAIDS 90-90-90 targets and the implementation of the new UNAIDS 95-95-95 targets. The study site was country-specific if a study was conducted in one country and regionally if a study was conducted in at least two countries. The study regions included Asia, Europe, Europe, Asia, West Africa (including Burkina Faso and Togo), Eastern and Southern Africa, Latin America, North America (including the United States of America (USA) and Canada), sub-Saharan Africa, and global reports (170 countries).

The study designs included two major groups: cross-sectional and longitudinal. A cross-sectional study design included articles that presented the cascade results of a single time point. A longitudinal design included articles that presented cascade outcomes either repeatedly over time (i.e. repeated cross-sectional study design) or prospective, or retrospective study designs (including cohort data)(38). In this review, studies that included cascade assessments using both cross-sectional and longitudinal designs were included in the longitudinal study design group.

Other variables included data sources (including cohort, laboratory test records, hospital records, surveys, simulated data, surveillance, and clinical trial data) and study level of representation, which were grouped into facility, sub-district/town/municipal/city, state/province, population (when a study estimated the number of PLHIV, diagnosed, and linkage in care), community/district/county, regional, country, and global studies. Another variable was the sample size (the number of diagnosed HIV-positive participants assessed). The sample size was grouped into four categories: <1000, 1000–5000, 6000–10000, and >10000. The types of participants were grouped as all PLHIV, children only, adolescents, adults, HIV key populations, prisoners, veterans, refugees, and military. The HIV key population included men who had sex with men, female sex workers, injecting drug users, and transgender men and women. Finally, the statistical methods applied in the different articles were grouped into two categories: descriptive statistics and regression models. Variables, such as study design, data sources, types of participants, and statistical methods, were allowed for multiple responses (multiple response variables are variables in which the article can have more than one answer in the same variable). The obtained responses were converted into

For peer review only - http://bmjopen.bmj.com/site/about/guidelines.xhtml

individual variables with binary responses, with a 'yes' indicating a response and a 'no' indicating no response for the variable.

The HIV cascade and continuum of care stages varied across articles and stage names with similar meanings were reorganised to formulate simple cascade stage names, such as HIV infection (PLHIV), diagnosis, linkage to care, loss to follow-up (LTFU), retention in care, transfer to other HIV clinics, ART eligibility, on ART, viral load suppression, and death. Articles that used a longitudinal design and multistate models had slightly different names for cascade stages. In this case, stage names were used as they were written by the authors. The size of the cascade and continuum of care frameworks were categorised into four groups based on the number of stages assessed. The four categories were extra-large cascades (> 6 stages), large cascades (5–6 stages), medium cascades (4 stages), and small cascades (≤ 3 stages). The cascade staging method used was grouped into three categories: dependent (also known as conditional), independent, and a combination of the two. In the dependent method, PLHIV were eligible for the next cascade stage if they were in the prior cascade stage, while in the independent method, each cascade stage was derived independent of the prior cascade stage (39).

Data analysis and presentation

The review findings were analysed and summarised using summary statistics, such as counts with percentages, median with interquartile range (IQR), and data ranges. The analysis of the multiple response variables focused on the obtained binary variables. These variables were analysed independently using the frequencies (with percentages) of the responses obtained from the review. Results were presented using tables. The geographical distribution of articles by country was summarised using world maps. Maps were plotted for country-specific articles that used cross-sectional and longitudinal study designs (excluding 29 regional articles). The constructed cascade and continuum of care frameworks were summarised in terms of the size and cascade stages used. All cascade stage names were further summarised using counts, and

For peer review only - http://bmjopen.bmj.com/site/about/guidelines.xhtml

the results were plotted using bar graphs for both designs. The frameworks of a subset of articles that used a longitudinal study design and multistate models were compiled to better document the similarities, differences, and existing gaps. All data management and statistical analyses were performed using Stata software version 14 (StataCorp LP, College Station, Texas, USA).

Patient and Public Involvement

No patient involved

Results

Description of the article selection process

The search query retrieved 5,805 titles and abstracts from all three databases (Figure 1). In the title-screening stage, 5,002 titles were excluded, and 803 titles remained for abstract review. The reasons for excluding the title and abstract were duplicates, articles in press, articles in languages other than English, errors in printing, modelling studies, and note/letter/editorial/commentary articles. Other reasons were pure qualitative articles, articles about other cascades/continuums of care, review articles, study protocols, formative research, and off-topic articles (only mentioned the HIV cascade and/or continuum of care but did not assess it). Of the 803 reviewed abstracts, 322 were identified as meriting full-text reviews, of which 313 were included in the analysis of this review.

Characteristics of articles

The majority of the articles were published between 2015 and 2019 (65%), and a few were published earlier (6%) (Table 1). Various PLHIV groups were assessed, including children, adolescents, adults, migrants,

For peer review only - http://bmjopen.bmj.com/site/about/guidelines.xhtml

For peer review only

prisoners, veterans, the military, and HIV key populations. Key HIV populations were reported in 77 articles, which included men having sex with men (12%), female sex workers (6%), injecting drug users (7%), transgender men (3%), and women (6%).

Assessments of the HIV cascade and continuum of care were performed using a cross-sectional (93%) and longitudinal (7%) design (Table 1). The review included 29 studies conducted in at least two countries or regions or globally. Many of these studies were conducted in Europe (21%) and Eastern and Southern Africa (21%). Focusing on country-specific studies, most of the articles that used a cross-sectional study design were from the USA, Canada, Brazil, Zimbabwe, South Africa, India, China, and Australia (Figure 2). Articles that used a longitudinal study design were mainly from the USA and South Africa.

Articles that used a cross-sectional study design had various levels of representation, with many being sub-district/town/municipal/city-based studies (24%), while for articles that used a longitudinal design, the majority were facility-based studies (35%) (Table 1). The sample size varied across articles, with the smallest study including 22 hard-to-reach individuals and the largest study including 37.9 million PLHIV (UNAIDS global estimates of 170 countries). In articles that used a cross-sectional study design, the median sample size was 1,738 PLHIV (IQR: 545–8,958), while in articles that used a longitudinal design, the median was 3,263 PLHIV (IQR: 1,080–11,510). Various data sources have been used to describe the HIV cascade and continuum of care. In general, the data sources frequently reported were surveys (37%), cohorts (25%), and surveillance data (24%) (Table 1). In articles that used a cross-sectional study design, the survey data source was common (39%), whereas, in a longitudinal design, the cohort data source was often used (52%).

Table 1: Characteristics of articles included in the scoping review (n=313)

Characteristics of articles	Cross-Sectional	Longitudinal	Overall
Papers included[1], n (%)	290 (92.7)	23 (7.4)	313 (100)
Publication year[2], n (%)			
2011 - 2014 (Before 90-90-90)	18 (6.2)	-	18 (5.6)
2015 - 2019 (Post 90-90-90)	186 (64.1)	16 (65.6)	202 (64.5)

For peer review only - http://bmjopen.bmj.com/site/about/guidelines.xhtml

2020 - 2022 (90-90-90 reporting)	86 (29.7)	7 (30.4)	93 (29.7)
Regional studies (involved ≥2 countries), n (%)			
Asia	2 (7.1)	-	2 (6.9)
Europe	5 (17.9)	1 (100.0)	6 (20.7)
Europe and Asia	4 (14.3)	-	4 (13.8)
West Africa[3]	1 (3.6)	-	1 (3.5)
Eastern and Southern Africa	6 (21.4)	-	6 (20.7)
Latin America	4 (14.3)	-	4 (13.8)
North america[4]	1 (3.6)	-	1 (3.5)
Sub-Saharan Africa	4 (14.3)	-	4 (13.8)
Global	1 (3.6)	-	1 (3.5)
Type of participants[5], n (%)			
Mixture (All PLHIV)	47 (16.3)	3 (13.0)	50 (16.0)
Children only	10 (3.5)	1 (4.4)	11 (3.5)
Adolescents	30 (10.4)	5 (21.7)	35 (11.2)
Adult	134 (46.4)	15 (65.2)	149 (47.8)
Key population	73 (25.3)	4 (17.4)	77 (24.7)
Migrants	5 (1.7)	-	5 (1.6)
Prisoners	2 (0.7)	-	2 (0.6)
Veterans	2 (0.7)	-	2 (0.6)
Refugees	-	1 (4.4)	1 (0.3)
Military	-	1 (4.4)	1 (0.3)
Types of HIV key population[5], n (%)			
Men having sex with men	34 (11.8)	3 (13.0)	37 (11.9)
Female sex workers	19 (6.6)	-	19 (6.1)
Injected drug users	20 (6.9)	1 (4.4)	21 (6.7)
Transgender women	19 (6.6)	-	19 (6.1)
Transgender men	8 (2.8)	1 (4.4)	9 (2.9)
Data sources[5], n (%)			
Cohort data	67 (23.2)	12 (52.2)	79 (25.3)
Laboratory test records	10 (3.5)	-	10 (3.2)
Hospital medical records	56 (19.3)	5 (21.7)	61 (19.5)
Survey data	112 (38.8)	3 (13.0)	115 (36.9)
Simulated data (mathematical modeling)	11 (3.8)	-	11 (3.5)
Surveillance data	72 (24.9)	3 (13.0)	75 (24.0)
Clinical trials	4 (1.4)	1 (4.4)	5 (1.6)
Others[6]	18 (6.2)	3 (13.0)	21 (6.7)
Sample size, median (IQR)	1738 (545-8958)	3263 (1080-11510)	1897 (578-9054)
Sample size range (minimum, maximum)	22 - 39000000	100 - 92215	22 - 39000000
Sample size (number of participants), n (%)			
<1000	105 (36.8)	5 (21.7)	110 (35.7)
1000 - 5000	88 (30.9)	9 (39.1)	97 (31.5)
6000 - 10000	25 (8.9)	3 (13.0)	28 (9.1)
>10000	67 (23.5)	6 (26.1)	73 (23.7)
Level of representation, n (%)			
Facility	52 (18.0)	7 (35.0)	59 (19.1)
Sub-district/Town/Municipal/City	68 (23.5)	3 (15.0)	71 (23.0)
State/Provide	38 (13.2)	-	38 (12.3)
Population	58 (20.1)	2 (10.0)	60 (19.4)

For peer review only - http://bmjopen.bmj.com/site/about/guidelines.xhtml

Community/District/County	32 (11.2)	3 (15.0)	35 (11.3)
Regional	19 (6.6)	2 (10.0)	21 (6.8)
Country	21 (7.3)	3 (15.0)	24 (7.8)
Global	1 (0.4)	-	1 (0.3)

[1]Row percentage
[2]The UNAIDS 90-90-90 target of the year 2020 stated that 90% of PLHIV know their HIV status, 90% of the diagnosed positive are initiated on ART, and 90% of those on ART are virally suppressed.
[3]Study included in Burkina Faso and Togo
[4]Study included the United States of America and Canada
[5]Percentage was estimated for each type of response and the report in this table is for 'yes' for all binary variables processed from multiple responses variables
[6]Other data sources largely included clinic and program registries, databases, different types of medical records, consortiums, population estimates reports, social insurance schemes, and AIDS program databases.

HIV cascade and continuum of care stages

In general, a median of four cascade stages (IQR: 3–5) was used (Table 2). The smallest cascade had two stages, and the largest cascade had nine stages for both study designs. Approximately 34% of the articles reported using large cascades in articles used a cross-sectional study design, while articles that used a longitudinal study design mostly used small cascades (35%). Furthermore, there was no clear pattern between sample size and the number of stages used in articles that used cross-sectional study design (Figure S1). In articles that used a longitudinal design, the larger the study the smaller the cascade. In all articles reviewed, 71% used dependent staging methods. In articles that used a cross-sectional study design, only 7% used both dependent and independent methods (Table 2).

Table 2: Summary of various forms of outcome used in articles included in the scoping review (n=313)

Outcome	Cross-sectional	Longitudinal	Overall
Number of cascade stages, median (IQR)	4 (3-5)	4 (3-5)	4 (3-5)
Size of the HIV care cascade, n (%)			
Extra-large cascade (> 6 stages)	17 (5.9)	2 (8.7)	19 (6.1)
Large cascade (5-6 stages)	98 (33.8)	7 (30.4)	105 (33.6)
Medium cascade (4 stages)	80 (27.8)	6 (26.1)	86 (27.5)
Small cascade (≤3 stages)	95 (32.8)	8 (34.8)	103 (32.9)
Cascade staging approaches[1], n (%)			

For peer review only - http://bmjopen.bmj.com/site/about/guidelines.xhtml
For peer review only

Dependent	189 (71.3)	7 (58.3)	196 (70.8)
Independent	57 (21.5)	4 (33.3)	61 (22.0)
Both dependent and independent	19 (7.2)	1 (8.3)	20 (7.2)

[1]Studies used longitudinal and multistate models or survival analyses, staging methods for each cascade stage are not applicable

In the articles that used a cross-sectional study design, different cascade stages were used. Broadly across all nine stages listed, there were five stages most frequently reported: PLHIV, diagnosis, linkage to care, ART status, and viral load suppression status (Figures S2 and S3). Most articles used diagnosis (n=157) or PLHIV (n=38) as the first stage (Figure S2). In the second stage, most articles used diagnosis (81 articles) and linkage to care (90 articles). From stages three to seven, the most common stages were ART status and viral load suppression status (Figures S2 and S3).

In a longitudinal study design, the reported stages can be grouped into four broad categories: diagnosis, linkage to care, ART status, and viral load status (Figures S4 and S5). Finally, two articles used a longitudinal design formulated stages in combination with either ART status or CD4 cell count (Figure S6).

Statistical methods applied

In the descriptive analyses, the majority of the articles used proportions to describe participants at each cascade stage (99% for cross-sectional design and 91% for longitudinal design) (Table 3). This review showed that articles that used a longitudinal study design also estimated transition probabilities between cascade stages (22%) compared to those that used a cross-sectional design (0.6%). Furthermore, the time spent in each cascade stage and the Kaplan–Meier or cumulative incidence function was used in the longitudinal studies. Approximately 30% of the articles in both designs used simple test statistics, such as the chi-square test, Fisher exact test, t-test, Wilcoxon rank-sum test, and the Kruskal–Wallis test.

To assess the association between cascade stages or transitions between stages and participant characteristics, 180 (58%) of the included articles performed regression analysis. The regression models

For peer review only - http://bmjopen.bmj.com/site/about/guidelines.xhtml

For peer review only

used in the reviewed articles primarily considered discrete and survival outcomes (Table 3). In the articles that used a cross-sectional study design, the logistic regression model was the most common (36%). Logistic and Cox proportional hazard models were commonly used in studies that used a longitudinal design (35% and 17%, respectively).

Table 3: Statistical methods used in articles included in the scoping review (n=313)

Methods	Cross-sectional	Longitudinal	Overall
Descriptive statistics[1], n (%)			
Proportion for each stage of the HIV care cascade	287 (99.3)	21 (91.3)	308 (98.7)
Simple test statistics[2]	81 (27.9)	6 (26.9)	87 (27.8)
Time spent in each stage of the HIV care cascade	7 (2.4)	6 (26.1)	13 (4.2)
Transition probabilities	1 (0.3)	5 (21.7)	6 (1.9)
Kaplan-Meier or cumulative incidence function	-	6 (26.1)	6 (1.9)
Regression models[1], n (%)			
Logistic model	104 (36.0)	8 (34.8)	112 (35.9)
Cox PH model	15 (5.2)	4 (17.4)	19 (6.1)
Poisson model	32 (11.1)	-	32 (10.3)
Competing risk model	3 (1.0)	-	3 (1.0)
Generalized linear model	10 (3.5)	-	10 (3.2)
Weighted and unweighted linear model	4 (1.4)	-	4 (1.3)
Log-Binomial regression model	5 (1.7)	-	5 (1.6)
Multinomial regression model	-	3 (16.7)	3 (1.5)
Generalized estimating equation model	1 (0.3)	1 (4.4)	2 (0.6)
Other regression models[3]	19 (6.6)	3 (13.0)	22 (7.1)

[1]Percentage was estimated for each type of response and the report in this table if for yes for all binary variables processed from multiple responses variables

[2]Simple test statistics used included the Chi-Square test, Fisher-Exact test, t-test, Wilcoxon rank sum test, and Kruskal–Wallis test

[3]Other regression models applied were ARIMA, bivariable and multivariable regression, difference in differences, generalized additive model, generalized structural equation model, and negative binomial model, generalized linear mixed model, spectrum model. generalized linear mixed models

Among the 23 articles that used a longitudinal study design, the review showed that different analysis methods were used. Five studies analysed the cascade using cross-sectional design methods (Table S3). The articles that used longitudinal analysis methods were grouped into five groups: survival analyses (n=2), survival analysis with competing risk methods (n=6), repeated cross-sectional methods (n=2), survival analysis with multistate models (n=6), and repeated measure analysis methods (n=2).

For peer review only - http://bmjopen.bmj.com/site/about/guidelines.xhtml

334 In articles that used a longitudinal study design and multistate models (n=6), five types of multistate models
335 were used. These included the non-parametric multistate, parametric, continuous-time multistate, time-
336 homogeneous Markov, discrete-time multistate Markov, and general multistate models (Table 4). Three
337 articles adopted the Markov process, and one study considered the Markov process with first-order
338 dependence only. In almost all the reviewed articles (n=5), the assessment of the care cascade started at
339 enrolment in HIV care, and one article considered ART initiation as the baseline. Regardless of the type of
340 multistate model used, death was considered an absorbing state in all the reviewed articles. Other absorbing
341 states included LTFU and transfers to other HIV clinics. Three studies used a model that allowed back and
342 forth for LTFU and disengagement states (Figure S6). The review shows that only two articles assessed the
343 goodness of fit of the multistate model, and the methods used were the Pearson chi-square test and plots
344 that compared fitted and observed values.

345

Table 4: Articles used longitudinal study design and multistate model (n=6)

Methods	Number of articles
Baseline	
Enrolment/recruitment	5
ART initiation	1
Reason for censoring	
End of follow-up	2
Transfer, death, and follow-up	1
Transfer out, death, and status less than 12 months	1
Absorbing state	
Death	2
Death and LTFU	2
Death and transfer out	2
Types of multistate models	
Non-Parametric multistate model	1
Parametric, continuous-time multistate	1
Time-homogeneous Markov model	1
Discrete-time multistate Markov model	2
General multistate model	1
Multistate model assumptions	
First-order dependence	1
Markov process	2
Markov, first-order dependence	1
Goodness of fit methods	

For peer review only - http://bmjopen.bmj.com/site/about/guidelines.xhtml

Person-type Chi-Square test	1
Visual comparison of observed and fitted value plots	1
Missing data	
Multiple imputations	2
Missing data indicator	3

Discussion

The findings of this scoping review showed that cross-sectional study design methods are most commonly used for assessing the progress of HIV service uptake and treatment responses (93%). The reviewed articles were mainly available at the subnational level. The articles reported a cross-sectional study design covering a wide range of PLHIV, including children, adolescents, adults, migrants, prisoners, and key populations. In general, the cascade size had a median of four stages (IQR: 3–5). The dependent staging method was often used in articles with a cross-sectional study design. In a subset of articles that used a longitudinal study design (n=6), three articles used analytical frameworks that integrated the in- and out-of-care movements of PLHIV enrolled in care. Although the challenges of using a cross-sectional design were pointed out by cascade researchers in 2015 (4,6), articles using a longitudinal design and addressing the in- and out-of-care pathways in cascade assessments are scarce.

Several authors have proposed different types of frameworks that include various pathways through the HIV cascade and the continuum of care. Researchers have proposed a framework that incorporates the in-and-out of HIV care using state and transition frameworks (Figure S6). In this group of frameworks, most of the possible pathways of engagement in care were addressed by including follow-up statuses, such as LTFU or disengagement from care, transfer to other HIV clinics, and death. Others proposed the 'HIV states and Transition' framework (6), 'comprehensive HIV care cascade' (40), and 'Queueing model' (41). This group of frameworks addresses the dynamics of HIV care engagement while preserving the original structure of the HIV care cascade designed in a cross-sectional design. Therefore, both proposed frameworks can be viewed as an extension of the standard HIV care cascade framework at the stage of linkage to HIV care. That is after PLHIV is diagnosed and successfully linked to care, engagement in care

For peer review only - http://bmjopen.bmj.com/site/about/guidelines.xhtml

is not linear. Regardless of ART status and viral load suppression status, PLHIV may be lost from care, return to care, transferred out, or die.

The review findings showed that different multistate models were applied to a subset of articles that used a longitudinal study design. These included parametric and non-parametric multistate, continuous-time and discrete-time multistate, and general multistate models. A multistate model is a continuous-time stochastic process allowing individuals to move between a finite number of states (42). In the HIV cascade and continuum of care, states correspond to the HIV cascade stages (5,6,43). These included HIV diagnosis, linkage to care, ART initiation, retention in care, LTFU, viral load suppression, and death. In multistate modelling frameworks, states can either be transient (participants can move in and out of the state) or absorbing (no transition is possible out of the state) (44). In this review, articles that used multistate models considered death and transfer to other HIV clinics to be absorbing states. Depending on the availability of data, LTFU and disengagement from care states were allowed to be transient or absorbing states (Figure S6).

In general, there are two broad types of multistate models: discrete-time multistate models for equally spaced longitudinal data and continuous-time multistate models for unequally spaced longitudinal data (45). Clinic visits by PLHIV are likely to be irregular. Researchers using discrete-time multistate models converted unequal follow-up times to equally spaced time intervals by using a window of either 200 days (5) or 12 months (3). In this case, the events were captured within a predefined time interval. However, this is challenging when individuals experience multiple events within a defined time interval. In this situation, Gillis et al. (3) used the predominant event. Another challenge is the incomplete time interval at the end of the follow-up period. In such a situation, events that occurred are sometimes discarded (5), while others included events regardless of the incomplete time interval (3).

The selected multistate model informs the regression models to use for assessing the association between the transition between states and the participants' characteristics. Survival models are used when the continuous-time multistate model is adapted, whereas multinomial regression models are used in discrete-

For peer review only - http://bmjopen.bmj.com/site/about/guidelines.xhtml

time multistate models (45). In this review, two articles used multinomial regression models (5,8), while four articles used the Cox proportional hazard regression model (3,43,46,47). Furthermore, the application of survival models mainly focused on time-homogenous models, and few applied non-homogenous models (47). Therefore, studies that compare time-homogeneous and time-inhomogeneous assumptions in the HIV cascade and continuum of care are crucial for guiding future studies.

Conclusion

Most literature on the HIV cascade and continuum of care applied cross-sectional study design methods. The use of a longitudinal study design in the assessment of the HIV cascade is growing, and the methods provide additional information about the transition dynamics along the cascade, establish detailed progress, and identify additional areas to intervene. Furthermore, the relation between the frameworks used in a longitudinal study design with multistate models and a cross-sectional study design is not very clear. Multistate model frameworks can be viewed as extensions of cross-sectional design frameworks in terms of engagement in HIV care. However, efforts to integrate ART status and viral load suppression status into analyses using these frameworks are crucial. Therefore, a methodological guide on the application of different types of multistate models in the HIV cascade and continuum care and their corresponding frameworks is needed to guide future studies.

For peer review only - http://bmjopen.bmj.com/site/about/guidelines.xhtml

Statements

Author contributions

AVK designed the study, obtained funding, implemented the review, and prepared the review manuscript. KO and FV supervised the study from its design to its implementation. TRG, MW, ASM, and HM participated in the study design and reviewed the manuscript. All authors reviewed and approved the final manuscript.

Conflict of interest

Maja Weiser was sponsored by different sponsors to attend eccmid conferences in the last 10 years and she is a member of the Menari advisory board in 2022. All other authors declared no conflict of interest.

Funding

This research was supported by the Consortium for Advanced Research Training in Africa (CARTA). CARTA is jointly led by the African Population and Health Research Center and the University of the Witwatersrand and funded by the Carnegie Corporation of New York (Grant No. G-19-57145), Sida (Grant No:**54100113**), Uppsala Monitoring Center, Norwegian Agency for Development Cooperation (Norad), and by the Wellcome Trust [reference no. 107768/Z/15/Z] and the UK Foreign, Commonwealth & Development Office, with support from the Developing Excellence in Leadership, Training and Science in Africa (DELTAS Africa) programme. The statements made and views expressed are solely the responsibility of the Fellow.

For peer review only - http://bmjopen.bmj.com/site/about/guidelines.xhtml

Role of the funder

The funder of this systematic scoping review had no role in review design, data extraction, synthesis of results and interpretation, preparation of the review manuscript, and the choice of journal for submission of the manuscript for publication.

Data availability

All review data extracted and processed is available upon request through the corresponding author.

Ethical approval

This study is part of the Ph.D. project that was approved by the University of the Witwatersrand Human Research Ethics Committee (Medical) with reference number R14/49, but this specific study did not involve human participants.

Acknowledgments

Apatsa Selemani: Librarian and CARTA fellow from the Kamuzu University of Health Sciences (formerly College of Medicine and Kamuzu College of Nursing of the University of Malawi), Malawi. The librarian contributed to the development of the review search strategies for various databases.

Samwel Godfrey Lwambura (Statistician and Data Officer) and Jacqueline Minja (Statistician and Research Scientist), Ifakara Health Institute, Tanzania. Research assistants assisted with the full-text review, data extraction, and data entry.

Dr. Yeromin Mlacha (Medical Entomologist and Senior Researcher), Ifakara Health Institute, Tanzania. Assisted with data preparation for maps of the included articles.

For peer review only - http://bmjopen.bmj.com/site/about/guidelines.xhtml

For peer review only

References

1. Gardner EM, McLees MP, Steiner JF, del Rio C, Burman WJ. The Spectrum of Engagement in HIV Care and its Relevance to Test-and-Treat Strategies for Prevention of HIV Infection. Clin Infect Dis. 2011 Mar 15;52(6):793–800.

2. Centers for Disease Control and Prevention (CDC). Vital signs: HIV prevention through care and treatment--United States. MMWR Morb Mortal Wkly Rep. 2011 Dec 2;60(47):1618–23.

3. Gillis J, Loutfy M, Bayoumi AM, Antoniou T, Burchell AN, Walmsley S, et al. A Multi-State Model Examining Patterns of Transitioning Among States of Engagement in Care in HIV-Positive Individuals Initiating Combination Antiretroviral Therapy. J Acquir Immune Defic Syndr. 2016 Dec 15;73(5):531–9.

4. Blitz S, Antoniou T, Burchell A, Walmsley S, Light L, Gardner S, et al. The Use of Multistate Models to Examine Associations of Stress and Adherence With Transitions Among HIV Care States Observed in a Clinical HIV Cohort. J Acquir Immune Defic Syndr. 2017 01;76(3):303–10.

5. Lee H, Hogan JW, Genberg BL, Wu XK, Musick BS, Mwangi A, et al. A state transition framework for patient-level modeling of engagement and retention in HIV care using longitudinal cohort data. Statistics in Medicine. 2018;37(2):302–19.

6. Powers KA, Miller WC. Building on the HIV cascade: a complementary "HIV States and Transitions" framework for describing HIV diagnosis, care, and treatment at the population level. J Acquir Immune Defic Syndr. 2015 Jul 1;69(3):341–7.

7. 2017 HIV Continuum of Care | CDC [Internet]. 2019 [cited 2020 Mar 29]. Available from: https://www.cdc.gov/nchhstp/newsroom/2017/HIV-Continuum-of-Care.html

8. Lee H, Wu XK, Genberg BL, Mugavero MJ, Cole SR, Lau B, et al. Beyond binary retention in HIV care: predictors of the dynamic processes of patient engagement, disengagement, and re-entry into care in a US clinical cohort. AIDS. 2018 24;32(15):2217–25.

9. Barrow GJ, Brandeau ML. A modified HIV continuum of care: A six-year evaluation of a viral load cascade at a hospital-based clinic in Kingston, Jamaica. Int J STD AIDS. 2019 Jul;30(8):748–55.

10. Kay ES, Batey DS, Mugavero MJ. The HIV treatment cascade and care continuum: updates, goals, and recommendations for the future. AIDS Research and Therapy. 2016 Nov 8;13(1):35.

11. 90-90-90: An ambitious treatment target to help end the AIDS epidemic. :40.

12. prevailing-against-pandemics_en.pdf [Internet]. [cited 2021 Mar 3]. Available from: https://aidstargets2025.unaids.org/assets/images/prevailing-against-pandemics_en.pdf

13. Van Beckhoven D, Florence E, Ruelle J, Deblonde J, Verhofstede C, Callens S, et al. Good continuum of HIV care in Belgium despite weaknesses in retention and linkage to care among migrants. BMC Infect Dis. 2015 Nov 3;15(1):496.

14. Marinda E, Simbayi L, Zuma K, Zungu N, Moyo S, Kondlo L, et al. Towards achieving the 90-90-90 HIV targets: results from the south African 2017 national HIV survey. BMC Public Health. 2020 Sep 9;20(1):1375.

For peer review only - http://bmjopen.bmj.com/site/about/guidelines.xhtml

15. Cowan FM, Davey CB, Fearon E, Mushati P, Dirawo J, Cambiano V, et al. The HIV Care Cascade Among Female Sex Workers in Zimbabwe: Results of a Population-Based Survey From the Sisters Antiretroviral Therapy Programme for Prevention of HIV, an Integrated Response (SAPPH-IRe) Trial. J Acquir Immune Defic Syndr. 2017 Apr 1;74(4):375–82.

16. Engelhard EAN, Smit C, Van Sighem A, Reiss P, Nieuwkerk PT, Kroon FP, et al. Impact of HIV care facility characteristics on the cascade of care in HIV-infected patients in the Netherlands. AIDS. 2016 Jan;30(2):301–10.

17. Haber N, Tanser F, Bor J, Naidu K, Mutevedzi T, Herbst K, et al. From HIV infection to therapeutic response: a population-based longitudinal HIV cascade-of-care study in KwaZulu-Natal, South Africa. Lancet HIV. 2017 May;4(5):e223–30.

18. Anglemyer A, Haber N, Noiman A, Rutherford G, Ganesan A, Blaylock J, et al. HIV Care Continuum and Meeting 90-90-90 Targets: Cascade of Care Analyses of a U.S. Military Cohort. Mil Med. 2020 Aug 14;185(7–8):e1147–54.

19. Elgalib A, Shah S, Al-Wahaibi A, Al-Habsi Z, Al-Fouri M, Lau R, et al. Disparities between HIV patient subgroups in Oman: An analysis of the 2019 cascade of care. PLoS One. 2021;16(7):e0254474.

20. Vogler IH, Alfieri DF, Gianjacomo HDB, Almeida ERD de, Reiche EMV. Cascade of care for people living with HIV infection in Southern Brazil: results from a public health network. Cad Saude Publica. 2018 Nov 29;34(12):e00009718.

21. Maman D, Zeh C, Mukui I, Kirubi B, Masson S, Opolo V, et al. Cascade of HIV care and population viral suppression in a high-burden region of Kenya. AIDS. 2015 Jul 31;29(12):1557–65.

22. Lourenço L, Colley G, Nosyk B, Shopin D, Montaner JSG, Lima VD, et al. High Levels of Heterogeneity in the HIV Cascade of Care across Different Population Subgroups in British Columbia, Canada. PLOS ONE. 2014 Dec 26;9(12):e115277.

23. LESKO CR, EDWARDS JK, MOORE RD, LAU B. A longitudinal, HIV care continuum: 10-year restricted mean time in each care continuum stage after enrollment in care, by history of injection drug use. AIDS. 2016 Sep 10;30(14):2227–34.

24. Supervie V, Marty L, Lacombe JM, Dray-Spira R, Costagliola D, FHDH-ANRS CO4 study group. Looking Beyond the Cascade of HIV Care to End the AIDS Epidemic: Estimation of the Time Interval From HIV Infection to Viral Suppression. J Acquir Immune Defic Syndr. 2016 01;73(3):348–55.

25. Iroh PA, Mayo H, Nijhawan AE. The HIV Care Cascade Before, During, and After Incarceration: A Systematic Review and Data Synthesis. Am J Public Health. 2015 Jul;105(7):e5-16.

26. Stannah J, Dale E, Elmes J, Staunton R, Beyrer C, Mitchell KM, et al. HIV testing and engagement with the HIV treatment cascade among men who have sex with men in Africa: a systematic review and meta-analysis. Lancet HIV. 2019 Nov;6(11):e769–87.

27. Vagenas P, Azar MM, Copenhaver MM, Springer SA, Molina PE, Altice FL. The Impact of Alcohol Use and Related Disorders on the HIV Continuum of Care: a Systematic Review : Alcohol and the HIV Continuum of Care. Curr HIV/AIDS Rep. 2015 Dec;12(4):421–36.

For peer review only - http://bmjopen.bmj.com/site/about/guidelines.xhtml

28. Mohd Salleh NA, Voon P, Karamouzian M, Milloy MJ, Richardson L. Methadone maintenance therapy service components linked to improvements in HIV care cascade outcomes: A systematic review of trials and observational studies. Drug Alcohol Depend. 2021 Jan 1;218:108342.

29. Mugglin C, Kläger D, Gueler A, Vanobberghen F, Rice B, Egger M, et al. The HIV care cascade in sub-Saharan Africa: systematic review of published criteria and definitions. J Int AIDS Soc. 2021 Jul;24(7):e25761.

30. Jongbloed K, Pooyak S, Sharma R, Mackie J, Pearce ME, Laliberte N, et al. Experiences of the HIV Cascade of Care Among Indigenous Peoples: A Systematic Review. AIDS Behav. 2019 Apr;23(4):984–1003.

31. Zanoni BC, Mayer KH. The adolescent and young adult HIV cascade of care in the United States: exaggerated health disparities. AIDS Patient Care STDS. 2014 Mar;28(3):128–35.

32. Bobat R, Archary M, Lawler M. An update on the HIV treatment cascade in children and adolescents. Curr Opin HIV AIDS. 2015 Nov;10(6):411–9.

33. Medland NA, McMahon JH, Chow EPF, Elliott JH, Hoy JF, Fairley CK. The HIV care cascade: a systematic review of data sources, methodology and comparability. J Int AIDS Soc. 2015;18:20634.

34. Granich R, Gupta S, Hall I, Aberle-Grasse J, Hader S, Mermin J. Status and methodology of publicly available national HIV care continua and 90-90-90 targets: A systematic review. PLoS Med. 2017 Apr;14(4):e1002253.

35. Vourli G, Katsarolis I, Pantazis N, Touloumi G. HIV continuum of care: expanding scope beyond a cross-sectional view to include time analysis: a systematic review. BMC Public Health. 2021 Sep 17;21(1):1699.

36. JBI Manual for Evidence Synthesis - JBI Global Wiki [Internet]. [cited 2022 Oct 20]. Available from: https://jbi-global-wiki.refined.site/space/MANUAL

37. Tricco AC, Lillie E, Zarin W, O'Brien KK, Colquhoun H, Levac D, et al. PRISMA Extension for Scoping Reviews (PRISMA-ScR): Checklist and Explanation. Ann Intern Med. 2018 Oct 2;169(7):467–73.

38. Caruana EJ, Roman M, Hernández-Sánchez J, Solli P. Longitudinal studies. J Thorac Dis. 2015 Nov;7(11):E537–40.

39. Horberg MA, Hurley LB, Klein DB, Towner WJ, Kadlecik P, Antoniskis D, et al. The HIV Care Cascade Measured Over Time and by Age, Sex, and Race in a Large National Integrated Care System. AIDS Patient Care STDS. 2015 Nov;29(11):582–90.

40. McNairy ML, Lamb MR, Abrams EJ, Elul B, Sahabo R, Hawken MP, et al. Use of a Comprehensive HIV Care Cascade for Evaluating HIV Program Performance: Findings From 4 Sub-Saharan African Countries. J Acquir Immune Defic Syndr. 2015 Oct 1;70(2):e44–51.

41. Gonsalves GS, Paltiel AD, Cleary PD, Gill MJ, Kitahata MM, Rebeiro PF, et al. A Flow-Based Model of the HIV Care Continuum in the United States. J Acquir Immune Defic Syndr. 2017 Aug 15;75(5):548–53.

For peer review only

For peer review only - http://bmjopen.bmj.com/site/about/guidelines.xhtml

42. Meira-Machado L, de Uña-Álvarez J, Cadarso-Suárez C, Andersen PK. Multi-state models for the analysis of time-to-event data. Stat Methods Med Res. 2009 Apr 1;18(2):195–222.

43. Mody A, Glidden DV, Eshun-Wilson I, Sikombe K, Simbeza S, Mukamba N, et al. Longitudinal Care Cascade Outcomes Among People Eligible for Antiretroviral Therapy Who Are Newly Linking to Care in Zambia: A Multistate Analysis. Clin Infect Dis. 2020 Dec 17;71(10):e561–70.

44. Andersen PK, Pohar Perme M. Inference for outcome probabilities in multi-state models. Lifetime Data Anal. 2008 Sep 13;14(4):405.

45. Wan L, Lou W, Abner E, Kryscio RJ. A comparison of time-homogeneous Markov chain and Markov process multi-state models. Commun Stat Case Stud Data Anal Appl. 2016;2(3–4):92–100.

46. Rahmalia A, Price MH, Hartantri Y, Alisjahbana B, Wisaksana R, Crevel R van, et al. Are there differences in HIV retention in care between female and male patients in Indonesia? A multi-state analysis of a retrospective cohort study. PLOS ONE. 2019 Jun 25;14(6):e0218781.

47. Wang L, Krebs E, Min JE, Mathews WC, Nijhawan A, Somboonwit C, et al. Combined estimation of disease progression and retention on antiretroviral therapy among treated individuals with HIV in the USA: a modelling study. Lancet HIV. 2019 Aug;6(8):e531–9.

For peer review only

For peer review only - http://bmjopen.bmj.com/site/about/guidelines.xhtml

Figure captions

Figure 1: Flow chart of inclusion and exclusion of research articles

Figure 2: The geographical location distribution of country-specific studies of cross-sectional (n=262) and longitudinal studies (n=22) (regional studies, such as sub-Saharan Africa or Europe, were not included)

For peer review only

For peer review only - http://bmjopen.bmj.com/site/about/guidelines.xhtml

Figure 1

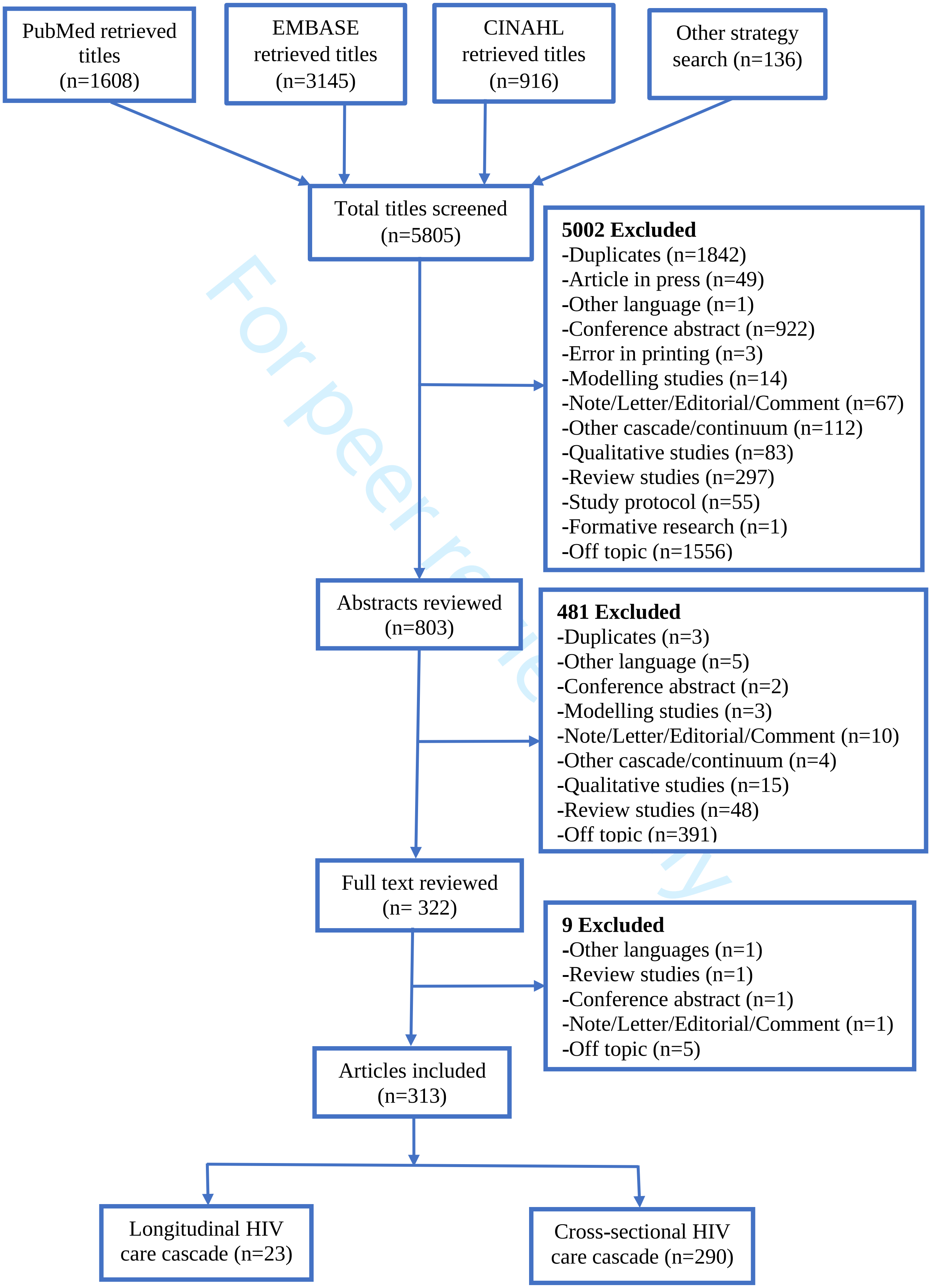

For peer review only

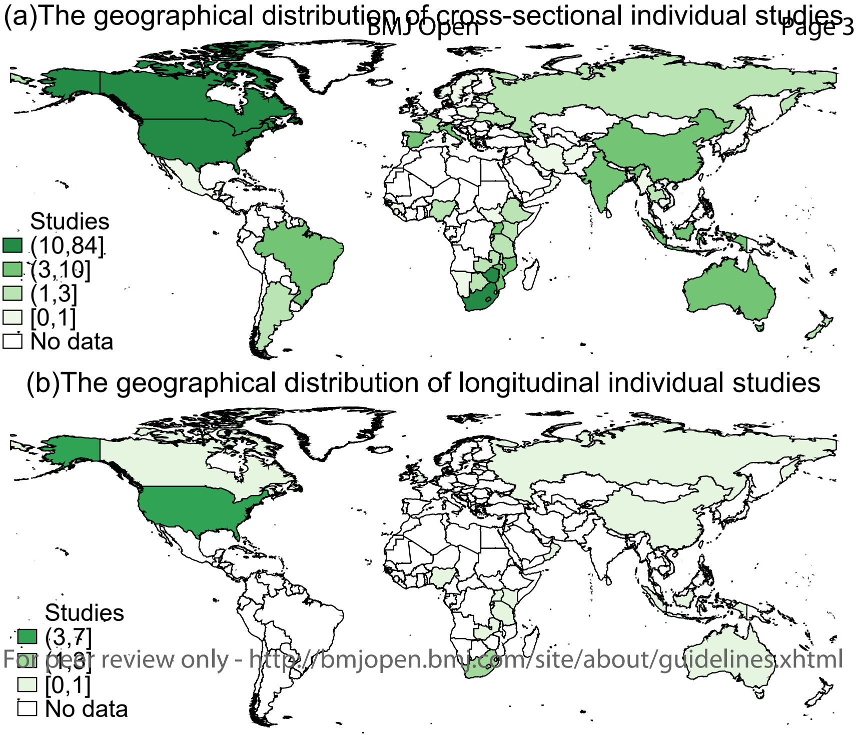
(a)The geographical distribution of cross-sectional individual studies
Studies
(10,84]
(3,10]
(1,3]
[0,1]
No data
(b)The geographical distribution of longitudinal individual studies
Studies
(3,7]
[0,1]
No data

Supplementary material

Table S1: Database search queries

Database	Query number	Query	Number of articles
PubMed (Searched on 05-04-2022)	1	((HIV) AND ((Care cascade) OR (cascade of care) OR (UNAIDS 90-90-90) OR (continuum of care[Title/Abstract]))) **OR** (((HIV) AND ((Care cascade) OR (UNAIDS 90-90-90) OR (cascade of care) OR (continuum of care[Title/Abstract]))) AND ((Multistate models[Title/Abstract]) OR (longitudinal[Title/Abstract]) OR (Markov models[Title/Abstract]) OR (Multi-state models[Title/Abstract])))	1,608
CINAHL Complete (04-07-2022)	S1	MH human immunodeficiency virus or hiv	32,691
	S2	MH hiv infection	945
	S3	TI (HIV infection or Human immunodeficiency virus or HIV) OR AB (HIV infection or Human immunodeficiency virus or HIV)	104,219
	S4	S1 OR S2 OR S3	106,982
	S5	MH care cascade	1,284
	S6	MH continuum of care	7,104
	S7	MH 90-90-90	17,568
	S8	TI (care cascade or cascade of care or continuum of care or "care cascade progress" or UNAIDS 90-90-90 progress" or UNAIDS 90-90-90 or "cross-sectional care cascade") OR AB (care cascade or cascade of care or continuum of care or "care cascade progress" or UNAIDS 90-90-90 progress" or UNAIDS 90-90-90 or "cross-sectional care cascade")	5,497
	S9	S5 OR S6 OR S7 OR S8	6,288
	S10	S4 AND S9	916
	S11	MH Multistate models	293
	S12	MH Multi-state models	1,763
	S13	MH Markov models	2,000
	S14	TI (Multistate models or Multi-state models or Markov models or "longitudinal care cascade") OR AB (Multistate models or Multi-state models or Markov models or "longitudinal care cascade")	3,925
	S15	S11 OR S12 OR S13 OR S14	3,992
	S16	S10 AND S15	5
	S17	S10 OR S16	916
EMBASE (Search date 15-07-2022)	#1	'human immunodeficiency virus'/exp OR 'human immunodeficiency virus infection'/exp OR 'human immunodeficiency virus':ti,ab,kw OR 'human immunodeficiency virus infection':ti,ab,kw OR hiv:ti,ab,kw OR 'hiv infection':ti,ab,kw	601495

For peer review only - http://bmjopen.bmj.com/site/about/guidelines.xhtml

	#2	'cascade of care'/exp OR 'continuum of care'/exp OR 'care continuum':ti,ab,kw OR '90 90 90':ti,ab,kw OR 'care cascade':ti,ab,kw OR 'cascade of care':ti,ab,kw OR 'continuum of care':ti,ab,kw OR 'care cascade progress':ti,ab,kw OR 'unaids 90-90-90 progress':ti,ab,kw OR 'unaids 90-90-90':ti,ab,kw OR 'cross-sectional care cascade':ti,ab,kw	9220
	#3	#1 AND #2	3145
	#4	'multistate model'/exp OR 'multistate model' OR 'multi state model'/exp OR 'multi state model' OR 'markov chain'/exp OR 'markov chain' OR 'multistate models':ti,ab,kw OR 'multi state models':ti,ab,kw OR 'markov models':ti,ab,kw OR 'longitudinal':ti,ab,kw	429711
	#5	#3 AND #4	171
	#6	#3 OR #5	3145
Other strategic searches	PubMed and Google scholar search using individual key works such as "HIV care cascade" or "HIV care cascade and multistate models" or "HIV care cascade and multi-state models" or "HIV care cascade and Markov models"		

For peer review only - http://bmjopen.bmj.com/site/about/guidelines.xhtml

Table S2: Data extraction tool for the scoping review

Information extracted from articles	Possible responses
Paper ID number	
Authors	
Study site in terms of country	
The specific study site of the research article	
The level of study representation	
The study design	Cross-sectional, Case-Control, Longitudinal, Cohort, Prospective cohort, Retrospective cohort, Clinical trial, Not stated in the article, Other
Other study design not listed above	
Data sources for the studies reviewed	Cohort data, Laboratory test data (including CD4 and viral load tests), Medical records from the hospital, Survey data, Simulated data (mathematical modeling), Surveillances, Surveillances, Others
Specify other data sources not listed in the list above	
Number of patients/participants included in the final analysis of the study	
The actual age of participants included in the study	
Categories of the age of participants included in the study	All (children and adults) PLHIV, Adults (≥18 years), Adults (≥15 years), Adults (≥16 years), Adolescents and adults (>=13 years), Adolescents and adults (>=12 years), Adolescents (10-19 years), Neonates
Type of participants included in a research article	All (mixture) PLHIV, Children only, Adolescents, Adults, Key populations, Migrants, Prisoners, Veterans, Refugees, Military
Key population, please specify	Men having sex with men, Female sex workers, Injected drug users, Transgender women, Transgender men
Cascade size	Full cascade stages, More than full cascade (> 6 stages), Medium cascade stages, Small to very small cascade stages, Other
Specify other study outcomes	
Number of the cascade stages	
Actual stage name	
stage 1	
stage 2	
stage 3	
stage 4	
stage 5	
stage 6	

For peer review only - http://bmjopen.bmj.com/site/about/guidelines.xhtml

**Information extracted from articles**	**Possible responses**
stage 7	
stage 8	
stage 9	
Absorbing state	Death, LTFU, Transfer, Other
Specify other absorbing states	
The type of multistate model used	Non-Parametric multistate model, Parametric, continuous-time multistate Markov model, Time-homogeneous Markov models, Discrete-time multistate Markov model, General multistate model, Other
Specify other types of multistate models used	
Multistate model assumptions	
Assessment starting point (baseline)	Enrolment/recruitment date, ART start date
Reason for censoring	
Descriptions of statistics used	Prevalence, Incidence rates, Proportion for each stage of HIV care cascade, Chi-square test of independence, Fisher Exact test (small sample size), t-test, Wilcoxon rank test, Time spent in each cascade stage, Transition probabilities, Mean, Standard deviation, Median, Interquartile range
Type of regression models used	Logistics regression, Cox PH regression, Poisson regression, competing risk regression, Generalized linear regression model (GLM), Weighted linear regression, Mathematical back-calculation, Log-Binomial regression, Generalized linear mixed models (GLMM), Multinomial regression model, Other
Other regression models, specify	
The method used for handling missing data	Weighted analysis, Multiple imputations, Missing data indicator, Not addressed
Methods used for assessing the goodness of fit of the model	Pearson-type Chi-Square test, Visual comparison of observed and fitted value plots, Not assessed, Other
Other methods for assessing the goodness of fit of the model	
Broad categories of approaches used in research articles	Cross-sectional, Longitudinal
Specification of the cascade staging method	Yes, no
Cascade staging method	Dependent, independent, and both dependent and independent

For peer review only - http://bmjopen.bmj.com/site/about/guidelines.xhtml

Table S3: The reported study design and data analysis methods used to assess the HIV cascade and continuum of care among longitudinal studies (n=23)

Reported study design	Data analysis methods used	Number of articles
Longitudinal	Survival analysis: Competing risk methods	6
Longitudinal	Repeated cross-sectional	1
Longitudinal	Survival analysis	2
Longitudinal	Survival analysis and multistate models	6
Longitudinal	Cross-sectional analysis	5
Longitudinal	Repeated cross-sectional and time spent in the cascade stage	1
Longitudinal	Repeated measure analysis	2

For peer review only

For peer review only - http://bmjopen.bmj.com/site/about/guidelines.xhtml

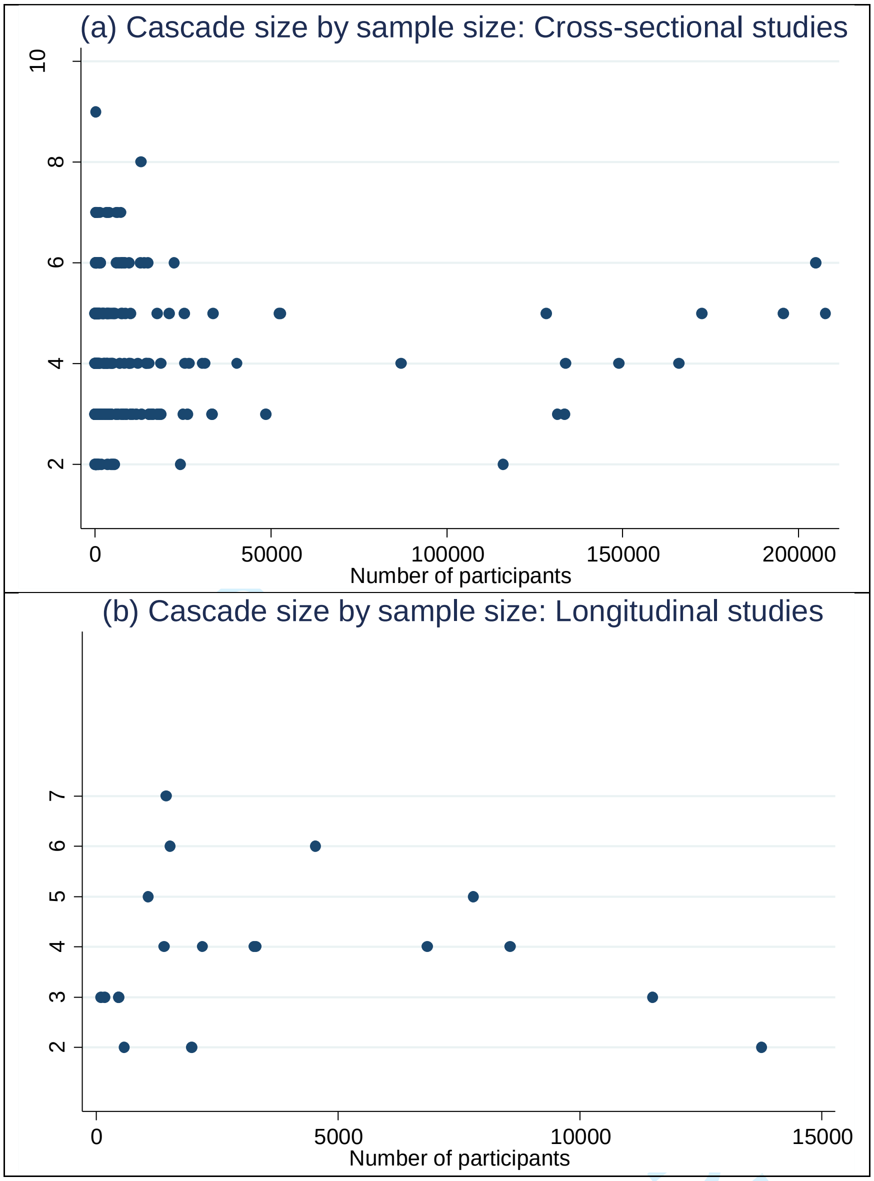

Figure S1: The distribution of cascade size by study number of participants included, in panel (a) sample size was truncated at 30,0000 (excluded 14 studies), and in panel (b) sample size was truncated at 20,000 (excluded 4 studies) to allow better visualization of the distribution

For peer review only - http://bmjopen.bmj.com/site/about/guidelines.xhtml

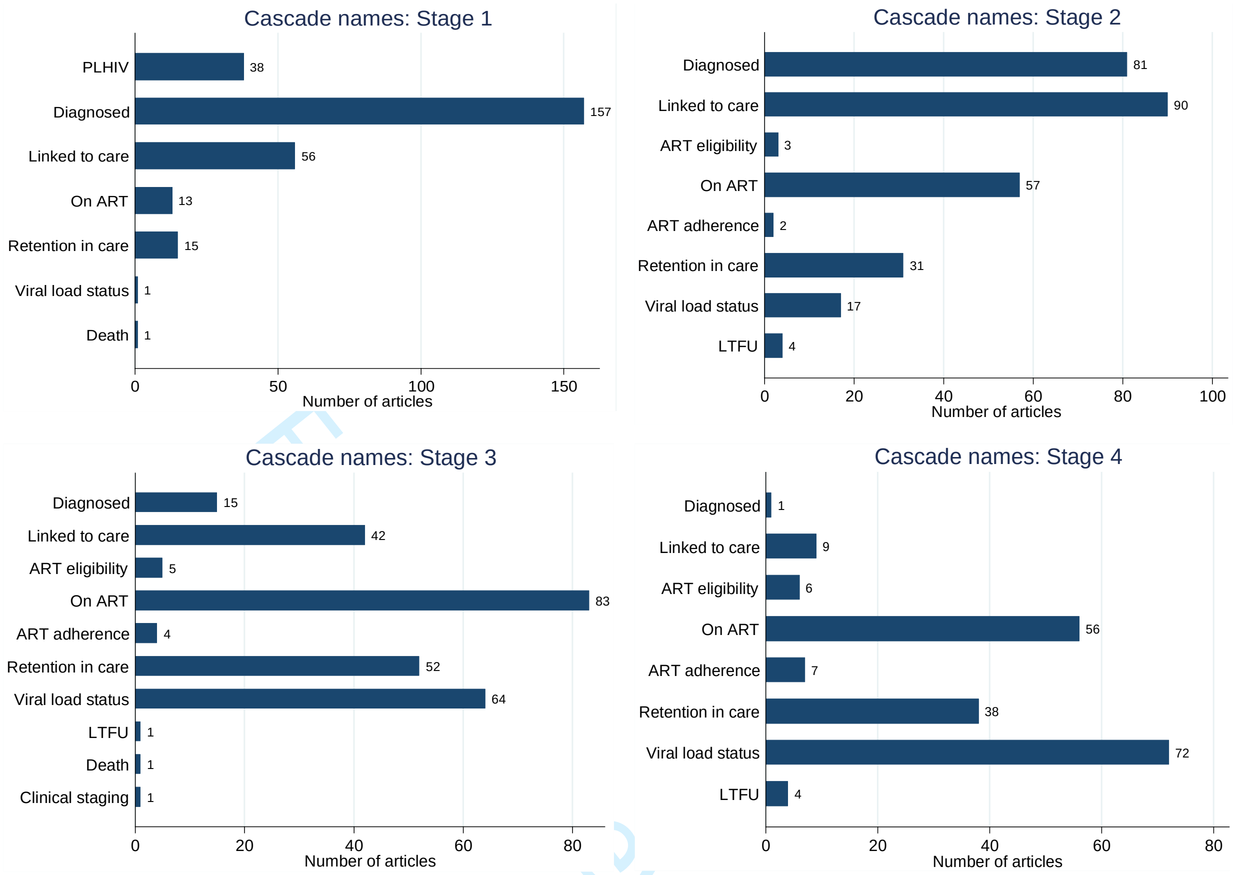

Figure S2: HIV care cascade names for stages 1–4 of articles used a cross-sectional study design

For peer review only - http://bmjopen.bmj.com/site/about/guidelines.xhtml

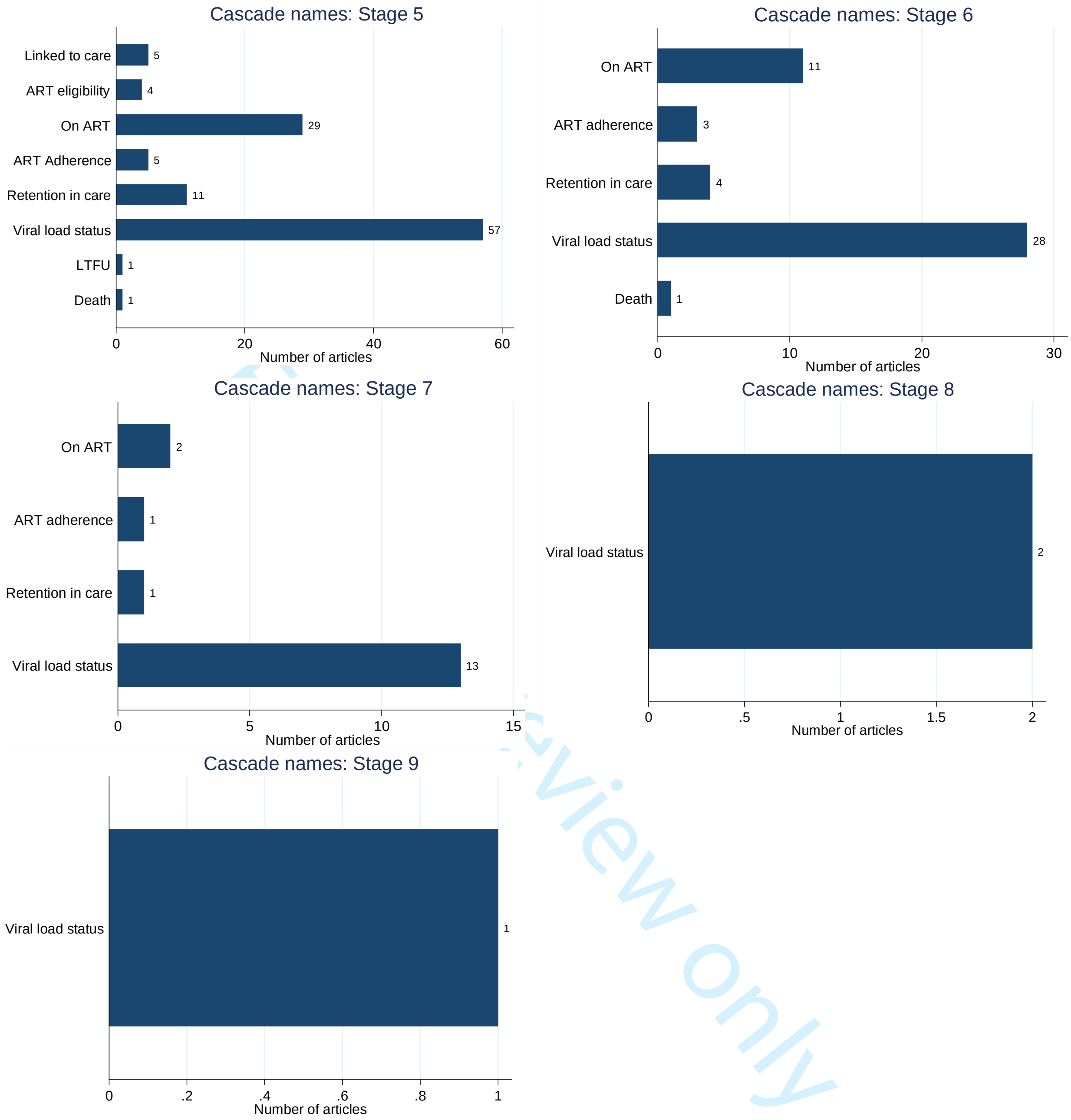

Figure S3: HIV care cascade names for stages 5–9 of articles using a cross-sectional study design

For peer review only - http://bmjopen.bmj.com/site/about/guidelines.xhtml

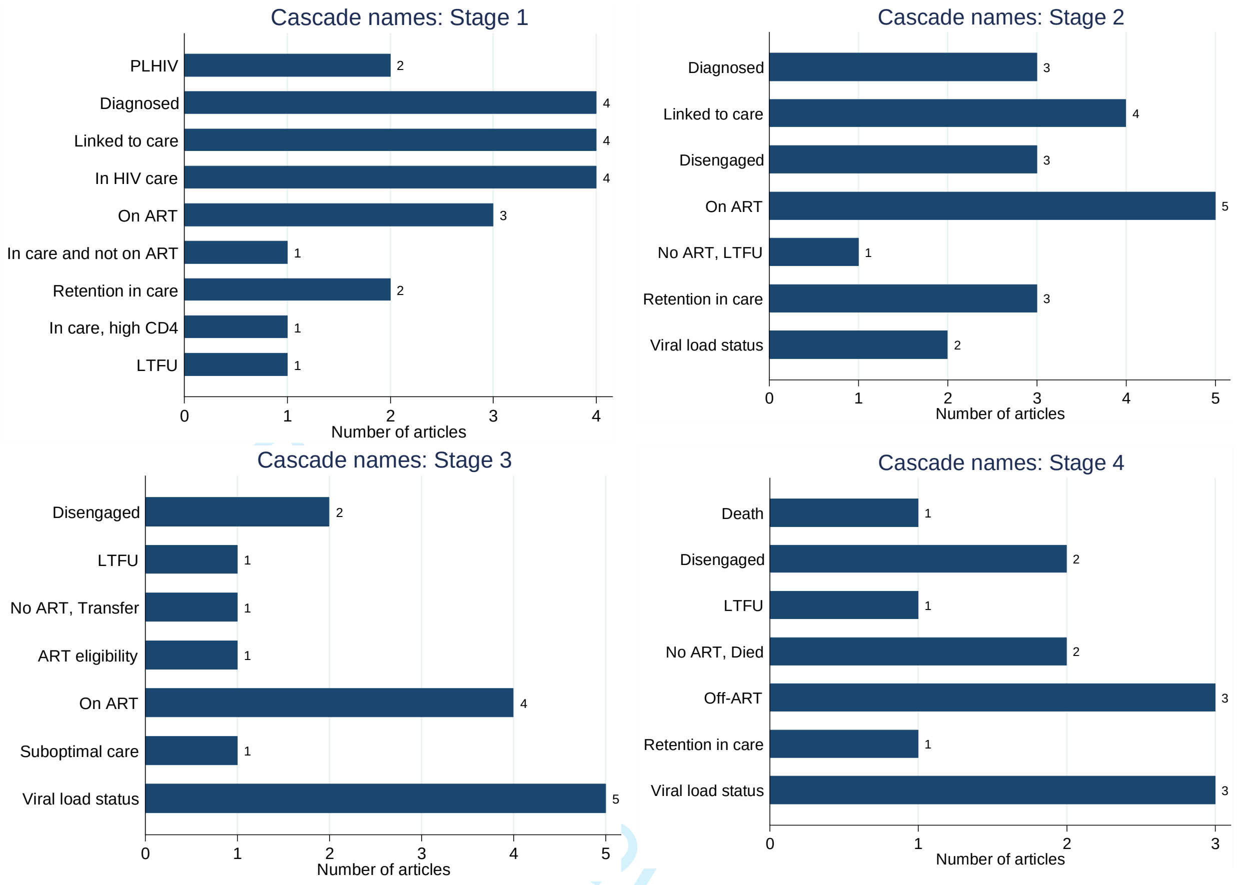

Figure S4: HIV care cascade stage names for stages 1–4 of articles using a longitudinal study design

For peer review only - http://bmjopen.bmj.com/site/about/guidelines.xhtml

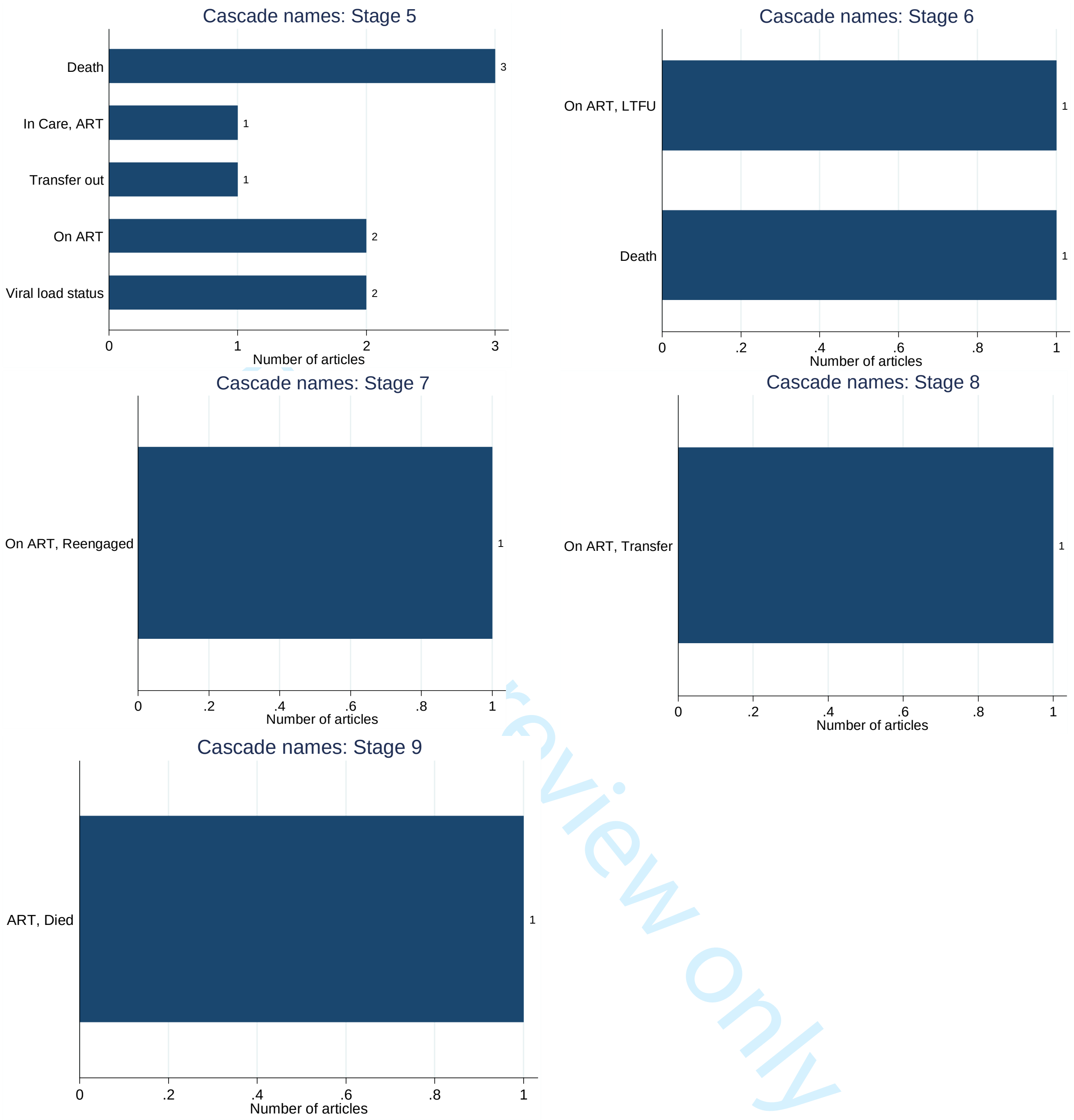

Figure S5: HIV care cascade stage names for stages 5–9 of articles using a longitudinal study design

For peer review only - http://bmjopen.bmj.com/site/about/guidelines.xhtml

(a) Mody et al 2020

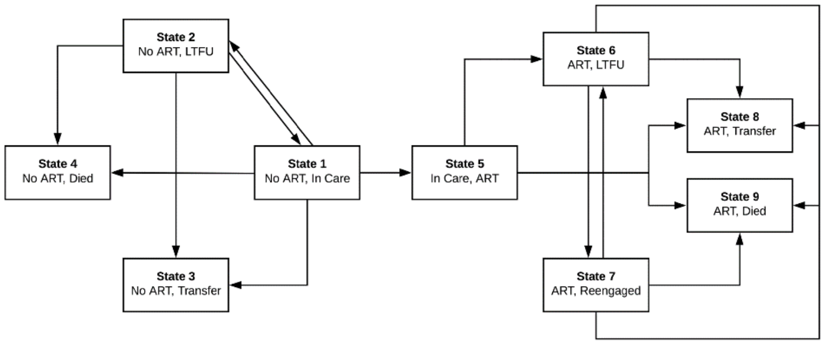

(b) Rahmalia et al 2019

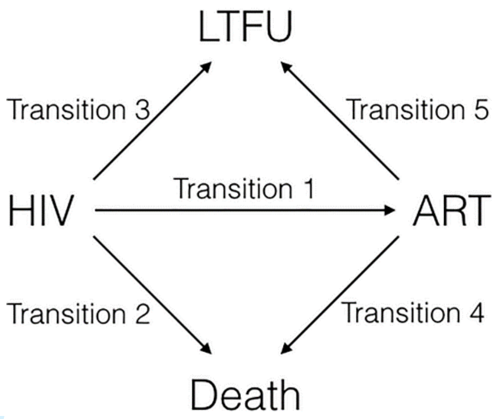

(c) Lee et al 2017

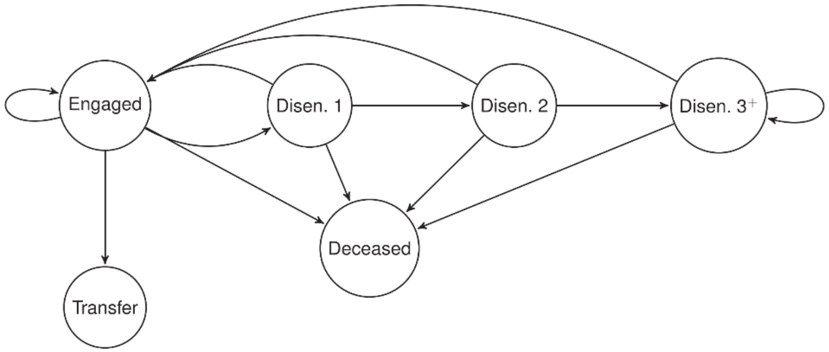

(d) Lee et al 2018

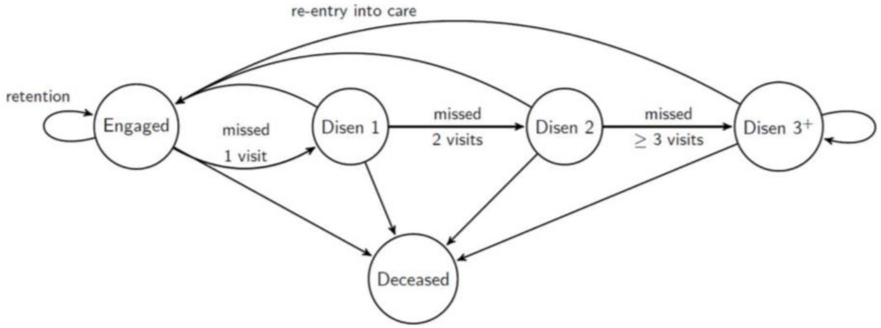

(E) Wang et al 2019

For peer review only - http://bmjopen.bmj.com/site/about/guidelines.xhtml

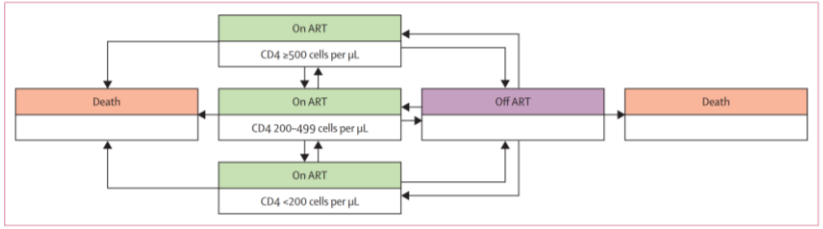

Figure S6: Multistate frameworks of included articles

For peer review only

For peer review only - http://bmjopen.bmj.com/site/about/guidelines.xhtml

Preferred Reporting Items for Systematic reviews and Meta-Analyses extension for Scoping Reviews (PRISMA-ScR) Checklist

SECTION	ITEM	PRISMA-ScR CHECKLIST ITEM	REPORTED ON PAGE #
**TITLE**			
Title	1	Identify the report as a scoping review.	1
**ABSTRACT**			
Structured summary	2	Provide a structured summary that includes (as applicable): background, objectives, eligibility criteria, sources of evidence, charting methods, results, and conclusions that relate to the review questions and objectives.	2
**INTRODUCTION**			
Rationale	3	Describe the rationale for the review in the context of what is already known. Explain why the review questions/objectives lend themselves to a scoping review approach.	5
Objectives	4	Provide an explicit statement of the questions and objectives being addressed with reference to their key elements (e.g., population or participants, concepts, and context) or other relevant key elements used to conceptualize the review questions and/or objectives.	6
**METHODS**			
Protocol and registration	5	Indicate whether a review protocol exists; state if and where it can be accessed (e.g., a Web address); and if available, provide registration information, including the registration number.	6
Eligibility criteria	6	Specify characteristics of the sources of evidence used as eligibility criteria (e.g., years considered, language, and publication status), and provide a rationale.	7
Information sources*	7	Describe all information sources in the search (e.g., databases with dates of coverage and contact with authors to identify additional sources), as well as the date the most recent search was executed.	7
Search	8	Present the full electronic search strategy for at least 1 database, including any limits used, such that it could be repeated.	7 and Supplementary materials Page 1. Table S1
Selection of sources of evidence†	9	State the process for selecting sources of evidence (i.e., screening and eligibility) included in the scoping review.	7-8
Data charting process‡	10	Describe the methods of charting data from the included sources of evidence (e.g., calibrated forms or forms that have been tested by the team before their use, and whether data charting was done independently or in duplicate) and any processes for obtaining and confirming data from investigators.	8 and Table S2
Data items	11	List and define all variables for which data were sought and any assumptions and simplifications made.	8-10
Critical appraisal of individual sources of evidence§	12	If done, provide a rationale for conducting a critical appraisal of included sources of evidence; describe the methods used and how this information was used in any data synthesis (if appropriate).	Not applicable
Synthesis of results	13	Describe the methods of handling and summarizing the data that were charted.	10-11

St. Michael's
Inspired Care.
Inspiring Science.

For peer review only - http://bmjopen.bmj.com/site/about/guidelines.xhtml

SECTION	ITEM	PRISMA-ScR CHECKLIST ITEM	REPORTED ON PAGE #
**RESULTS**			
Selection of sources of evidence	14	Give numbers of sources of evidence screened, assessed for eligibility, and included in the review, with reasons for exclusions at each stage, ideally using a flow diagram.	11 and Figure 1
Characteristics of sources of evidence	15	For each source of evidence, present characteristics for which data were charted and provide the citations.	11-15
Critical appraisal within sources of evidence	16	If done, present data on critical appraisal of included sources of evidence (see item 12).	Not applicable
Results of individual sources of evidence	17	For each included source of evidence, present the relevant data that were charted that relate to the review questions and objectives.	15-18
Synthesis of results	18	Summarize and/or present the charting results as they relate to the review questions and objectives.	11-18
**DISCUSSION**			
Summary of evidence	19	Summarize the main results (including an overview of concepts, themes, and types of evidence available), link to the review questions and objectives, and consider the relevance to key groups.	18-20
Limitations	20	Discuss the limitations of the scoping review process.	4
Conclusions	21	Provide a general interpretation of the results with respect to the review questions and objectives, as well as potential implications and/or next steps.	20
**FUNDING**			
Funding	22	Describe sources of funding for the included sources of evidence, as well as sources of funding for the scoping review. Describe the role of the funders of the scoping review.	21 and 22

JBI = Joanna Briggs Institute; PRISMA-ScR = Preferred Reporting Items for Systematic reviews and Meta-Analyses extension for Scoping Reviews.

* Where *sources of evidence* (see second footnote) are compiled from, such as bibliographic databases, social media platforms, and Web sites.

† A more inclusive/heterogeneous term used to account for the different types of evidence or data sources (e.g., quantitative and/or qualitative research, expert opinion, and policy documents) that may be eligible in a scoping review as opposed to only studies. This is not to be confused with *information sources* (see first footnote).

‡ The frameworks by Arksey and O'Malley (6) and Levac and colleagues (7) and the JBI guidance (4, 5) refer to the process of data extraction in a scoping review as data charting.

§ The process of systematically examining research evidence to assess its validity, results, and relevance before using it to inform a decision. This term is used for items 12 and 19 instead of "risk of bias" (which is more applicable to systematic reviews of interventions) to include and acknowledge the various sources of evidence that may be used in a scoping review (e.g., quantitative and/or qualitative research, expert opinion, and policy document).

From: Tricco AC, Lillie E, Zarin W, O'Brien KK, Colquhoun H, Levac D, et al. PRISMA Extension for Scoping Reviews (PRISMAScR): Checklist and Explanation. Ann Intern Med. 2018;169:467–473. doi: 10.7326/M18-0850.

For peer review only

ResearchGate

See discussions, stats, and author profiles for this publication at: https://www.researchgate.net/publication/319713049

2017 Guidance for the Conduct of JBI Scoping Reviews

Chapter · September 2017

CITATIONS
29

READS
34,092

6 authors, including:

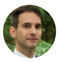
Micah Peters
University of South Australia
107 PUBLICATIONS **9,825** CITATIONS
SEE PROFILE

Christina M Godfrey
Queen's University
67 PUBLICATIONS **8,557** CITATIONS
SEE PROFILE

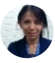
Hanan Khalil
La Trobe University
97 PUBLICATIONS **4,104** CITATIONS
SEE PROFILE

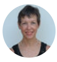
Patricia Mcinerney
University of the Witwatersrand
66 PUBLICATIONS **3,526** CITATIONS
SEE PROFILE

Some of the authors of this publication are also working on these related projects:

Project Guidance for the Conduct and Reporting of Scoping Reviews View project

Project Education in Collective Health View project

All content following this page was uploaded by Micah Peters on 21 September 2017.

The user has requested enhancement of the downloaded file.

Chapter 11: Scoping Reviews

For peer review only

Preamble:

The Joanna Briggs Institute Reviewer's Manual^ provides guidance to authors for the conduct and preparation of JBI systematic reviews and evidence syntheses. The JBI Reviewer's Manual has separate chapters devoted synthesis of different types of evidence and to address different types of review questions.

This document is contains Chapter 11: Scoping Reviews from the 4th Edition of the JBI Reviewer's Manual, and is up to date as of September 21, 2017. This Chapter includes changes that correspond to the latest methodological developments determined by the JBI Methodology Groups and JBI Scientific Committee, the latest developments with the JBI SUMARI software (https://www.jbisumari.org/) and also feedback by users.

The online chapter can be accessed at:

https://reviewersmanual.joannabriggs.org/display/MANUAL/Chapter+11%3A+Scoping+reviews

The open-access published journal article can be cited as:

Peters MDJ, Godfrey CM, McInerney, Khalil H, Parker D, and Baldini Soares C. Guidance for conducting systematic scoping reviews. Int J Evid Based Healthc. 2015. 13(3):141-146. Available: http://www.ncbi.nlm.nih.gov/pubmed/26134548

^Aromataris E, Munn Z (Editors). *Joanna Briggs Institute Reviewer's Manual.* The Joanna Briggs Institute, 2017. Available from https://reviewersmanual.joannabriggs.org/

For peer review only

For peer review only

For peer review only - http://bmjopen.bmj.com/site/about/guidelines.xhtml

Contents

0: Contributors and how to cite... 5
1: Scoping reviews and evidence-based practice 6
1.1 Why a scoping review? 6
1.2 JBI scoping reviews 7
1.3 The scoping review framework 9
2: Development of a JBI scoping review protocol 10
2.1 Author information 10
2.2 Developing the title, objective, and question 10
2.3 Background 11
2.4 Inclusion criteria 12
2.5 Search strategy 13
2.6 Extraction of the results. 14
2.7 Presentation of the results 15
3: The scoping review and summary of the evidence 16
3.1 Title of the scoping review 16
3.2 Review authors 16
3.3 Executive summary 16
3.4 Main body of the report. 18
3.5 Method of the report 19
3.6 Presenting the results. 20
3.7 Discussion, Conclusion, and Implications for research and practice 22
3.8 End matter 22
3.9 References 22
4: Appendices 23
4.1 Appendix I: Template study details and characteristics and results extraction instrument 23
References 24

For peer review only

For peer review only - http://bmjopen.bmj.com/site/about/guidelines.xhtml

Authors:

Micah D J Peters

The Joanna Briggs Institute, Faculty of Health Sciences, The University of Adelaide, Australia

Christina M Godfrey

Queen's Joanna Briggs Collaboration, Queen's University, Kingston, Canada

Patricia McInerney

The Wits - JBI Centre for Evidence-based practice, Faculty of Health Sciences, University of the Witwatersrand, South Africa

Cassia Baldini Soares

The Brazilian Centre for Evidence-based Healthcare, School of Nursing, University of São Paulo, Brazil

Hanan Khalil

Monash University, School of Rural Health, The Centre for Chronic Diseases Management, Australia

Deborah Parker

The University of Queensland, Australian Centre for Evidence Based Community Care

How to Cite the Online Manual:

Peters MDJ, Godfrey C, McInerney P, Baldini Soares C, Khalil H, Parker D. Chapter 11: Scoping Reviews. In: Aromataris E, Munn Z (Editors). Joanna Briggs Institute Reviewer's Manual. The Joanna Briggs Institute, 2017. Available from https://reviewersmanual.joannabriggs.org/

For peer review only

For peer review only - http://bmjopen.bmj.com/site/about/guidelines.xhtml

1: Scoping reviews and evidence-based practice

Evidence-based practice is an expanding field and together with a rapid increase in the availability of primary research, the conduct of reviews has also escalated. Different forms of evidence and different kinds of review objectives and questions call for the development of new approaches that are designed to more effectively and rigorously synthesize the evidence. In 2009 Grant and Booth identified 14 different types of reviews (Grant and Booth, 2009). Scoping reviews, also called “mapping reviews” or “scoping studies” are one of these (Ehrich et al. 2002; Anderson et al. 2008). In 2005 Arksey and O’Malley proposed a framework for conducting them (Arksey and O’Malley, 2005). The Arksey and O’Malley framework was advanced and extended in 2010 by Levac, Colquhoun and O’Brien (2010) and then further refined and corresponding guidance developed by the present authors (Peters et al. 2015).

1.1 Why a scoping review?

There are a number of reasons why a scoping review might be conducted. Unlike other reviews that address relatively precise questions, such as a systematic review of the effectiveness of a particular intervention based on a precise set of outcomes, scoping reviews can be used to map the key concepts underpinning a research area as well as to clarify working definitions, and/or the conceptual boundaries of a topic (Arksey and O’Malley, 2005). A scoping review may focus on one of these aims or all of them as a set.

Scoping reviews can usefully map evidence in a number of ways (Anderson et al. 2008). Scoping reviews undertaken with the objective of providing a map of the range of the available evidence can be undertaken as a preliminary exercise prior to the conduct of a systematic review. Scoping reviews are useful for examining emerging evidence when it is still unclear what other, more specific questions can be posed and valuably addressed. For example, while there are few studies on the sustainability of knowledge translation interventions in the area of chronic disease management, a scoping review has provided the foundation for a future systematic review to investigate the impact of sustainable knowledge translation interventions on health outcomes (Tricco et al. 2016a).

Beyond underpinning future systematic review, scoping reviews can also inform clinical decision making and practice. For example, a scoping review might seek to map the types and details of tools used to assess quality of life following tonsillectomy (Kao et al. 2017a).. In this case, the scoping review could potentially be used both to provide direction for future systematic reviews as well as having value to knowledge users by providing a comprehensive evidence base to aid in the selection of quality of life tools for use in clinical practice.

Scoping reviews can be conducted to examine and clarify broad areas to identify gaps in the evidence, clarify key concepts, and report on the types of evidence that address and inform practice in a topic area. Scoping reviews can be used to map evidence in relation to time (when it was published), location (country), source (peer reviewed or grey literature), approach (how it was studied/researched), and/or origin (healthcare or academic discipline) (Anderson et al. 2008).

Davis, Drey and Gould (2009) explain how, as useful tools for evidence reconnaissance, scoping reviews can be used to provide a broad overview of a topic. For instance, a scoping review that seeks to develop a “concept map” may aim to explore how, by whom and for what

For peer review only - http://bmjopen.bmj.com/site/about/guidelines.xhtml

purpose a particular term is used in a given field (Anderson et al. 2008). Similarly, scoping reviews can be conducted in order to establish a comprehensive understanding of how scoping reviews themselves have been conducted and reported (Pham et al, 2014; Tricco et al. 2016b). Scoping review methodology was used to identify papers and guidelines that had either utilized or described scoping review methods and/or assessed the quality of reporting for scoping reviews (Tricco et al. 2016b). This review showed how the number of scoping reviews had steadily increased since 2012, that there was variation in terms of how they were conducted and reported, and that standardized reporting guidelines were absent.

Scoping reviews may also be used to develop "policy maps" by identifying and mapping evidence from policy documents and reports that guide practice in a particular field (Anderson et al. 2008). For example, a scoping review might include the objective of mapping research papers and policy documents that concern models of transition for young people to adult health services to provide evidence for best practice transitional care for children with complex health needs (Watson et al. 2011).

For peer review only

For peer review only - http://bmjopen.bmj.com/site/about/guidelines.xhtml

For peer review only

The value of scoping reviews to evidence-based practice is the examination of a broader area to identify gaps in the research knowledge base (Crilly, Jashapara and Ferlie, 2010), clarify key concepts (de Chavez et al. 2005), and report on the types of evidence that address and inform practice in the field (Decaria, Sharp and Petrella, 2012).

1.2 JBI scoping reviews

The synthesis of evidence in the form of the systematic review is at the center of evidence-based practice (Pearson et al. 2005). Systematic reviews traditionally bring together evidence from quantitative literature to answer questions on the effectiveness of a specific intervention for a particular condition. Beyond effectiveness, the Joanna Briggs Institute (JBI) is also interested in the context of care delivery, its cost-effectiveness, as well as patient, carer and healthcare provider preferences. These foci are explored in terms of the appropriateness, meaningfulness, and feasibility of healthcare practices and delivery. These sorts of questions are most commonly answered by consideration of other forms of primary evidence found in qualitative and economic research. The results of well-designed research studies of any methodology are regarded by the JBI as potential sources of credible evidence. To match this broader and more inclusive view of evidence, the Institute has developed a number of methodologies and methods for the synthesis of evidence to support healthcare decision-making.

All JBI systematic reviews – including scoping reviews – begin with the development of an *a-priori* protocol with inclusion and exclusion criteria that relate clearly to the objective/s and review question/s. A typical systematic review aims to answer a specific question (or series of questions) based on very precise inclusion criteria, for example, a systematic review may pose the following precise question based upon the PICO (Population, Intervention, Comparator, and Outcome) elements of its inclusion criteria:

> "What is the effectiveness of the Gardasil vaccine compared with the Cervarix vaccine in preventing Human Papillomavirus infection in adolescent and young adult women?"

It is clear from this question that only certain types of quantitative evidence and data would be relevant and that the review will be very specific in terms of the population, intervention, comparator, and kinds of outcomes against which it will measure effectiveness.

A scoping review will have a broader "scope" with correspondingly less restrictive inclusion criteria. The following question based upon the PCC (Population, Concept and Context) elements of the inclusion criteria may be posed:

> "What types of neurological reactions to the Human Papillomavirus vaccination have been reported?"

This question leaves the population rather "open" and implies that both men and women of any age will be suitable for inclusion as long as they have received a HPV vaccination. The intervention in this example is also 'open' to any kind of HPV vaccine and does not stipulate that there will be any kind of measurement of outcomes or comparison involved. The "concept" of this scoping review (neurological reactions) is also broad, and could cover any kind of neurological outcome as long as it is a reaction to HPV vaccination. For this particular question, the 'context' has also been left open, so the evidence may come from any context (e.g. geographical, healthcare setting, sociocultural).

For peer review only - http://bmjopen.bmj.com/site/about/guidelines.xhtml

For peer review only

An especially important point is that the scoping review question may draw upon data from any type of evidence and research methodology, and is not restricted to quantitative studies (or any other study design) alone. This however is not prescriptive; reviewers may decide that particular study designs would be beyond the scope of their scoping review, or not be appropriate or useful for consideration. For example, in the protocol, this scoping review example may specify that text and opinion literature will not be included. Because of the broad nature of scoping reviews, they are particularly useful for bringing together evidence from disparate or heterogeneous sources. In the example scoping review question regarding HPV vaccination above, reports of neurological side effects such as syncope (fainting) from randomized controlled trials can be considered side by side with qualitative accounts of patients' experiences of paralysis following HPV vaccination.

It is important to highlight the distinction between scoping reviews and "mixed methods" systematic reviews that also rely on evidence from a number of different study designs (Pearson et al. 2015). While in a scoping review the goal is to determine what kind of evidence (quantitative and/or qualitative) is available on the topic and to represent this evidence by mapping or charting the data, mixed methods systematic reviews are designed to answer a question or questions based on the synthesis of evidence from for example qualitative, quantitative and economic research (Reilly et al. 2016). For example:

> "What is the effectiveness, cost effectiveness, acceptability and implementation barriers/enablers for chronic kidney disease management programs for Aboriginal and Torres Strait Islander Australians?"

The goal of this mixed methods systematic review was to: i) report on a the effectiveness of chronic kidney disease management programs from quantitative evidence of effectiveness, ii) report on the relative cost-effectives of chronic kidney disease management programs from economic evidence, and to iii) examine the experiences of Aboriginal and Torres Strait Islander Australians and healthcare providers regarding chronic kidney disease management programs in terms of acceptability as well as barriers and enablers of implementation.. Following separate methodological quality assessment, data extraction and synthesis, the results of each synthesis were then brought together in a comprehensive synthesis using evidence from each research type to answer a specific question. In this example, the knowledge gained from the qualitative and economic evidence can be used to enhance the knowledge gained from the quantitative evidence.

Another important distinction between scoping reviews and systematic reviews is that, unlike systematic reviews, scoping reviews provide an overview of the existing evidence, regardless of quality. This is because scoping reviews aim to provide a map of what evidence has been produced as opposed to seeking only the best available evidence to answer more specific questions related to policy and practice. Hence, unless otherwise specified, a formal assessment of methodological quality of the included studies of a scoping review is generally not performed or congruent with the purpose of scoping reviews.

While implications for research, including for primary research, other scoping reviews, or systematic reviews, may be generated from the results of a scoping review – especially those conducted with the objective of being precursors to systematic reviews, implications for practice are limited by the fact that a formal assessment of methodological quality of the included studies of a scoping review is generally not performed. If implications for practice are

For peer review only - http://bmjopen.bmj.com/site/about/guidelines.xhtml

developed, it is expected that they will clearly flow from the objectives of the scoping review.

For peer review only - http://bmjopen.bmj.com/site/about/guidelines.xhtml

1.3 The scoping review framework

The framework proposed by Arksey and O'Malley (2005) has been influential in the conduct of scoping reviews for some time. Their framework has been further enhanced by the work of Levac, Colquhoun and O'Brien (2010) (see Table 11.1). Levac and colleagues (2010) provide more explicit detail regarding what occurs at each stage of the review process and this enhancement increases both the clarity and rigor of the review process. Both of these frameworks have been drawn on in the development of the JBI approach to the conduct of scoping reviews (Peters et al. 2015).

Table 11.1: Scoping review frameworks

	Arksey and O'Malley framework (2005, p. 22-23)	Enhancements proposed by Levac, Colquhoun and O'Brien. (2010, p. 4-8)	Enhancements proposed by Peters et al (2015).
1.	Identifying the research question	Clarifying and linking the purpose and research question	Defining and aligning the objective/s and question/s
2.	Identifying relevant studies	Balancing feasibility with breadth and comprehensiveness of the scoping process	Developing and aligning the inclusion criteria with the objective/s and question/s
3.	Study selection	Using an iterative team approach to selecting studies and extracting data	Describing the planned approach to evidence searching, selection,
4.	Charting the data	Incorporating a numerical summary and qualitative thematic analysis	Searching for the evidence
5.	Collating, summarizing and reporting the results	Identifying the implications of the study findings for policy, practice or research	Selecting the evidence
6.	Consultation (optional)	Adopting consultation as a required component of scoping study methodology	Extracting the evidence
7.			Charting the evidence
8.			Summarizing the evidence in relation to the objective/s and question/s
9.			Consultation of information scientists, librarians, and/or experts (throughout)

For peer review only - http://bmjopen.bmj.com/site/about/guidelines.xhtml

2: Development of a JBI scoping review protocol

As with all systematic reviews using JBI methodologies, an *a-priori* protocol must be developed before undertaking the scoping review. A scoping review protocol is important as it pre-defines the objectives and methods of the scoping review. It is a systematic approach to the conduct and reporting of the review and allows transparency of process. This in turn allows readers to see how the results of the scoping review were arrived at. The protocol should detail the criteria that the reviewers intend on using to include and exclude studies and to identify what data is relevant, and how the data will be extracted and mapped. The protocol provides the plan for the scoping review and is important in limiting the occurrence of reporting bias. Any deviations of the scoping review report from the protocol should be clearly addressed and explained in the scoping review report. It is also recommended that all scoping reviews should contain the following sentence:

> "The objectives, inclusion criteria and methods for this scoping review were specified in advance and documented in a protocol." (citation)

The citation should be to the corresponding protocol which may be published in the JBI Database of Systematic Reviews and Implementation Reports. Reviewers should also be aware that PROSPERO (the international prospective register of systematic reviews administered by the University of York's Centre for Reviews and Dissemination) states that scoping reviews (and literature reviews) are currently ineligible for registration in the database (Centre for Reviews and Dissemination, n.d. 'inclusion criteria', para. 5) despite appearing to contain a small number of recently registered scoping reviews. In accordance with the recommendations for reporting of systematic reviews detailed in the Preferred Reporting Items for Systematic Reviews and Meta-Analyses (PRISMA) guidelines, this sentence should appear as the final line of the background/introduction section of the review report. Another point prospective scoping reviewers should be aware of is that an extension of the PRISMA statement called the PRISMA-ScR is currently under development and registered with the Enhancing the Quality and Transparency Of health Research (EQUATOR) Network (Tricco, Strauss and Moher, 2015). Led by Tricco, Strauss and Moher, this reporting checklist is being developed with the involvement of numerous international experts on scoping review methodology and evidence synthesis including authors of the JBI methodological guidance described in this chapter. The JBI approach to conducting and reporting scoping reviews described here and in Peters et al. (2015) will be congruent with the PRISMA-ScR checklist which will assist in standardizing the conduct and reporting of scoping reviews. Reviewers are therefore encouraged to use and cite the JBI methodology (Peters et al. 2015) and also indicate their compliance with the PRISMA-ScR checklist when it becomes available.

2.1 Author information

All reviews using JBI methodologies require at least two reviewers in order to minimize reporting bias. The names of all reviewers, institutional affiliations, and JBI center affiliations (if relevant), and email address for the corresponding author must be included.

2.2 Developing the title, objective, and question

Title of the scoping review protocol

The title of the protocol (and the subsequent review) should be informative and give a clear indication of the topic of the scoping review. It is recommended that the title should always include the

For peer review only - http://bmjopen.bmj.com/site/about/guidelines.xhtml

phrase "…: a scoping review" to allow easy identification of the type of document it represents. Correspondingly, protocols should also be identified as such. This is a simple example of a scoping review protocol title by Mordiffi, Peters and Ang, 2016):

"Non-invasive thermometers used in healthcare facilities: A scoping review protocol"

Scoping review titles should not be phrased as questions. For example:

"What types non-invasive thermometers have been used in healthcare facilities?"

The JBI uses a range of mnemonics for different types of review (and research) questions. It is suggested that the "PCC" mnemonic be used as a guide to construct a clear and meaningful title for a scoping review. The PCC mnemonic stands for the Population, Concept, and Context. There is no need for explicit outcomes, interventions or phenomena of interest to be stated for a scoping review; however elements of each of these may be implicit in the Concept under examination.

For peer review only

For peer review only - http://bmjopen.bmj.com/site/about/guidelines.xhtml

For peer review only

The title of the protocol (and subsequent review) should be structured to reflect the core elements of the PCC. Using the PCC mnemonic helps to construct a title that provides potential readers with important information about the focus and scope of the review, and its applicability to their needs. For example, if the review aims to map a range of devices as part of the concept (such as non-invasive thermometers) this should be stated in the title. Including the context in the title helps readers to position the review when they are searching for evidence related to their own particular information needs.

As discussed in further depth below, there should be congruence between the title, review objective/s, question/s and inclusion criteria.

Scoping review objectives

The objective of a scoping review must be clearly stated and be congruent with the title. The objective of the scoping review should indicate what the scoping review project is trying to achieve. The objective may be broad and will guide the scope of the enquiry. For the title example above, the objective has been phrased:

> "The objective of the review is to map the available evidence to provide an overview of the use of non-invasive thermometers in the general context of health care." (Mordiffi, Peters and Ang, 2016, p. 106)

The objective should also clearly underpin the question posed by the scoping review and direct the development of the specific inclusion criteria based on clearly identifiable PCC. Further considerations around the importance of clearly identifying the objective/s and review question/s for scoping reviews has been discussed elsewhere (Peters, 2016).

Scoping review questions

The scoping review question guides and directs the development of the specific inclusion criteria for the scoping review. Clarity in the review question assists in developing the protocol, facilitates effectiveness in the literature search, and provides a clear structure for the development of the scoping review report. As with the title, the question should incorporate the PCC elements. A scoping review will generally have one primary question, e.g.

> "What quality of life questionnaires are available for pediatric patients following tonsillectomies with or without adenoidectomies for chronic infections or sleep-disordered breathing?" (Kao, Peters and Ooi, 2017a, p. 1).

If that question sufficiently addresses the PCC and adequately corresponds with the objective of the review, sub questions will not be needed. However, some scoping review questions benefit from one or more sub questions that delve into particular attributes of Context, Population or Concept. Sub questions can be useful in outlining how the evidence is likely to be mapped. For example, the primary question relates to the broad population; however, the sub questions delve into potential particular issues relate to males or particular age groups of females as distinct sub populations may be relevant. Likewise, a sub question may help to justify mapping the evidence by context, e.g.

> "What nutritional screening instruments have been validated for use for the adult population in primary healthcare settings?" (Håkonsen et al. 2015, p. 92).

2.3 Background

The background section should be comprehensive and cover all the main elements of the topic

For peer review only - http://bmjopen.bmj.com/site/about/guidelines.xhtml

under review. Due to scoping reviews being essentially exploratory, it is not expected that the background covers the extant knowledge in the area under review. The reason for undertaking the scoping review should be clearly stated together with what the scoping review is intended to inform.

For peer review only

For peer review only - http://bmjopen.bmj.com/site/about/guidelines.xhtml

For publication in the JBI Database of Systematic Reviews and Implementation Reports, the suggested length for the background section of the scoping review protocol is approximately 1000 words. The background should detail any definitions important to the topic of interest. The information in the background section must also be sufficient to put the inclusion criteria in context, including an indication of whether or not there are existing scoping reviews, systematic reviews, research syntheses, and/or primary research papers available on the topic, hence supporting the rationale to conduct the scoping review. While the inclusion criteria section of the protocol (explained below) should contain clear details of each of the PCC elements, the background must provide sufficient detail in terms of the rationale for each element. Explaining for example, why only primary care settings are of interest in terms of the context of the review question above.

The background section should conclude with a statement that a preliminary search for existing scoping reviews (and ideally systematic reviews too) on the topic has been conducted. The date of the search/es and databases searched or search platforms utilized must be stated, e.g. JBI Database of Systematic Reviews and Implementation Reports, Cochrane Database of Systematic Reviews, CINAHL, PubMed, EPPI, and Epistemonikos, where relevant. If existing reviews or systematic reviews are available on the topic, a justification that specifies how the proposed review will differ from those already conducted should be detailed. This is so that readers can easily establish what new knowledge or insight the proposed review will contain in relation to existing evidence syntheses.

For publication in the JBI Database of Systematic Reviews and Implementation Reports, The Vancouver style of referencing should be used throughout the protocol with superscript numbers without brackets, used for in-text citations. A guide to the Vancouver style of referencing can be found at: http://openjournals.net/files/Ref/VANCOUVER%20Reference%20guide.pdf

2.4 Inclusion criteria

The "inclusion criteria" of the protocol details the basis on which sources will be considered for inclusion in the scoping review and should be clearly defined. These criteria provide a guide for the reader to clearly understand what is proposed by the reviewers and, more importantly, a guide for the reviewers themselves on which to base decisions about the sources to be included in the scoping review. As explained above, as for other review types, there must be clear congruence between the tile, objective/s, question/s, and inclusion criteria of a scoping review.

Types of participants

Important characteristics of participants should be detailed, including age and other qualifying criteria that make them appropriate for the objectives of the scoping review and for the review question. In the example question above regarding pediatric tonsillectomy quality of life assessment instruments, these characteristics included pediatric patients 16 years of age or younger undergoing tonsillectomy with or without adenoidectomy for chronic tonsillitis or sleep-disordered breathing.. Justification for the inclusion or exclusion of participants should be explained. Confounding population factors, e.g. co-morbidities or co-existing conditions (e.g. congenital heart defects), can also be detailed here as exclusion criteria.

Concept

For peer review only - http://bmjopen.bmj.com/site/about/guidelines.xhtml

The core concept examined by the scoping review should be clearly articulated to guide the scope and breadth of the inquiry. This may include details that pertain to elements that would be detailed in a standard systematic review, such as the “interventions” and/or “phenomena of interest” and/or “outcomes”. For example, quality of life assessment tools – which could be understood to be interventions or approaches to measuring outcomes – was part of the concept of a scoping review designed to map the types and details of existing tools . It would then be necessary to explain any relevant details pertaining to the concept that may be important for the review, for example, whether only particular tools are to be investigated (e.g. validated tools only) or whether any/all tools are eligible for inclusion.

For peer review only - http://bmjopen.bmj.com/site/about/guidelines.xhtml

Outcomes may also be a component of a scoping review's "Concept". If outcomes of interest are to be explained, they should be linked closely to the objective and purpose for undertaking the scoping review. For example, the exemplar review was also concerned with mapping the outcomes of any psychometric assessments that had been used to measure the validity, reliability, and responsiveness to change of included quality of life tools. Details of this were also a part of the "Concept" in terms of defining psychometric testing as well as the different types of validity outcomes being sought (e.g. construct and criterion).

Context

The "Context" element of a scoping review of will vary depending on the objective/s and question/s of the review. The context should be clearly defined and may include, but is not limited to, consideration of cultural factors such as geographic location and/or specific racial or gender-based interests. In some cases, context may also encompass details about the specific setting (such as acute care, primary health care or the community). Reviewers may choose to limit the context of their review to a particular country or health system or healthcare setting, depending on the topic and objectives.

In the scoping review example above, quality of life assessment tools were broadly sought from within the context of pediatric care both pre- and/or postoperatively.

Types of sources

For the purposes of a scoping review, the "source" of information can include any existing literature, e.g. primary research studies, systematic reviews, meta-analyses, letters, guidelines, websites etc. Reviewers may wish to leave the source of information "open" to allow for the inclusion of any and all sources. Otherwise, the reviewers may wish to impose limits on the types of sources they wish to include. This may be done on the basis of having some knowledge of the types of sources that would be most useful and appropriate for a particular topic. For example, the scoping review example on quality of life assessment tools sought only quantitative studies based upon the knowledge that qualitative papers focusing on peoples' experiences were unlikely to contain relevant details regarding the tools themselves or the results of psychometric testing as as such would not be particularly appropriate or useful for meeting the objectives of the review.

2.5 Search strategy

The search strategy for a scoping review should ideally aim to be as comprehensive as possible within the constraints of time and resources in order to identify both published and unpublished (grey literature) primary studies as well as reviews. Any limitations in terms of the breadth and comprehensiveness of the search strategy should be detailed and justified. As recommended in all JBI types of reviews, a three-step search strategy is to be utilized. Each step must be clearly stated in this section of the protocol. The first step is an initial limited search of at least two appropriate online databases relevant to the topic. The databases MEDLINE (PubMed) and CINAHL would be appropriate for a scoping review on quality of life assessment tools. This initial search is then followed by an analysis of the text words contained in the title and abstract of retrieved papers, and of the index terms used to describe the articles. A second search using all identified keywords and index terms should then be undertaken across all included databases. Thirdly, the reference list of identified reports and articles should be searched for

For peer review only - http://bmjopen.bmj.com/site/about/guidelines.xhtml

additional studies. This third stage may examine the reference lists of all identified studies or examine solely the reference lists of the studies that have been selected from full-text and/or included in the review. In any case, it should be clearly stated which group of studies will be examined. A statement should be included of the reviewers' intent to contact authors of primary studies or reviews for further information, if this is relevant. Finally, a full search strategy for at least one database should be included as an appendix to the protocol.

For peer review only

For peer review only - http://bmjopen.bmj.com/site/about/guidelines.xhtml

Reviewers should include the languages that will be considered for inclusion in the review as well as the timeframe, with an appropriate and clear justification for choices.

As the review question might be broad, authors may find that it is appropriate to search for all sources of evidence (e.g. primary studies and text/opinion articles) simultaneously with the one search strategy. This also depends on the relevance of the evidence sources to the topic under review and its objectives. This approach will lead to a greater sensitivity in the search, which is desirable for scoping reviews.

The search for a scoping review may be quite iterative as reviewers become more familiar with the evidence base, additional keywords and sources, and potentially useful search terms may be discovered and incorporated into the search strategy. The input of a research librarian or information scientist can be invaluable in designing and refining the search.

2.6 Extraction of the results

In scoping reviews, the data extraction process may be referred to as "charting the results". This process provides the reader with a logical and descriptive summary of the results that aligns with the objective/s and question/s of the scoping review.

A draft charting table or form should be developed at the protocol stage to record the key information of the source, such as author, reference, and results or findings relevant to the review question/s. This may be further refined at the review stage and the charting table updated accordingly. Some key information that reviewers might choose to chart are:

a. Author(s)
b. Year of publication
c. Origin/country of origin (where the study was published or conducted)
d. Aims/purpose
e. Study population and sample size (if applicable)
f. Methodology / methods
g. Intervention type, comparator and details of these (e.g. duration of the intervention) (if applicable)
h. Duration of the intervention (if applicable)
i. Outcomes and details of these (e.g. how measures) (if applicable)
j. Key findings that relate to the scoping review question/s.

A template data extraction instrument for study details, characteristics and results extraction is provided in Appendix 1 of this chapter which can be adapted by reviewers to use in their own scoping review protocols and reviews with citation to the JBI methodology guidance for scoping reviews.

For ease of reference and tracking, it is suggested that reviewers keep careful records to identity each source. As reviewers chart each study, it may become apparent that additional unforeseen data can be usefully charted. Charting the results can therefore be an iterative process whereby the charting table is continually updated. It is suggested that the review team become familiar with the source results and trial the extraction form on two or three studies to ensure all relevant results are extracted. This approach is favored by other experts on the conduct of scoping

For peer review only - http://bmjopen.bmj.com/site/about/guidelines.xhtml

reviews (Arksey and O'Malley, 2005; Armstrong et al. 2011; Valaitis et al. 2012).

For peer review only

For peer review only - http://bmjopen.bmj.com/site/about/guidelines.xhtml

2.7 Presentation of the results

At the time of protocol development, the reviewers should provide some plan for the presentation of results – for example, a draft chart or table. This would be expected to be further refined toward the end of the review when the reviewers have the greatest awareness of the contents of their included studies.

The results of a scoping review may be presented as a map of the data extracted from the included papers in a diagrammatic or tabular form, and/or in a descriptive format that aligns with the objective/s and scope of the review. The elements of the PCC inclusion criteria may be useful to guide how the data should be mapped most appropriately. In the scoping review example, because the objective was to map the types and details of quality of life assessment tools used in relation to pediatric tonsillectomy, the data may be usefully mapped by a tabular presentation of how the different tools reported in each included paper contain the same or different measurement domains, number of items, and differences in terms of the results of validity, reliability, and/or responsiveness to change testing.

The tables and charts may also show results as: distribution of studies by year or period of publication (depends on each case), countries of origin, area of intervention (clinical, policy, educational, etc.) and research methods. A narrative summary should accompany the tabulated and/or charted results and should describe how the results relate to the review objective/s and question/s.

The results can also be classified under main conceptual categories, such as: "intervention type", "study population" (and sample size, if it is the case), "duration of intervention", "aims", "methodology adopted", "key findings" (evidence established), and "gaps in the research". For each category reported, a clear explanation should be provided.

For peer review only - http://bmjopen.bmj.com/site/about/guidelines.xhtml

3: The scoping review and summary of the evidence

This section provides further guidance on the components that should comprise the final report of a scoping review and the information that each component should contain. It illustrates how each component of the review is to be managed in the scoping reviews analytical module of JBI's System for the Unified Management, Assessment and Review of Information (SUMARI) software. This section also provides a brief outline of how the scoping review should be formatted and the stylistic conventions that should be followed to ensure the review meets the criteria for publication in the JBI Database of Systematic Reviews and Implementation Reports. For further information please refer to the Author Guidelines of the journal:

http://journals.lww.com/jbisrir/Pages/informationforauthors.aspx Specifically, guidance is provided on the following components: outline of the report, inclusion criteria (i.e. PCC), search strategy, extraction, presenting and summarizing the results, and any potential implications for research and practice. All scoping reviews published in the JBI Database of Systematic Reviews and Implementation Reports must be based on a peer reviewed, scoping review protocol that has been accepted for publication in the same journal. For a traditional systematic review, while deviations from a published review protocol are rare, due to the more iterative nature of a scoping review, some changes may be necessary. These must still be clearly detailed and justified in the methods section of the scoping review report if and when they occur.

3.1 Title of the scoping review

The title should be clear, explicit and reflect the core elements of the review. Titles should not be phrased as questions or conclusions and there should be congruence between the title, review objective/question/s, and inclusion criteria. The title should include the phrase: "….: a scoping review". The title should not be more than 12-14 words for ease of understanding (see example above in Section 2).

3.2 Review authors

Affiliations for each author need to be stated, including the JBI affiliation of each reviewer (if relevant). A valid email address must be provided as contact details for the corresponding author.

3.3 Executive summary

This section is a structured abstract of the main features of the scoping review. It should be no longer than 500 words and should contain no abbreviations or references. The executive summary must accurately reflect and summarize the review for the reader, in particular the results of the review. The executive summary includes the following required headings:

3.3.1 Background

This section briefly describes the issue under review. While avoiding self-plagiarism, much of the detail in the background section of the scoping review report may be adapted from the background of the protocol.

For peer review only - http://bmjopen.bmj.com/site/about/guidelines.xhtml

3.3.2
Objective/s
and Question/s

The review objective/s and question/s should be stated in full, as described in the protocol section.

3.3.3 Inclusion criteria

Types of participants

Important characteristics of participants should be detailed, including age and other qualifying criteria that make them appropriate for the objectives of the scoping review and match the review question.

Concept

The core concept examined by the scoping review should be clearly articulated to guide the scope and breadth of the inquiry should be explained.

Context

The context should be clearly defined and explained.

Types of sources

The source of information can include any existing literature e.g. primary research studies, systematic reviews, meta-analyses, letters, guidelines, etc. should be explained.

3.3.4 Search Strategy

Details of the approach to searching as well as the sources searched should be detailed. Any limits on the search such as dates or languages should also be included.

3.3.5 Extraction of results

The methods/tools used to extract results from the included sources should be described in brief (see Appendix 11.1 for an example).

3.3.6 Presentation of results

Details of results should be described in brief as well as how they have been organized in relation to the objective and question/s of the scoping review. This should be the principle focus of the Executive Summary. Important details of the results, including the number of studies located and included. The results extracted from the literature should be clearly detailed as well as an explanation of how the data has been charted.

3.3.7 Conclusions

Brief overall conclusions based on the scoping review results should be articulated, including a clear answer to the question/s of the scoping review and how the objective was/was not met.

Implications for research

Succinctly detail the key implications for research and further need for primary research and or systematic reviews in the field.

Implications for practice

Succinctly detail the key results that can be used to inform practice. There may be significant limitations on the kinds of implications for practice able to be developed from the results of a

For peer review only - http://bmjopen.bmj.com/site/about/guidelines.xhtml

scoping review as no methodological appraisal of the quality of included studies takes place. This section may be left out if no implications for practice are made.

For peer review only

For peer review only - http://bmjopen.bmj.com/site/about/guidelines.xhtml

3.4 Main body of the scoping review

The main body of the scoping review report follows much the same structure as the protocol; there is a section for the background, objective/s and question/s, the inclusion criteria, methods, and then the presentation of the results of the scoping review, discussion of the results and conclusions. The following sections deal with each in detail.

3.4.1 Background

The background section should be comprehensive and cover all of the main elements of the topic under review, as well as appropriate information important to the review and why the topic or question of interest lends itself to a scoping review. The primary objective of the scoping review should be evident in the background as the background situates the justification and importance of the question/s posed. While many of these details will already have been addressed in the "Background" section of the protocol, reviewers may often find that the background provided with the protocol needs modification or extension following the conduct of the scoping review itself. The background section should conclude with a statement that a preliminary search for previous scoping reviews (and ideally, systematic reviews) on the topic aligning to the same concept was conducted (state the sources searched e.g. JBI Database of Systematic Reviews and Implementation Reports, The Cochrane Database of Systematic Reviews, Campbell Collection, etc.). The background section must include a citation of the original protocol and the following sentence: "The objectives, inclusion criteria and methods of analysis for this review were specified in advance and documented in a protocol." (citation)

For publication in the JBI Database of Systematic Reviews and Implementation Reports , Vancouver style referencing must be used throughout the review with superscript numbers without brackets used for in-text citations.

3.4.2 Objective/s and Question/s

The primary objective/s of the scoping review should be stated. It can be followed by specific objectives that relate to differing conceptual foci contained in the scoping review, such as, participant groups, interventions or outcome measures or a more in depth understanding of a particular phenomenon of interest or concept. (See example above in Section 2.)

The question/s posed by the scoping review should also be included in this section.

3.4.3 Inclusion criteria

This section of the scoping review specifies the basis upon which sources were considered for inclusion in the scoping review. This section should necessarily be as transparent and unambiguous as possible. The inclusion criteria for a scoping review will be contingent on the question/s asked. The PCC should be stipulated (Population, Concept, and Context).

Types of participants

The types of participants in the papers specified sought for inclusion should be related to the objectives of the scoping review. The reasons for the inclusion or exclusion of particular participants detailed in this section should be explained clearly in the background section of the scoping review report.

Concept

For peer review only - http://bmjopen.bmj.com/site/about/guidelines.xhtml

The core concept examined by the scoping review should be clearly articulated to guide the scope and breadth of the inquiry. This may include details that pertain to the "interventions" and/or "phenomena of interest" that would be explained in greater detail in a systematic review.

Outcomes may also be a component of a scoping review's "Concept". If outcomes of interest are to be explained, they should be linked closely to the objective and the purpose for undertaking the scoping review.

For peer review only

For peer review only - http://bmjopen.bmj.com/site/about/guidelines.xhtml

Context

Context will vary depending on the objective/s and question/s of the review. The context should be clearly defined and may include, but is not limited to, consideration of cultural factors, such as geographic location and/or specific racial or gender-based interests. In some cases, context may also encompass details about the specific setting (such as acute care, primary health care or the community).

Types of sources

The sources of information for the scoping review should be explained. Sources can include any existing literature, e.g. primary research studies, systematic reviews, meta-analyses, letters, guidelines, etc. The source of information may be left "open" to allow for the inclusion of any, and all sources and rationale for this should be provided. Otherwise, any limits imposed on the types of sources should be detailed and explained. For example, some sources such as text and opinion papers and letters would not be particularly appropriate or useful in order to meet the objectives of particular scoping reviews.

3.4.4 Search strategy

This section documents how the reviewers search for relevant sources of information for inclusion in the scoping review. The search strategy must be comprehensively reported and the detailed search strategy for a minimum of three major bibliographic citation databases that have been searched should be appended to the review. Ideally the individual search strategies for every database searched should be presented in sequence and in a consistent format in an appendix. Clear documentation of the search strategy is a vital component of the scientific validity of any scoping review. A scoping review should ideally consider papers (primary studies, textual papers and reviews) both published and unpublished (grey literature). The timeframe (start and end dates) chosen for the search should be clearly justified and any language restrictions specified (e.g. "only studies published in English were considered for inclusion"). Any hand searching of particular relevant journals should be detailed with the journal names and years examined. Author contact, for example, to request access to known but unavailable articles should also be included along with the outcomes of that contact.

3.4.5 Extraction of results

Extraction of results for a scoping review should include extraction of all data relevant to inform the scoping review objective/s and question/s. Charting table or forms may be used (see Appendix 11.1 for a template tool). A descriptive summary of the main results organized based on the theoretical concept underpinning the review must be included. Examples of extraction fields are identified below.

Author/year

Citation details should be consistent throughout the document. The citation details include the name of the first author (Vancouver referencing style) and year of publication.

Objective/s

A clear description of the objective of the paper should be stated.

For peer review only - http://bmjopen.bmj.com/site/about/guidelines.xhtml

For peer review only

Participants (characteristics/total number)

The defining characteristics of the participants in included sources should be provided. This includes demographic details and total numbers.

Concept

Data from included studies in relation to the concept should be extracted and mapped. The concept examined by the scoping review will vary depending on the review, and should be clearly articulated to guide the scope and breadth of the inquiry. This may include details that pertain to the "interventions" and/or "phenomena of interest" that would be explained in greater detail in a systematic review. Outcomes may also be a component of a scoping review's "Concept". If outcomes of interest are to be explained, they should be linked closely to the objective and the purpose for undertaking the scoping review.

Context

Details of the context, such as location of care (acute, primary health care, community, long term care, etc.) or a particular geographical location, should be described. Cultural, racial or gender factors may be relevant.

3.6 Presenting the results

Results

The presentation of results section should identify how many studies were identified and selected. There should be a narrative description of the search decision process accompanied by the search decision flowchart (see Figure 11.1). This flowchart has been adapted from the PRISMA flowchart developed by Moher et al. (2009), but will be likely to be replaced with a purpose designed flowchart when the PRISMA-ScR is released. The flow chart should clearly detail the review decision process, indicating the results from the search, removal of duplicate citations, study selection, full retrieval and additions from a third search, and final summary presentation.

The narrative summary should logically describe the aims or purposes of the reviewed sources, concepts adopted and results that relate to the review question/s.

The results may be classified under main conceptual categories such as: "intervention type", "study population" (and sample size, if it is the case), "duration of intervention", "aims", "methodology adopted", "key findings" (evidence established) and "gaps in the research". For each category, a clear explanation should be provided.

This section should include an overall description of the included sources with reference to the detailed Table of Included Study Characteristics in the appendices (the template data extraction tool in Appendix 1 can be readily modified by reviewers to suit this purpose) . The aim of this section is to provide detail to support the inclusion of each source (paper, study, report, etc.) in the scoping review. For each source, identify the relevance to the scoping review objective and evidence for the review question. Specific results from sources may be highlighted. A summary table of included studies should be provided in the appendices of the scoping review.

Presentation of the results may map out the reviewed material in logical, diagrammatic or tabular form, and/or in a descriptive format that aligns with the objective and scope of the review. The tables and charts may show results as: distribution of studies by year or period of publication (depends on each case), countries of origin, area of intervention (clinical, policy,

For peer review only - http://bmjopen.bmj.com/site/about/guidelines.xhtml

educational, etc.), and research methods.

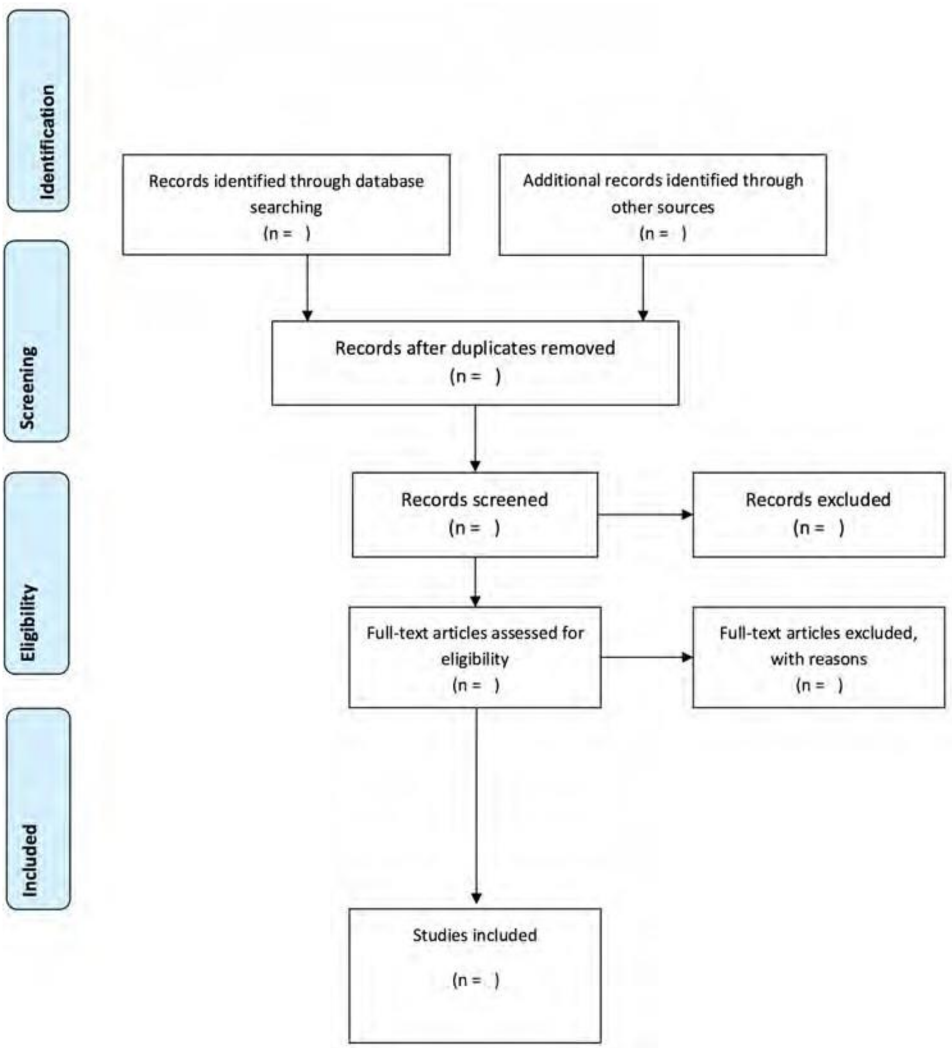

Figure 11.1: Flow Diagram for the scoping review process adapted from the PRISMA statement by Moher and colleagues (2009).

For peer review only - http://bmjopen.bmj.com/site/about/guidelines.xhtml

3.7 Discussion, Conclusion, and Implications for research and practice

3.7.1 Discussion

This section should discuss the results of the review as well as any limitations of the sources included in the scoping review. Results should be discussed in the context of current literature, practice and policy. Scoping reviews are subject to the limitations of any review, relevant sources of information may be omitted and the review is dependent on information on the review question being available. In a scoping review no rating of the quality of evidence is provided, therefore recommendations for practice cannot be graded.

3.7.2 Conclusions

This section should begin with an overall conclusion based on the results. The conclusions drawn should match the review objective/s and question/s.

3.7.3 Implications for research

This section should include clear, specific recommendations for future research based on gaps in knowledge identified from the results of the review. Authors may be able to make comments about the future conduct of systematic reviews that may be appropriate, or primary research in the area of interest.

3.7.4 Implications for practice

This section should include clear results from the scoping review that can be used to inform practice. It may not be possible to develop recommendations for practice from the results of a scoping review as no assessment of methodological quality takes place as part of a scoping review. As such this section may be left out. If implications for practice are included, the JBI Grades of Recommendation must be used (The Joanna Briggs Institute, 2014).

3.5 References

For publication in the JBI Database of Systematic Reviews and Implementation Reports, all references should be listed in full using the Vancouver referencing style, in the order in which they appear in the review. Abbreviated journal titles must be used in accordance with the United States National Library of Medicine (2016).

For peer review only

For peer review only - http://bmjopen.bmj.com/site/about/guidelines.xhtml

For peer review only

4 Review Appendices

Appendices should be numbered using Roman numerals in the order in which they have been referred to in the body of the text. While reviewers may choose to develop additional appendices for details that are unfeasible to present in the main body of the report, there are two required appendices for a JBI scoping review:

Appendix I: Search strategy

A detailed search strategy for at least one of the major databases searched must be appended.

Appendix II: Data extraction instrument

The data extraction instrument used must be appended (see the template in Appendix 11.1)

For peer review only

For peer review only - http://bmjopen.bmj.com/site/about/guidelines.xhtml

5. Chapter References

Anderson, S, Allen, P, Peckham, S & Goodwin, N 2008, ‘Asking the right questions: scoping studies in the commissioning of research on the organisation and delivery of health services’, *Health Res Policy Syst*, vol. 6, no. 7, viewed 20 March 2017, (online PubMed Central/US National Library of Medicine, National Institutes of Health).

Arksey, H & O’Malley, L 2005, ‘Scoping studies: towards a methodological framework’, *Int J Soc Res Methodol*, vol. 8, no. 1, pp.19-32.

Armstrong, R, Hall, BJ, Doyle, J, & Waters, E 2011, ‘Scoping the scope of a cochrane review’, *J Public* Health vol. 33, no. 1, pp. 147-50.

Centre for Reviews and Dissemination n.d., ‘*PROSPERO International prospective register of systematic reviews’*, CRD The University of York, York, viewed 17 March 2017, https://www.crd.york.ac.uk/PROSPERO/about.php?about=inclusioncriteria

Crilly, T, Jashapara, A & Ferlie, E 2009, *‘Research utilisation and knowledge mobilisation: a scoping review of the literature*’. London: Department of Management, King’s College London.

Davis, K, Drey, N & Gould, D 2009, ‘What are scoping studies? A review of the nursing literature’, *Int J Nurs Stud*, vol. 46, no. 10, pp.1386-400.

Decaria, J, Sharp, C & Petrella, R 2012, ‘Scoping review report: obesity in older adults’, *Int J Obesity*, vol. 36, no. 9, pp. 1141-50.

de Chavez, AC, Backett-Milburn, K, Parry, O & Platt, S 2005, ‘ *Understanding and researching wellbeing: Its usage in different disciplines and potential for health research and health promotion*’, Health Educ J, vol. 64, no.1, pp. 70-87.

Ehrich, K, Freeman, GK, Richards, SC, Robinson, IC, & Shepperd, S 2002, ‘*How to do a scoping exercise: continuity of care*’, Res Pol Plan, vol. 20, no. 1, pp. 25-9.

Grant, MJ & Booth, A 2009, *‘A typology of reviews: an analysis of 14 review types and associated methodologies’*, Health Info Libr J, vol.26, no. 2, pp. 91-108.

The Joanna Briggs Institute Levels of Evidence and Grades of Recommendation Working Party 2014, *Supporting Document for the Joanna Briggs Institute Levels of Evidence and Grades of Recommendation*: The Joanna Briggs Institute, Adelaide, viewed 20 March 2017,

For peer review only - http://bmjopen.bmj.com/site/about/guidelines.xhtml

http://joannabriggs.org/jbi-approach.html#tabbed-nav=Levels-of-Evidence

Kao, SS, Peters, MDJ, Dharmawardana, N, Stew, B, & Ooi, EH 2017a, 'Pediatric tonsillectomy quality of life assessment instruments: a scoping review', *Laryngoscope.* 8 March, [Epub ahead of print].

Kao, SS, Peters, MDJ, & Ooi, E 2017b, 'Pediatric tonsillectomy quality of life assessment instruments: a scoping review protocol', *JBI Database System Rev Implement Rep.* [In Press].

Levac, D, Colquhoun, H & O'Brien, KK 2010, 'Scoping studies: advancing the methodology', *Implement Sci*, ed. 5, vol. 1, pp. 1-9.

Moher, D, Liberati, A, Tetzlaff, J & Altman, DG 2009, 'Preferred reporting items for systematic reviews and meta-analyses: the PRISMA statement', *Ann Intern Med*, vol. 151, ed. 4, pp. 264-9.

Mordiffi, SZ, Peters, MDJ & Ang, ENK 2016, 'Non-invasive thermometers used in healthcare facilities: A scoping review protocol'. *JBI Database System Rev Implement Rep'*, vol. 14, ed. 11, pp. 106-12.

Pearson, A, Wiechula, R, Court, A & Lockwood, C 2005, 'The JBI model of evidence–based healthcare', *Int J Evid Based Healthc*, vol. 3 ed. 8, pp. 207-15.

Pearson, A, White, H, Bath-Hextall, F, Salmond, S., Apostolo, J. and Kirkpatrick, P 2015, 'A mixed-methods approach to systematic reviews', *Int J Evid Based Healthc*, vol. 13, ed. 3, pp.121-31.

Peters, MDJ, Godfrey, C, Kahlil, H, McInerney, P, Baldini Soares, C & Parker, D 2015, 'Guidance for conducting systematic scoping reviews'. *Int J Evid Based Healthc,* vol. 13, ed. 3, pp. 141-46.

Peters, MDJ 2016, 'In no uncertain terms: the importance of a defined objective in scoping reviews', *JBI Database System Rev Implement Rep,* vol. 14, ed. 2, pp. 1-4.

Pham, MT, Rajić, A, Greig, JD, Sargeant, JM, Papadopoulos, A & McEwen, SA 2014, 'A scoping review of scoping reviews: advancing the approach and enhancing the consistency'. *Res Synth Methods,* vol. 5, ed. 4, pp. 371-85.

Reilly, R, Evans, K, Gorham, G, Peters, MDJ, Warren, S, O'Shea, R, Brown, A, Cass, A & Gomersall, J 2016, 'Effectiveness, cost effectiveness, acceptability and implementation barriers/enablers of chronic kidney disease management programs for Indigenous people in

For peer review only - http://bmjopen.bmj.com/site/about/guidelines.xhtml

Australia, New Zealand and Canada: a systematic review of mixed evidence', *BMC Health Serv Research,* vol. 16. 6 April, pp.119-33.

Tricco, AC, Ashoor, HM, Cardoso, R, MacDonald, H, Cogo, E, Kastner, M, Perrier, L, McKibbon, A, Grimshaw, JM & Straus, SE 2016a, 'Sustainability of knowledge translation interventions in healthcare decision-making: a scoping review' *Implement Sci*, ed. 11, vol. 1, p.55.

Tricco, AC, Lillie, E, Zarin, W, O'Brien, K, Colquhoun, H, Kastner, M, Levac, D, Ng, C, Pearson Sharpe, J, Wilson, K, Kenny, M, Warren, R, Wilson, C, Stelfox, HT & Straus, SE 2016b, 'A scoping review on the conduct and reporting of scoping reviews', *BMC Med Res Methodol*, vol. 16, ed. 15, viewed 20 March 2017, (online PubMed Central/US National Library of Medicine, National Institutes of Health).

Tricco, AC, Strauss, S & Moher, D 2015, *Preferred Reporting Items for Systematic Reviews and Meta-Analysis: extension for Scoping Reviews (PRISMA-ScR)*, Enhancing the QUAlity and Transparency Of health Research (EQUATOR) Network, Centre for Statistics in Medicine, NDORMS, University of Oxford, London, viewed 17 March 2017, http://www.equator-network.org/library/reporting-guidelines-under-development/#55

The United States National Library of Medicine (U.S. NLM). 2016. *FAQ: NLM® LocatorPlus® -- Database of NLM-Held Journal Titles and Abbreviations*, Rockville Pike, Bethesda, viewed 17 March 2017, https://www.nlm.nih.gov/services/lpabbrev.html

Valaitis, R, Martin-Misener, R, Wong, ST, MacDonald, M, Meagher-Stewart, D, Austin, P, Kaczorowksi, J, O-Mara, L, Savage, R, Strengthening Primary Health Care through Public Health and Primary Care Collaboration Team 2012, 'Methods, strategies and technologies used to conduct a scoping literature review of collaboration between primary care and public health', *Prim Health Care Res Dev*, vol. 13, ed. 3, pp. 219-36.

Watson, R, Parr, JR, Joyce, C, May, C & Le Couteur, AS 2011, 'Models of transitional care for young people with complex health needs: a scoping review', *Child Care Health Dev,* vol. 37, ed. 6, pp.780-91.

For peer review only - http://bmjopen.bmj.com/site/about/guidelines.xhtml

4.1:Appendix 1: JBI Template study details, characteristics and results extraction instrument

**Scoping Review Details**	
Scoping Review title:	
Review objective/s:	
Review question/s:	
**Inclusion/Exclusion Criteria**	
Population	
Concept	
Context	
Types of Study	
**Study Details and Characteristics**	
Study citation details (e.g. author/s, date, title, journal, volume, issue, pages)	
Country	
Context	
Participants (details e.g. age/sex and number)	
**Details/Results extracted from study** (in relation to the concept of the scoping review)	
E.g. Quality of Life Domains assessed	
E.g. Number of items in tool	
E.g. details of psychometric validation of tool	

For peer review only

For peer review only - http://bmjopen.bmj.com/site/about/guidelines.xhtml

For peer review only

For peer review only - http://bmjopen.bmj.com/site/about/guidelines.xhtml

View publication stats

BMJ Open

Statistical Methods Applied for the Assessment of the HIV Cascade and Continuum of Care: A Systematic Scoping Review

Journal:	*BMJ Open*
Manuscript ID	bmjopen-2022-071392.R1
Article Type:	Original research
Date Submitted by the Author:	02-Jul-2023
Complete List of Authors:	Kalinjuma, Aneth Vedastus; Ifakara Health Institute; Faculty of Health Sciences University of the Witwatersrand<br>Glass, Tracy Renée; Swiss Tropical and Public Health Institute; University of Basel<br>Masanja, Honorati; Ifakara Health Institute, P.O Box 78373, Dar es Salaam, Tanzania, Health<br>Weisser, Maja; Ifakara Health Institute; University of Basel<br>Msengwa, Amina Suleiman; University of Dar es Salaam, Department of Statistics<br>Vanobberghen, Fiona; Swiss Tropical and Public Health Institute; University of Basel<br>Otwombe, Kennedy; University of the Witwatersrand Perinatal HIV Research Unit, Statistics and Data Management Centre; School of Public Health, Faculty of Health Sciences, University of the Witwatersrand, Johannesburg, South Africa
**Primary Subject Heading**:	HIV/AIDS
Secondary Subject Heading:	Research methods
Keywords:	STATISTICS & RESEARCH METHODS, HIV & AIDS < INFECTIOUS DISEASES, Public health < INFECTIOUS DISEASES

SCHOLARONE™
Manuscripts

I, the Submitting Author has the right to grant and does grant on behalf of all authors of the Work (as defined in the below author licence), an exclusive licence and/or a non-exclusive licence for contributions from authors who are: i) UK Crown employees; ii) where BMJ has agreed a CC-BY licence shall apply, and/or iii) in accordance with the terms applicable for US Federal Government officers or employees acting as part of their official duties; on a worldwide, perpetual, irrevocable, royalty-free basis to BMJ Publishing Group Ltd ("BMJ") its licensees and where the relevant Journal is co-owned by BMJ to the co-owners of the Journal, to publish the Work in this journal and any other BMJ products and to exploit all rights, as set out in our licence.

The Submitting Author accepts and understands that any supply made under these terms is made by BMJ to the Submitting Author unless you are acting as an employee on behalf of your employer or a postgraduate student of an affiliated institution which is paying any applicable article publishing charge ("APC") for Open Access articles. Where the Submitting Author wishes to make the Work available on an Open Access basis (and intends to pay the relevant APC), the terms of reuse of such Open Access shall be governed by a Creative Commons licence – details of these licences and which Creative Commons licence will apply to this Work are set out in our licence referred to above.

Other than as permitted in any relevant BMJ Author's Self Archiving Policies, I confirm this Work has not been accepted for publication elsewhere, is not being considered for publication elsewhere and does not duplicate material already published. I confirm all authors consent to publication of this Work and authorise the granting of this licence.

For peer review only

Statistical Methods Applied for the Assessment of the HIV Cascade and Continuum of Care: A Systematic Scoping Review

Aneth Vedastus Kalinjuma[1,2*], Tracy Renée Glass[3,4], Honorati Masanja[1], Maja Weisser[1,3,4,5], Amina Suleiman Msengwa[6], Fiona Vanobberghen[3,4]†, and Kennedy Otwombe[2,7]†

†Contributed equally

Affiliations

[1]Ifakara Health Institute, Ifakara, Morogoro, Tanzania

[2]School of Public Health, Faculty of Health Sciences, University of the Witwatersrand, Johannesburg, South Africa

[3]Swiss Tropical and Public Health Institute, Allschwil, Switzerland

[4]University of Basel, Basel, Switzerland.

[5]Division of Infectious Diseases and Hospital Epidemiology, University Hospital Basel, University of Basel, Switzerland

[6]Department of Statistics, University of Dar es Salaam, Tanzania

[7]Perinatal HIV Research Unit, Chris Hani Baragwanath Academic Hospital, Faculty of Health Sciences, University of the Witwatersrand, Johannesburg, South Africa

***Corresponding Author**

Aneth Vedastus Kalinjuma, Ifakara Health Institute, P.O. Box 78383, Mikocheni, Dar es Salaam, Tanzania.

Email: avedastus@ihi.or.tz and Phone: +255 222 774 714

Word count: Abstract = 360 and Main text = 4168

For peer review only

For peer review only - http://bmjopen.bmj.com/site/about/guidelines.xhtml

Abstract

Objectives: This scoping review aims to identify and synthesise existing statistical methods used to assess the progress of human immunodeficiency virus (HIV) treatment programs in terms of the HIV cascade and continuum of care among people living with HIV (PLHIV).

Design: Systematic scoping review.

Data sources: Published articles were retrieved from PubMed, Cumulative Index to Nursing and Allied Health Literature (CINAHL) Complete, and Excerpta Medica dataBASE (EMBASE) databases between April and July 2022. We strategically search using the Google Scholar search engine and reference lists of published articles.

Eligibility criteria: This scoping review included original English articles that estimated and described the HIV cascade and continuum of care progress in PLHIV. The review considered quantitative articles that evaluated either HIV care cascade progress in terms of the Joint United Nations Programme on HIV and AIDS (UNAIDS) targets or the dynamics of engagement in HIV care.

Data extraction and synthesis: The first author and the librarian developed database search queries and screened the retrieved titles and abstracts. Two independent reviewers and the first author extracted data using a standardized data extraction tool. The data analysis was descriptive and the findings are presented in tables and visuals.

Results: This review included 300 articles. Cross-sectional study design methods were the most commonly used to assess the HIV care cascade (n=279, 93%). In cross-sectional and longitudinal studies, the majority used proportions to describe individuals at each cascade stage (276/279 (99%) and 20/21 (95%), respectively). In longitudinal studies, the time spent in cascade stages, transition probabilities, and cumulative incidence functions were estimated. The logistic regression model was common in both cross-sectional (101/279, 36%) and longitudinal studies (7/21, 33%). Of the 21 articles that used a longitudinal

For peer review only

For peer review only - http://bmjopen.bmj.com/site/about/guidelines.xhtml

design, six articles used multistate models, which included non-parametric, parametric, continuous-time, time-homogeneous, and discrete-time multistate Markov models.

Conclusions: Most literature on the HIV cascade and continuum of care arises from cross-sectional studies. The use of longitudinal study design methods in the HIV cascade is growing because such methods can provide additional information about transition dynamics along the cascade. Therefore, a methodological guide for applying different types of longitudinal design methods to the HIV continuum of care assessments is warranted.

Keywords: Cross-sectional design; Longitudinal design; Multistate Modelling; HIV care and treatment programs

For peer review only

For peer review only - http://bmjopen.bmj.com/site/about/guidelines.xhtml

Strengths and limitations of this study

Strengths

- This review is one of the largest and most recent systematic scoping reviews conducted on the methods used to assess the HIV cascade and continuum of care with wide coverage and diversity.
- The review highlights areas with limited research in terms of methods and their applications to the HIV cascade and continuum of care and it provides recommendations for future research.

Limitation

- This review was limited to articles published in English. Therefore, the impact of including other languages in this review is unknown.
- The review included published articles. However, the pattern study findings would not be expected to change if grey literature were included.

For peer review only

For peer review only - http://bmjopen.bmj.com/site/about/guidelines.xhtml

Introduction

The HIV care cascade is a framework that describes the stages of people living with HIV (PLHIV) transitioning through HIV infection, diagnosis, linkage to care, initiation of antiretroviral therapy (ART), retention in care, and viral load suppression (1,2). This framework characterizes and quantifies the proportion of PLHIV in each stage of the cascade (3,4). It is a useful tool for monitoring the uptake of HIV services and the progress of HIV treatment programs (5–7).

In the literature, the terms 'HIV care cascade' and 'HIV continuum of care' are used interchangeably (4,5,8,9), but Kay et al. (10) distinguished the two concepts. They defined the 'HIV continuum of care' as a dynamic and bidirectional engagement in HIV care, whereas the 'HIV care cascade' was defined as a static and cross-sectional representation of HIV care stages at the population level. In recent years, many researchers have embraced these concepts, and the distinction between them has been recognised in literature, particularly after the Joint United Nations Programme on HIV and AIDS (UNAIDS) announced the 90-90-90 targets for HIV for 2020 (11) and 95-95-95 targets for 2025 (12). The 95-95-95 target states that 95% of PLHIV know their HIV status, 95% of those diagnosed are initiated on ART, and 95% of those on ART are virally suppressed.

Currently, most HIV treatment program assessments and evaluations are performed following the HIV care cascade approach, which aims to report progress toward UNAIDS targets for 2020 or 2025 (13–22). These assessments often apply descriptive statistics and show progress at a particular time point, referred to as a cross-sectional design. In recent years, assessments of the HIV cascade and continuum of care that use a longitudinal design have been increasingly featured in the published literature. In these applications, methods such as multistate models (3,5), competing risk methods (23), and the distribution time spent in each cascade stage (24) were used. The existence of various methods and their applications in the HIV cascade and continuum of care evaluation call for a review that identifies the statistical methods used in these assessments.

For peer review only - http://bmjopen.bmj.com/site/about/guidelines.xhtml

Different types of reviews exist on this topic, the majority of which focus on synthesising estimates for each HIV care cascade stage. These reviews mainly focused on HIV testing, engagement in care and treatment (25,26), cascades among alcohol users (27), longitudinal methods for assessing retention in care (28), and cascades among people who inject methadone drug(29). Others reviewed cascade stage definitions (30), measures of patient engagement in HIV care (31), cascade experiences among indigenous people (32), cascades among young children and adolescents (33) and cascades among children and adolescents (34). Currently, few systematic reviews have synthesised methods used to assess the HIV cascade and continuum of care progress. The first systematic review was conducted by Medland et al. (35) and published in 2015. At that time, few cascade articles were published, and most studies used descriptive statistics in a cross-sectional study design. Granich et al. (36) conducted a review of methods and the status of progress towards the UNAIDS 90-90-90 from 2010–2016, and they showed that different methods were used. However, they focussed mainly on articles that used a cross-sectional study design and methods used to determine indicators and data sources. In 2021, Vourli et al. (37) published a systematic review that highlighted the importance of expanding the scope of cascade assessment to include time to the next cascade stage. In these reviews, the components of the statistical methods applied to the HIV cascade and continuum of care assessments were limited. Therefore, this systematic scoping review aimed to identify and summarise the statistical methods used to assess HIV treatment program progress in terms of the HIV cascade and continuum of care among PLHIV using both cross-sectional and longitudinal study designs.

Methods

Review protocol

The scoping review protocol was developed from November to December 2021, following the Joanna Briggs Institute System for the Unified Management, Assessment, and Review of Information (JBI SUMARI) Manual for Evidence Synthesis (Scoping Review Chapter) (38).

For peer review only - http://bmjopen.bmj.com/site/about/guidelines.xhtml

Eligibility criteria

This scoping review included original articles written in English that focused on estimating and describing the HIV cascade and continuum of care among PLHIV. Specifically, the review considered quantitative articles that either evaluated or assessed the HIV care cascade in terms of UNAIDS targets or the dynamics of engagement in HIV care and treatment clinics for the HIV continuum of care. Mixed method articles were included if the quantitative component was well described (i.e., the method used to assess the cascade and results of the cascade were presented in the article). The review excluded articles comparing the HIV care cascade with other cascades, such as the TB care cascade, hepatitis cascade, and prevention of mother-to-child transmission of HIV. The review further excluded review and meta-analysis articles, opinions, editorials, commentary articles, and non-peer-reviewed articles (because the methods sections were not well-described). Finally, the review did not include published conference abstracts or proceedings, because the methods were typically not provided in sufficient detail.

Search strategy

The development of an electronic bibliographical database search strategy began with a pilot phase, whereby review concepts were searched independently in PubMed and Google Scholar. The retrieved relevant titles and abstracts were screened to identify keywords to include in the development of electronic database search queries. The database search query was first developed in English using the PubMed database and adapted to the Cumulative Index to Nursing and Allied Health Literature (CINAHL) Complete and Excerpta Medica dataBASE (EMBASE) databases as needed (Table S1). The database search dates were from April to July 2022.

Source of the evidence selection process

For peer review only - http://bmjopen.bmj.com/site/about/guidelines.xhtml

Titles and abstracts were retrieved from all three databases, and duplicates were removed based on the digital object identifier and article content. Titles and abstracts were screened for eligibility by the lead author and irrelevant articles were excluded. All potentially relevant full-text articles were downloaded for review. The first author and two independent reviewers each conducted a full-text review of all articles, during which the review criteria were applied. The article search and assessment of inclusion and exclusion criteria were summarised and presented using the Preferred Reporting Items for Systematic Reviews and Meta-Analyses extension for Scoping Reviews (PRISMA-ScR) flow diagram (39).

Data extraction and data entry process

The extracted information included the first author's name and publication year, type of participants, age, country, sample size, study design, study level of representation, and data source. Other extracted information included cascade stages, cascade size, cascade staging methods, and statistical methods used to assess the HIV cascade and continuum of care (Table S2). All extracted information was first entered into an Excel spreadsheet. The data extraction tool was modified and revised whenever necessary, depending on new information available in the articles and its relevance to this review. The first author reviewed all extracted data. Disagreements between reviewers were resolved by consensus. After finalising the data collection template, an electronic data capture system was developed using Open Data Kit (ODK), and data entry was performed by the reviewers. The information entered into ODK was pooled in Excel with a comma-separated value file format using the ODK briefcase.

Data items

The variables used in this review included publication year, which included three groups: 2011–2014, referring to the time between the first cascade report and before the announcement of the UNAIDS 90-90-90 targets; 2015–2019, following the UNAIDS 90-90-90 targets announcement and the implementation

For peer review only - http://bmjopen.bmj.com/site/about/guidelines.xhtml

For peer review only

time; 2020–2022 (by July), representing the reporting time for the UNAIDS 90-90-90 targets and the implementation of the new UNAIDS 95-95-95 targets. The study site was country-specific if a study was conducted in one country and regionally if a study was conducted in at least two countries. The study regions included Asia and Europe, North and South America, sub-Saharan Africa, and global (a single report that included multiple regions).

A cross-sectional study design included articles that presented cascade results at a single time point. A longitudinal design included articles that presented cascade outcomes either repeatedly over time (i.e. repeated cross-sectional study design), or prospective or retrospective study designs (including cohort data) (40). In this review, studies that included cascade assessments using both cross-sectional and longitudinal designs were included in the longitudinal study design group, because cross-sectional analyses can be considered a subset of longitudinal analyses.

Other variables included data sources (cohort, laboratory test records, hospital records, surveys, surveillance, and clinical trial data) and study level of representation, which was grouped into facility, sub-district/town/municipal/city, state/province, population (when a study estimated the number of PLHIV, diagnosed, and linkage to care), community/district/county, regional, country, and global studies. Another variable was the sample size (the number of diagnosed HIV-positive participants assessed). The sample size was grouped into four categories: <1000, 1000–5000, 6000–10000, and >10000. The types of participants were grouped into all PLHIV, children only, adolescents, adults, HIV key populations, prisoners, veterans, refugees, and the military. The HIV key populations included men who had sex with men, female sex workers, people who inject drugs, and transgender men and women. Finally, the statistical methods applied in the different articles were grouped into two categories: descriptive statistics and regression models. Variables such as study design, data sources, types of participants, and statistical methods, were allowed for multiple responses; the obtained responses were converted into multiple individual variables with binary responses.

For peer review only

For peer review only - http://bmjopen.bmj.com/site/about/guidelines.xhtml

Stage names with similar meanings were reorganised to formulate simple cascade stage names, such as HIV infection (PLHIV), diagnosis, linkage to care, loss to follow-up (LTFU), retention in care, transfer to other HIV clinics, ART eligibility, on ART, viral load suppression, and death. Articles that used a longitudinal design and multistate models had slightly different names for their cascade stages. In this case, stage names were used as they were written by the authors. The size of the cascade and continuum of care frameworks was categorised into four groups based on the number of stages assessed. The four categories were extra-large cascades (> 6 stages), large cascades (5–6 stages), medium cascades (4 stages), and small cascades (≤ 3 stages).

Types of cascade framework used were grouped into six: the CDC 2011 cascade framework (including PLHIV, diagnosis, linkage to care, retention in care, ART status, and viral load suppression status stages (2)). CDC with five stages (starting from the diagnosis stage), and CDC with four stages (starting from linkage to care). Gourlay and others in 2017 (here abbreviated as Gourlay 2017) included PLHIV, diagnosis, ART status, and viral load suppression stages (41). Gourlay 2017 with three stages started from the diagnosis stage. Other cascade formulations included stages such as ART eligibility, clinical staging, ART adherence, LTFU/disengagement, engagement in care, death, transfer out, CD4 testing, current use of ART, and history of ART. The cascade staging method used was grouped into three categories: dependent (also known as conditional), independent, and a combination of the two. In the dependent method, PLHIV are eligible for the next cascade stage if they are in the previous cascade stage, whereas in the independent method, each cascade stage was derived independently of the prior cascade stage (42).

Data analysis and presentation

The review findings were analysed and summarised using summary statistics, such as counts with percentages, medians with interquartile ranges (IQR), and data ranges. Results were presented using tables. The geographical distribution of articles by country was summarised using world maps. Maps were plotted for country-specific articles (excluding 28 regional articles). The constructed cascade and continuum of

For peer review only - http://bmjopen.bmj.com/site/about/guidelines.xhtml

care frameworks were summarised in terms of the size and cascade stages used. Types of cascade framework used were summarised using counts with percentage. The frameworks of a subset of articles that used a longitudinal study design and multistate models were compiled to document similarities, differences, and existing gaps. All data management and statistical analyses were performed using Stata software version 14 (StataCorp LP, College Station, Texas, USA).

Patient and Public Involvement

No patient involved

Results

Description of the article selection process

The search query retrieved 5,805 titles and abstracts from all three databases, and 5,505 titles were excluded in the title and abstract screening stages (Figure 1). and 310 abstracts were identified as meriting full-text reviews, of which 300 articles were included (Table S3).

Characteristics of articles

The majority of the 300 articles were published between 2015 and 2019 (n=177, 63%) (Table 1). Various PLHIV groups were assessed, including children, adolescents, adults, migrants, prisoners, veterans, the military, and HIV key populations. Key HIV populations were reported in 74 articles, which included men having sex with men (n=35, 47%), female sex workers (n=19, 26%), people who inject drugs (n=20, 27%), transgender men (n=8, 11%), and women (n=19, 26%).

For peer review only

For peer review only - http://bmjopen.bmj.com/site/about/guidelines.xhtml

Assessments of the HIV cascade and continuum of care were performed using cross-sectional (279/300, 93%) and longitudinal (21/300, 7%) designs (Table 1). The review included 28 studies conducted in regions or globally. Many of these studies were conducted in Asia and Europe (n=11, 39%) and in Sub-Saharan Africa (n=11, 39%). Focusing on country-specific studies, most of the articles that used a cross-sectional study design were from the USA, Canada, Zimbabwe, and South Africa (these countries each contributed between 9 and 83 of 252 studies) (Figure 2). Articles that used a longitudinal study design were mainly from the USA (6/20 studies)

Various data sources have been used to describe the HIV cascade and continuum of care. In the 279 articles that used a cross-sectional study design, the survey data source was common (n=110, 39%), whereas, in the 21 articles that used a longitudinal design, the cohort data source was often used (n=10, 48%) (Table 1). The sample size varied across articles, with the smallest study including 22 hard-to-reach individuals and the largest study including 37.9 million PLHIV (UNAIDS report of 170 countries). In articles that used a cross-sectional study design, the median sample size was 1,721 PLHIV (IQR: 524–8,744), whereas in articles that used a longitudinal design, the median was 3,311 PLHIV (IQR: 1,080–11,510). Articles that used a cross-sectional study design had various levels of representation, with many being sub-district/town/municipal/city-based studies (68/279, 24%), while for articles that used a longitudinal design, the majority were facility-based studies (6/18, 33%).

Table 1: Characteristics of articles included in the scoping review (n=300)

**Characteristics of articles**	**Cross-Sectional**	**Longitudinal[1]**	**Overall**
Papers included[2]	279/300 (93.0%)	21/300 (7.0%)	300/300 (100%)
Publication year[3]			
2011 - 2014 (Before 90-90-90)	18/279 (6.5%)	0/21 (0%)	18/300 (6.0%)
2015 - 2019 (Post 90-90-90)	177/279 (63.4%)	14/21 (66.7%)	191/300 (63.7%)
2020 - 2022 (90-90-90 reporting)	84/279 (30.1%)	7/21 (33.3%)	91/300 (30.3%)
Regional studies (involved ≥2 countries)			
Asia and Europe	10/27 (37.4%)	1/1 (100%)	11/28 (39.3%)
North and South America	5/27 (18.5%)	0/1 (0%)	5/28 (17.9%)
Sub-Saharan Africa	11/27 (40.7%)	0/1 (0%)	11/28 (39.3 %)
Global[4]	1/27 (3.7%)	0/1 (0%)	1/28 (3.6%)

For peer review only - http://bmjopen.bmj.com/site/about/guidelines.xhtml

Type of participants[5]			
Mixture (All PLHIV)	47/279 (16.9%)	3/21 (14.3%)	50/300 (16.7%)
Children only	10/279 (3.6%)	1/21 (4.8%)	11/300 (3.7%)
Adolescents	29/279 (10.4%)	5/21 (23.8%)	34/300 (11.3%)
Adult	127/279 (45.5%)	14/21 (66.7%)	141/300 (47.0%)
Key population	72/279 (25.8%)	2/21 (9.5%)	74/300 (24.7%)
Migrants	5/279 (1.8%)	0/21 (0%)	5/300 (1.7%)
Prisoners	2/279 (0.7%)	0/21 (0%)	2/300 (0.7%)
Veterans	2/279 (0.7%)	0/21 (0%)	2/300 (0.7%)
Refugees	0/279 (0%)	1/21 (4.8%)	1/300 (0.3%)
Military	0/279 (0%)	1/21 (4.8%)	1/300 (0.3%)
Types of HIV key population[5,6], n (%)			
Men having sex with men	33/72 (45.8)	2/2 (100%)	35/74 (47.3)
Female sex workers	19/72 (26.4)	0/2 (0%)	19/74(25.7)
People who inject drugs	20/72 (27.8)	0/2 (0%)	20/74 (27.0)
Transgender women	19/72 (26.4)	0/2 (0%)	19/74 (25.7)
Transgender men	7/72 (9.7)	1/2 (50.0)	8/74 (10.8)
Data sources[5]			
Cohort data	65/279 (23.3%)	10/21 (47.6%)	75/300 (25.0%)
Laboratory test records	11/279 (3.9%)	0/21 (0%)	11/300 (3.7%)
Hospital medical records	55/279 (19.7%)	5/21 (23.8%)	60/300 (20.0%)
Survey data	110/279 (39.4%)	3/21 (14.3%)	113/300 (37.7%)
Surveillance data	70/279 (25.1%)	3/21 (14.3%)	73/300 (24.3%)
Clinical trials data	4/279 (1.4%)	1/21 (4.8%)	5/300 (1.7%)
Others[7]	19/279 (6.8%)	3/21 (14.3%)	22/300 (7.3%)
Sample size, median (IQR)	1721 (524-8744)	3311 (1080-11510)	1862 (545-8859)
Sample size range (minimum, maximum)	22 – 39000000	100 – 92215	22 – 39000000
Sample size (number of participants)			
<1000	103/279 (37.3%)	5/21 (21.8%)	108/297 (36.4%)
1000 – 5000	85/279 (30.8%)	7/21 (33.3%)	92/297 (31.0%)
6000 – 10000	24/279 (8.7%)	3/21 (14.3%)	27/297 (9.1%)
>10000	64/279 (23.2)	6/21 (28.6%)	70/297 (23.6%)
Level of representation			
Facility[8]	51/278 (18.4%)	6/18 (33.3%)	57/296 (19.3%)
Sub-district/Town/Municipal/City	68/278 (24.5%)	2/18 (11.1%)	70/296 (23.7%)
State/Provide	36/278 (13.0%)	0/18 (0%)	36/296 (12.2%)
Population[9]	52/278 (18.7%)	2/18 (11.1%)	54/296 (18.2%)
Community/District/County	31/278 (11.2%)	3/18 (16.7%)	34/296 (11.5%)
Regional	17/278 (6.1%)	2/18 (11.1%)	19/296 (6.4%)
Country	22/278 (7.9%)	3/18 (16.7%)	25/296 (8.5%)
Global[4]	1/278 (0.4%)	0/18 (0%)	1/296 (0.3%)

[1]Longitudinal design articles included articles that conducted both longitudinal and cross-sectional analyses
[2]Row percentage
[3]The UNAIDS 90-90-90 target of the year 2020 stated that 90% of PLHIV know their HIV status, 90% of the diagnosed positive are initiated on ART, and 90% of those on ART are virally suppressed.
[4]Global studies are the studies included multiple regions such as Europe, Asia, America, and sub-Saharan Africa
[5]Percentage was estimated for each type of response and the report in this table is for 'yes' for all binary variables processed from multiple responses variables
[6]Analysis was restricted among the articles reported the key population participants (n=74)

For peer review only - http://bmjopen.bmj.com/site/about/guidelines.xhtml

[7]Other data sources largely included clinic and program registries, databases, different types of medical records, consortiums, population estimates reports, social insurance schemes, and AIDS program databases. IQR is interquartile range
[8]Ficility level studies at a health facility or clinic or hospital-based studies
[9]Population studies are the studies that included population-level cascade stages which included PLHIV, diagnosis, and linkage to care

HIV cascade and continuum of care stages

In general, a median of four cascade stages (IQR: 3–5) was used (Table 2). The smallest cascade had two stages, and the largest cascade had nine stages. Approximately 34% (95/279) of the cross-sectional studies used large cascades (5-6 stages), while articles that used a longitudinal study design mostly used large (5-6 stages) and small cascades (≤3 stages) (7/21, 33%). In articles that used a longitudinal design, the larger the cascade size the larger the median sample size. However, there was no clear pattern between sample size and the number of stages used in articles that used cross-sectional study design methods (Figure S1).

In articles that used a cross-sectional study design, 12% (34/279) used Gourlay 2017 cascade framework (Table 2). The CDC 2011 cascade framework was used in various forms: 15/279 (5%) studies used all six stages, and others used the shorter form with either five stages (9/279, 3%) or four stages (11/279, 4%). In 21 articles that a longitudinal study design, few used CDC 2011 framework (n=1, 5%) and Gourlay 2017 (n=1, 5%). In all articles reviewed, 71% (190/267) used dependent staging methods. In articles that used a cross-sectional study design, only 7% (17/255) used both dependent and independent methods (Table 2).

For peer review only

Table 2: Summary of various forms of outcomes used in the articles included in the scoping review (n=300)

**Outcome**	**Cross-sectional**	**Longitudinal[1]**	**Overall**
Number of cascade stages, median (IQR)	4 (3-5)	4 (3-5)	4 (3-5)
Size of the HIV care cascade, n (%)			
Extra-large cascade (> 6 stages)	15/279 (5.4%)	1/21 (4.8%)	16/300 (5.3%)
Large cascade (5-6 stages)	95/279 (34.1%)	7/21 (33.3%)	102/300 (34.0%)
Medium cascade (4 stages)	75/279(26.8%)	6/21 (28.6%)	81/300 (27.0%)

For peer review only - http://bmjopen.bmj.com/site/about/guidelines.xhtml

Small cascade (≤3 stages)	94/279 (33.7%)	7/21 (33.3%)	101/300 (33.7%)
Sample size by size of the HIV care cascade, Median (IQR)			
Extra-large cascade (> 6 stages)	3295 (595-7489)	NA	3674 (645-10315)
Large cascade (5-6 stages)	1418 (500-7843)	7810 (1532-32242)	1532 (592-8382)
Medium cascade (4 stages)	2355 (440-10076)	3287 (2196-6850)	2617 (463-9829)
Small cascade (≤3 stages)	1999 (579-9150)	476 (175-11510)	1931 (528-9150)
Types of cascade framework used			
CDC 2011[2]	15/279 (5.4%)	1/21 (4.8%)	16/300 (5.3%)
Gourlay 2017[3]	34/279 (12.2%)	1/21 (4.8%)	35/300 (11.7%)
CDC 2011 with 5 stages[4]	9/279 (3.2%)	0/21 (0%)	9/300 (3.0%)
CDC 2011 with 4 stages[5]	11/279 (3.9%)	0/21 (0%)	11/300 (3.8%)
Gourlay 2017 with 3 stages[6]	12/279 (4.3%)	0/21 (0%)	12/300 (4.0%)
Other[7]	198/279 (71.0%)	19/21 (90.5%)	217/300 (72.3%)
Cascade staging approaches[8], n (%)			
Dependent[9]	183/255 (71.8%)	7/12 (58.3%)	190/267 (71.2%)
Independent[10]	55/255 (21.6%)	4/12 (33.3%)	59/267 (22.1%)
Both dependent and independent	17/255 (6.9%)	1/12 (8.3%)	18/267 (6.7%)

[1]Longitudinal design articles included articles that conducted both longitudinal and cross-sectional analyses

IQR is the interquartile range

[2]Six cascade stages were used as follows: PLHIV, diagnosis, linkage in care, retention in care, on ART, and viral load suppression

NA is not applicable because there was one study under this group with a sample size of 23,227

[3]Four stages were used as follows: PLHIV, diagnosis, on ART, and viral load suppression

[4]The cascade adopted stages like CDC 2011, but the assessment started from the diagnosis stage

[5]The cascade adopted stages like CDC 2011, but the assessment started from linkage in the care stage

[6]The cascade adopted stages like Gourlay and others in 2017, but the assessments started from the diagnosis

[7]Other cascade formulations that included other stages and longitudinal frameworks

[8]Studies used longitudinal and multistate models or survival analyses, staging methods for each cascade stage are not applicable

[9]The dependent method, PLHIV are eligible for the next cascade stage if they achieved the prior cascade stage

[10]Independent method, each cascade stage is derived independently of the prior cascade stage

In both study designs, common stages were PLHIV, diagnosis, linkage to care, ART status, viral load suppression, death, and LTFU (Table S4). In articles that used a longitudinal design, some used other stages such as optimal care, sub-optimal care, disengaged, and no ART. Lastly, Two articles used a longitudinal design with formulated stages in combination with either ART status or CD4 cell count (Figure S2).

For peer review only - http://bmjopen.bmj.com/site/about/guidelines.xhtml

Statistical methods applied

In the descriptive analyses, the majority of articles used proportions to describe participants at each cascade stage (cross-sectional design (276/279, 99%) and longitudinal design (20/21, 95%)) (Table 3). Articles that used a longitudinal study design often estimated transition probabilities between cascade stages (5/21, 24%), which was rare among studiesused a cross-sectional design (1/279, 0.4%). Furthermore, the time spent in each cascade stage (5/21, 24%) and the Kaplan–Meier or cumulative incidence function (5/21, 24%) were often estimated in the longitudinal studies. Approximately 28% (78/300) of the articles in both designs used simple test statistics, such as the Chi-square test, Fisher's exact test, t-test, Wilcoxon rank-sum test, and Kruskal–Wallis test.

To assess the association between cascade stages or transitions and participant characteristics, 176/300 (59%) of the included articles performed regression analysis. The regression models used in the reviewed articles primarily considered discrete and survival outcomes (Table 3). In the articles that used a cross-sectional study design, the logistic regression model was common (101/279, 36%). Logistic and Cox proportional hazard models were commonly used in studies that used a longitudinal design (7/21, 33%) and (4/21, 19%), respectively.

For peer review only

Table 3: Statistical methods used in articles included in the scoping review (n=300)

**Methods**	**Cross-sectional**	**Longitudinal**[1]	**Overall**
Descriptive statistics[2], n (%)			
Proportion for each stage of the HIV care cascade	276/279 (98.9%)	20/21 (95.2%)	296/300 (98.7%)
Simple test statistics[3]	78/279 (28.0%)	6/21 (28.6%)	84/300 (28.0%)
Time spent in each stage of the HIV care cascade	7/279 (2.5%)	5/21 (23.8%)	12/300 (4.0%)
Transition probabilities[4]	1/279 (0.4%)	5/21 (23.8%)	6/300 (2.0%)
Kaplan-Meier or cumulative incidence function	0/279 (0%)	5/21 (23.8%)	5/300 (1.7%)
Regression models[1], n (%)			
Logistic model	101/279 (36.2%)	7/21 (33.3%)	108/300 (36.0%)
Cox PH model	15/279 (5.4%)	4/21 (19.1%)	19/300 (6.3%)
Poisson model	32/279 (11.5%)	0/21 (0%)	32/300 (10.7%)
Competing risk model	3/279 (1.1%)	0/21 (0%)	3/300 (1.0%)
Generalized linear model	10/279 (3.5%)	0/21 (0%)	10/300 (3.3%)
Weighted and unweighted linear model	4/279 (1.4%)	0/21 (0%)	4/300 (1.3%)

For peer review only - http://bmjopen.bmj.com/site/about/guidelines.xhtml

Log-Binomial regression model	5/279 (1.8%)	0/21 (0%)	5/300 (1.7%)
Multinomial regression model	0/279 (0%)	3/21 (14.3%)	3/300 (1.0%)
Generalized estimating equation model	1/279(0.4%)	0/21 (0%)	1/300 (0.3%)
Other models[5]	19/279 (6.8%)	2/21 (9.5%)	21/300 (7.0%)

[1]Longitudinal design articles included articles that conducted both longitudinal and cross-sectional analyses
[2]Percentage was estimated for each type of response and the report in this table if for yes for all binary variables processed from multiple responses variables
[3]Simple test statistics used included the Chi-Square test, Fisher-Exact test, t-test, Wilcoxon rank sum test, and Kruskal–Wallis test
[4]Transition probability is a predicted probability of moving between two consecutive states in a given time
[5]Other models applied were ARIMA and SIRIMA, Bayesian hierarchical models, bivariable and multivariable regression, generalized additive model, negative binomial model, generalized linear mixed model, spectrum model, and generalized linear mixed models

Longitudinal design with multistate models

Among the 21 articles that used a longitudinal study design, different analysis methods were used. Articles that used longitudinal analysis methods were mainly in five groups (Table S5): classic survival analyses (n=2), survival analysis with competing risk methods (n=5), repeated cross-sectional and time spent in each cascade method (n=2), survival analysis with multistate models (n=6), and repeated measure analysis methods (n=1).

In articles that used a longitudinal study design and multistate models (n=6), five types of multistate models were used. These included non-parametric multistate; parametric, continuous-time multistate; time-homogeneous Markov models; discrete-time multistate Markov; and general multistate models (Table 4). Three articles adopted the Markov process, and one study considered the Markov process with first-order dependence. In almost all reviewed articles (n=5), the assessment of the care cascade started at enrolment in HIV care, and one article considered ART initiation as the baseline. Regardless of the type of multistate model used, death was considered an absorbing state in all the reviewed articles. Other absorbing states included LTFU and transfer to other HIV clinics. Three studies used a model that allowed back and forth for LTFU and disengagement states (Figure S2).

For peer review only - http://bmjopen.bmj.com/site/about/guidelines.xhtml

Table 4: Articles that used longitudinal study design and multistate model (n=6)

Methods	Number of articles
Baseline[1]	
Enrolment/recruitment	5
ART initiation	1
Reason for censoring[2]	
End of follow-up	2
Transfer, death, and follow-up	1
Transfer out, death, and status less than 12 months	1
Absorbing[3] state	
Death	2
Death and LTFU	2
Death and transfer out	2
Types of multistate models	
Non-Parametric[4] multistate model	1
Parametric[5], continuous-time[6] multistate	1
Time-homogeneous[7] Markov model	1
Discrete-time[8] multistate Markov model	2
General multistate model	1
Multistate model assumptions	
First-order dependence	1
Markov process[9]	2
Markov, first-order dependence	1
Missing data techniques	
Multiple imputations	2
Missing data indicator	3

[1]Baseline is the time at which the assessment of the cascade started
[2]Censoring occurs when the exact survival time is unknown from the start until the end of the study
[3]Absorbing state is a state in which once visited no out-of-state transition is possible
[4]Non-parametric model is the model that does not take any distribution assumptions
[5]Parametric model is the model that follows a certain distribution for example Exponential and Weibull
[6]Continuous-time model is the model used in a dataset collected in unequal-spaced time
[7]Time-homogenous model is the model that assumes transition intensity rates are constant over time
[8]Descrite-time model is the model used in a dataset collected in the equally-spaced time interval
[9]Markov assumption is the assumption used in multistate models that assumes the future transition is only dependent on the current state

Discussion

The findings of this scoping review showed that cross-sectional study design methods were most commonly used to assess the progress of HIV service uptake and treatment responses (93%). The reviewed articles

For peer review only - http://bmjopen.bmj.com/site/about/guidelines.xhtml

were mainly available at the subnational level. The articles used a cross-sectional study design covering a wide range of PLHIV, including children, adolescents, adults, migrants, prisoners, and key populations. In general, the cascade size had a median of four stages (IQR: 3–5). The dependent staging method was often used in articles with a cross-sectional study design. Various longitudinal design methods were used, including; repeated cross-sectional, repeated measures, and survival analyses. In articles that used a longitudinal study design and multistate methods (n=6 out of 21 articles), only three articles used analytical frameworks that integrated the in- and out-of-care movements of PLHIV enrolled in care. Although cascade researchers pointed out challenges of using a cross-sectional design in 2015 (4,6), articles using a longitudinal design and addressing the in- and out-of-care pathways in cascade assessments are scarce. The major reason being these methods requires advanced data management and analysis skills (43,44).

Several authors have proposed different types of frameworks that include various pathways through the HIV cascade and continuum of care. The 'HIV states and Transition' framework was formulated to capture the interaction of PLHIV with HIV testing, care, and treatment services at a given time (6). It has two key features: mutually exclusive states (cascade stages) and transitions between states. This framework was adapted by the articles that expanded the cascade assessment using longitudinal design with multistate methods (Figure S2) (5,8,45–47). Others proposed the 'comprehensive HIV care cascade' which is a framework formulated by combining stages used in the standard HIV care cascade with the additional consideration of poor outcomes (LTFU or death before or after ART initiation) (48). The 'Cyclical cascade of HIV care' was proposed to capture the nonlinearity of PLHIV engagement in HIV treatment clinics (49). The framework also maintained stages of the standard HIV care cascade (diagnosis, linkage, and ART initiation) with additional stages of early retention (<6 months) and long retention (>6 months). Disengagement is incorporated with the possibility of re-engaging in care.

In this review, less than half (n=83, 28%) used either the CDC 2011 or Gourlay 2017 cascade frameworks to quantify and describe the HIV cascade and continuum of care. Other researchers modified the cascade for various reasons. First, data availability, which affects mostly stages included; for example, some studies

For peer review only - http://bmjopen.bmj.com/site/about/guidelines.xhtml

started with PLHIV linked in care (50–52), while others did not have viral load data (53). In articles that used longitudinal design and multistate methods, the lack of transfer-out information (8) and failure to capture re-engagement in care after LTFU (46) affected the inclusion of these stages. Second, the HIV management guidelines used during the cascade assessments changed over time. This resulted in the inclusion of cascade stages such as ART eligibility (54), and CD4 testing (55). Third, additional information on the cascade. This was the case when ART status was further divided into two stages ever on ART or history of ART use and current use of ART (56–58). Others added the ART adherence stage (56,59,60) or used the retention stage in both pre-and post-ART initiation (61). Lastly, addressing the in and out of care transitions. In assessments that used a longitudinal design with multistate methods, engagement in care was expanded to capture PLHIV who were LTFU or disengaged from care and later returned to care (5,45).

Almost all articles quantified the cascade using the proportion of PLHIV achieving each cascade stage. Quantification was done either by using a dependent or independent staging method (17). The independent staging method is often used in combination with the dependent method, but it is used mainly to assess the proportion of PLHIV with suppressed viral load (62). This is because the independent staging method reflects the actual uptake of health services (63), while dependent staging methods mainly focus on PLHIV retained in care (16,64). Furthermore, approximately 60% of the articles performed regression analyses to examine the relationship between patient characteristics and either each of the cascade stages (54,65–68) or a few selected cascade stages (69–73). In these assessments, modifiable characteristics were identified to plan for interventions that will improve cascade progress in stages with poor progress.

This review revealed different longitudinal design methods. Assessments were mostly conducted using survival methods. These were mainly in three groups; classic survival analysis, competing risk methods, and multistate models. Competing risk methods were used primarily used to estimate the cumulative probabilities function of each stage (17,23), whereas, in multistate methods, the aim was to capture all possible transitions between stages (5,47).

For peer review only

For peer review only - http://bmjopen.bmj.com/site/about/guidelines.xhtml

The multistate methods used included parametric and non-parametric multistate, continuous-time and discrete-time multistate, and general multistate models. A multistate model is a continuous-time stochastic process that allows individuals to move between a finite number of states (74). In the HIV cascade and continuum of care, states correspond to the HIV cascade stages (5,6,45). In multistate modelling frameworks, states can be either transient (participants can move in and out of the state) or absorbing (no transition is possible out of the state) (75). In this review, articles that used multistate models considered death and transfer to other HIV clinics as absorbing states. Depending on the availability of data, LTFU and disengagement from care were allowed to be transient or absorbing states (Figure S2).

This review is among the largest and most recent systematic scoping reviews conducted on the methods used to assess the HIV cascade and continuum of care with extensive coverage and diversity. Furthermore, because of its coverage, the review identifies areas with limited research in terms of methods and their applications to the HIV cascade and continuum of care and provides recommendations for future studies. However, there were some limitations. First, this review was restricted to English articles. The impact of including other languages in this review is unknown, but areas with a high burden of HIV are mostly Anglophone countries. Therefore, the pattern of review findings is not expected to change. Second, the review included peer-reviewed articles. The impact of omitting grey literature is also unknown, but because cross-sectional design methods are widely used, the conclusion of this review is unlikely to be affected by the missed grey literature.

Conclusion

Most studies on the HIV cascade and continuum of care applied cross-sectional study design methods. The use of longitudinal study design methods in the assessment of the HIV cascade is increasing. These methods provide additional information about the transition dynamics along the cascade, establish detailed progress, and identify additional areas for interventions. Multistate model frameworks can be viewed as extensions of established cross-sectional design frameworks regarding engagement in HIV care, but further work is needed to integrate ART status and viral load suppression status into these frameworks. Therefore, a

For peer review only - http://bmjopen.bmj.com/site/about/guidelines.xhtml

methodological guide on the application of different types of longitudinal design methods in the HIV cascade and continuum care and their corresponding frameworks is needed to guide future studies.

For peer review only

For peer review only - http://bmjopen.bmj.com/site/about/guidelines.xhtml

Statements

Author contributions

AVK designed the study, obtained funding, conducted the review, and prepared the manuscript. KO and FV supervised the study design and implementation. TRG, MW, ASM, and HM participated in the study design and reviewed the manuscript. All authors have reviewed and approved the final manuscript.

Conflict of interest

Maja Weiser was sponsored by different sponsors to attend the ECCMID conferences during the last 10 years and she was a member of the Menari Advisory Board in 2022. All other authors declared no conflict of interest.

Funding

This research was supported by the Consortium for Advanced Research Training in Africa (CARTA). CARTA is jointly led by the African Population and Health Research Center and the University of the Witwatersrand and funded by the Carnegie Corporation of New York (Grant No. G-19-57145), Sida (Grant No:**54100113**), Uppsala Monitoring Center, Norwegian Agency for Development Cooperation (Norad), and by the Wellcome Trust [reference no. 107768/Z/15/Z] and the UK Foreign, Commonwealth & Development Office, with support from the Developing Excellence in Leadership, Training and Science in Africa (DELTAS Africa) programme. The statements made and views expressed are solely the responsibility of the Fellow.

For peer review only - http://bmjopen.bmj.com/site/about/guidelines.xhtml

Role of the funder

The funder of this systematic scoping review had no role in the review design, data extraction, synthesis of results and interpretation, preparation of the review manuscript, and the choice of journal for submission of the manuscript for publication.

Data availability

All review data extracted and processed is available upon request through the corresponding author.

Ethical approval

This study is part of the Ph.D. project that was approved by the University of the Witwatersrand Human Research Ethics Committee (Medical) with reference number R14/49, however, this study did not involve human participants.

Acknowledgments

Apatsa Selemani: Librarian and CARTA fellow at the Kamuzu University of Health Sciences, Malawi (formerly College of Medicine and Kamuzu College of Nursing of the University of Malawi), Malawi. The librarian contributed to the development of the review search strategies for various databases.

Samwel Godfrey Lwambura (Statistician and Data Officer) and Jacqueline Minja (Statistician and Research Scientist), Ifakara Health Institute, Tanzania. Research assistants assisted with full-text review, data extraction, and data entry.

Dr. Yeromin Mlacha (Medical Entomologist and Senior Researcher), Ifakara Health Institute, Tanzania, assisted in the data preparation for maps of the included articles.

For peer review only

For peer review only - http://bmjopen.bmj.com/site/about/guidelines.xhtml

References

1. Gardner EM, McLees MP, Steiner JF, del Rio C, Burman WJ. The Spectrum of Engagement in HIV Care and its Relevance to Test-and-Treat Strategies for Prevention of HIV Infection. Clin Infect Dis. 2011 Mar 15;52(6):793–800.

2. Centers for Disease Control and Prevention (CDC). Vital signs: HIV prevention through care and treatment--United States. MMWR Morb Mortal Wkly Rep. 2011 Dec 2;60(47):1618–23.

3. Gillis J, Loutfy M, Bayoumi AM, Antoniou T, Burchell AN, Walmsley S, et al. A Multi-State Model Examining Patterns of Transitioning Among States of Engagement in Care in HIV-Positive Individuals Initiating Combination Antiretroviral Therapy. J Acquir Immune Defic Syndr. 2016 Dec 15;73(5):531–9.

4. Blitz S, Antoniou T, Burchell A, Walmsley S, Light L, Gardner S, et al. The Use of Multistate Models to Examine Associations of Stress and Adherence With Transitions Among HIV Care States Observed in a Clinical HIV Cohort. J Acquir Immune Defic Syndr. 2017 01;76(3):303–10.

5. Lee H, Hogan JW, Genberg BL, Wu XK, Musick BS, Mwangi A, et al. A state transition framework for patient-level modeling of engagement and retention in HIV care using longitudinal cohort data. Statistics in Medicine. 2018;37(2):302–19.

6. Powers KA, Miller WC. Building on the HIV cascade: a complementary “HIV States and Transitions” framework for describing HIV diagnosis, care, and treatment at the population level. J Acquir Immune Defic Syndr. 2015 Jul 1;69(3):341–7.

7. 2017 HIV Continuum of Care | CDC [Internet]. 2019 [cited 2020 Mar 29]. Available from: https://www.cdc.gov/nchhstp/newsroom/2017/HIV-Continuum-of-Care.html

8. Lee H, Wu XK, Genberg BL, Mugavero MJ, Cole SR, Lau B, et al. Beyond binary retention in HIV care: predictors of the dynamic processes of patient engagement, disengagement, and re-entry into care in a US clinical cohort. AIDS. 2018 24;32(15):2217–25.

9. Barrow GJ, Brandeau ML. A modified HIV continuum of care: A six-year evaluation of a viral load cascade at a hospital-based clinic in Kingston, Jamaica. Int J STD AIDS. 2019 Jul;30(8):748–55.

10. Kay ES, Batey DS, Mugavero MJ. The HIV treatment cascade and care continuum: updates, goals, and recommendations for the future. AIDS Research and Therapy. 2016 Nov 8;13(1):35.

11. 90-90-90: An ambitious treatment target to help end the AIDS epidemic. :40.

12. prevailing-against-pandemics_en.pdf [Internet]. [cited 2021 Mar 3]. Available from: https://aidstargets2025.unaids.org/assets/images/prevailing-against-pandemics_en.pdf

13. Van Beckhoven D, Florence E, Ruelle J, Deblonde J, Verhofstede C, Callens S, et al. Good continuum of HIV care in Belgium despite weaknesses in retention and linkage to care among migrants. BMC Infect Dis. 2015 Nov 3;15(1):496.

14. Marinda E, Simbayi L, Zuma K, Zungu N, Moyo S, Kondlo L, et al. Towards achieving the 90-90-90 HIV targets: results from the south African 2017 national HIV survey. BMC Public Health. 2020 Sep 9;20(1):1375.

For peer review only

For peer review only - http://bmjopen.bmj.com/site/about/guidelines.xhtml

15. Cowan FM, Davey CB, Fearon E, Mushati P, Dirawo J, Cambiano V, et al. The HIV Care Cascade Among Female Sex Workers in Zimbabwe: Results of a Population-Based Survey From the Sisters Antiretroviral Therapy Programme for Prevention of HIV, an Integrated Response (SAPPH-IRe) Trial. J Acquir Immune Defic Syndr. 2017 Apr 1;74(4):375–82.

16. Engelhard EAN, Smit C, Van Sighem A, Reiss P, Nieuwkerk PT, Kroon FP, et al. Impact of HIV care facility characteristics on the cascade of care in HIV-infected patients in the Netherlands. AIDS. 2016 Jan;30(2):301–10.

17. Haber N, Tanser F, Bor J, Naidu K, Mutevedzi T, Herbst K, et al. From HIV infection to therapeutic response: a population-based longitudinal HIV cascade-of-care study in KwaZulu-Natal, South Africa. Lancet HIV. 2017 May;4(5):e223–30.

18. Anglemyer A, Haber N, Noiman A, Rutherford G, Ganesan A, Blaylock J, et al. HIV Care Continuum and Meeting 90-90-90 Targets: Cascade of Care Analyses of a U.S. Military Cohort. Mil Med. 2020 Aug 14;185(7–8):e1147–54.

19. Elgalib A, Shah S, Al-Wahaibi A, Al-Habsi Z, Al-Fouri M, Lau R, et al. Disparities between HIV patient subgroups in Oman: An analysis of the 2019 cascade of care. PLoS One. 2021;16(7):e0254474.

20. Vogler IH, Alfieri DF, Gianjacomo HDB, Almeida ERD de, Reiche EMV. Cascade of care for people living with HIV infection in Southern Brazil: results from a public health network. Cad Saude Publica. 2018 Nov 29;34(12):e00009718.

21. Maman D, Zeh C, Mukui I, Kirubi B, Masson S, Opolo V, et al. Cascade of HIV care and population viral suppression in a high-burden region of Kenya. AIDS. 2015 Jul 31;29(12):1557–65.

22. Lourenço L, Colley G, Nosyk B, Shopin D, Montaner JSG, Lima VD, et al. High Levels of Heterogeneity in the HIV Cascade of Care across Different Population Subgroups in British Columbia, Canada. PLOS ONE. 2014 Dec 26;9(12):e115277.

23. LESKO CR, EDWARDS JK, MOORE RD, LAU B. A longitudinal, HIV care continuum: 10-year restricted mean time in each care continuum stage after enrollment in care, by history of injection drug use. AIDS. 2016 Sep 10;30(14):2227–34.

24. Supervie V, Marty L, Lacombe JM, Dray-Spira R, Costagliola D, FHDH-ANRS CO4 study group. Looking Beyond the Cascade of HIV Care to End the AIDS Epidemic: Estimation of the Time Interval From HIV Infection to Viral Suppression. J Acquir Immune Defic Syndr. 2016 01;73(3):348–55.

25. Iroh PA, Mayo H, Nijhawan AE. The HIV Care Cascade Before, During, and After Incarceration: A Systematic Review and Data Synthesis. Am J Public Health. 2015 Jul;105(7):e5-16.

26. Stannah J, Dale E, Elmes J, Staunton R, Beyrer C, Mitchell KM, et al. HIV testing and engagement with the HIV treatment cascade among men who have sex with men in Africa: a systematic review and meta-analysis. Lancet HIV. 2019 Nov;6(11):e769–87.

27. Vagenas P, Azar MM, Copenhaver MM, Springer SA, Molina PE, Altice FL. The Impact of Alcohol Use and Related Disorders on the HIV Continuum of Care: a Systematic Review : Alcohol and the HIV Continuum of Care. Curr HIV/AIDS Rep. 2015 Dec;12(4):421–36.

For peer review only - http://bmjopen.bmj.com/site/about/guidelines.xhtml

28. Mody A, Tram KH, Glidden DV, Eshun-Wilson I, Sikombe K, Mehrotra M, et al. Novel Longitudinal Methods for Assessing Retention in Care: a Synthetic Review. Curr HIV/AIDS Rep. 2021 Aug;18(4):299–308.

29. Mohd Salleh NA, Voon P, Karamouzian M, Milloy MJ, Richardson L. Methadone maintenance therapy service components linked to improvements in HIV care cascade outcomes: A systematic review of trials and observational studies. Drug Alcohol Depend. 2021 Jan 1;218:108342.

30. Mugglin C, Kläger D, Gueler A, Vanobberghen F, Rice B, Egger M, et al. The HIV care cascade in sub-Saharan Africa: systematic review of published criteria and definitions. J Int AIDS Soc. 2021 Jul;24(7):e25761.

31. Keene CM, Ragunathan A, Euvrard J, English M, McKnight J, Orrell C, et al. Measuring patient engagement with HIV care in sub-Saharan Africa: a scoping study. Journal of the International AIDS Society. 2022;25(10):e26025.

32. Jongbloed K, Pooyak S, Sharma R, Mackie J, Pearce ME, Laliberte N, et al. Experiences of the HIV Cascade of Care Among Indigenous Peoples: A Systematic Review. AIDS Behav. 2019 Apr;23(4):984–1003.

33. Zanoni BC, Mayer KH. The adolescent and young adult HIV cascade of care in the United States: exaggerated health disparities. AIDS Patient Care STDS. 2014 Mar;28(3):128–35.

34. Bobat R, Archary M, Lawler M. An update on the HIV treatment cascade in children and adolescents. Curr Opin HIV AIDS. 2015 Nov;10(6):411–9.

35. Medland NA, McMahon JH, Chow EPF, Elliott JH, Hoy JF, Fairley CK. The HIV care cascade: a systematic review of data sources, methodology and comparability. J Int AIDS Soc. 2015;18:20634.

36. Granich R, Gupta S, Hall I, Aberle-Grasse J, Hader S, Mermin J. Status and methodology of publicly available national HIV care continua and 90-90-90 targets: A systematic review. PLoS Med. 2017 Apr;14(4):e1002253.

37. Vourli G, Katsarolis I, Pantazis N, Touloumi G. HIV continuum of care: expanding scope beyond a cross-sectional view to include time analysis: a systematic review. BMC Public Health. 2021 Sep 17;21(1):1699.

38. JBI Manual for Evidence Synthesis - JBI Global Wiki [Internet]. [cited 2022 Oct 20]. Available from: https://jbi-global-wiki.refined.site/space/MANUAL

39. Tricco AC, Lillie E, Zarin W, O'Brien KK, Colquhoun H, Levac D, et al. PRISMA Extension for Scoping Reviews (PRISMA-ScR): Checklist and Explanation. Ann Intern Med. 2018 Oct 2;169(7):467–73.

40. Caruana EJ, Roman M, Hernández-Sánchez J, Solli P. Longitudinal studies. J Thorac Dis. 2015 Nov;7(11):E537–40.

41. Gourlay AJ, Pharris AM, Noori T, Supervie V, Rosinska M, van Sighem A, et al. Towards standardized definitions for monitoring the continuum of HIV care in Europe. AIDS. 2017 Sep 24;31(15):2053–8.

For peer review only

For peer review only - http://bmjopen.bmj.com/site/about/guidelines.xhtml

42. Horberg MA, Hurley LB, Klein DB, Towner WJ, Kadlecik P, Antoniskis D, et al. The HIV Care Cascade Measured Over Time and by Age, Sex, and Race in a Large National Integrated Care System. AIDS Patient Care STDS. 2015 Nov;29(11):582–90.

43. Haber N, Pillay D, Porter K, Bärnighausen T. Constructing the cascade of HIV care: methods for measurement. Curr Opin HIV AIDS. 2016 Jan;11(1):102–8.

44. Touloumi G, Thomadakis C, Pantazis N, Papastamopoulos V, Paparizos V, Metallidis S, et al. HIV continuum of care: bridging cross-sectional and longitudinal analyses. AIDS. 2022 Mar 15;36(4):583–91.

45. Mody A, Glidden DV, Eshun-Wilson I, Sikombe K, Simbeza S, Mukamba N, et al. Longitudinal Care Cascade Outcomes Among People Eligible for Antiretroviral Therapy Who Are Newly Linking to Care in Zambia: A Multistate Analysis. Clin Infect Dis. 2020 Dec 17;71(10):e561–70.

46. Rahmalia A, Price MH, Hartantri Y, Alisjahbana B, Wisaksana R, Crevel R van, et al. Are there differences in HIV retention in care between female and male patients in Indonesia? A multi-state analysis of a retrospective cohort study. PLOS ONE. 2019 Jun 25;14(6):e0218781.

47. Wang L, Krebs E, Min JE, Mathews WC, Nijhawan A, Somboonwit C, et al. Combined estimation of disease progression and retention on antiretroviral therapy among treated individuals with HIV in the USA: a modelling study. Lancet HIV. 2019 Aug;6(8):e531–9.

48. McNairy ML, Lamb MR, Abrams EJ, Elul B, Sahabo R, Hawken MP, et al. Use of a Comprehensive HIV Care Cascade for Evaluating HIV Program Performance: Findings From 4 Sub-Saharan African Countries. J Acquir Immune Defic Syndr. 2015 Oct 1;70(2):e44–51.

49. Ehrenkranz P, Rosen S, Boulle A, Eaton JW, Ford N, Fox MP, et al. The revolving door of HIV care: Revising the service delivery cascade to achieve the UNAIDS 95-95-95 goals. PLoS Med. 2021 May;18(5):e1003651.

50. Hawk M, Maulsby C, Enobun B, Kinsky S. HIV Treatment Cascade by Housing Status at Enrollment: Results from a Retention in Care Cohort. AIDS Behav. 2019 Mar;23(3):765–75.

51. McGettrick P, Ghavami-Kia B, Tinago W, Macken A, O'Halloran J, Lambert JS, et al. The HIV Care Cascade and sub-analysis of those linked to but not retained in care: the experience from a tertiary HIV referral service in Dublin Ireland. HIV Clin Trials. 2017 May;18(3):93–9.

52. Comelli A, Izzo I, Donato F, Celotti A, Focà E, Pezzoli C, et al. Disengagement and reengagement of HIV continuum of care in a single center cohort in northern Italy. HIV Res Clin Pract. 2019;20(1):1–11.

53. Boothe MAS, Sathane I, Baltazar CS, Chicuecue N, Horth R, Fazito E, et al. Low engagement in HIV services and progress through the treatment cascade among key populations living with HIV in Mozambique: alarming gaps in knowledge of status. BMC Public Health. 2021 Jan 15;21(1):146.

54. Zhang N, Bussell S, Wang G, Zhu X, Yang X, Huang T, et al. Disparities in HIV Care Along the Path From Infection to Viral Suppression: A Cross-sectional Study of HIV/AIDS Patient Records in 2013, Shandong Province, China. Clin Infect Dis. 2016 Jul 1;63(1):115–21.

For peer review only - http://bmjopen.bmj.com/site/about/guidelines.xhtml

55. Zhang J, Xu JJ, Song W, Pan S, Chu ZX, Hu QH, et al. HIV Incidence and Care Linkage among MSM First-Time-Testers in Shenyang, China 2012-2014. AIDS Behav. 2018 Mar;22(3):711–21.

56. Kerrigan D, Mbwambo J, Likindikoki S, Davis W, Mantsios A, Beckham SW, et al. Project Shikamana: Community Empowerment-Based Combination HIV Prevention Significantly Impacts HIV Incidence and Care Continuum Outcomes Among Female Sex Workers in Iringa, Tanzania. J Acquir Immune Defic Syndr. 2019 Oct 1;82(2):141–8.

57. Mehta SH, Lucas GM, Solomon S, Srikrishnan AK, McFall AM, Dhingra N, et al. HIV care continuum among men who have sex with men and persons who inject drugs in India: barriers to successful engagement. Clin Infect Dis. 2015 Dec 1;61(11):1732–41.

58. Philbin MM, Feaster DJ, Gooden L, Duan R, Das M, Jacobs P, et al. The North-South Divide: Substance Use Risk, Care Engagement, and Viral Suppression Among Hospitalized Human Immunodeficiency Virus-Infected Patients in 11 US Cities. Clin Infect Dis. 2019 Jan 1;68(1):146–9.

59. Zulliger R, Barrington C, Donastorg Y, Perez M, Kerrigan D. High Drop-off Along the HIV Care Continuum and ART Interruption Among Female Sex Workers in the Dominican Republic. J Acquir Immune Defic Syndr. 2015 Jun 1;69(2):216–22.

60. Lippman SA, Shade SB, El Ayadi AM, Gilvydis JM, Grignon JS, Liegler T, et al. Attrition and Opportunities Along the HIV Care Continuum: Findings From a Population-Based Sample, North West Province, South Africa. J Acquir Immune Defic Syndr. 2016 01;73(1):91–9.

61. Alvarez-Uria G, Pakam R, Midde M, Naik PK. Entry, Retention, and Virological Suppression in an HIV Cohort Study in India: Description of the Cascade of Care and Implications for Reducing HIV-Related Mortality in Low- and Middle-Income Countries. Interdiscip Perspect Infect Dis. 2013;2013:384805.

62. Marsh K, Eaton JW, Mahy M, Sabin K, Autenrieth CS, Wanyeki I, et al. Global, regional and country-level 90-90-90 estimates for 2018: assessing progress towards the 2020 target. AIDS. 2019 Dec 15;33 Suppl 3:S213–26.

63. Mangal JP, Rimland D, Marconi VC. The Continuum of HIV Care in a Veterans' Affairs Clinic. AIDS Research and Human Retroviruses. 2014 Jan 8;30(5):409–15.

64. Elgalib A, Shah S, Al-Habsi Z, Al-Fouri M, Lau R, Al-Kindi H, et al. The cascade of HIV care in Oman, 2015-2018: A population-based study from the Middle East. Int J Infect Dis. 2020 Jan;90:28–34.

65. Hightow-Weidman L, LeGrand S, Choi SK, Egger J, Hurt CB, Muessig KE. Exploring the HIV continuum of care among young black MSM. PLoS One. 2017;12(6):e0179688.

66. Ross J, Felsen UR, Cunningham CO, Patel VV, Hanna DB. Outcomes Along the HIV Care Continuum Among Undocumented Immigrants in Clinical Care. AIDS Res Hum Retroviruses. 2017 Oct;33(10):1038–44.

67. Jin H, Ogunbajo A, Mimiaga MJ, Duncan DT, Boyer E, Chai P, et al. Over the influence: The HIV care continuum among methamphetamine-using men who have sex with men. Drug Alcohol Depend. 2018 Nov 1;192:125–8.

For peer review only - http://bmjopen.bmj.com/site/about/guidelines.xhtml

68. Lancaster KE, Powers KA, Lungu T, Mmodzi P, Hosseinipour MC, Chadwick K, et al. The HIV Care Continuum among Female Sex Workers: A Key Population in Lilongwe, Malawi. PLoS One. 2016;11(1):e0147662.

69. Xia Q, Shah D, Gill B, Torian LV, Braunstein SL. Continuum of Care Among People Living with Perinatally Acquired HIV Infection in New York City, 2014. Public Health Rep. 2016;131(4):566–73.

70. Helleberg M, Häggblom A, Sönnerborg A, Obel N. HIV care in the Swedish-Danish HIV cohort 1995-2010, closing the gaps. PLoS One. 2013;8(8):e72257.

71. Kapogiannis BG, Koenig LJ, Xu J, Mayer KH, Loeb J, Greenberg L, et al. The HIV Continuum of Care for Adolescents and Young Adults Attending 13 Urban US HIV Care Centers of the NICHD-ATN-CDC-HRSA SMILE Collaborative. J Acquir Immune Defic Syndr. 2020 May 1;84(1):92–100.

72. Matson TE, McGinnis KA, Rubinsky AD, Frost MC, Czarnogorski M, Bryant KJ, et al. Gender and alcohol use: influences on HIV care continuum in a national cohort of patients with HIV. AIDS. 2018 Sep 24;32(15):2247–53.

73. Genberg BL, Naanyu V, Wachira J, Hogan JW, Sang E, Nyambura M, et al. Linkage to and engagement in HIV care in western Kenya: an observational study using population-based estimates from home-based counselling and testing. Lancet HIV. 2015 Jan;2(1):e20-26.

74. Meira-Machado L, de Uña-Álvarez J, Cadarso-Suárez C, Andersen PK. Multi-state models for the analysis of time-to-event data. Stat Methods Med Res. 2009 Apr 1;18(2):195–222.

75. Andersen PK, Pohar Perme M. Inference for outcome probabilities in multi-state models. Lifetime Data Anal. 2008 Sep 13;14(4):405.

For peer review only

For peer review only - http://bmjopen.bmj.com/site/about/guidelines.xhtml

658 **Figure captions**

659 **Figure 1:** Flow chart of the inclusion and exclusion of research articles

660 **Figure 2:** The geographical location distribution of country-specific studies of cross-sectional (n=252)
661 and longitudinal studies (n=20) (regional studies, such as sub-Saharan Africa or Asia and Europe, were
662 excluded)

663

For peer review only

For peer review only - http://bmjopen.bmj.com/site/about/guidelines.xhtml

Figure 1

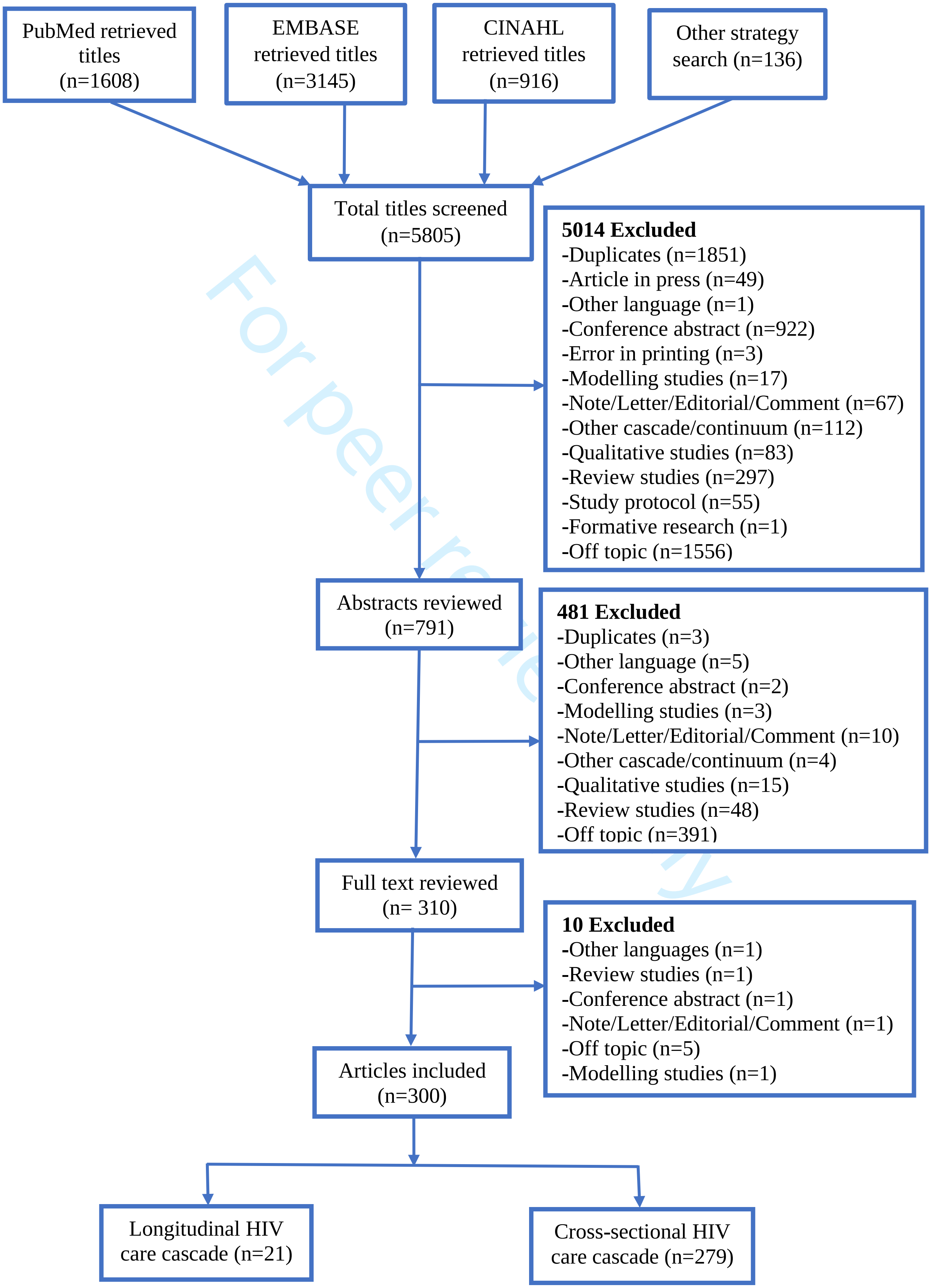

For peer review only

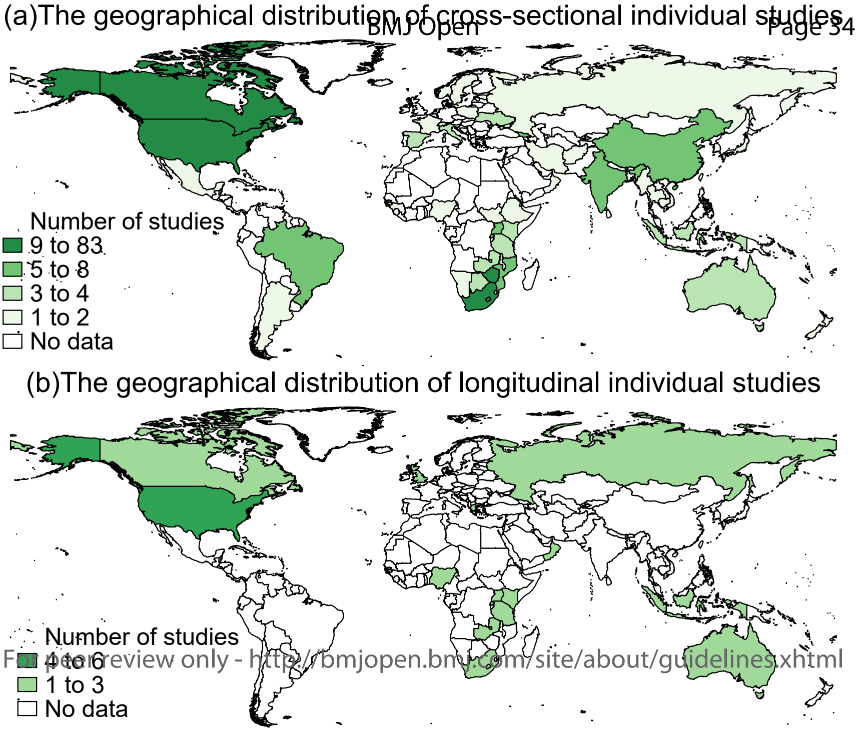
(a)The geographical distribution of cross-sectional individual studies
Number of studies
9 to 83
5 to 8
3 to 4
1 to 2
No data
(b)The geographical distribution of longitudinal individual studies
Number of studies
4 to 6
1 to 3
No data

Supplementary material

Table S1: Database search queries

Database	Query number	Query	Number of articles
PubMed (Searched on 05-04-2022)	1	((HIV) AND ((Care cascade) OR (cascade of care) OR (UNAIDS 90-90-90) OR (continuum of care[Title/Abstract]))) **OR** (((HIV) AND ((Care cascade) OR (UNAIDS 90-90-90) OR (cascade of care) OR (continuum of care[Title/Abstract]))) AND ((Multistate models[Title/Abstract]) OR (longitudinal[Title/Abstract]) OR (Markov models[Title/Abstract]) OR (Multi-state models[Title/Abstract])))	1,608
CINAHL Complete (04-07-2022)	S1	MH human immunodeficiency virus or hiv	32,691
	S2	MH hiv infection	945
	S3	TI (HIV infection or Human immunodeficiency virus or HIV) OR AB (HIV infection or Human immunodeficiency virus or HIV)	104,219
	S4	S1 OR S2 OR S3	106,982
	S5	MH care cascade	1,284
	S6	MH continuum of care	7,104
	S7	MH 90-90-90	17,568
	S8	TI (care cascade or cascade of care or continuum of care or "care cascade progress" or UNAIDS 90-90-90 progress" or UNAIDS 90-90-90 or "cross-sectional care cascade") OR AB (care cascade or cascade of care or continuum of care or "care cascade progress" or UNAIDS 90-90-90 progress" or UNAIDS 90-90-90 or "cross-sectional care cascade")	5,497
	S9	S5 OR S6 OR S7 OR S8	6,288
	S10	S4 AND S9	916
	S11	MH Multistate models	293
	S12	MH Multi-state models	1,763
	S13	MH Markov models	2,000
	S14	TI (Multistate models or Multi-state models or Markov models or "longitudinal care cascade") OR AB (Multistate models or Multi-state models or Markov models or "longitudinal care cascade")	3,925
	S15	S11 OR S12 OR S13 OR S14	3,992
	S16	S10 AND S15	5
	S17	S10 OR S16	916
EMBASE (Search date 15-07-2022)	#1	'human immunodeficiency virus'/exp OR 'human immunodeficiency virus infection'/exp OR 'human immunodeficiency virus':ti,ab,kw OR 'human immunodeficiency virus infection':ti,ab,kw OR hiv:ti,ab,kw OR 'hiv infection':ti,ab,kw	601495
	#2	'cascade of care'/exp OR 'continuum of care'/exp OR 'care continuum':ti,ab,kw OR '90 90 90':ti,ab,kw OR 'care cascade':ti,ab,kw OR 'cascade of care':ti,ab,kw OR 'continuum of care':ti,ab,kw OR 'care cascade progress':ti,ab,kw OR 'unaids 90-90-90 progress':ti,ab,kw	9220

For peer review only - http://bmjopen.bmj.com/site/about/guidelines.xhtml

		OR 'unaids 90-90-90':ti,ab,kw OR 'cross-sectional care cascade':ti,ab,kw	
	#3	#1 AND #2	3145
	#4	'multistate model'/exp OR 'multistate model' OR 'multi state model'/exp OR 'multi state model' OR 'markov chain'/exp OR 'markov chain' OR 'multistate models':ti,ab,kw OR 'multi state models':ti,ab,kw OR 'markov models':ti,ab,kw OR 'longitudinal':ti,ab,kw	429711
	#5	#3 AND #4	171
	#6	#3 OR #5	3145
Other strategic searches	PubMed and Google scholar search using individual key works such as "HIV care cascade" or "HIV care cascade and multistate models" or "HIV care cascade and multi-state models" or "HIV care cascade and Markov models". Bibliographic list of the articles.		136

For peer review only - http://bmjopen.bmj.com/site/about/guidelines.xhtml

Table S2: Data extraction tool for the scoping review

Information extracted from articles	Possible responses
Paper ID number	
Authors	
Study site in terms of country	
The specific study site of the research article	
The level of study representation	
The study design	Cross-sectional, Case-Control, Longitudinal, Cohort, Prospective cohort, Retrospective cohort, Clinical trial, Not stated in the article, Other
Other study design not listed above	
Data sources for the studies reviewed	Cohort data, Laboratory test data (including CD4 and viral load tests), Medical records from the hospital, Survey data, Simulated data (mathematical modeling), Surveillances, Surveillances, Others
Specify other data sources not listed in the list above	
Number of patients/participants included in the final analysis of the study	
The actual age of participants included in the study	
Categories of the age of participants included in the study	All (children and adults) PLHIV, Adults (≥18 years), Adults (≥15 years), Adults (≥16 years), Adolescents and adults (>=13 years), Adolescents and adults (>=12 years), Adolescents (10-19 years), Neonates
Type of participants included in a research article	All (mixture) PLHIV, Children only, Adolescents, Adults, Key populations, Migrants, Prisoners, Veterans, Refugees, Military
Key population, please specify	Men having sex with men, Female sex workers, Injected drug users, Transgender women, Transgender men
Cascade size	Full cascade stages, More than full cascade (> 6 stages), Medium cascade stages, Small to very small cascade stages, Other
Specify other study outcomes	
Number of the cascade stages	
Actual stage name	
stage 1	
stage 2	
stage 3	
stage 4	
stage 5	
stage 6	
stage 7	
stage 8	
stage 9	
Absorbing state	Death, LTFU, Transfer, Other
Specify other absorbing states	

For peer review only - http://bmjopen.bmj.com/site/about/guidelines.xhtml

Information extracted from articles	Possible responses
The type of multistate model used	Non-Parametric multistate model, Parametric, continuous-time multistate Markov model, Time-homogeneous Markov models, Discrete-time multistate Markov model, General multistate model, Other
Specify other types of multistate models used	
Multistate model assumptions	
Assessment starting point (baseline)	Enrolment/recruitment date, ART start date
Reason for censoring	
Descriptions of statistics used	Prevalence, Incidence rates, Proportion for each stage of HIV care cascade, Chi-square test of independence, Fisher Exact test (small sample size), t-test, Wilcoxon rank test, Time spent in each cascade stage, Transition probabilities, Mean, Standard deviation, Median, Interquartile range
Type of regression models used	Logistics regression, Cox PH regression, Poisson regression, competing risk regression, Generalized linear regression model (GLM), Weighted linear regression, Mathematical back-calculation, Log-Binomial regression, Generalized linear mixed models (GLMM), Multinomial regression model, Other
Other regression models, specify	
The method used for handling missing data	Weighted analysis, Multiple imputations, Missing data indicator, Not addressed
Methods used for assessing the goodness of fit of the model	Pearson-type Chi-Square test, Visual comparison of observed and fitted value plots, Not assessed, Other
Other methods for assessing the goodness of fit of the model	
Broad categories of approaches used in research articles	Cross-sectional, Longitudinal
Specification of the cascade staging method	Yes, no
Cascade staging method	Dependent, independent, and both dependent and independent

For peer review only - http://bmjopen.bmj.com/site/about/guidelines.xhtml

Table S3: List of articles included

Paper Number	Authors	Reference
1	Lippman et al 2016	Lippman, S. A., Shade, S. B., El Ayadi, A. M., Gilvydis, J. M., Grignon, J. S., Liegler, T., Morris, J., Naidoo, E., Prach, L. M., Puren, A., & Barnhart, S. (2016). Attrition and Opportunities Along the HIV Care Continuum: Findings From a Population-Based Sample, North West Province, South Africa. Journal of acquired immune deficiency syndromes (1999), 73(1), 91–99. https://doi.org/10.1097/QAI.0000000000001026
2	Supervie et al 2016	Supervie, V., Marty, L., Lacombe, J. M., Dray-Spira, R., Costagliola, D., & FHDH-ANRS CO4 study group (2016). Looking Beyond the Cascade of HIV Care to End the AIDS Epidemic: Estimation of the Time Interval From HIV Infection to Viral Suppression. Journal of acquired immune deficiency syndromes (1999), 73(3), 348–355. https://doi.org/10.1097/QAI.0000000000001120
3	CDC 2011	Centers for Disease Control and Prevention (CDC) (2011). Vital signs: HIV prevention through care and treatment--United States. MMWR. Morbidity and mortality weekly report, 60(47), 1618–1623.
4	Nosyk et al 2014	Nosyk, B., Montaner, J. S. G., Colley, G., Lima, V. D., Chan, K., Heath, K., Yip, B., Samji, H., Gilbert, M., Barrios, R., Gustafson, R., Hogg, R. S., & STOP HIV/AIDS Study Group (2014). The cascade of HIV care in British Columbia, Canada, 1996-2011: a population-based retrospective cohort study. The Lancet. Infectious diseases, 14(1), 40–49. https://doi.org/10.1016/S1473-3099(13)70254-8
5	Kohler et al 2015	Kohler, P., Schmidt, A. J., Cavassini, M., Furrer, H., Calmy, A., Battegay, M., Bernasconi, E., Ledergerber, B., Vernazza, P., & Swiss HIV Cohort Study (2015). The HIV care cascade in Switzerland: reaching the UNAIDS/WHO targets for patients diagnosed with HIV. AIDS (London, England), 29(18), 2509–2515. https://doi.org/10.1097/QAD.0000000000000878
6	Maman et al 2015	Maman, D., Zeh, C., Mukui, I., Kirubi, B., Masson, S., Opolo, V., Szumilin, E., Riche, B., & Etard, J. F. (2015). Cascade of HIV care and population viral suppression in a high-burden region of Kenya. AIDS (London, England), 29(12), 1557–1565. https://doi.org/10.1097/QAD.0000000000000741
7	Takuva et al 2017	Takuva, S., Brown, A. E., Pillay, Y., Delpech, V., & Puren, A. J. (2017). The continuum of HIV care in South Africa: implications for achieving the second and third UNAIDS 90-90-90 targets. AIDS (London, England), 31(4), 545–552. https://doi.org/10.1097/QAD.0000000000001340
8	Mangal et al 2014	Mangal, J. P., Rimland, D., & Marconi, V. C. (2014). The continuum of HIV care in a Veterans' Affairs clinic. AIDS research and human retroviruses, 30(5), 409–415. https://doi.org/10.1089/aid.2013.0232

For peer review only - http://bmjopen.bmj.com/site/about/guidelines.xhtml

Paper Number	Authors	Reference
9	Wilton et al 2019	Wilton, J., Liu, J., Sullivan, A., Rachlis, B., Marchand-Austin, A., Giles, M., Light, L., Rank, C., Burchell, A. N., Gardner, S., Sider, D., Gilbert, M., Kroch, A. E., & Ontario HIV Epidemiology and Surveillance Initiative (2019). Trends in HIV care cascade engagement among diagnosed people living with HIV in Ontario, Canada: A retrospective, population-based cohort study. PloS one, 14(1), e0210096. https://doi.org/10.1371/journal.pone.0210096
10	Kerkerian et al 2018	Kerkerian, G., Kestler, M., Carter, A., Wang, L., Kronfli, N., Sereda, P., Roth, E., Milloy, M. J., Pick, N., Money, D., Webster, K., Hogg, R. S., de Pokomandy, A., Loutfy, M., & Kaida, A. (2018). Attrition Across the HIV Cascade of Care Among a Diverse Cohort of Women Living With HIV in Canada. Journal of acquired immune deficiency syndromes (1999), 79(2), 226–236. https://doi.org/10.1097/QAI.0000000000001775
11	Lourenço et al 2014	Lourenço, L., Colley, G., Nosyk, B., Shopin, D., Montaner, J. S., Lima, V. D., & STOP HIV/AIDS Study Group (2014). High levels of heterogeneity in the HIV cascade of care across different population subgroups in British Columbia, Canada. PloS one, 9(12), e115277. https://doi.org/10.1371/journal.pone.0115277
12	Marsh et al 2019	Marsh, K., Eaton, J. W., Mahy, M., Sabin, K., Autenrieth, C. S., Wanyeki, I., Daher, J., & Ghys, P. D. (2019). Global, regional and country-level 90-90-90 estimates for 2018: assessing progress towards the 2020 target. AIDS (London, England), 33 Suppl 3(Suppl 3), S213–S226. https://doi.org/10.1097/QAD.0000000000002355
13	Muddu et al 2019	Muddu, M., Tusubira, A. K., Sharma, S. K., Akiteng, A. R., Ssinabulya, I., & Schwartz, J. I. (2019). Integrated Hypertension and HIV Care Cascades in an HIV Treatment Program in Eastern Uganda: A Retrospective Cohort Study. Journal of acquired immune deficiency syndromes (1999), 81(5), 552–561. https://doi.org/10.1097/QAI.0000000000002067
14	Nom et al 2020	Nom, N. A. M., Kyaw, K. W. Y., Kumar, A. M. V., Hone, S., Thida, T., Nwe, T. W., Soan, P., Htun, T., & Oo, H. N. (2020). HIV Care Cascade among Prisoners of the Mandalay Central Prison in Myanmar: 2011-2018. Tropical medicine and infectious disease, 5(1), 4. https://doi.org/10.3390/tropicalmed5010004
15	Stevenson et al 2020	Stevenson, K. A., Podewils, L. J., Zishiri, V. K., Castro, K. G., & Charalambous, S. (2020). HIV prevalence and the cascade of care in five South African correctional facilities. PloS one, 15(7), e0235178. https://doi.org/10.1371/journal.pone.0235178
16	McClarty et al 2021	McClarty, L. M., Kasper, K., Ireland, L., Loeppky, C., Blanchard, J. F., & Becker, M. L. (2021). The HIV care cascade in Manitoba, Canada: Methods, measures, and estimates to meet local needs. Journal of clinical

For peer review only - http://bmjopen.bmj.com/site/about/guidelines.xhtml

Paper Number	Authors	Reference
		epidemiology, 132, 26–33. https://doi.org/10.1016/j.jclinepi.2020.11.026
17	Shanaube et al 2020	Shanaube, K., Macleod, D., Chaila, M. J., Mackworth-Young, C., Hoddinott, G., Schaap, A., Floyd, S., Bock, P., Hayes, R., Fidler, S., & Ayles, H. (2021). HIV Care Cascade Among Adolescents in a "Test and Treat" Community-Based Intervention: HPTN 071 (PopART) for Youth Study. The Journal of adolescent health : official publication of the Society for Adolescent Medicine, 68(4), 719–727. https://doi.org/10.1016/j.jadohealth.2020.07.029
18	Burgos-soto et al 2020	Burgos-Soto, J., Ben Farhat, J., Alley, I., Ojuka, P., Mulogo, E., Kise-Sete, T., Bouhenia, M., Salumu, L., Mathela, R., Langendorf, C., Cohuet, S., & Huerga, H. (2020). HIV epidemic and cascade of care in 12 east African rural fishing communities: results from a population-based survey in Uganda. BMC public health, 20(1), 970. https://doi.org/10.1186/s12889-020-09121-6
19	Rwema et al 2020	Twahirwa Rwema, J. O., Lyons, C. E., Herbst, S., Liestman, B., Nyombayire, J., Ketende, S., Mazzei, A., Olawore, O., Nsanzimana, S., Mugwaneza, P., Kagaba, A., Sullivan, P. S., Allen, S., Karita, E., & Baral, S. D. (2020). HIV infection and engagement in HIV care cascade among men who have sex with men and transgender women in Kigali, Rwanda: a cross-sectional study. Journal of the International AIDS Society, 23 Suppl 6(Suppl 6), e25604. https://doi.org/10.1002/jia2.25604
20	Fern'andez-Luis et al 2020	Fernández-Luis, S., Nhampossa, T., Fuente-Soro, L., Augusto, O., Casellas, A., Bernardo, E., Ruperez, M., Gonzalez, R., Maculuve, S., Saura-Lázaro, A., Menendez, C., Naniche, D., & Lopez-Varela, E. (2020). Pediatric HIV Care Cascade in Southern Mozambique: Missed Opportunities for Early ART and Re-engagement in Care. The Pediatric infectious disease journal, 39(5), 429–434. https://doi.org/10.1097/INF.0000000000002612
21	Marukutira et al 2020	Marukutira, T., Gray, R. T., Douglass, C., El-Hayek, C., Moreira, C., Asselin, J., Donovan, B., Vickers, T., Spelman, T., Crowe, S., Guy, R., Stoove, M., & Hellard, M. (2020). Gaps in the HIV diagnosis and care cascade for migrants in Australia, 2013-2017: A cross-sectional study. PLoS medicine, 17(3), e1003044. https://doi.org/10.1371/journal.pmed.1003044
22	Conan et al 2021	Conan, N., Paye, C. P., Ortuno, R., Chijuwa, A., Chiwandira, B., Goemaere, E., Belen Garone, D., Coulborn, R. M., Chihana, M., & Maman, D. (2021). What gaps remain in the HIV cascade of care? Results of a population-based survey in Nsanje District, Malawi. PloS one, 16(4), e0248410. https://doi.org/10.1371/journal.pone.0248410

For peer review only - http://bmjopen.bmj.com/site/about/guidelines.xhtml

Paper Number	Authors	Reference
23	Elgalib et al 2021	Elgalib, A., Shah, S., Al-Wahaibi, A., Al-Habsi, Z., Al-Fouri, M., Lau, R., Al-Kindi, H., Al-Rawahi, B., & Al-Abri, S. (2021). Disparities between HIV patient subgroups in Oman: An analysis of the 2019 cascade of care. PloS one, 16(7), e0254474. https://doi.org/10.1371/journal.pone.0254474
24	Januraga et al 2018	Januraga, P. P., Reekie, J., Mulyani, T., Lestari, B. W., Iskandar, S., Wisaksana, R., Kusmayanti, N. A., Subronto, Y. W., Widyanthini, D. N., Wirawan, D. N., Wongso, L. V., Sudewo, A. G., Sukmaningrum, E., Nisa, T., Prabowo, B. R., Law, M., Cooper, D. A., & Kaldor, J. M. (2018). The cascade of HIV care among key populations in Indonesia: a prospective cohort study. The lancet. HIV, 5(10), e560–e568. https://doi.org/10.1016/S2352-3018(18)30148-6
26	Hawk et al 2019	Hawk, M., Maulsby, C., Enobun, B., Kinsky, S., & AIDS United Retention in Care Intervention Team (2019). HIV Treatment Cascade by Housing Status at Enrollment: Results from a Retention in Care Cohort. AIDS and behavior, 23(3), 765–775. https://doi.org/10.1007/s10461-018-2295-y
27	McAllister et al 2020	McAllister, S., van Asten, H., Anglemyer, A., Crengle, S., Zeng, J., Raymond, N., Handy, R., Giola, M., Dickson, N., & Priest, P. (2021). Cascade of care of people diagnosed with HIV in New Zealand between 2006 and 2017. HIV medicine, 22(2), 122–130. https://doi.org/10.1111/hiv.12983
28	Volger et al 2018	Vogler, I. H., Alfieri, D. F., Gianjacomo, H. D. B., Almeida, E. R. D., & Reiche, E. M. V. (2018). Cascade of care for people living with HIV infection in Southern Brazil: results from a public health network. Cadernos de saude publica, 34(12), e00009718. https://doi.org/10.1590/0102-311X00009718
29	Sazonova et al 2020	Sazonova, Y., Kulchynska, R., Sereda, Y., Azarskova, M., Novak, Y., Saliuk, T., Kornilova, M., Liulchuk, M., Vitek, C., & Dumchev, K. (2020). HIV treatment cascade among people who inject drugs in Ukraine. PloS one, 15(12), e0244572. https://doi.org/10.1371/journal.pone.0244572
30	Puryear et al 2020	Puryear, S. B., Balzer, L. B., Ayieko, J., Kwarisiima, D., Hahn, J. A., Charlebois, E. D., Clark, T. D., Cohen, C. R., Bukusi, E. A., Kamya, M. R., Petersen, M. L., Havlir, D. V., & Chamie, G. (2020). Associations between alcohol use and HIV care cascade outcomes among adults undergoing population-based HIV testing in East Africa. AIDS (London, England), 34(3), 405–413. https://doi.org/10.1097/QAD.0000000000002427
31	Ho et al 2019	Ho, Z. J. M., Huang, F., Wong, C. S., Chua, L., Ma, S., Chen, M. I., & Lee, V. J. (2019). Using a HIV registry to develop accurate estimates for the HIV care cascade - the Singapore experience. Journal of the International AIDS Society, 22(7), e25356. https://doi.org/10.1002/jia2.25356

For peer review only - http://bmjopen.bmj.com/site/about/guidelines.xhtml

Paper Number	Authors	Reference
32	Manne-Goehler et al 2019	Manne-Goehler, J., Siedner, M. J., Montana, L., Harling, G., Geldsetzer, P., Rohr, J., Gómez-Olivé, F. X., Goehler, A., Wade, A., Gaziano, T., Kahn, K., Davies, J. I., Tollman, S., & Bärnighausen, T. W. (2019). Hypertension and diabetes control along the HIV care cascade in rural South Africa. Journal of the International AIDS Society, 22(3), e25213. https://doi.org/10.1002/jia2.25213
33	Fuente-Soro et al 2019	Fuente-Soro, L., Iniesta, C., López-Varela, E., Cuna, M., Guilaze, R., Maixenchs, M., Bernardo, E. L., Augusto, O., Gonzalez, R., Couto, A., Munguambe, K., & Naniche, D. (2019). Tipping the balance towards long-term retention in the HIV care cascade: A mixed methods study in southern Mozambique. PloS one, 14(9), e0222028. https://doi.org/10.1371/journal.pone.0222028
34	Edun et al 2016	Edun, B., Iyer, M., Albrecht, H., & Weissman, S. (2017). The South Carolina rural-urban HIV continuum of care. AIDS care, 29(7), 817–822. https://doi.org/10.1080/09540121.2016.1270397
35	O'laughlin et al 2017	O'Laughlin, K. N., Kasozi, J., Rabideau, D. J., Parker, R. A., Mulogo, E., Faustin, Z. M., Greenwald, K. E., Doraiswamy, S., Walensky, R. P., & Bassett, I. V. (2017). The cascade of HIV care among refugees and nationals in Nakivale Refugee Settlement in Uganda. HIV medicine, 18(7), 513–518. https://doi.org/10.1111/hiv.12476
37	Barrow et al 2019	Barrow, G. J., & Brandeau, M. L. (2019). A modified HIV continuum of care: A six-year evaluation of a viral load cascade at a hospital-based clinic in Kingston, Jamaica. International journal of STD & AIDS, 30(8), 748–755. https://doi.org/10.1177/0956462419839514
38	Ikeda et al 2018	Ikeda, D. J., Hollander, L., Weigl, S., Sawicki, S. V., Belanger, D. R., West, N. Y., Brey Magnani, N., Wells, C. G., Gordon, P., Morne, J., & Agins, B. D. (2018). The Facility-Level HIV Treatment Cascade: Using a Population Health Tool in Health Care Facilities to End the Epidemic in New York State. Open forum infectious diseases, 5(10), ofy254. https://doi.org/10.1093/ofid/ofy254
39	Keen et al 2018	Keen, P., Gray, R. T., Telfer, B., Guy, R., Schmidt, H. M., Whittaker, B., Holden, J., Holt, M., Kelleher, A., Wilson, D., Callander, D., Cooper, D. A., Prestage, G., Selvey, C., Grulich, A. E., & NSW HIV Prevention Partnership Project (2018). The 2016 HIV diagnosis and care cascade in New South Wales, Australia: meeting the UNAIDS 90-90-90 targets. Journal of the International AIDS Society, 21(4), e25109. https://doi.org/10.1002/jia2.25109
40	Kowalska et al 2018	Kowalska, J. D., Ankiersztejn-Bartczak, M., Shepherd, L., & Mocroft, A. (2018). Cascade of care and factors associated with virological suppression among HIV-positive persons linked to care in the Test and Keep in Care (TAK) project. Infection, 46(4), 533–540. https://doi.org/10.1007/s15010-018-1154-0

For peer review only - http://bmjopen.bmj.com/site/about/guidelines.xhtml

Paper Number	Authors	Reference
41	Haghighat et al 2021	Haghighat, R., Toska, E., Bungane, N., & Cluver, L. (2021). The HIV care cascade for adolescents initiated on antiretroviral therapy in a health district of South Africa: a retrospective cohort study. BMC infectious diseases, 21(1), 60. https://doi.org/10.1186/s12879-020-05742-9
42	Zaller et al 2017	Zaller, N., Gordon, M., Bazerman, L., Kuo, I., & Beckwith, C. (2017). The HIV Care Cascade Among Individuals Under Community Supervision in Baltimore, Maryland. Journal of correctional health care : the official journal of the National Commission on Correctional Health Care, 23(3), 305–312. https://doi.org/10.1177/1078345817709285
43	Larmarange et al 2018	Larmarange, J., Diallo, M. H., McGrath, N., Iwuji, C., Plazy, M., Thiébaut, R., Tanser, F., Bärnighausen, T., Pillay, D., Dabis, F., Orne-Gliemann, J., & ANRS 12249 TasP Study Group (2018). The impact of population dynamics on the population HIV care cascade: results from the ANRS 12249 Treatment as Prevention trial in rural KwaZulu-Natal (South Africa). Journal of the International AIDS Society, 21 Suppl 4(Suppl Suppl 4), e25128. https://doi.org/10.1002/jia2.25128
44	Idrisov et al 2017	Idrisov, B., Lunze, K., Cheng, D. M., Blokhina, E., Gnatienko, N., Quinn, E., Bridden, C., Walley, A. Y., Bryant, K. J., Lioznov, D., Krupitsky, E., & Samet, J. H. (2017). Role of substance use in HIV care cascade outcomes among people who inject drugs in Russia. Addiction science & clinical practice, 12(1), 30. https://doi.org/10.1186/s13722-017-0098-5
45	Engelhard et al 2016	Engelhard, E. A., Smit, C., Van Sighem, A., Reiss, P., Nieuwkerk, P. T., Kroon, F. P., Brinkman, K., & Geerlings, S. E. (2016). Impact of HIV care facility characteristics on the cascade of care in HIV-infected patients in the Netherlands. AIDS (London, England), 30(2), 301–310. https://doi.org/10.1097/QAD.0000000000000938
46	Campbell et al 2015	Campbell, C. N., Ambrosioni, J., Miro, J. M., Esteve, A., Casabona, J., Navarro, G., García, I., Ferrer, E., Force, L., & Tural, C. (2015). The continuum of HIV care in Catalonia. AIDS care, 27(12), 1449–1454. https://doi.org/10.1080/09540121.2015.1109584
47	Rocha et al 2020	Rocha, A. B. M. D., Barros, C., Generoso, I. P., Bastos, F. I., & Veras, M. A. (2020). HIV continuum of care among trans women and travestis living in São Paulo, Brazil. Revista de saude publica, 54, 118. https://doi.org/10.11606/s1518-8787.2020054002374
48	Goldenberg et al 2019	Goldenberg, S. M., Muzaaya, G., Akello, M., Braschel, M., Birungi, J., & Shannon, K. (2019). High burden of previously undiagnosed HIV infections and gaps in HIV care cascade for conflict-affected female sex workers in northern Uganda. International journal of STD & AIDS, 30(3), 275–283. https://doi.org/10.1177/0956462418804658

For peer review only - http://bmjopen.bmj.com/site/about/guidelines.xhtml

Paper Number	Authors	Reference
49	Raymond et al 2016	Raymond, N., Bargh, K., Aung, K. L., & Rice, J. (2016). Cascade of care for people living with HIV infection in the Wellington region. The New Zealand medical journal, 129(1432), 41–51.
50	McNairy et al 2015	McNairy, M. L., Lamb, M. R., Abrams, E. J., Elul, B., Sahabo, R., Hawken, M. P., Mussa, A., Zwede, A., Justman, J., El-Sadr, W. M., & Identifying Optimal Models of HIV Care and Treatment in Sub-Saharan Africa Study (2015). Use of a Comprehensive HIV Care Cascade for Evaluating HIV Program Performance: Findings From 4 Sub-Saharan African Countries. Journal of acquired immune deficiency syndromes (1999), 70(2), e44–e51. https://doi.org/10.1097/QAI.0000000000000745
51	Lippman et al 2019	Lippman, S. A., El Ayadi, A. M., Grignon, J. S., Puren, A., Liegler, T., Venter, W. D. F., Ratlhagana, M. J., Morris, J. L., Naidoo, E., Agnew, E., Barnhart, S., & Shade, S. B. (2019). Improvements in the South African HIV care cascade: findings on 90-90-90 targets from successive population-representative surveys in North West Province. Journal of the International AIDS Society, 22(6), e25295. https://doi.org/10.1002/jia2.25295
52	McGettrick et al 2017	McGettrick, P., Ghavami-Kia, B., Tinago, W., Macken, A., O'Halloran, J., Lambert, J. S., Sheehan, G., & Mallon, P. W. G. (2017). The HIV Care Cascade and sub-analysis of those linked to but not retained in care: the experience from a tertiary HIV referral service in Dublin Ireland. HIV clinical trials, 18(3), 93–99. https://doi.org/10.1080/15284336.2017.1298317
53	Castro et al 2016	Castro, R., Ribeiro-Alves, M., Corrêa, R. G., Derrico, M., Lemos, K., Grangeiro, J. R., Jesus, B.d, Pires, D., Veloso, V. G., & Grinsztejn, B. (2016). The Men Who Have Sex with Men HIV Care Cascade in Rio de Janeiro, Brazil. PloS one, 11(6), e0157309. https://doi.org/10.1371/journal.pone.0157309
54	Cowan et al 2017	Cowan, F. M., Davey, C. B., Fearon, E., Mushati, P., Dirawo, J., Cambiano, V., Napierala Mavedzenge, S., Hanisch, D., Wong-Gruenwald, R., Chemhuru, M., Masuka, N., Hatzold, K., Mugurungi, O., Busza, J., Philips, A. N., & Hargreaves, J. R. (2017). The HIV Care Cascade Among Female Sex Workers in Zimbabwe: Results of a Population-Based Survey From the Sisters Antiretroviral Therapy Programme for Prevention of HIV, an Integrated Response (SAPPH-IRe) Trial. Journal of acquired immune deficiency syndromes (1999), 74(4), 375–382. https://doi.org/10.1097/QAI.0000000000001255
57	Schwartz et al 2016	Schwartz, S., Lambert, A., Phaswana-Mafuya, N., Kose, Z., Mcingana, M., Holland, C., Ketende, S., Yah, C., Sweitzer, S., Hausler, H., & Baral, S. (2017). Engagement in the HIV care cascade and barriers to antiretroviral therapy uptake among female sex workers in Port Elizabeth, South Africa: findings from a respondent-driven sampling study. Sexually transmitted infections,

For peer review only - http://bmjopen.bmj.com/site/about/guidelines.xhtml

Paper Number	Authors	Reference
		93(4), 290–296. https://doi.org/10.1136/sextrans-2016-052773
58	Tarigan et al 2020	Tarigan, Y. N., Woodman, R. J., Miller, E. R., Wisaksana, R., Wignall, F. S., & Ward, P. R. (2020). Changes in the HIV continuum of care following expanded access to HIV testing and treatment in Indonesia: A retrospective population-based cohort study. PloS one, 15(9), e0239041. https://doi.org/10.1371/journal.pone.0239041
59	Laisaae et al 2016	Laisaar, K. T., Raag, M., Lutsar, I., & Uusküla, A. (2016). People living with HIV in Estonia: engagement in HIV care in 2013. Euro surveillance : bulletin Europeen sur les maladies transmissibles = European communicable disease bulletin, 21(43), 30380. https://doi.org/10.2807/1560-7917.ES.2016.21.43.30380
60	Ruadze et al 2017	Ruadze, E., Chkhartishvili, N., Chokoshvili, O., & Tsertsvadze, T. (2017). Cascade of care among HIV patients diagnosed in 2013 in Georgia: Risk factors for late diagnosis and attrition from HIV care. SAGE open medicine, 5, 2050312117731977. https://doi.org/10.1177/2050312117731977
61	Elgalib et al 2020	Elgalib, A., Shah, S., Al-Habsi, Z., Al-Fouri, M., Lau, R., Al-Kindi, H., Al-Rawahi, B., & Al-Abri, S. (2020). The cascade of HIV care in Oman, 2015-2018: A population-based study from the Middle East. International journal of infectious diseases : IJID : official publication of the International Society for Infectious Diseases, 90, 28–34. https://doi.org/10.1016/j.ijid.2019.09.017
65	Auld et al 2017	Auld, A. F., Valerie Pelletier, Robin, E. G., Shiraishi, R. W., Dee, J., Antoine, M., Desir, Y., Desforges, G., Delcher, C., Duval, N., Joseph, N., Francois, K., Griswold, M., Domercant, J. W., Patrice Joseph, Y. A., Van Onacker, J. D., Deyde, V., Lowrance, D. W., & And The Groupe d'Analyses Salvh (2017). Retention Throughout the HIV Care and Treatment Cascade: From Diagnosis to Antiretroviral Treatment of Adults and Children Living with HIV-Haiti, 1985-2015. The American journal of tropical medicine and hygiene, 97(4_Suppl), 57–70. https://doi.org/10.4269/ajtmh.17-0116
66	Lurie et al 2020	Lurie, M. N., Kirwa, K., Callaway, J., Cornell, M., Boulle, A., Bengtson, A. M., Smith, M., Leon, N., & Colvin, C. (2020). Quantifying the HIV treatment cascade in a South African health sub-district by gender: retrospective cohort study. Tropical medicine & international health : TM & IH, 25(2), 186–192. https://doi.org/10.1111/tmi.13334

For peer review only - http://bmjopen.bmj.com/site/about/guidelines.xhtml

Paper Number	Authors	Reference
68	Chung et al 2017	Chung, N. C., Bolton-Moore, C., Chilengi, R., Kasaro, M. P., Stringer, J. S., & Chi, B. H. (2017). Patient engagement in HIV care and treatment in Zambia, 2004-2014. Tropical medicine & international health : TM & IH, 22(3), 332–339. https://doi.org/10.1111/tmi.12832
69	Drew et al 2017	Drew, R. S., Rice, B., Rüütel, K., Delpech, V., Attawell, K. A., Hales, D. K., Velasco, C., Amato-Gauci, A. J., Pharris, A., Tavoschi, L., & Noori, T. (2017). HIV continuum of care in Europe and Central Asia. HIV medicine, 18(7), 490–499. https://doi.org/10.1111/hiv.12480
71	Conan et al 2020	Conan, N., Coulborn, R. M., Simons, E., Mapfumo, A., Apollo, T., Garone, D. B., Casas, E. C., Puren, A. J., Chihana, M. L., & Maman, D. (2020). Successes and gaps in the HIV cascade of care of a high HIV prevalence setting in Zimbabwe: a population-based survey. Journal of the International AIDS Society, 23(9), e25613. https://doi.org/10.1002/jia2.25613
72	Ostermann et al 2015	Ostermann, J., Pence, B., Whetten, K., Yao, J., Itemba, D., Maro, V., Reddy, E., & Thielman, N. (2015). HIV serostatus disclosure in the treatment cascade: evidence from Northern Tanzania. AIDS care, 27 Suppl 1(sup1), 59–64. https://doi.org/10.1080/09540121.2015.1090534
73	Ghalehkhani et al 2019	Ghalehkhani, N., Farhoudi, B., Gouya, M. M., Sharifi, H., SeyedAlinaghi, S., Kamali, K., Fahimfar, N., Rajabpour, Z., Doosti-Irani, A., Sedaghat, A., & Mirzazadeh, A. (2019). The HIV treatment cascade in people living with HIV in Iran in 2014: Mixed-method study to measure losses and reasons. International journal of STD & AIDS, 30(13), 1257–1264. https://doi.org/10.1177/0956462419867573
75	Chkhartishvili et al 2015	Chkhartishvili, N., Sharavdze, L., Chokoshvili, O., DeHovitz, J. A., del Rio, C., & Tsertsvadze, T. (2015). The cascade of care in the Eastern European country of Georgia. HIV medicine, 16(1), 62–66. https://doi.org/10.1111/hiv.12172
76	Forbes et al 2016	Forbes, N., Johnson, G., Mortimer, A., Martin, I., Frankson, M., Johnston, K., Thompson, T., & Weissman, S. (2016). The HIV continuum of care in the Bahamas in 2014. Revista panamericana de salud publica = Pan American journal of public health, 40(6), 443–447.
77	Horberg et al 2015	Horberg, M. A., Hurley, L. B., Klein, D. B., Towner, W. J., Kadlecik, P., Antoniskis, D., Mogyoros, M., Brachman, P. S., Remmers, C. L., Gambatese, R. C., Blank, J., Ellis, C. G., & Silverberg, M. J. (2015). The HIV Care Cascade Measured Over Time and by Age, Sex, and Race in a Large National Integrated Care System. AIDS patient care and STDs, 29(11), 582–590. https://doi.org/10.1089/apc.2015.0139

For peer review only - http://bmjopen.bmj.com/site/about/guidelines.xhtml

Paper Number	Authors	Reference
78	Edwards et al 2019	Edwards, J. K., Arimi, P., Ssengooba, F., Mulholland, G., Markiewicz, M., Bukusi, E. A., Orikiiriza, J. T., Virkud, A., & Weir, S. (2019). The HIV care continuum among resident and non-resident populations found in venues in East Africa cross-border areas. Journal of the International AIDS Society, 22(1), e25226. https://doi.org/10.1002/jia2.25226
79	Alvarez-Uria et al 2013	Alvarez-Uria, G., Pakam, R., Midde, M., & Naik, P. K. (2013). Entry, Retention, and Virological Suppression in an HIV Cohort Study in India: Description of the Cascade of Care and Implications for Reducing HIV-Related Mortality in Low- and Middle-Income Countries. Interdisciplinary perspectives on infectious diseases, 2013, 384805. https://doi.org/10.1155/2013/384805
80	Napierala et al 2018	Napierala, S., Chabata, S. T., Fearon, E., Davey, C., Hargreaves, J., Busza, J., Mushati, P., Mtetwa, S., Chiyaka, T., Mugurungi, O., Hanisch, D., Hatzold, K., Phillips, A., & Cowan, F. M. (2018). Engagement in HIV Care Among Young Female Sex Workers in Zimbabwe. Journal of acquired immune deficiency syndromes (1999), 79(3), 358–366. https://doi.org/10.1097/QAI.0000000000001815
81	Wester et al 2016	Wester, C., Rebeiro, P. F., Shavor, T. J., Shepherd, B. E., McGoy, S. L., Daley, B., Morrison, M., Vermund, S. H., & Pettit, A. C. (2016). The 2013 HIV Continuum of Care in Tennessee: Progress Made, but Disparities Persist. Public health reports (Washington, D.C. : 1974), 131(5), 695–703. https://doi.org/10.1177/0033354916660082
82	Heimer et al 2017	Heimer, R., Usacheva, N., Barbour, R., Niccolai, L. M., Uusküla, A., & Levina, O. S. (2017). Engagement in HIV care and its correlates among people who inject drugs in St Petersburg, Russian Federation and Kohtla-Järve, Estonia. Addiction (Abingdon, England), 112(8), 1421–1431. https://doi.org/10.1111/add.13798
83	Gebreegziabher et al 2020	Gebreegziabher, E. A., McCoy, S. I., Ycasas, J. C., & Murgai, N. (2020). The Role of Neighborhood Poverty in the Association between Foreign-Born status and HIV Care Continuum Outcomes in Alameda County, California. Journal of immigrant and minority health, 22(5), 1023–1030. https://doi.org/10.1007/s10903-020-01002-9
84	Davoglio et al 2021	Davoglio, R. S., Gandin, H., & Mocellin, L. P. (2021). HIV/AIDS epidemic in a western border municipality of Rio Grande do Sul, Brazil: evolution, HIV cascade of care and lethality. Revista brasileira de epidemiologia = Brazilian journal of epidemiology, 24(suppl 1), e210018. https://doi.org/10.1590/1980-549720210018.supl.1

For peer review only - http://bmjopen.bmj.com/site/about/guidelines.xhtml

Paper Number	Authors	Reference
85	Muth et al 2017	Muth, S., Len, A., Evans, J. L., Phou, M., Chhit, S., Neak, Y., Ngak, S., Stein, E. S., Carrico, A. W., Maher, L., & Page, K. (2017). HIV treatment cascade among female entertainment and sex workers in Cambodia: impact of amphetamine use and an HIV prevention program. Addiction science & clinical practice, 12(1), 20. https://doi.org/10.1186/s13722-017-0085-x
86	Boothe et al 2021	Boothe, M. A. S., Sathane, I., Baltazar, C. S., Chicuecue, N., Horth, R., Fazito, E., & Raymond, H. F. (2021). Low engagement in HIV services and progress through the treatment cascade among key populations living with HIV in Mozambique: alarming gaps in knowledge of status. BMC public health, 21(1), 146. https://doi.org/10.1186/s12889-020-10039-2
87	Jalil et al 2017	Jalil, E. M., Wilson, E. C., Luz, P. M., Velasque, L., Moreira, R. I., Castro, C. V., Monteiro, L., Garcia, A. C. F., Cardoso, S. W., Coelho, L. E., McFarland, W., Liu, A. Y., Veloso, V. G., Buchbinder, S., & Grinsztejn, B. (2017). HIV testing and the care continuum among transgender women: population estimates from Rio de Janeiro, Brazil. Journal of the International AIDS Society, 20(1), 21873. https://doi.org/10.7448/IAS.20.1.21873
88	Billioux et al 2017	Billioux, V. G., Chang, L. W., Reynolds, S. J., Nakigozi, G., Ssekasanvu, J., Grabowski, M. K., Ssekubugu, R., Nalugoda, F., Kigozi, G., Kagaayi, J., Serwadda, D., Gray, R. H., & Wawer, M. J. (2017). Human immunodeficiency virus care cascade among sub-populations in Rakai, Uganda: an observational study. Journal of the International AIDS Society, 20(1), 21590. https://doi.org/10.7448/IAS.20.1.21590
90	Chowers et al 2020	Chowers, M., Chemtob, D., Mor, O., Levy, I., Elbirt, D., Elinav, H., Rizenberg, K., Lorber, M., Istomin, V., Nemet, S., Shahak, G., & Turner, D. (2020). Continuum of HIV care of newly diagnosed individuals in Israel, 2011-2015: a population-based cohort study. International journal of STD & AIDS, 31(4), 326–334. https://doi.org/10.1177/0956462419891023
91	De Boni et al 2018	De Boni, R. B., Peratikos, M. B., Shepherd, B. E., Grinsztejn, B., Cortés, C., Padgett, D., Gotuzzo, E., Belaunzarán-Zamudio, P. F., Rebeiro, P. F., Duda, S. N., McGowan, C. C., & for CCASAnet (2018). Is substance use associated with HIV cascade outcomes in Latin America?. PloS one, 13(3), e0194228. https://doi.org/10.1371/journal.pone.0194228
92	Ndudzo et al 2019	Ndudzo, C., Tripathy, J. P., Tauro, F., Sibanda, C., Chiramba, M., Shamu, A., Masinire, K., Muchengwa, T., & Kumar, A. M. (2019). HIV care among patients with presumptive tuberculosis in Masvingo district of Zimbabwe, 2017: how well are we doing?. The Pan African medical journal, 33, 158. https://doi.org/10.11604/pamj.2019.33.158.15847

For peer review only - http://bmjopen.bmj.com/site/about/guidelines.xhtml

Paper Number	Authors	Reference
93	Koirala et al 2017	Koirala, S., Deuba, K., Nampaisan, O., Marrone, G., Ekström, A. M., & CAT-S group (2017). Facilitators and barriers for retention in HIV care between testing and treatment in Asia-A study in Bangladesh, Indonesia, Lao, Nepal, Pakistan, Philippines and Vietnam. PloS one, 12(5), e0176914. https://doi.org/10.1371/journal.pone.0176914
94	Alvarez-Uria et al 2014	Alvarez-Uria G. (2014). Description of the cascade of care and factors associated with attrition before and after initiating antiretroviral therapy of HIV infected children in a cohort study in India. PeerJ, 2, e304. https://doi.org/10.7717/peerj.304
95	Wolff et al 2018	Wolff, M. J., Cortes, C. P., Mejìa, F. A., Padgett, D., Belaunzarán-Zamudio, P., Grinsztejn, B., Giganti, M. J., McGowan, C. C., Rebeiro, P. F., & Caribbean, Central and South America network for HIV epidemiology (CCASAnet) (2018). Evaluating the care cascade after antiretroviral therapy initiation in Latin America. International journal of STD & AIDS, 29(1), 4–12. https://doi.org/10.1177/0956462417714094
96	Reyes-uruena et al 2017	Reyes-Urueña, J., Campbell, C., Hernando, C., Vives, N., Folch, C., Ferrer, L., Fernández-López, L., Esteve, A., & Casabona, J. (2017). Differences between migrants and Spanish-born population through the HIV care cascade, Catalonia: an analysis using multiple data sources. Epidemiology and infection, 145(8), 1670–1681. https://doi.org/10.1017/S0950268817000437
97	Santos et al 2014	Santos, G. M., Wilson, E. C., Rapues, J., Macias, O., Packer, T., & Raymond, H. F. (2014). HIV treatment cascade among transgender women in a San Francisco respondent driven sampling study. Sexually transmitted infections, 90(5), 430–433. https://doi.org/10.1136/sextrans-2013-051342
98	Wechsberg et al 2017	Wechsberg, W. M., van der Horst, C., Ndirangu, J., Doherty, I. A., Kline, T., Browne, F. A., Belus, J. M., Nance, R., & Zule, W. A. (2017). Seek, test, treat: substance-using women in the HIV treatment cascade in South Africa. Addiction science & clinical practice, 12(1), 12. https://doi.org/10.1186/s13722-017-0077-x
99	Jaries et al 2017	Jaries, R., Vantilcke, V., Clevenbergh, P., Adoissi, J., Boukhari, R., Misslin, C., Nacher, M., Vreden, S., & Jolivet, A. (2017). Population movements and the HIV cascade in recently diagnosed patients at the French Guiana -Suriname border. AIDS care, 29(11), 1448–1452. https://doi.org/10.1080/09540121.2017.1291899
100	Kelly et al 2016	Kelly, J. D., Schlough, G. W., Conteh, S., Barrie, M. B., Kargbo, B., & Giordano, T. P. (2016). The Majority of the Pre-Antiretroviral Population Who Were Lost to Follow-Up Stopped Their Care in Freetown, Sierra Leone: A 12-Month Prospective Cohort Study Starting with HIV Diagnosis. PloS one, 11(2), e0149584. https://doi.org/10.1371/journal.pone.0149584

For peer review only - http://bmjopen.bmj.com/site/about/guidelines.xhtml

Paper Number	Authors	Reference
101	Medland et al 2017	Medland, N. A., Chow, E. P. F., McMahon, J. H., Elliott, J. H., Hoy, J. F., & Fairley, C. K. (2017). Time from HIV diagnosis to commencement of antiretroviral therapy as an indicator to supplement the HIV cascade: Dramatic fall from 2011 to 2015. PloS one, 12(5), e0177634. https://doi.org/10.1371/journal.pone.0177634
102	Kalawan et al 2020	Kalawan, V., Naidoo, K., & Archary, M. (2020). Impact of routine birth early infant diagnosis on neonatal HIV treatment cascade in eThekwini district, South Africa. Southern African journal of HIV medicine, 21(1), 1084. https://doi.org/10.4102/sajhivmed.v21i1.1084
103	Gissl'en et al 2017	Gisslén, M., Svedhem, V., Lindborg, L., Flamholc, L., Norrgren, H., Wendahl, S., Axelsson, M., & Sönnerborg, A. (2017). Sweden, the first country to achieve the Joint United Nations Programme on HIV/AIDS (UNAIDS)/World Health Organization (WHO) 90-90-90 continuum of HIV care targets. HIV medicine, 18(4), 305–307. https://doi.org/10.1111/hiv.12431
104	Maman et al 2016	Maman, D., Chilima, B., Masiku, C., Ayouba, A., Masson, S., Szumilin, E., Peeters, M., Ford, N., Heinzelmann, A., Riche, B., & Etard, J. F. (2016). Closer to 90-90-90. The cascade of care after 10 years of ART scale-up in rural Malawi: a population study. Journal of the International AIDS Society, 19(1), 20673. https://doi.org/10.7448/IAS.19.1.20673
106	Jin et al 2018	Jin, H., Restar, A., Biello, K., Kuhns, L., Reisner, S., Garofalo, R., & Mimiaga, M. J. (2019). Burden of HIV among young transgender women: factors associated with HIV infection and HIV treatment engagement. AIDS care, 31(1), 125–130. https://doi.org/10.1080/09540121.2018.1539213
107	Krentz et al 2015	Krentz, H. B., MacDonald, J., & Gill, M. J. (2015). The impact of transfer patients on the local cascade of HIV care continuum. Journal of acquired immune deficiency syndromes (1999), 68(2), 236–240. https://doi.org/10.1097/QAI.0000000000000430
108	Marukutira et al 2019	Marukutira, T., Yin, D., Cressman, L., Kariuki, R., Malone, B., Spelman, T., Mawandia, S., Ledikwe, J. H., Semo, B. W., Crowe, S., Stoove, M., Hellard, M., & Dickinson, D. (2019). Clinical outcomes of a cohort of migrants and citizens living with human immunodeficiency virus in Botswana: implications for Joint United Nation Program on HIV and AIDS 90-90-90 targets. Medicine, 98(23), e15994. https://doi.org/10.1097/MD.0000000000015994
109	Govender et al 2020	Govender, K., Durevall, D., Cowden, R. G., Beckett, S., Kharsany, A. B., Lewis, L., George, G., Cawood, C., & Khanyile, D. (2022). Depression symptoms, HIV testing, linkage to ART, and viral suppression among women in a high HIV burden district in KwaZulu-Natal, South Africa: A cross-sectional household study. Journal of health

For peer review only - http://bmjopen.bmj.com/site/about/guidelines.xhtml

Paper Number	Authors	Reference
		psychology, 27(4), 936–945. https://doi.org/10.1177/1359105320982042
111	Rhead et al 2018	Rhead, R., Elmes, J., Otobo, E., Nhongo, K., Takaruza, A., White, P. J., Nyamukapa, C. A., & Gregson, S. (2018). Do female sex workers have lower uptake of HIV treatment services than non-sex workers? A cross-sectional study from east Zimbabwe. BMJ open, 8(2), e018751. https://doi.org/10.1136/bmjopen-2017-018751
112	Khumalo et al 2020	Khumalo, P. N., Sacks, E., Chouraya, C., Tsabedze, B., Masuku, T., Nyoni, G., Zikalala, T., Nhlabatsi, B., Mthethwa, N., & Cohn, J. (2020). The Cascade of Care From Routine Point-of-Care HIV Testing at Birth: Results From an 18-Months Pilot Program in Eswatini. Journal of acquired immune deficiency syndromes (1999), 84 Suppl 1(1), S22–S27. https://doi.org/10.1097/QAI.0000000000002380
113	Moore et al 2021	Moore, D. M., Cui, Z., Skakoon-Sparling, S., Sang, J., Barath, J., Wang, L., Lachowsky, N., Cox, J., Lambert, G., Noor, S. W., Grace, D., Jollimore, J., Apelian, H., Lal, A., Parlette, A., & Hart, T. A. (2021). Characteristics of the HIV cascade of care and unsuppressed viral load among gay, bisexual and other men who have sex with men living with HIV across Canada's three largest cities. Journal of the International AIDS Society, 24(4), e25699. https://doi.org/10.1002/jia2.25699
114	Fearon et al 2020	Fearon, E., Tenza, S., Mokoena, C., Moodley, K., Smith, A. D., Bourne, A., Weatherburn, P., & Palanee-Phillips, T. (2020). HIV testing, care and viral suppression among men who have sex with men and transgender individuals in Johannesburg, South Africa. PloS one, 15(6), e0234384. https://doi.org/10.1371/journal.pone.0234384
115	Rossi et al 2020	Rossi, A. D. M., Albanese, S. P. R., Vogler, I. H., Pieri, F. M., Lentine, E. C., Birolim, M. M., & Dessunti, E. M. (2020). HIV Care Continuum from diagnosis in a Counseling and Testing Center. Revista brasileira de enfermagem, 73(6), e20190680. https://doi.org/10.1590/0034-7167-2019-0680
116	Sirirungsi et al 2016	Sirirungsi, W., Khamduang, W., Collins, I. J., Pusamang, A., Leechanachai, P., Chaivooth, S., Ngo-Giang-Huong, N., & Samleerat, T. (2016). Early infant HIV diagnosis and entry to HIV care cascade in Thailand: an observational study. The lancet. HIV, 3(6), e259–e265. https://doi.org/10.1016/S2352-3018(16)00045-X

For peer review only - http://bmjopen.bmj.com/site/about/guidelines.xhtml

Paper Number	Authors	Reference
118	Bachanas et al 2021	Bachanas, P., Alwano, M. G., Lebelonyane, R., Block, L., Behel, S., Raizes, E., Ussery, G., Wang, H., Ussery, F., Pretorius Holme, M., Sexton, C., Pals, S., Lasry, A., Del Castillo, L., Hader, S., Lockman, S., Bock, N., & Moore, J. (2021). Finding, treating and retaining persons with HIV in a high HIV prevalence and high treatment coverage country: Results from the Botswana Combination Prevention Project. PloS one, 16(4), e0250211. https://doi.org/10.1371/journal.pone.0250211
119	Huerga et al 2018	Huerga, H., Van Cutsem, G., Ben Farhat, J., Puren, A., Bouhenia, M., Wiesner, L., Dlamini, L., Maman, D., Ellman, T., & Etard, J. F. (2018). Progress towards the UNAIDS 90-90-90 goals by age and gender in a rural area of KwaZulu-Natal, South Africa: a household-based community cross-sectional survey. BMC public health, 18(1), 303. https://doi.org/10.1186/s12889-018-5208-0
120	Vetrova et al 2018	Vetrova, M. V., Aleksandrova, O. V., Paschenko, A. E., Toropov, S. E., Rassokhin, V. V., Abyshev, R. A., Levina, O. S., Niccolai, L. M., & Heimer, R. (2018). Early stages of HIV treatment cascade in people living with HIV in Saint-Petersburg, Russia. AIDS care, 30(7), 857–862. https://doi.org/10.1080/09540121.2017.1417536
122	Bijker et al 2020	Bijker, R., Kumarasamy, N., Kiertiburanakul, S., Pujari, S., Ng, O. T., Sun, L. P., Merati, T. P., Van Nguyen, K., Lee, M. P., Cuong, D. D., Chan, Y. J., Choi, J. Y., Ross, J., Law, M., & IeDEA Asia-Pacific (2020). An expanded HIV care cascade: ART uptake, viral load suppression and comorbidity monitoring among adults living with HIV in Asia. Antiviral therapy, 25(5), 275–285. https://doi.org/10.3851/IMP3379
123	Jean et al 2016	Jean, K., Puren, A., Cutler, E., Singh, B., Bouscaillou, J., Rain-Taljaard, R., Taljaard, D., Gouws, E., Lissouba, P., Lewis, D. A., Peytavin, G., & Auvert, B. (2016). Level of viral suppression and the cascade of HIV care in a South African semi-urban setting in 2012. AIDS (London, England), 30(13), 2107–2116. https://doi.org/10.1097/QAD.0000000000001155
124	Anglemyer et al 2020	Anglemyer, A., Haber, N., Noiman, A., Rutherford, G., Ganesan, A., Blaylock, J., Okulicz, J., Maves, R. C., Lalani, T., Schofield, C., Mancuso, J., Agan, B. K., & Infectious Disease Clinical Research Program HIV Working Group (2020). HIV Care Continuum and Meeting 90-90-90 Targets: Cascade of Care Analyses of a U.S. Military Cohort. Military medicine, 185(7-8), e1147–e1154. https://doi.org/10.1093/milmed/usaa021
136	Lally et al 2018	Lally, M. A., van den Berg, J. J., Westfall, A. O., Rudy, B. J., Hosek, S. G., Fortenberry, J. D., Monte, D., Tanney, M. R., McFarland, E. J., Xu, J., Kapogiannis, B. G., Wilson, C. M., & Adolescent Medicine Trials Network for HIV/AIDS Interventions (ATN) (2018). HIV Continuum of Care for Youth in the United States. Journal of acquired

For peer review only - http://bmjopen.bmj.com/site/about/guidelines.xhtml

Paper Number	Authors	Reference
		immune deficiency syndromes (1999), 77(1), 110–117. https://doi.org/10.1097/QAI.0000000000001563
137	Colombe et al 2019	Colombe, S., Machemba, R., Mtenga, B., Lutonja, P., Safari, W., Beard, J., Downs, J. A., Urassa, M., Todd, J., & Changalucha, J. (2020). Cascade of care for HIV-seroconverters in rural Tanzania: a longitudinal study. AIDS care, 32(5), 666–671. https://doi.org/10.1080/09540121.2019.1640842
139	Gesesew et al 2020	Gesesew, H. A., Ward, P., Woldemichael, K., & Mwanri, L. (2020). HIV Care continuum Outcomes: Can Ethiopia Meet the UNAIDS 90-90-90 Targets?. Ethiopian journal of health sciences, 30(2), 179–188. https://doi.org/10.4314/ejhs.v30i2.5
140	Stephens et al 2021	Jacqueline H. Stephens, Richard T Gray, Rebecca Guy, Tobias Vickers & James Ward (2021): A HIV diagnosis and treatment cascade for Aboriginal and Torres Strait Islander peoples of Australia, AIDS Care, DOI: 10.1080/09540121.2021.2001416
141	Vourli et al 2018	Vourli, G., Nikolopoulos, G., Paparizos, V., Skoutelis, A., Metallidis, S., Gargalianos, P., Papadopoulos, A., Chini, M., Sipsas, N. V., Psychogiou, M., Chrysos, G., Sambatakou, H., Gogos, C., Katsarou, O., Paraskeva, D., Dedes, N., Touloumi, G., & Greek HIV Prevention Group (2018). HIV cascade of care in Greece: Useful insights from additional stages. PloS one, 13(11), e0207355. https://doi.org/10.1371/journal.pone.0207355
142	Nijhawan et al 2020	Nijhawan, A. E., Bhattatiry, M., Chansard, M., Zhang, S., & Halm, E. A. (2020). HIV care cascade before and after hospitalization: impact of a multidisciplinary inpatient team in the US South. AIDS care, 32(11), 1343–1352. https://doi.org/10.1080/09540121.2019.1698704
144	Brown et al 2018	Brown, A. E., Attawell, K., Hales, D., Rice, B. D., Pharris, A., Supervie, V., Van Beckhoven, D., Delpech, V. C., An der Heiden, M., Marcus, U., Maly, M., & Noori, T. (2018). Monitoring the HIV continuum of care in key populations across Europe and Central Asia. HIV medicine, 10.1111/hiv.12603. Advance online publication. https://doi.org/10.1111/hiv.12603
145	Johansson et al 2021	Johansson, M., Penno, C., Winqvist, N., Tesfaye, F., & Björkman, P. (2021). How does HIV testing modality impact the cascade of care among persons diagnosed with HIV in Ethiopia?. Global health action, 14(1), 1933788. https://doi.org/10.1080/16549716.2021.1933788

For peer review only - http://bmjopen.bmj.com/site/about/guidelines.xhtml

Paper Number	Authors	Reference
146	Meloni et al 2020	Meloni, S. T., Agaba, P., Chang, C. A., Yiltok, E., Oguche, S., Ejeliogu, E., Agbaji, O., Okonkwo, P., & Kanki, P. J. (2020). Longitudinal evaluation of adherence, retention, and transition patterns of adolescents living with HIV in Nigeria. PloS one, 15(7), e0236801. https://doi.org/10.1371/journal.pone.0236801
147	Guaraldi et al 2021	Guaraldi, G., Borghi, V., Milic, J., Carli, F., Cuomo, G., Menozzi, M., Santoro, A., Orlando, G., Puzzolante, C., Meschiari, M., Franceschini, E., Bedini, A., Ferrari, F., Gennari, W., Sarti, M., & Mussini, C. (2021). The Impact of COVID-19 on UNAIDS 90-90-90 Targets: Calls for New HIV Care Models. Open forum infectious diseases, 8(7), ofab283. https://doi.org/10.1093/ofid/ofab283
148	Wang et al 2020	Wang, G., Lu, C., Qin, S., Wei, W., Lai, J., Jiang, J., Liang, B., Zhou, O., Han, J., Yang, Y., Ye, L., Liang, H., & Ning, C. (2020). 90-90-90 cascade analysis on reported CLHIV infected by mother-to-child transmission in Guangxi, China: a modeling study. Scientific reports, 10(1), 5295. https://doi.org/10.1038/s41598-020-62281-8
150	Lacombe-Duncan et al 2019	Lacombe-Duncan, A., Bauer, G. R., Logie, C. H., Newman, P. A., Shokoohi, M., Kay, E. S., Persad, Y., O'Brien, N., Kaida, A., de Pokomandy, A., & Loutfy, M. (2019). The HIV Care Cascade Among Transgender Women with HIV in Canada: A Mixed-Methods Study. AIDS patient care and STDs, 33(7), 308–322. https://doi.org/10.1089/apc.2019.0013
151	Boerma et al 2019	Boerma, R., Schellekens, O., Rinke de Wit, T. F., Wit, F. W., van der Borght, S., Rijckborst, H., Chukwumah, P., & Schilthuis, H. (2019). Reaching 90-90-90: outcomes of a 15-year multi-country HIV workplace programme in sub-Saharan Africa. Antiviral therapy, 24(5), 363–370. https://doi.org/10.3851/IMP3311
152	Van Santen et al 2021	van Santen, D. K., Asselin, J., Haber, N. A., Traeger, M. W., Callander, D., Donovan, B., El-Hayek, C., McMahon, J. H., Petoumenos, K., McManus, H., Hoy, J. F., Hellard, M., Guy, R., Stoové, M., & TAIPAN investigators (2021). Improvements in transition times through the HIV cascade of care among gay and bisexual men with a new HIV diagnosis in New South Wales and Victoria, Australia (2012-19): a longitudinal cohort study. The lancet. HIV, 8(10), e623–e632. https://doi.org/10.1016/S2352-3018(21)00155-7
153	Comelli et al 2019	Comelli, A., Izzo, I., Donato, F., Celotti, A., Focà, E., Pezzoli, C., Castelli, F., & Quiros-Roldan, E. (2019). Disengagement and reengagement of HIV continuum of care in a single center cohort in northern Italy. HIV research & clinical practice, 20(1), 1–11. https://doi.org/10.1080/15284336.2019.1595887

For peer review only - http://bmjopen.bmj.com/site/about/guidelines.xhtml

Paper Number	Authors	Reference
154	Rohr et al 2019	Rohr, J. K., Manne-Goehler, J., Gómez-Olivé, F. X., Wagner, R. G., Rosenberg, M., Geldsetzer, P., Kabudula, C., Kahn, K., Tollman, S., Bärnighausen, T., & Salomon, J. A. (2020). HIV treatment cascade for older adults in rural South Africa. Sexually transmitted infections, 96(4), 271–276. https://doi.org/10.1136/sextrans-2018-053925
156	Iwamoto et al 2017	Iwamoto, A., Taira, R., Yokomaku, Y., Koibuchi, T., Rahman, M., Izumi, Y., & Tadokoro, K. (2017). The HIV care cascade: Japanese perspectives. PloS one, 12(3), e0174360. https://doi.org/10.1371/journal.pone.0174360
157	Chappell et al 2019	Chappell, E., Lyall, H., Riordan, A., Thorne, C., Foster, C., Butler, K., Prime, K., Bamford, A., Peters, H., Judd, A., Collins, I. J., & Collaborative HIV Paediatric Study Steering Committee (2019). The cascade of care for children and adolescents with HIV in the UK and Ireland, 2010 to 2016. Journal of the International AIDS Society, 22(9), e25379. https://doi.org/10.1002/jia2.25379
159	Touloumi et al 2022	Touloumi, G., Thomadakis, C., Pantazis, N., Papastamopoulos, V., Paparizos, V., Metallidis, S., Adamis, G., Chini, M., Psichogiou, M., Chrysos, G., Sambatakou, H., Barbunakis, E., Vourli, G., Antoniadou, A., & AMACS (2022). HIV continuum of care: bridging cross-sectional and longitudinal analyses. AIDS (London, England), 36(4), 583–591. https://doi.org/10.1097/QAD.0000000000003131
161	Agolory et al 2018	Agolory, S., de Klerk, M., Baughman, A. L., Sawadogo, S., Mutenda, N., Pentikainen, N., Shoopala, N., Wolkon, A., Taffa, N., Mutandi, G., Jonas, A., Mengistu, A. T., Dzinotyiweyi, E., Prybylski, D., Hamunime, N., & Medley, A. (2018). Low Case Finding Among Men and Poor Viral Load Suppression Among Adolescents Are Impeding Namibia's Ability to Achieve UNAIDS 90-90-90 Targets. Open forum infectious diseases, 5(9), ofy200. https://doi.org/10.1093/ofid/ofy200
162	Lopez-Varela et al 2021	Lopez-Varela, E., Augusto, O., Fuente-Soro, L., Sacoor, C., Nhacolo, A., Casavant, I., Karajeanes, E., Vaz, P., & Naniche, D. (2021). Quantifying the gender gap in the HIV care cascade in southern Mozambique: We are missing the men. PloS one, 16(2), e0245461. https://doi.org/10.1371/journal.pone.0245461
163	Kalichman et al 2017	Kalichman, S. C., Hernandez, D., Finneran, S., Price, D., & Driver, R. (2017). Transgender women and HIV-related health disparities: falling off the HIV treatment cascade. Sexual health, 14(5), 469–476. https://doi.org/10.1071/SH17015
164	Lopez-Varela et al 2018	Lopez-Varela, E., Fuente-Soro, L., Augusto, O. J., Sacoor, C., Nhacolo, A., Karajeanes, E., Vaz, P., & Naniche, D. (2018). Continuum of HIV Care in Rural Mozambique: The Implications of HIV Testing Modality on Linkage and Retention. Journal of acquired immune deficiency syndromes (1999), 78(5), 527–535. https://doi.org/10.1097/QAI.0000000000001720

For peer review only - http://bmjopen.bmj.com/site/about/guidelines.xhtml

Paper Number	Authors	Reference
165	Schiaroli et al 2020	Schiaroli, E., De Socio, G. V., Gabrielli, C., Papalini, C., Nofri, M., Baldelli, F., & Francisci, D. (2020). Partial Achievement of the 90-90-90 UNAIDS Target in a Cohort of HIV Infected Patients from Central Italy. Mediterranean journal of hematology and infectious diseases, 12(1), e2020017. https://doi.org/10.4084/MJHID.2020.017
166	Tarigan et al 2021	Tarigan, Y. N., Woodman, R. J., Miller, E. R., Wisaksana, R., & Ward, P. R. (2021). Impact of strategic use of antiretroviral therapy intervention to the HIV continuum of care in 13 cities in Indonesia: an interrupted time series analysis. AIDS research and therapy, 18(1), 22. https://doi.org/10.1186/s12981-021-00340-4
167	Edun et al 2015	Edun, B., Iyer, M., Albrecht, H., & Weissman, S. (2015). The South Carolina HIV Cascade of Care. Southern medical journal, 108(11), 670–674. https://doi.org/10.14423/SMJ.0000000000000368
168	Ssekalembe et al 2020	Ssekalembe, G., Isfandiari, M. A., & Suprianto, H. (2020). Current Status Towards 90-90-90 UNAIDS Target and Factors Associated with HIV Viral Load Suppression in Kediri City, Indonesia. HIV/AIDS (Auckland, N.Z.), 12, 47–57. https://doi.org/10.2147/HIV.S231173
169	Whittaker et al 2020	Whittaker, R., Case, K. K., Nilsen, Ø., Blystad, H., Cowan, S., Kløvstad, H., & van Sighem, A. (2020). Monitoring progress towards the first UNAIDS 90-90-90 target in key populations living with HIV in Norway. BMC infectious diseases, 20(1), 451. https://doi.org/10.1186/s12879-020-05178-1
170	Porter et al 2018	Porter, K., Gourlay, A., Attawell, K., Hales, D., Supervie, V., Touloumi, G., Rosinska, M., Vourli, G., van Sighem, A., Pharris, A., Noori, T., & ECDC Dublin Declaration Monitoring Network (2018). Substantial Heterogeneity in Progress Toward Reaching the 90-90-90 HIV Target in the WHO European Region. Journal of acquired immune deficiency syndromes (1999), 79(1), 28–37. https://doi.org/10.1097/QAI.0000000000001761
171	Ma et al 2018	Ma, Y., Dou, Z., Guo, W., Mao, Y., Zhang, F., McGoogan, J. M., Zhao, Y., Zhao, D., Wu, Y., Liu, Z., & Wu, Z. (2018). The Human Immunodeficiency Virus Care Continuum in China: 1985-2015. Clinical infectious diseases : an official publication of the Infectious Diseases Society of America, 66(6), 833–839. https://doi.org/10.1093/cid/cix911

For peer review only

For peer review only - http://bmjopen.bmj.com/site/about/guidelines.xhtml

Paper Number	Authors	Reference
172	Vourli et al 2020	Vourli, G., Noori, T., Pharris, A., Porter, K., Axelsson, M., Begovac, J., Cazein, F., Costagliola, D., Cowan, S., Croxford, S., d'Arminio Monforte, A., Delpech, V., Díaz, A., Girardi, E., Gunsenheimer-Bartmeyer, B., Hernando, V., Leierer, G., Lot, F., Nunez, O., Obel, N., … European HIV Continuum of Care Working Group (2020). Human Immunodeficiency Virus Continuum of Care in 11 European Union Countries at the End of 2016 Overall and by Key Population: Have We Made Progress?. Clinical infectious diseases : an official publication of the Infectious Diseases Society of America, 71(11), 2905–2916. https://doi.org/10.1093/cid/ciaa696
174	Marinda et al 2020	Marinda, E., Simbayi, L., Zuma, K., Zungu, N., Moyo, S., Kondlo, L., Jooste, S., Nadol, P., Igumbor, E., Dietrich, C., & Briggs-Hagen, M. (2020). Towards achieving the 90-90-90 HIV targets: results from the south African 2017 national HIV survey. BMC public health, 20(1), 1375. https://doi.org/10.1186/s12889-020-09457-z
176	Cesar et al 2016	Cesar, C., Blugerman, G., Valiente, J. A., Rebeiro, P., Sued, O., Fink, V., Soto, M. R., Cillis, R., Yamamoto, C., Falistocco, C., Cahn, P., & Pérez, H. (2016). The HIV care cascade in Buenos Aires, Argentina: results in a tertiary referral hospital. Revista panamericana de salud publica = Pan American journal of public health, 40(6), 448–454.
177	Poteat at al 2020	Poteat, T., Hanna, D. B., Rebeiro, P. F., Klein, M., Silverberg, M. J., Eron, J. J., Horberg, M. A., Kitahata, M. M., Mathews, W. C., Mattocks, K., Mayor, A., Rich, A. J., Reisner, S., Thorne, J., Moore, R. D., Jing, Y., & Althoff, K. N. (2020). Characterizing the Human Immunodeficiency Virus Care Continuum Among Transgender Women and Cisgender Women and Men in Clinical Care: A Retrospective Time-series Analysis. Clinical infectious diseases : an official publication of the Infectious Diseases Society of America, 70(6), 1131–1138. https://doi.org/10.1093/cid/ciz322
178	Bowman et al 2015	Bowman, A. S., Lerebours, L., Amesty, S., de la Rosa, M., Gil, E., Halpern, M., Nicholas, S., & Lamb, M. R. (2016). Evaluation of patient care cascade for HIV-positive patients diagnosed in La Romana, Dominican Republic in 2011: a retrospective cohort study. International journal of STD & AIDS, 27(5), 394–401. https://doi.org/10.1177/0956462415584487
179	Chkhartishvili et al 2016	Chkhartishvili, N., Chokoshvili, O., Dvali, N., Abutidze, A., Sharvadze, L., & Tsertsvadze, T. (2016). Significant Improvements Are Needed in HIV Care Continuum to Meet 90-90-90 Targets in Georgia. Journal of the International Association of Providers of AIDS Care, 15(6), 451–454. https://doi.org/10.1177/2325957416667487

For peer review only - http://bmjopen.bmj.com/site/about/guidelines.xhtml

Paper Number	Authors	Reference
180	Grobler et al 2017	Grobler, A., Cawood, C., Khanyile, D., Puren, A., & Kharsany, A. B. M. (2017). Progress of UNAIDS 90-90-90 targets in a district in KwaZulu-Natal, South Africa, with high HIV burden, in the HIPSS study: a household-based complex multilevel community survey. The lancet. HIV, 4(11), e505–e513. https://doi.org/10.1016/S2352-3018(17)30122-4
181	Meteliuk et al 2020	Meteliuk, A., Prokhorova, T., Filippovych, S., Ompad, D. C., & Zaller, N. (2020). The role of access to integrated services at opioid agonist treatment sites in reaching 90-90-90 cascade in people who inject drugs in Ukraine: Country-level data. Drug and alcohol dependence, 216, 108216. https://doi.org/10.1016/j.drugalcdep.2020.108216
182	Mazhnaya et al 2018	Mazhnaya, A., Marcus, R., Bojko, M. J., Zelenev, A., Makarenko, I., Pykalo, I., Filippovych, S., Dvoriak, S., & Altice, F. L. (2018). Opioid Agonist Treatment and Improved Outcomes at Each Stage of the HIV Treatment Cascade in People Who Inject Drugs in Ukraine. Journal of acquired immune deficiency syndromes (1999), 79(3), 288–295. https://doi.org/10.1097/QAI.0000000000001827
184	Chkhartishvili et al 2017	Chkhartishvili, N., Chokoshvili, O., Abutidze, A., Dvali, N., Del Rio, C., & Tsertsvadze, T. (2017). Progress Toward Achieving the UNAIDS 90-90-90 Goals in HIV Care From Diagnosis to Durable Viral Suppression in the Country of Georgia. AIDS research and human retroviruses, 33(10), 999–1003. https://doi.org/10.1089/AID.2016.0103
185	Psichogiou et al 2019	Psichogiou, M., Giallouros, G., Pantavou, K., Pavlitina, E., Papadopoulou, M., Williams, L. D., Hadjikou, A., Kakalou, E., Skoutelis, A., Protopapas, K., Antoniadou, A., Boulmetis, G., Paraskevis, D., Hatzakis, A., Friedman, S. R., & Nikolopoulos, G. K. (2019). Identifying, linking, and treating people who inject drugs and were recently infected with HIV in the context of a network-based intervention. AIDS care, 31(11), 1376–1383. https://doi.org/10.1080/09540121.2019.1601671
186	Bowman et al 2017	Bowman, A. S., Mehta, M., Lerebours Nadal, L., Halpern, M., Nicholas, S. W., & Amesty, S. (2017). Strengthening the HIV Care Continuum in the Dominican Republic: Application of a Triadic Implementation Framework to Meet the UNAIDS 90-90-90 Treatment Goal. AIDS patient care and STDs, 31(10), 407–412. https://doi.org/10.1089/apc.2017.0118
187	Ghiam et al 2017	Ghiam, M. K., Rebeiro, P. F., Turner, M., Rogers, W. B., Bebawy, S. S., Raffanti, S. P., Person, A. K., & Pettit, A. C. (2017). Trends in HIV Continuum of Care Outcomes over Ten Years of Follow-Up at a Large HIV Primary Medical Home in the Southeastern United States. AIDS research and human retroviruses, 33(10), 1027–1034. https://doi.org/10.1089/AID.2017.0016

For peer review only - http://bmjopen.bmj.com/site/about/guidelines.xhtml

Paper Number	Authors	Reference
188	Aboobaker et al 2022	Aboobaker, A., Zingela, Z., & Adeniyi, O. V. (2022). Correlates and cascade of HIV care in patients with psychiatric disorders in the Eastern Cape province, South Africa. The South African journal of psychiatry : SAJP : the journal of the Society of Psychiatrists of South Africa, 28, 1753. https://doi.org/10.4102/sajpsychiatry.v28i0.1753
190	Jaffer et al 2022	Jaffer, M., Christofides, N., Hlongwane, K., Otwombe, K., Milovanovic, M., Hopkins, K. L., Matuludi, M., Mbowane, V., Abdullah, F., Gray, G., Jewkes, R., & Coetzee, J. (2022). The HIV Cascade of Care and Service Utilisation at Sex Work Programmes Among Female Sex Workers in South Africa. AIDS and behavior, 26(9), 2907–2919. https://doi.org/10.1007/s10461-022-03616-6
191	Balayan et al 2019	Balayan, T., Oprea, C., Yurin, O., Jevtovic, D., Begovac, J., Lakatos, B., Sedlacek, D., Karpov, I., Horban, A., Kowalska, J. D., & Euro-guidelines in Central and Eastern Europe Network Group (2019). People who inject drugs remain hard-to-reach population across all HIV continuum stages in Central, Eastern and South Eastern Europe - data from Euro-guidelines in Central and Eastern Europe Network. Infectious diseases (London, England), 51(4), 277–286. https://doi.org/10.1080/23744235.2019.1565415
192	Colasanti et al 2016	Colasanti, J., Kelly, J., Pennisi, E., Hu, Y. J., Root, C., Hughes, D., Del Rio, C., & Armstrong, W. S. (2016). Continuous Retention and Viral Suppression Provide Further Insights Into the HIV Care Continuum Compared to the Cross-sectional HIV Care Cascade. Clinical infectious diseases : an official publication of the Infectious Diseases Society of America, 62(5), 648–654. https://doi.org/10.1093/cid/civ941
193	Raymond et al 2016	Raymond, H. F., Scheer, S., Santos, G. M., & McFarland, W. (2016). Examining progress toward the UNAIDS 90-90-90 framework among men who have sex with men, San Francisco, 2014. AIDS care, 28(9), 1177–1180. https://doi.org/10.1080/09540121.2016.1153593
194	Kelly et al 2014	Kelly, J. D., Hartman, C., Graham, J., Kallen, M. A., & Giordano, T. P. (2014). Social support as a predictor of early diagnosis, linkage, retention, and adherence to HIV care: results from the steps study. The Journal of the Association of Nurses in AIDS Care : JANAC, 25(5), 405–413. https://doi.org/10.1016/j.jana.2013.12.002
195	Hakim et al 2022	Hakim, A. J., Bolo, A., Coy, K. C., Achut, V., Katoro, J., Caesar, G., Lako, R., Taban, A. I., Sleeman, K., Wesson, J., & Okiria, A. G. (2022). Progress toward the UNAIDS 90-90-90 targets among female sex workers and sexually exploited female adolescents in Juba and Nimule, South Sudan. BMC public health, 22(1), 132. https://doi.org/10.1186/s12889-022-12533-1

For peer review only - http://bmjopen.bmj.com/site/about/guidelines.xhtml

Paper Number	Authors	Reference
196	Holland et al 2016	Holland, C. E., Kouanda, S., Lougué, M., Pitche, V. P., Schwartz, S., Anato, S., Ouedraogo, H. G., Tchalla, J., Yah, C. S., Kapesa, L., Ketende, S., Beyrer, C., & Baral, S. (2016). Using Population-Size Estimation and Cross-sectional Survey Methods to Evaluate HIV Service Coverage Among Key Populations in Burkina Faso and Togo. Public health reports (Washington, D.C. : 1974), 131(6), 773–782. https://doi.org/10.1177/0033354916677237
198	Krentz et al 2014	Krentz, H. B., MacDonald, J., & John Gill, M. (2014). High Mortality Among Human Immunodeficiency Virus (HIV)-Infected Individuals Before Accessing or Linking to HIV Care: A Missing Outcome in the Cascade of Care?. Open forum infectious diseases, 1(1), ofu011. https://doi.org/10.1093/ofid/ofu011
199	Lima et al 2017	Lima, V. D., St-Jean, M., Rozada, I., Shoveller, J. A., Nosyk, B., Hogg, R. S., Sereda, P., Barrios, R., & Montaner, J. S. G. (2017). Progress towards the United Nations 90-90-90 and 95-95-95 targets: the experience in British Columbia, Canada. Journal of the International AIDS Society, 20(3), e25011. https://doi.org/10.1002/jia2.25011
200	Doshi et al 2018	Doshi, R. H., Sande, E., Ogwal, M., Kiyingi, H., McIntyre, A., Kusiima, J., Musinguzi, G., Serwadda, D., & Hladik, W. (2018). Progress toward UNAIDS 90-90-90 targets: A respondent-driven survey among female sex workers in Kampala, Uganda. PloS one, 13(9), e0201352. https://doi.org/10.1371/journal.pone.0201352
201	Harooni et al 2022	Harooni, M. Z., Atarud, A. A., Ehsan, E., Alokozai, A., McFarland, W., & Mirzazadeh, A. (2022). Gaps in the continuum of care among people living with HIV in Afghanistan. International journal of STD & AIDS, 33(3), 282–288. https://doi.org/10.1177/09564624211055299
202	Harris et al 2022	Harris, T. G., Wu, Y., Parmley, L. E., Musuka, G., Mapingure, M. P., Chingombe, I., Mugurungi, O., Hakim, A., Gozhora, P., Miller, S. S., Lamb, M. R., Samba, C., & Rogers, J. H. (2022). HIV care cascade and associated factors among men who have sex with men, transgender women, and genderqueer individuals in Zimbabwe: findings from a biobehavioural survey using respondent-driven sampling. The lancet. HIV, 9(3), e182–e201. https://doi.org/10.1016/S2352-3018(21)00297-6
204	Wilson Dib et al 2020	Wilson Dib, R., Dandachi, D., Matar, M., Shayya, A., Davila, J. A., Giordano, T. P., & Mokhbat, J. E. (2020). HIV in Lebanon: Reasons for Testing, Engagement in Care, and Outcomes in Patients with Newly Diagnosed HIV Infections. AIDS and behavior, 24(8), 2290–2298. https://doi.org/10.1007/s10461-020-02788-3

For peer review only - http://bmjopen.bmj.com/site/about/guidelines.xhtml

Paper Number	Authors	Reference
205	Magro et al 2021	Magro, P., Cerini, C., da Gloria, A., Tembe, S., Castelli, F., & Tomasoni, L. R. (2021). The cascade of care of HIV after one year of follow-up in a cohort of HIV-positive adult patients in three health settings of Morrumbene in rural Mozambique. Tropical medicine & international health : TM & IH, 26(11), 1503–1511. https://doi.org/10.1111/tmi.13671
206	Mathews et al 2021	Mathews, C., Cheyip, M., Beauclair, R., Puren, A., Lombard, C., Jonas, K., Ayalew, K. A., Govindasamy, D., Kuo, C., Dietrich, J., Abdullah, F., & Gray, G. (2021). HIV care coverage among HIV-positive adolescent girls and young women in South Africa: Results from the HERStory Study. South African medical journal = Suid-Afrikaanse tydskrif vir geneeskunde, 111(5), 460–468. https://doi.org/10.7196/SAMJ.2021.v111i5.15351
208	Lacombe-Duncan et al 2022	Lacombe-Duncan, A., Shokoohi, M., Persad, Y., Underhill, A., Machouf, N., Côté, P., Wheatley, M., Gupta, M., Kyne, L. T., Besharati, A. A., Fung, R., Chan, L. Y. L., Arbess, G., Bourns, A., Nguyen, Q., & Loutfy, M. (2022). Short report: Characterizing HIV care among a clinical sample of transgender women living with HIV. HIV medicine, 23(4), 324–330. https://doi.org/10.1111/hiv.13261
210	Gaolathe et al 2016	Gaolathe, T., Wirth, K. E., Holme, M. P., Makhema, J., Moyo, S., Chakalisa, U., Yankinda, E. K., Lei, Q., Mmalane, M., Novitsky, V., Okui, L., van Widenfelt, E., Powis, K. M., Khan, N., Bennett, K., Bussmann, H., Dryden-Peterson, S., Lebelonyane, R., El-Halabi, S., Mills, L. A., … Botswana Combination Prevention Project study team (2016). Botswana's progress toward achieving the 2020 UNAIDS 90-90-90 antiretroviral therapy and virological suppression goals: a population-based survey. The lancet. HIV, 3(5), e221–e230. https://doi.org/10.1016/S2352-3018(16)00037-0
211	Tlhajoane et al 2018	Tlhajoane, M., Eaton, J. W., Takaruza, A., Rhead, R., Maswera, R., Schur, N., Sherr, L., Nyamukapa, C., & Gregson, S. (2018). Prevalence and Associations of Psychological Distress, HIV Infection and HIV Care Service Utilization in East Zimbabwe. AIDS and behavior, 22(5), 1485–1495. https://doi.org/10.1007/s10461-017-1705-x
212	Eberhart et al 2013	Eberhart, M. G., Yehia, B. R., Hillier, A., Voytek, C. D., Blank, M. B., Frank, I., Metzger, D. S., & Brady, K. A. (2013). Behind the cascade: analyzing spatial patterns along the HIV care continuum. Journal of acquired immune deficiency syndromes (1999), 64 Suppl 1(0 1), S42–S51. https://doi.org/10.1097/QAI.0b013e3182a90112

For peer review only - http://bmjopen.bmj.com/site/about/guidelines.xhtml

Paper Number	Authors	Reference
213	Maughan-Brown et al 2020	Maughan-Brown, B., Beckett, S., Kharsany, A. B. M., Cawood, C., Khanyile, D., Lewis, L., Venkataramani, A., & George, G. (2021). Poor rates of linkage to HIV care and uptake of treatment after home-based HIV testing among newly diagnosed 15-to-49 year-old men and women in a high HIV prevalence setting in South Africa. AIDS care, 33(1), 70–79. https://doi.org/10.1080/09540121.2020.1719025
214	Chihana et al 2021	Chihana, M. L., Conan, N., Ellman, T., Poulet, E., Garone, D. B., Ortuno, R., Wanjala, S., Masiku, C., Etard, J. F., Davies, M. A., & Maman, D. (2021). The HIV cascade of care among serodiscordant couples in four high HIV prevalence settings in sub-Saharan Africa. South African medical journal = Suid-Afrikaanse tydskrif vir geneeskunde, 111(8), 768–776. https://doi.org/10.7196/SAMJ.2021.v111i8.15489
215	Tsondai et al 2020	Tsondai, P. R., Sohn, A. H., Phiri, S., Sikombe, K., Sawry, S., Chimbetete, C., Fatti, G., Hobbins, M. A., Technau, K. G., Rabie, H., Bernheimer, J., Fox, M. P., Judd, A., Collins, I. J., Davies, M. A., & International epidemiology to Evaluate AIDS Southern Africa (IeDEA-SA) Collaboration (2020). Characterizing the double-sided cascade of care for adolescents living with HIV transitioning to adulthood across Southern Africa. Journal of the International AIDS Society, 23(1), e25447. https://doi.org/10.1002/jia2.25447
216	Gibbs et al 2022	Gibbs, A., Reddy, T., Closson, K., Cawood, C., Khanyile, D., & Hatcher, A. (2022). Intimate Partner Violence and the HIV Care and Treatment Cascade Among Adolescent Girls and Young Women in DREAMS, South Africa. Journal of acquired immune deficiency syndromes (1999), 89(2), 136–142. https://doi.org/10.1097/QAI.0000000000002843
217	Lambdin et al 2017	Lambdin, B. H., Kral, A. H., Comfort, M., Lopez, A. M., & Lorvick, J. (2017). Associations of criminal justice and substance use treatment involvement with HIV/HCV testing and the HIV treatment cascade among people who use drugs in Oakland, California. Addiction science & clinical practice, 12(1), 13. https://doi.org/10.1186/s13722-017-0078-9
218	Bacha et al 2022	Bacha, J. M., Dlamini, S., Anabwani, F., Gwimile, J., Kanywa, J. B., Farirai, J., Bvumbwe, M., Steffy, T., Nguyen, D., Wanless, R. S., & Haq, H. (2022). Achieving Antiretroviral Therapy Uptake and Viral Suppression Among Children and Adolescents Living With HIV in the UNAIDS 90-90-90 Era Across Six Countries in Eastern and Southern Africa-Lessons From the BIPAI Network. Journal of acquired immune deficiency syndromes (1999), 90(3), 300–308. https://doi.org/10.1097/QAI.0000000000002957

For peer review only - http://bmjopen.bmj.com/site/about/guidelines.xhtml

Paper Number	Authors	Reference
219	Williams et al 2018	Williams, E. C., McGinnis, K. A., Edelman, E. J., Matson, T. E., Gordon, A. J., Marshall, B. D. L., Bryant, K. J., Rubinsky, A. D., Lapham, G. T., Satre, D. D., Richards, J. E., Catz, S. L., Fiellin, D. A., Justice, A. C., & Bradley, K. A. (2019). Level of Alcohol Use Associated with HIV Care Continuum Targets in a National U.S. Sample of Persons Living with HIV Receiving Healthcare. AIDS and behavior, 23(1), 140–151. https://doi.org/10.1007/s10461-018-2210-6
220	Mulholland et al 2021	Mulholland, G. E., Markiewicz, M., Arimi, P., Ssengooba, F., Weir, S., & Edwards, J. K. (2022). HIV Prevalence and the HIV Treatment Cascade Among Female Sex Workers in Cross-Border Areas in East Africa. AIDS and behavior, 26(2), 556–568. https://doi.org/10.1007/s10461-021-03411-9
221	Kalichman et al 2020	Kalichman, S. C., Banas, E., Katner, H., Hill, M., & Kalichman, M. O. (2020). Individual Social Capital and the HIV Continuum of Care in a Rural Setting of the Southeast United States. Rural mental health, 44(2), 75–86. https://doi.org/10.1037/rmh0000134
222	Hayes et al 2017	Hayes, R., Floyd, S., Schaap, A., Shanaube, K., Bock, P., Sabapathy, K., Griffith, S., Donnell, D., Piwowar-Manning, E., El-Sadr, W., Beyers, N., Ayles, H., Fidler, S., & HPTN 071 (PopART) Study Team (2017). A universal testing and treatment intervention to improve HIV control: One-year results from intervention communities in Zambia in the HPTN 071 (PopART) cluster-randomised trial. PLoS medicine, 14(5), e1002292. https://doi.org/10.1371/journal.pmed.1002292
223	King et al 2020	King, C., Giang, L. M., Bart, G., Kunkel, L., & Korthuis, P. T. (2020). HIV care continuum characteristics among people with opioid use disorder and HIV in Vietnam: baseline results from the BRAVO study. BMC public health, 20(1), 421. https://doi.org/10.1186/s12889-020-08538-3
224	Pascom et al 2018	Pascom, A. R. P., Meireles, M. V., & Benzaken, A. S. (2018). Sociodemographic determinants of attrition in the HIV continuum of care in Brazil, in 2016. Medicine, 97(1S Suppl 1), S69–S74. https://doi.org/10.1097/MD.0000000000009857
225	Chandler et al 2019	Chandler, C. J., Sang, J. M., Bukowski, L. A., Andrade, E., Eaton, L. A., Stall, R. D., & Matthews, D. D. (2019). Characterizing the HIV care continuum among a community sample of black men who have sex with men in the United States. AIDS care, 31(7), 816–820. https://doi.org/10.1080/09540121.2018.1549724
226	Gray et al 2014	Gray, K. M., Cohen, S. M., Hu, X., Li, J., Mermin, J., & Hall, H. I. (2014). Jurisdiction level differences in HIV diagnosis, retention in care, and viral suppression in the United States. Journal of acquired immune deficiency syndromes (1999), 65(2), 129–132. https://doi.org/10.1097/QAI.0000000000000028

For peer review only - http://bmjopen.bmj.com/site/about/guidelines.xhtml

Paper Number	Authors	Reference
227	Gokengin et al 2018	Gokengin, D., Oprea, C., Begovac, J., Horban, A., Zeka, A. N., Sedlacek, D., Allabergan, B., Almamedova, E. A., Balayan, T., Banhegyi, D., Bukovinova, P., Chkhartishvili, N., Damira, A., Deva, E., Elenkov, I., Gashi, L., Gexha-Bunjaku, D., Hadciosmanovic, V., Harxhi, A., Holban, T., … Yurin, O. (2018). HIV care in Central and Eastern Europe: How close are we to the target?. International journal of infectious diseases : IJID : official publication of the International Society for Infectious Diseases, 70, 121–130. https://doi.org/10.1016/j.ijid.2018.03.007
228	Whitham et al 2017	Whitham, H. K., Sansom, S. L., Wejnert, C., Finlayson, T., Huang, Y. A., An, Q., Paz-Bailey, G., & NHBS Study Group (2017). Sex Practices by HIV Awareness and Engagement in the Continuum of Care Among MSM: A National HIV Behavioral Surveillance Analysis in 21 U.S. Cities. AIDS and behavior, 22(3), 840–847. https://doi.org/10.1007/s10461-017-1966-4
229	Halperin et al 2019	Halperin, J., Conner, K., Butler, I., Zeng, P., Myers, L., Clark, R., & Van Sickels, N. (2019). A Care Continuum of Immediate ART for Newly Diagnosed Patients and Patients Presenting Later to Care at a Federally Qualified Health Center in New Orleans. Open forum infectious diseases, 6(4), ofz161. https://doi.org/10.1093/ofid/ofz161
231	Okeke et al 2016	Okeke, N., McFarland, W., & Raymond, H. F. (2016). Closing the Gap? The HIV Continuum in Care for African-American Men Who Have Sex with Men, San Francisco, 2004-2014. AIDS and behavior, 21(6), 1741–1744. https://doi.org/10.1007/s10461-016-1472-0
233	Lagi et al 2018	Lagi, F., Kiros, S. T., Campolmi, I., Giachè, S., Rogasi, P. G., Mazzetti, M., Bartalesi, F., Trotta, M., Nizzoli, P., Bartoloni, A., & Sterrantino, G. (2018). Continuum of care among HIV-1 positive patients in a single center in Italy (2007-2017). Patient preference and adherence, 12, 2545–2551. https://doi.org/10.2147/PPA.S180736
234	Smith et al 2015	Smith, L. R., Patterson, T. L., Magis-Rodriguez, C., Ojeda, V. D., Burgos, J. L., Rojas, S. A., Zúñiga, M. L., & Strathdee, S. A. (2015). Engagement in the HIV Care Continuum among Key Populations in Tijuana, Mexico. AIDS and behavior, 20(5), 1017–1025. https://doi.org/10.1007/s10461-015-1186-8
235	Hightow-Weidman et al 2017	Hightow-Weidman, L., LeGrand, S., Choi, S. K., Egger, J., Hurt, C. B., & Muessig, K. E. (2017). Exploring the HIV continuum of care among young black MSM. PloS one, 12(6), e0179688. https://doi.org/10.1371/journal.pone.0179688

For peer review only

For peer review only - http://bmjopen.bmj.com/site/about/guidelines.xhtml

Paper Number	Authors	Reference
237	Gonsalves et al 2017	Gonsalves, G. S., Paltiel, A. D., Cleary, P. D., Gill, M. J., Kitahata, M. M., Rebeiro, P. F., Silverberg, M. J., Horberg, M., Abraham, A. G., Althoff, K. N., Moore, R., Bosch, R. J., Tang, T., Hall, H. I., & Kaplan, E. H. (2017). A Flow-Based Model of the HIV Care Continuum in the United States. Journal of acquired immune deficiency syndromes (1999), 75(5), 548–553. https://doi.org/10.1097/QAI.0000000000001429
238	Hellenberg et al 2013	Helleberg, M., Häggblom, A., Sönnerborg, A., & Obel, N. (2013). HIV care in the Swedish-Danish HIV cohort 1995-2010, closing the gaps. PloS one, 8(8), e72257. https://doi.org/10.1371/journal.pone.0072257
240	Zhang et al 2016	Zhang, N., Bussell, S., Wang, G., Zhu, X., Yang, X., Huang, T., Qian, Y., Tao, X., Kang, D., & Wang, N. (2016). Disparities in HIV Care Along the Path From Infection to Viral Suppression: A Cross-sectional Study of HIV/AIDS Patient Records in 2013, Shandong Province, China. Clinical infectious diseases : an official publication of the Infectious Diseases Society of America, 63(1), 115–121. https://doi.org/10.1093/cid/ciw190
242	Xia et al 2016	Xia, Q., Shah, D., Gill, B., Torian, L. V., & Braunstein, S. L. (2016). Continuum of Care Among People Living with Perinatally Acquired HIV Infection in New York City, 2014. Public health reports (Washington, D.C. : 1974), 131(4), 566–573. https://doi.org/10.1177/0033354916662215
243	Kapogiannis et al 2020	Kapogiannis, B. G., Koenig, L. J., Xu, J., Mayer, K. H., Loeb, J., Greenberg, L., Monte, D., Banks-Shields, M., Fortenberry, J. D., & Adolescent Medicine Trials Network for HIV/AIDS Interventions (2020). The HIV Continuum of Care for Adolescents and Young Adults Attending 13 Urban US HIV Care Centers of the NICHD-ATN-CDC-HRSA SMILE Collaborative. Journal of acquired immune deficiency syndromes (1999), 84(1), 92–100. https://doi.org/10.1097/QAI.0000000000002308
244	Rajabiun et al 2018	Rajabiun, S., Tryon, J., Feaster, M., Pan, A., McKeithan, L., Fortu, K., Cabral, H. J., Borne, D., & Altice, F. L. (2018). The Influence of Housing Status on the HIV Continuum of Care: Results From a Multisite Study of Patient Navigation Models to Build a Medical Home for People Living With HIV Experiencing Homelessness. American journal of public health, 108(S7), S539–S545. https://doi.org/10.2105/AJPH.2018.304736
246	Beckwith et al 2018	Beckwith, C. G., Kuo, I., Fredericksen, R. J., Brinkley-Rubinstein, L., Cunningham, W. E., Springer, S. A., Loeliger, K. B., Franks, J., Christopoulos, K., Lorvick, J., Kahana, S. Y., Young, R., Seal, D. W., Zawitz, C., Delaney, J. A., Crane, H. M., & Biggs, M. L. (2018). Risk behaviors and HIV care continuum outcomes among criminal justice-involved HIV-infected transgender women and cisgender men: Data from the Seek, Test, Treat, and Retain Harmonization Initiative. PloS one,

For peer review only - http://bmjopen.bmj.com/site/about/guidelines.xhtml

Paper Number	Authors	Reference
		13(5), e0197730. https://doi.org/10.1371/journal.pone.0197730
249	Xia et al 2016	Xia, Q., Lazar, R., Bernard, M. A., McNamee, P., Daskalakis, D. C., Torian, L. V., & Braunstein, S. L. (2016). New York City Achieves the UNAIDS 90-90-90 Targets for HIV-Infected Whites but Not Latinos/Hispanics and Blacks. Journal of acquired immune deficiency syndromes (1999), 73(3), e59–e62. https://doi.org/10.1097/QAI.0000000000001132
250	Skarbinski et al 2015	Skarbinski, J., Rosenberg, E., Paz-Bailey, G., Hall, H. I., Rose, C. E., Viall, A. H., Fagan, J. L., Lansky, A., & Mermin, J. H. (2015). Human immunodeficiency virus transmission at each step of the care continuum in the United States. JAMA internal medicine, 175(4), 588–596. https://doi.org/10.1001/jamainternmed.2014.8180
251	Vallecillo et al 2020	Vallecillo, G., Fonseca, F., Marín, G. et al. Reaching the 90–90–90 UNAIDS treatment target for people who inject drugs receiving integrated clinical care at a drug-use outpatient treatment facility. J Public Health (Berl.) 30, 481–486 (2022). https://doi.org/10.1007/s10389-020-01298-9
252	Tan et al 2016	Tan, J. Y., Pollack, L., Rebchook, G., Peterson, J., Huebner, D., Eke, A., Johnson, W., & Kegeles, S. (2016). The Role of the Primary Romantic Relationship in HIV Care Engagement Outcomes Among Young HIV-Positive Black Men Who Have Sex with Men. AIDS and behavior, 22(3), 774–790. https://doi.org/10.1007/s10461-016-1601-9
253	Anderson et al 2017	Anderson, E. A., Momplaisir, F. M., Corson, C., & Brady, K. A. (2017). Assessing the Impact of Perinatal HIV Case Management on Outcomes Along the HIV Care Continuum for Pregnant and Postpartum Women Living With HIV, Philadelphia 2005-2013. AIDS and behavior, 21(9), 2670–2681. https://doi.org/10.1007/s10461-017-1714-9
254	Wong et al 2017	Wong, N. S., Mao, J., Cheng, W., Tang, W., Cohen, M. S., Tucker, J. D., & Xu, H. (2018). HIV Linkage to Care and Retention in Care Rate Among MSM in Guangzhou, China. AIDS and behavior, 22(3), 701–710. https://doi.org/10.1007/s10461-017-1893-4
255	Terzian et al 2018	Terzian, A. S., Younes, N., Greenberg, A. E., Opoku, J., Hubbard, J., Happ, L. P., Kumar, P., Jones, R. R., Castel, A. D., & DC Cohort Executive Committee (2018). Identifying Spatial Variation Along the HIV Care Continuum: The Role of Distance to Care on Retention

For peer review only - http://bmjopen.bmj.com/site/about/guidelines.xhtml

Paper Number	Authors	Reference
		and Viral Suppression. AIDS and behavior, 22(9), 3009–3023. https://doi.org/10.1007/s10461-018-2103-8
256	Takada et al 2019	Takada, S., Ettner, S. L., Harawa, N. T., Garland, W. H., Shoptaw, S. J., & Cunningham, W. E. (2019). Life Chaos is Associated with Reduced HIV Testing, Engagement in Care, and ART Adherence Among Cisgender Men and Transgender Women upon Entry into Jail. AIDS and behavior, 24(2), 491–505. https://doi.org/10.1007/s10461-019-02570-0
257	Mimiaga et al 2019	Mimiaga, M. J., August Oddleifson, D., Meersman, S. C., Silvia, A., Hughto, J. M. W., Landers, S., Brown, E., & Loberti, P. (2019). Multilevel Barriers to Engagement in the HIV Care Continuum Among Residents of the State of Rhode Island Living with HIV. AIDS and behavior, 24(4), 1133–1150. https://doi.org/10.1007/s10461-019-02677-4
258	Abia et al 2020	Abia, A., Rothman, R. E., Mohareb, A. M., Lim, G. L. H., Patel, A. V., Bigelow, B., Klein, E. Y., Cole, G., Gebo, K. A., Moore, R. D., & Hsieh, Y. H. (2020). Resource utilization across the continuum of HIV care: An emergency department-based cohort study. The American journal of emergency medicine, 43, 164–169. https://doi.org/10.1016/j.ajem.2020.02.037
259	Hsieh et al 2015	Hsieh, Y. H., Kelen, G. D., Laeyendecker, O., Kraus, C. K., Quinn, T. C., & Rothman, R. E. (2015). HIV Care Continuum for HIV-Infected Emergency Department Patients in an Inner-City Academic Emergency Department. Annals of emergency medicine, 66(1), 69–78. https://doi.org/10.1016/j.annemergmed.2015.01.001
260	Jin et al 2018	Jin, H., Ogunbajo, A., Mimiaga, M. J., Duncan, D. T., Boyer, E., Chai, P., Dilworth, S. E., & Carrico, A. W. (2018). Over the influence: The HIV care continuum among methamphetamine-using men who have sex with men. Drug and alcohol dependence, 192, 125–128. https://doi.org/10.1016/j.drugalcdep.2018.07.038
261	Satre et al 2021	Satre, D. D., Levine-Hall, T., Sterling, S. A., Young-Wolff, K. C., Lam, J. O., Alexeeff, S., Hojilla, J. C., Williams, A., Justice, A. C., Sterne, J., Cavassini, M., Bryant, K. J., Williams, E. C., Horberg, M. A., Volberding, P., Weisner, C., & Silverberg, M. J. (2021). The relationship of smoking and unhealthy alcohol use to the HIV care continuum among people with HIV in an integrated health care system. Drug and alcohol dependence, 219, 108481. https://doi.org/10.1016/j.drugalcdep.2020.108481

For peer review only

For peer review only - http://bmjopen.bmj.com/site/about/guidelines.xhtml

Paper Number	Authors	Reference
262	Nwangu-ike et al 2015	Nwangwu-Ike, N., Hernandez, A. L., An, Q., Huang, T., & Hall, H. I. (2015). The Epidemiology of Human Immunodeficiency Virus Infection and Care among Adult and Adolescent Females in the United States, 2008-2012. Women's health issues : official publication of the Jacobs Institute of Women's Health, 25(6), 711–719. https://doi.org/10.1016/j.whi.2015.07.004
264	Parchure et al 2015	Parchure, R., Kulkarni, V., Kulkarni, S., & Gangakhedkar, R. (2015). Pattern of linkage and retention in HIV care continuum among patients attending referral HIV care clinic in private sector in India. AIDS care, 27(6), 716–722. https://doi.org/10.1080/09540121.2014.996518
265	Yoon et al 2017	Yoon, I. S., Downing, M. J., Jr, Teran, R., Chiasson, M. A., Houang, S. T., Parsons, J. T., & Hirshfield, S. (2017). Sexual risk taking and the HIV care continuum in an online sample of men who have sex with men. AIDS care, 30(7), 921–929. https://doi.org/10.1080/09540121.2017.1417535
266	Takada et al 2020	Takada, S., Gorbach, P., Brookmeyer, R., & Shoptaw, S. (2021). Associations of social capital resources and experiences of homophobia with HIV transmission risk behavior and HIV care continuum among men who have sex with men in Los Angeles. AIDS care, 33(5), 663–674. https://doi.org/10.1080/09540121.2020.1828798
267	Morgan et al 2016	Morgan, E., Khanna, A. S., Skaathun, B., Michaels, S., Young, L., Duvoisin, R., Chang, M., Voisin, D., Cornwell, B., Coombs, R. W., Friedman, S. R., & Schneider, J. A. (2016). Marijuana Use Among Young Black Men Who Have Sex With Men and the HIV Care Continuum: Findings From the uConnect Cohort. Substance use & misuse, 51(13), 1751–1759. https://doi.org/10.1080/10826084.2016.1197265
268	Rowan et al 2014	Rowan, S. E., Burman, W. J., Johnson, S. C., Connick, E., Reirden, D., Daniloff, E., & Gardner, E. M. (2014). Engagement-in-care during the first 5 years after HIV diagnosis: data from a cohort of newly HIV-diagnosed individuals in a large US city. AIDS patient care and STDs, 28(9), 475–482. https://doi.org/10.1089/apc.2013.0340
269	Almirol et al 2018	Almirol, E. A., McNulty, M. C., Schmitt, J., Eavou, R., Taylor, M., Tobin, A., Ramirez, K., Glick, N., Stamos, M., Schuette, S., Ridgway, J. P., & Pitrak, D. (2018). Gender Differences in HIV Testing, Diagnosis, and Linkage to Care in Healthcare Settings: Identifying African American Women with HIV in Chicago. AIDS patient care and STDs, 32(10), 399–407. https://doi.org/10.1089/apc.2018.0066
270	Parcesepe et al 2020	Parcesepe, A. M., Lahuerta, M., Lamb, M. R., Ahoua, L., Abacassamo, F., & Elul, B. (2020). Household Decision-Making and HIV Care Continuum Outcomes Among Women Living with HIV in Mozambique. AIDS patient

For peer review only - http://bmjopen.bmj.com/site/about/guidelines.xhtml

Paper Number	Authors	Reference
		care and STDs, 34(4), 173–183. https://doi.org/10.1089/apc.2019.0268
271	Desir et al 2018	Desir, F. A., Lesko, C. R., Moore, R. D., Horberg, M. A., Wong, C., Crane, H. M., Silverberg, M., Thorne, J. E., Rachlis, B., Rabkin, C., Mayor, A. M., Mathews, W. C., Althoff, K. N., & North American AIDS Cohort Collaboration on Research and Design (NA-ACCORD) Region of the International Epidemiologic Databases to Evaluate AIDS (IeDEA) Consortium (2018). One Size Fits (n)One: The Influence of Sex, Age, and Sexual Human Immunodeficiency Virus (HIV) Acquisition Risk on Racial/Ethnic Disparities in the HIV Care Continuum in the United States. Clinical infectious diseases : an official publication of the Infectious Diseases Society of America, 68(5), 795–802. https://doi.org/10.1093/cid/ciy556
272	Cuzin et al 2021	Cuzin, L., Allavena, C., Cotte, L., Delpierre, C., Huleux, T., Palich, R., Delobel, P., Raffi, F., Cabié, A., & Dat'AIDS Study Group (2021). No barrier to care, yet disparities in the HIV care continuum in France: a nationwide population study. The Journal of antimicrobial chemotherapy, 76(6), 1573–1579. https://doi.org/10.1093/jac/dkab061
273	Lesko et al 2016	Lesko, C. R., Edwards, J. K., Moore, R. D., & Lau, B. (2016). A longitudinal, HIV care continuum: 10-year restricted mean time in each care continuum stage after enrollment in care, by history of IDU. AIDS (London, England), 30(14), 2227–2234. https://doi.org/10.1097/QAD.0000000000001183
274	Schneider et al 2017	Schneider, J. A., Kozloski, M., Michaels, S., Skaathun, B., Voisin, D., Lancki, N., Morgan, E., Khanna, A., Green, K., Coombs, R. W., Friedman, S. R., Laumann, E., Schumm, P., & uConnectand BARS study teams (2017). Criminal justice involvement history is associated with better HIV care continuum metrics among a population-based sample of young black MSM. AIDS (London, England), 31(1), 159–165. https://doi.org/10.1097/QAD.0000000000001269
275	Matson et al 2018	Matson, T. E., McGinnis, K. A., Rubinsky, A. D., Frost, M. C., Czarnogorski, M., Bryant, K. J., Edelman, E. J., Satre, D. D., Catz, S. L., Bensley, K. M., Fiellin, D. A., Justice, A. C., & Williams, E. C. (2018). Gender and alcohol use: influences on HIV care continuum in a national cohort of patients with HIV. AIDS (London, England), 32(15), 2247–2253. https://doi.org/10.1097/QAD.0000000000001946

For peer review only

For peer review only - http://bmjopen.bmj.com/site/about/guidelines.xhtml

Paper Number	Authors	Reference
276	Chen et al 2019	Chen, J. S., Pence, B. W., Rahangdale, L., Patterson, K. B., Farel, C. E., Durr, A. L., Antono, A. C., Zakharova, O., Eron, J. J., & Napravnik, S. (2019). Postpartum HIV care continuum outcomes in the southeastern USA. AIDS (London, England), 33(4), 637–644. https://doi.org/10.1097/QAD.0000000000002094
277	Nelson et al 2016	Nelson, J. A., Kinder, A., Johnson, A. S., Hall, H. I., Hu, X., Sweet, D., Guido, A., Katner, H., Janelle, J., Gonzalez, M., Paz, N. M., Ledonne, C., Henry, J., Bramel, T., & Harris, J. (2018). Differences in Selected HIV Care Continuum Outcomes Among People Residing in Rural, Urban, and Metropolitan Areas-28 US Jurisdictions. The Journal of rural health : official journal of the American Rural Health Association and the National Rural Health Care Association, 34(1), 63–70. https://doi.org/10.1111/jrh.12208
278	Wirtz et al 2015	Wirtz, A. L., Zelaya, C. E., Latkin, C., Peryshkina, A., Galai, N., Mogilniy, V., Dzhigun, P., Kostetskaya, I., Mehta, S. H., & Beyrer, C. (2015). The HIV care continuum among men who have sex with men in Moscow, Russia: a cross-sectional study of infection awareness and engagement in care. Sexually transmitted infections, 92(2), 161–167. https://doi.org/10.1136/sextrans-2015-052076
279	Mitsch et al 2016	Mitsch, A., Surendera Babu, A., Seneca, D., Whiteside, Y. O., & Warne, D. (2016). HIV care and treatment of American Indians/Alaska natives with diagnosed HIV infection - 27 states and the District of Columbia, 2012. International journal of STD & AIDS, 28(10), 953–961. https://doi.org/10.1177/0956462416681183
280	Van Beckhoven et al 2015	Van Beckhoven, D., Florence, E., Ruelle, J., Deblonde, J., Verhofstede, C., Callens, S., Vancutsem, E., Lacor, P., Demeester, R., Goffard, J. C., Sasse, A., & BREACH Belgian Research on AIDS and HIV Consortium (2015). Good continuum of HIV care in Belgium despite weaknesses in retention and linkage to care among migrants. BMC infectious diseases, 15, 496. https://doi.org/10.1186/s12879-015-1230-3
282	Ntombela et al 2022	Ntombela, N. P., Kharsany, A. B. M., Soogun, A., Yende-Zuma, N., Baxter, C., Kohler, H. P., & McKinnon, L. R. (2022). Viral suppression among pregnant adolescents and women living with HIV in rural KwaZulu-Natal, South Africa: a cross sectional study to assess progress towards UNAIDS indicators and Implications for HIV Epidemic Control. Reproductive health, 19(1), 116. https://doi.org/10.1186/s12978-022-01419-5
283	Lindman et al 2020	Lindman, J., Djalo, M. A., Biai, A., Månsson, F., Esbjörnsson, J., Jansson, M., Medstrand, P., Norrgren, H., & SWEGUB CORE group (2020). The HIV care continuum and HIV-1 drug resistance among female sex workers: a key population in Guinea-Bissau. AIDS

For peer review only - http://bmjopen.bmj.com/site/about/guidelines.xhtml

Paper Number	Authors	Reference
		research and therapy, 17(1), 33. https://doi.org/10.1186/s12981-020-00290-3
284	Mcnairy et al 2017	McNairy, M. L., Lamb, M. R., Gachuhi, A. B., Nuwagaba-Biribonwoha, H., Burke, S., Mazibuko, S., Okello, V., Ehrenkranz, P., Sahabo, R., & El-Sadr, W. M. (2017). Effectiveness of a combination strategy for linkage and retention in adult HIV care in Swaziland: The Link4Health cluster randomized trial. PLoS medicine, 14(11), e1002420. https://doi.org/10.1371/journal.pmed.1002420
285	Shade et al 2021	Shade, S. B., Kirby, V. B., Stephens, S., Moran, L., Charlebois, E. D., Xavier, J., Cajina, A., Steward, W. T., & Myers, J. J. (2021). Outcomes and costs of publicly funded patient navigation interventions to enhance HIV care continuum outcomes in the United States: A before-and-after study. PLoS medicine, 18(5), e1003418. https://doi.org/10.1371/journal.pmed.1003418
286	Dailey et al 2017	Dailey, A. F., Johnson, A. S., & Wu, B. (2017). HIV Care Outcomes Among Blacks with Diagnosed HIV - United States, 2014. MMWR. Morbidity and mortality weekly report, 66(4), 97–103. https://doi.org/10.15585/mmwr.mm6604a2
287	Harxhi et al 2020	Harxhi, A., Vrapi, E., Gjataj, A., Meta, E., Simaku, A., Bani, R., ... & Youle, M. HIV care cascade in Albania: analysis of newly diagnosed cases in 2016. HIV & AIDS Review. International Journal of HIV-Related Problems, 19(4), 267-272.
288	Alvarezi - Uria et al 2013	Alvarez-Uria, G., Pakam, R., Midde, M., & Naik, P. K. (2013). Entry, Retention, and Virological Suppression in an HIV Cohort Study in India: Description of the Cascade of Care and Implications for Reducing HIV-Related Mortality in Low- and Middle-Income Countries. Interdisciplinary perspectives on infectious diseases, 2013, 384805. https://doi.org/10.1155/2013/384805
289	Gant et al 2014	Gant, Z., Bradley, H., Hu, X., Skarbinski, J., Hall, H. I., Lansky, A., & Centers for Disease Control and Prevention (CDC) (2014). Hispanics or Latinos living with diagnosed HIV: progress along the continuum of HIV care - United States, 2010. MMWR. Morbidity and mortality weekly report, 63(40), 886–890.
290	Shi et al 2021	Shi, L., Tang, W., Hu, H., Qiu, T., Marley, G., Liu, X., Chen, Y., Chen, Y., & Fu, G. (2021). The impact of COVID-19 pandemic on HIV care continuum in Jiangsu, China. BMC infectious diseases, 21(1), 768. https://doi.org/10.1186/s12879-021-06490-0

For peer review only - http://bmjopen.bmj.com/site/about/guidelines.xhtml

Paper Number	Authors	Reference
292	Greene et al 2018	Greene, R. E., Luong, A., Barton, S. C., Kapadia, F., & Halkitis, P. N. (2018). Assessing Gaps in the HIV Care Continuum in Young Men Who Have Sex With Men: The P18 Cohort Study. The Journal of the Association of Nurses in AIDS Care : JANAC, 29(3), 475–478. https://doi.org/10.1016/j.jana.2017.12.004
295	Kilcrease et al 2019	Kilcrease, C., Miller, M. M., Neely, S., & Liedtke, M. D. (2020). Pharmacist impact on the HIV Care Continuum: Decreasing time to care. Journal of the American College of Clinical Pharmacy, 3(3), 586-592.
296	Ndori-mharadze et al 2018	Ndori-Mharadze, T., Fearon, E., Busza, J., Dirawo, J., Musemburi, S., Davey, C., Acharya, X., Mtetwa, S., Hargreaves, J. R., & Cowan, F. (2018). Changes in engagement in HIV prevention and care services among female sex workers during intensified community mobilization in 3 sites in Zimbabwe, 2011 to 2015. Journal of the International AIDS Society, 21 Suppl 5(Suppl Suppl 5), e25138. https://doi.org/10.1002/jia2.25138
297	Baguso et al 2019	Baguso, G. N., Turner, C. M., Santos, G. M., Raymond, H. F., Dawson-Rose, C., Lin, J., & Wilson, E. C. (2019). Successes and final challenges along the HIV care continuum with transwomen in San Francisco. Journal of the International AIDS Society, 22(4), e25270. https://doi.org/10.1002/jia2.25270
298	Bhattacharjee et al 2020	Bhattacharjee, P., Isac, S., Musyoki, H., Emmanuel, F., Olango, K., Kuria, S., Ongaro, M. K., Walimbwa, J., Musimbi, J., Mugambi, M., Kaosa, S., Kioko, J., Njraini, M., Melon, M., Onyoni, J., Bartilol, K., Becker, M., Lorway, R., Pickles, M., Moses, S., … Mishra, S. (2020). HIV prevalence, testing and treatment among men who have sex with men through engagement in virtual sexual networks in Kenya: a cross-sectional bio-behavioural study. Journal of the International AIDS Society, 23 Suppl 2(Suppl 2), e25516. https://doi.org/10.1002/jia2.25516
299	Ramadhani et al 2020	Ramadhani, H. O., Crowell, T. A., Nowak, R. G., Ndembi, N., Kayode, B. O., Kokogho, A., Ononaku, U., Shoyemi, E., Ekeh, C., Adebajo, S., Baral, S. D., & Charurat, M. E. (2020). Association of age with healthcare needs and engagement among Nigerian men who have sex with men and transgender women: cross-sectional and longitudinal analyses from an observational cohort. Journal of the International AIDS Society, 23 Suppl 6(Suppl 6), e25599. https://doi.org/10.1002/jia2.25599
300	Yohannes et al 2021	Yohannes, N. T., Jenkins, C. A., Clouse, K., Cortés, C. P., Mejía Cordero, F., Padgett, D., Rouzier, V., Friedman, R. K., McGowan, C. C., Shepherd, B. E., & Rebeiro, P. F. (2021). Timing of HIV diagnosis relative to pregnancy and postpartum HIV care continuum outcomes among Latin American women, 2000 to 2017. Journal of the International AIDS Society, 24(5), e25740. https://doi.org/10.1002/jia2.25740

For peer review only - http://bmjopen.bmj.com/site/about/guidelines.xhtml

Paper Number	Authors	Reference
301	Lesko et al 2016	Lesko, C. R., Tong, W., Moore, R. D., & Lau, B. (2016). Retention, Antiretroviral Therapy Use and Viral Suppression by History of Injection Drug Use Among HIV-Infected Patients in an Urban HIV Clinical Cohort. AIDS and behavior, 21(4), 1016–1024. https://doi.org/10.1007/s10461-016-1585-5
302	Zhang et al 2017	Zhang, J., Xu, J. J., Song, W., Pan, S., Chu, Z. X., Hu, Q. H., Yu, H., Mao, X., Jiang, Y. J., Geng, W. Q., Shang, H., & Wang, N. (2018). HIV Incidence and Care Linkage among MSM First-Time-Testers in Shenyang, China 2012-2014. AIDS and behavior, 22(3), 711–721. https://doi.org/10.1007/s10461-017-1840-4
304	Norwood et al 2022	Norwood, J., Kheshti, A., Shepherd, B. E., Rebeiro, P. F., Ahonkhai, A., Kelly, S., & Wanjalla, C. (2022). The Impact of COVID-19 on the HIV Care Continuum in a Large Urban Southern Clinic. AIDS and behavior, 26(8), 2825–2829. https://doi.org/10.1007/s10461-022-03615-7
305	Brewer et al 2020	Brewer, R., Hood, K. B., Hotton, A., Moore, M., Spieldenner, A., Daunis, C., Mukherjee, S., Sprague, L., Schneider, J. A., Smith-Davis, M., Brown, G., & Bowen, B. (2022). Associations Between Experienced HIV Stigma, Resulting Consequences, and the HIV Care Continuum: Moderating Effects of Two Resilience Characteristics Among Persons Living with HIV (PLWH) in Louisiana. Journal of racial and ethnic health disparities, 9(1), 9–22. https://doi.org/10.1007/s40615-020-00925-1
306	Genberg et al 2015	Genberg, B. L., Naanyu, V., Wachira, J., Hogan, J. W., Sang, E., Nyambura, M., Odawa, M., Duefield, C., Ndege, S., & Braitstein, P. (2015). Linkage to and engagement in HIV care in western Kenya: an observational study using population-based estimates from home-based counselling and testing. The lancet. HIV, 2(1), e20–e26. https://doi.org/10.1016/S2352-3018(14)00034-4
307	Jose et al 2018	Jose, S., Delpech, V., Howarth, A., Burns, F., Hill, T., Porter, K., Sabin, C. A., & UK CHIC Study Steering Committee (2018). A continuum of HIV care describing mortality and loss to follow-up: a longitudinal cohort study. The lancet. HIV, 5(6), e301–e308. https://doi.org/10.1016/S2352-3018(18)30048-1
308	Steiner et al 2020	Steiner, C., MacKellar, D., Cham, H. J., Rwabiyago, O. E., Maruyama, H., Msumi, O., Pals, S., Weber, R., Kundi, G., Byrd, J., Kazaura, K., Madevu-Matson, C., Morales, F., Justman, J., Rutachunzibwa, T., & Rwebembera, A. (2020). Community-wide HIV testing, linkage case management, and defaulter tracing in Bukoba, Tanzania: pre-intervention and post-intervention, population-based survey evaluation. The lancet. HIV, 7(10), e699–e710. https://doi.org/10.1016/S2352-3018(20)30199-5

For peer review only - http://bmjopen.bmj.com/site/about/guidelines.xhtml

Paper Number	Authors	Reference
310	Kouyoumdjian et al 2019	Kouyoumdjian, F. G., Lamarche, L., McCormack, D., Rowe, J., Kiefer, L., Kroch, A., & Antoniou, T. (2020). 90-90-90 for everyone?: Access to HIV care and treatment for people with HIV who experience imprisonment in Ontario, Canada. AIDS care, 32(9), 1168–1176. https://doi.org/10.1080/09540121.2019.1679710
312	Ross et al 2017	Ross, J., Felsen, U. R., Cunningham, C. O., Patel, V. V., & Hanna, D. B. (2017). Outcomes Along the HIV Care Continuum Among Undocumented Immigrants in Clinical Care. AIDS research and human retroviruses, 33(10), 1038–1044. https://doi.org/10.1089/AID.2017.0015
313	Tymejczyk et al 2018	Tymejczyk, O., Jamison, K., Pathela, P., Braunstein, S., Schillinger, J. A., & Nash, D. (2018). HIV Care and Viral Load Suppression After Sexual Health Clinic Visits by Out-of-Care HIV-Positive Persons. AIDS patient care and STDs, 32(10), 390–398. https://doi.org/10.1089/apc.2018.0097
314	Raj et al 2018	Raj, A., Yore, J., Urada, L., Triplett, D. P., Vaida, F., & Smith, L. R. (2018). Multi-Site Evaluation of Community-Based Efforts to Improve Engagement in HIV Care Among Populations Disproportionately Affected by HIV in the United States. AIDS patient care and STDs, 32(11), 438–449. https://doi.org/10.1089/apc.2018.0128
315	Doshi et al 2014	Doshi, R. K., Milberg, J., Isenberg, D., Matthews, T., Malitz, F., Matosky, M., Trent-Adams, S., Parham Hopson, D., & Cheever, L. W. (2014). High rates of retention and viral suppression in the US HIV safety net system: HIV care continuum in the Ryan White HIV/AIDS Program, 2011. Clinical infectious diseases : an official publication of the Infectious Diseases Society of America, 60(1), 117–125. https://doi.org/10.1093/cid/ciu722
316	Mehta et al 2015	Mehta, S. H., Lucas, G. M., Solomon, S., Srikrishnan, A. K., McFall, A. M., Dhingra, N., Nandagopal, P., Kumar, M. S., Celentano, D. D., & Solomon, S. S. (2015). HIV care continuum among men who have sex with men and persons who inject drugs in India: barriers to successful engagement. Clinical infectious diseases : an official publication of the Infectious Diseases Society of America, 61(11), 1732–1741. https://doi.org/10.1093/cid/civ669
317	Adams et al 2015	Adams, J. W., Brady, K. A., Michael, Y. L., Yehia, B. R., & Momplaisir, F. M. (2015). Postpartum Engagement in HIV Care: An Important Predictor of Long-term Retention in Care and Viral Suppression. Clinical infectious diseases : an official publication of the Infectious Diseases Society of America, 61(12), 1880–1887. https://doi.org/10.1093/cid/civ678

For peer review only - http://bmjopen.bmj.com/site/about/guidelines.xhtml

Paper Number	Authors	Reference
318	Philbin et al 2019	Philbin, M. M., Feaster, D. J., Gooden, L., Duan, R., Das, M., Jacobs, P., Lucas, G. M., Batey, D. S., Nijhawan, A., Jacobson, J. M., Mandler, R., Daar, E., McMahon, D. K., Armstrong, W. S., Del Rio, C., & Metsch, L. R. (2019). The North-South Divide: Substance Use Risk, Care Engagement, and Viral Suppression Among Hospitalized Human Immunodeficiency Virus-Infected Patients in 11 US Cities. Clinical infectious diseases : an official publication of the Infectious Diseases Society of America, 68(1), 146–149. https://doi.org/10.1093/cid/ciy506
319	Summers et al 2021	Summers, N. A., Huynh, T. T., Dunn, R. C., Cross, S. L., & Fuchs, C. J. (2021). Effects of Gender-Affirming Hormone Therapy on Progression Along the HIV Care Continuum in Transgender Women. Open forum infectious diseases, 8(9), ofab404. https://doi.org/10.1093/ofid/ofab404
320	Schechter et al 2018	Schechter, M. C., Bizune, D., Kagei, M., Holland, D. P., Del Rio, C., Yamin, A., Mohamed, O., Oladele, A., Wang, Y. F., Rebolledo, P. A., Ray, S. M., & Kempker, R. R. (2018). Challenges Across the HIV Care Continuum for Patients With HIV/TB Co-infection in Atlanta, GA [corrected]. Open forum infectious diseases, 5(4), ofy063. https://doi.org/10.1093/ofid/ofy063
321	Haachambwa et al 2019	Haachambwa, L., Kandiwo, N., Zulu, P. M., Rutagwera, D., Geng, E., Holmes, C. B., Sinkala, E., Claassen, C. W., Mugavero, M. J., Wa Mwanza, M., Turan, J. M., & Vinikoor, M. J. (2019). Care Continuum and Postdischarge Outcomes Among HIV-Infected Adults Admitted to the Hospital in Zambia. Open forum infectious diseases, 6(10), ofz336. https://doi.org/10.1093/ofid/ofz336
322	Luo et al 2019	Luo, Y., Wu, H., Zhang, X. L., Li, X. T., Scott, S. R., Chen, J. F., & Wu, Z. Y. (2019). HIV care continuum among newly diagnosed student and non-student youths between 2012 and 2016 in Hangzhou, China. Chinese medical journal, 132(12), 1420–1428. https://doi.org/10.1097/CM9.0000000000000264
323	Katz et al 2019	Katz, I. T., Bogart, L. M., Dietrich, J. J., Leslie, H. H., Iyer, H. S., Leone, D., Magidson, J. F., Earnshaw, V. A., Courtney, I., Tshabalala, G., Fitzmaurice, G. M., Orrell, C., Gray, G., & Bangsberg, D. R. (2019). Understanding the role of resilience resources, antiretroviral therapy initiation, and HIV-1 RNA suppression among people living with HIV in South Africa: a prospective cohort study. AIDS (London, England), 33 Suppl 1(Suppl 1), S71–S79. https://doi.org/10.1097/QAD.0000000000002175
324	Dombrowski et al 2012	Dombrowski, J. C., Kent, J. B., Buskin, S. E., Stekler, J. D., & Golden, M. R. (2012). Population-based metrics for the timing of HIV diagnosis, engagement in HIV care, and virologic suppression. AIDS (London, England), 26(1), 77–86. https://doi.org/10.1097/QAD.0b013e32834dcee9

For peer review only - http://bmjopen.bmj.com/site/about/guidelines.xhtml

Paper Number	Authors	Reference
325	Dombrowski et al 2014	Dombrowski, J. C., Buskin, S. E., Bennett, A., Thiede, H., & Golden, M. R. (2014). Use of multiple data sources and individual case investigation to refine surveillance-based estimates of the HIV care continuum. Journal of acquired immune deficiency syndromes (1999), 67(3), 323–330. https://doi.org/10.1097/QAI.0000000000000302
326	Wiewel et al 2015	Wiewel, E. W., Braunstein, S. L., Xia, Q., Shepard, C. W., & Torian, L. V. (2015). Monitoring outcomes for newly diagnosed and prevalent HIV cases using a care continuum created with New York city surveillance data. Journal of acquired immune deficiency syndromes (1999), 68(2), 217–226. https://doi.org/10.1097/QAI.0000000000000424
327	Zulliger et al 2015	Zulliger, R., Barrington, C., Donastorg, Y., Perez, M., & Kerrigan, D. (2015). High Drop-off Along the HIV Care Continuum and ART Interruption Among Female Sex Workers in the Dominican Republic. Journal of acquired immune deficiency syndromes (1999), 69(2), 216–222. https://doi.org/10.1097/QAI.0000000000000590
328	Backus et al 2015	Backus, L., Czarnogorski, M., Yip, G., Thomas, B. P., Torres, M., Bell, T., & Ross, D. (2015). HIV Care Continuum Applied to the US Department of Veterans Affairs: HIV Virologic Outcomes in an Integrated Health Care System. Journal of acquired immune deficiency syndromes (1999), 69(4), 474–480. https://doi.org/10.1097/QAI.0000000000000615
330	Patrick et al 2017	Patrick, R., Greenberg, A., Magnus, M., Opoku, J., Kharfen, M., & Kuo, I. (2017). Development of an HIV Testing Dashboard to Complement the HIV Care Continuum Among MSM, PWID, and Heterosexuals in Washington, DC, 2007-2015. Journal of acquired immune deficiency syndromes (1999), 75 Suppl 3(Suppl 3), S397–S407. https://doi.org/10.1097/QAI.0000000000001417
331	Friedman et al 2018	Friedman, M. R., Sang, J. M., Bukowski, L. A., Matthews, D. D., Eaton, L. A., Raymond, H. F., & Stall, R. (2018). HIV Care Continuum Disparities Among Black Bisexual Men and the Mediating Effect of Psychosocial Comorbidities. Journal of acquired immune deficiency syndromes (1999), 77(5), 451–458. https://doi.org/10.1097/QAI.0000000000001631
332	Pathela et al 2018	Pathela, P., Jamison, K., Braunstein, S. L., Schillinger, J. A., Tymejczyk, O., & Nash, D. (2018). Gaps Along the HIV Care Continuum: Findings Among a Population Seeking Sexual Health Care Services in New York City. Journal of acquired immune deficiency syndromes (1999), 78(3), 314–321. https://doi.org/10.1097/QAI.0000000000001674

For peer review only - http://bmjopen.bmj.com/site/about/guidelines.xhtml

Paper Number	Authors	Reference
333	Ramadhani et al 2018	Ramadhani, H. O., Ndembi, N., Nowak, R. G., Ononaku, U., Gwamna, J., Orazulike, I., Adebajo, S., Crowell, T. A., Liu, H., Baral, S. D., Ake, J., Charurat, M. E., & TRUST/RV368 Study Group (2018). Individual and Network Factors Associated With HIV Care Continuum Outcomes Among Nigerian MSM Accessing Health Care Services. Journal of acquired immune deficiency syndromes (1999), 79(1), e7–e16. https://doi.org/10.1097/QAI.0000000000001756
334	Bukowski et al 2018	Bukowski, L. A., Chandler, C. J., Creasy, S. L., Matthews, D. D., Friedman, M. R., & Stall, R. D. (2018). Characterizing the HIV Care Continuum and Identifying Barriers and Facilitators to HIV Diagnosis and Viral Suppression Among Black Transgender Women in the United States. Journal of acquired immune deficiency syndromes (1999), 79(4), 413–420. https://doi.org/10.1097/QAI.0000000000001831
335	Hoover et al 2019	Hoover, K. W., Hu, X., Porter, S. E., Buchacz, K., Bond, M. D., Siddiqi, A. E., & Haynes, S. G. (2019). HIV Diagnoses and the HIV Care Continuum Among Women and Girls Aged ≥13 Years-39 States and the District of Columbia, 2015-2016. Journal of acquired immune deficiency syndromes (1999), 81(3), 251–256. https://doi.org/10.1097/QAI.0000000000002023
336	Kerrigan et al 2019	Kerrigan, D., Mbwambo, J., Likindikoki, S., Davis, W., Mantsios, A., Beckham, S. W., Leddy, A., Shembilu, C., Mwampashi, A., Aboud, S., & Galai, N. (2019). Project Shikamana: Community Empowerment-Based Combination HIV Prevention Significantly Impacts HIV Incidence and Care Continuum Outcomes Among Female Sex Workers in Iringa, Tanzania. Journal of acquired immune deficiency syndromes (1999), 82(2), 141–148. https://doi.org/10.1097/QAI.0000000000002123
337	Hakim et al 2021	Hakim, A. J., Tippett Barr, B. A., Kinchen, S., Musuka, G., Manjengwa, J., Munyati, S., Gwanzura, L., Mugurungi, O., Ncube, G., Saito, S., Parekh, B. S., Patel, H., Duong, Y. T., Gonese, E., Sleeman, K., Ruangtragool, L., Justman, J., Herman-Roloff, A., & Radin, E. (2021). Progress Toward the 90-90-90 HIV Targets in Zimbabwe and Identifying Those Left Behind. Journal of acquired immune deficiency syndromes (1999), 88(3), 272–281. https://doi.org/10.1097/QAI.0000000000002772
338	Dailey et al 2021	Dailey, A., Johnson, A. S., Hu, X., Gant, Z., Lyons, S. J., & Adih, W. (2021). Trends in HIV Care Outcomes Among Adults and Adolescents-33 Jurisdictions, United States, 2014-2018. Journal of acquired immune deficiency syndromes (1999), 88(4), 333–339. https://doi.org/10.1097/QAI.0000000000002778

For peer review only - http://bmjopen.bmj.com/site/about/guidelines.xhtml

Paper Number	Authors	Reference
339	Hakim et al 2019	Hakim, A. J., Badman, S. G., Weikum, D., Amos, A., Willie, B., Narokobi, R., Gabuzzi, J., Pekon, S., Kupul, M., Hou, P., Aeno, H., Neo Boli, R., Nembari, J., Ase, S., Kaldor, J. M., Vallely, A. J., Kelly-Hanku, A., & Kauntim mi tu Study Team (2019). Considerable distance to reach 90-90-90 targets among female sex workers, men who have sex with men and transgender women in Port Moresby, Papua New Guinea: findings from a cross-sectional respondent-driven sampling survey. Sexually transmitted infections, 96(2), 143–150. https://doi.org/10.1136/sextrans-2019-053961
340	Eamsakulrat et al 2022	Eamsakulrat, P., & Kiertiburanakul, S. (2022). The Impact of Timing of Antiretroviral Therapy Initiation on Retention in Care, Viral Load Suppression and Mortality in People Living with HIV: A Study in a University Hospital in Thailand. Journal of the International Association of Providers of AIDS Care, 21, 23259582221082607. https://doi.org/10.1177/23259582221082607
341	Cope et al 2015	Cope, A. B., Powers, K. A., Kuruc, J. D., Leone, P. A., Anderson, J. A., Ping, L. H., Kincer, L. P., Swanstrom, R., Mobley, V. L., Foust, E., Gay, C. L., Eron, J. J., Cohen, M. S., & Miller, W. C. (2015). Ongoing HIV Transmission and the HIV Care Continuum in North Carolina. PloS one, 10(6), e0127950. https://doi.org/10.1371/journal.pone.0127950
342	Lancaster et al 2016	Lancaster, K. E., Powers, K. A., Lungu, T., Mmodzi, P., Hosseinipour, M. C., Chadwick, K., Go, V. F., Pence, B. W., Hoffman, I. F., & Miller, W. C. (2016). The HIV Care Continuum among Female Sex Workers: A Key Population in Lilongwe, Malawi. PloS one, 11(1), e0147662. https://doi.org/10.1371/journal.pone.0147662
343	Poon et al 2018	Poon, C. M., Wong, N. S., Kwan, T. H., Wong, H. T. H., Chan, K. C. W., & Lee, S. S. (2018). Changes of sexual risk behaviors and sexual connections among HIV-positive men who have sex with men along their HIV care continuum. PloS one, 13(12), e0209008. https://doi.org/10.1371/journal.pone.0209008
344	Sandfort et al 2019	Sandfort, T. G. M., Dominguez, K., Kayange, N., Ogendo, A., Panchia, R., Chen, Y. Q., Chege, W., Cummings, V., Guo, X., Hamilton, E. L., Stirratt, M., & Eshleman, S. H. (2019). HIV testing and the HIV care continuum among sub-Saharan African men who have sex with men and transgender women screened for participation in HPTN 075. PloS one, 14(5), e0217501. https://doi.org/10.1371/journal.pone.0217501
345	Uuskula et al 2020	Uusküla, A., Vickerman, P., Raag, M., Walker, J., Paraskevis, D., Eritsyan, K., Sypsa, V., Lioznov, D., Avi, R., & Des Jarlais, D. (2020). Presenting a conceptual framework for an HIV prevention and care continuum and assessing the feasibility of empirical measurement in

For peer review only - http://bmjopen.bmj.com/site/about/guidelines.xhtml

Paper Number	Authors	Reference
		Estonia: A case study. PloS one, 15(10), e0240224. https://doi.org/10.1371/journal.pone.0240224
347	Karch et al 2016	Karch, D. L., Gray, K. M., Shi, J., & Hall, H. I. (2016). HIV Infection Care and Viral Suppression Among People Who Inject Drugs, 28 U.S. Jurisdictions, 2012-2013. The open AIDS journal, 10, 127–135. https://doi.org/10.2174/1874613601610010127
350	Robeiro et al 2016	Rebeiro, P. F., Cesar, C., Shepherd, B. E., De Boni, R. B., Cortés, C. P., Rodriguez, F., Belaunzarán-Zamudio, P., Pape, J. W., Padgett, D., Hoces, D., McGowan, C. C., & Cahn, P. (2016). Assessing the HIV Care Continuum in Latin America: progress in clinical retention, cART use and viral suppression. Journal of the International AIDS Society, 19(1), 20636. https://doi.org/10.7448/IAS.19.1.20636
351	Hussen et al 2017	Hussen, S. A., Chakraborty, R., Knezevic, A., Camacho-Gonzalez, A., Huang, E., Stephenson, R., & Del Rio, C. (2017). Transitioning young adults from paediatric to adult care and the HIV care continuum in Atlanta, Georgia, USA: a retrospective cohort study. Journal of the International AIDS Society, 20(1), 21848. https://doi.org/10.7448/IAS.20.1.21848
352	Tapera et al 2019	Tapera, T., Willis, N., Madzeke, K., Napei, T., Mawodzeke, M., Chamoko, S., Mutsinze, A., Zvirawa, T., Dupwa, B., Mangombe, A., Chimwaza, A., Makoni, T. M., Mandewo, W., Senkoro, M., Owiti, P., Tripathy, J. P., & Kumar, A. M. V. (2019). Effects of a Peer-Led Intervention on HIV Care Continuum Outcomes Among Contacts of Children, Adolescents, and Young Adults Living With HIV in Zimbabwe. Global health, science and practice, 7(4), 575–584. https://doi.org/10.9745/GHSP-D-19-00210
126	Mody et al 2020	Mody, A., Glidden, D. V., Eshun-Wilson, I., Sikombe, K., Simbeza, S., Mukamba, N., Somwe, P., Beres, L. K., Pry, J., Bolton-Moore, C., Padian, N., Holmes, C. B., Sikazwe, I., & Geng, E. H. (2020). Longitudinal Care Cascade Outcomes Among People Eligible for Antiretroviral Therapy Who Are Newly Linking to Care in Zambia: A Multistate Analysis. Clinical infectious diseases : an official publication of the Infectious Diseases Society of America, 71(10), e561–e570. https://doi.org/10.1093/cid/ciaa268
127	Wang et al 2019	Wang, L., Krebs, E., Min, J. E., Mathews, W. C., Nijhawan, A., Somboonwit, C., Aberg, J. A., Moore, R. D., Gebo, K. A., Nosyk, B., & HIV Research Network (2019). Combined estimation of disease progression and retention on antiretroviral therapy among treated individuals with HIV in the USA: a modelling study. The

For peer review only - http://bmjopen.bmj.com/site/about/guidelines.xhtml

Paper Number	Authors	Reference
		lancet. HIV, 6(8), e531–e539. https://doi.org/10.1016/S2352-3018(19)30148-1
129	Rahmalia et al 2019	Rahmalia, A., Price, M. H., Hartantri, Y., Alisjahbana, B., Wisaksana, R., van Crevel, R., & van der Ven, A. J. A. M. (2019). Are there differences in HIV retention in care between female and male patients in Indonesia? A multi-state analysis of a retrospective cohort study. PloS one, 14(6), e0218781. https://doi.org/10.1371/journal.pone.0218781
130	Gillis et al 2016	Gillis, J., Loutfy, M., Bayoumi, A. M., Antoniou, T., Burchell, A. N., Walmsley, S., Cooper, C., Klein, M. B., Machouf, N., Montaner, J. S., Rourke, S. B., Tsoukas, C., Hogg, R., Raboud, J., & CANOC Collaboration (2016). A Multi-State Model Examining Patterns of Transitioning Among States of Engagement in Care in HIV-Positive Individuals Initiating Combination Antiretroviral Therapy. Journal of acquired immune deficiency syndromes (1999), 73(5), 531–539. https://doi.org/10.1097/QAI.0000000000001109
131	Lee et al 2017	Lee, H., Hogan, J. W., Genberg, B. L., Wu, X. K., Musick, B. S., Mwangi, A., & Braitstein, P. (2017). A state transition framework for patient-level modeling of engagement and retention in HIV care using longitudinal cohort data. Statistics in medicine, 37(2), 302–319. https://doi.org/10.1002/sim.7502
132	Lee et al 2018	Lee, H., Wu, X. K., Genberg, B. L., Mugavero, M. J., Cole, S. R., Lau, B., Hogan, J. W., & Centers for AIDS Research Network of Integrated Clinical Systems (CNICS) Investigators (2018). Beyond binary retention in HIV care: predictors of the dynamic processes of patient engagement, disengagement, and re-entry into care in a US clinical cohort. AIDS (London, England), 32(15), 2217–2225. https://doi.org/10.1097/QAD.0000000000001936
134	Nsanzimana et al 2015	Nsanzimana, S., Kanters, S., Remera, E., Forrest, J. I., Binagwaho, A., Condo, J., & Mills, E. J. (2015). HIV care continuum in Rwanda: a cross-sectional analysis of the national programme. The lancet. HIV, 2(5), e208–e215. https://doi.org/10.1016/S2352-3018(15)00024-7
135	Haber et al 2017	Haber, N., Tanser, F., Bor, J., Naidu, K., Mutevedzi, T., Herbst, K., Porter, K., Pillay, D., & Bärnighausen, T. (2017). From HIV infection to therapeutic response: a population-based longitudinal HIV cascade-of-care study in KwaZulu-Natal, South Africa. The lancet. HIV, 4(5), e223–e230. https://doi.org/10.1016/S2352-3018(16)30224-7

For peer review only - http://bmjopen.bmj.com/site/about/guidelines.xhtml

Table S4: Cascades stages used in HIV cascade and continuum of care assessments in articles included in this review

**Cross-sectional**	**Longitudinal**
ALL (HIV negative)	PLHIV
PLHIV (incidence and prevalence)	Diagnosis
Diagnosis	Linkage to care or in HIV care
Linkage to care	Optimal care
ART eligibility	On ART (in care or transferred out or LTFU or re-engaged or death)
Clinical staging	No ART or off-ART (in care or transferred out or LTFU or death)
On ART (history of ART or ever on ART and current use of ART)	ART eligibility
ART adherence	Retention in care
Retention in care	LTFU or disengaged
Viral load suppression	Sub-optimal care
LTFU	Engagement in care
In care	Transferred out
Death	Death
CD4 testing	Viral load suppression or high CD4

For peer review only - http://bmjopen.bmj.com/site/about/guidelines.xhtml

Table S5: The reported study design and data analysis methods used to assess the HIV cascade and continuum of care among longitudinal studies (n=21)

Reported study design	Data analysis methods used	Number of articles
Longitudinal	Survival analysis: Competing risk methods	5
Longitudinal	Repeated cross-sectional	1
Longitudinal	Survival analysis	2
Longitudinal	Survival analysis and multistate models	6
Longitudinal	Cross-sectional analysis	5
Longitudinal	Repeated cross-sectional and time spent in the cascade stage	1
Longitudinal	Repeated measure analysis	1

For peer review only

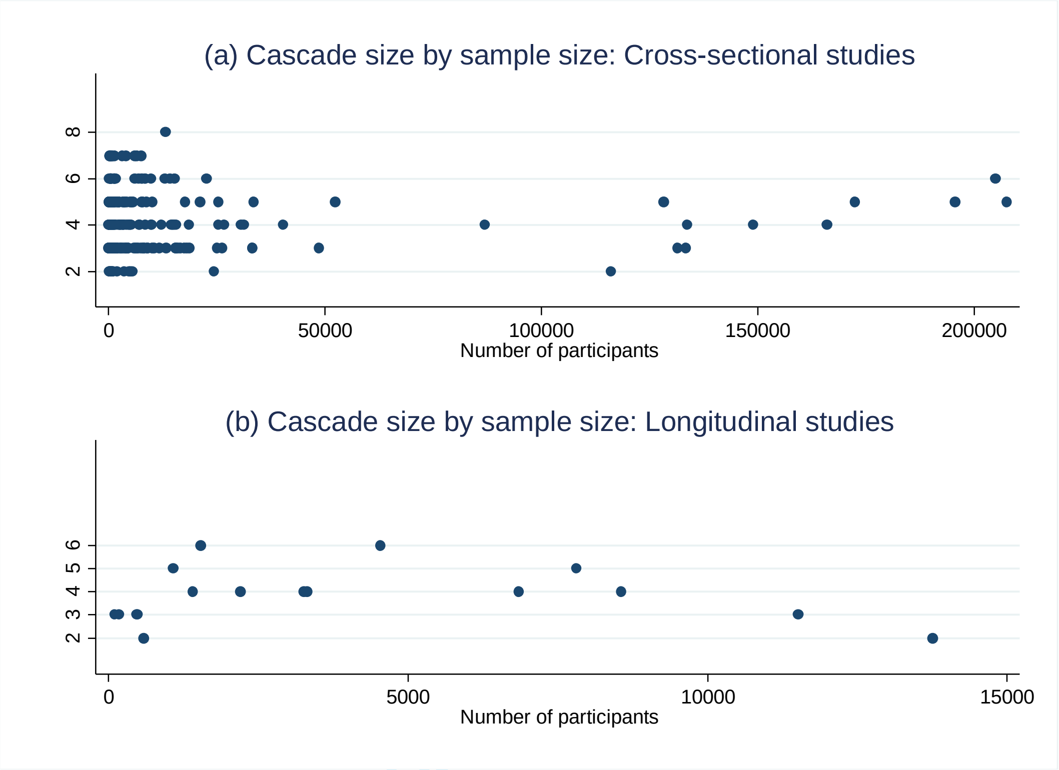

Figure S1: The distribution of cascade size by study number of participants included, in panel (a) sample size was truncated at 300,000 (excluded 12 studies), and in panel (b) sample size was truncated at 20,000 (excluded 4 studies) to allow better visualization of the distribution

For peer review only - http://bmjopen.bmj.com/site/about/guidelines.xhtml

(a) Mody et al 2020

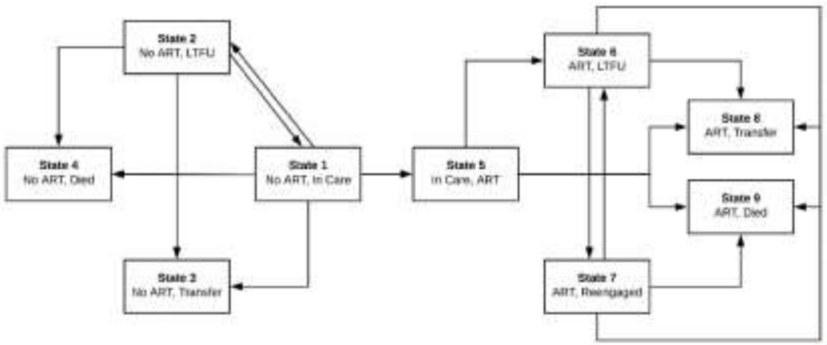

(b) Rahmalia et al 2019

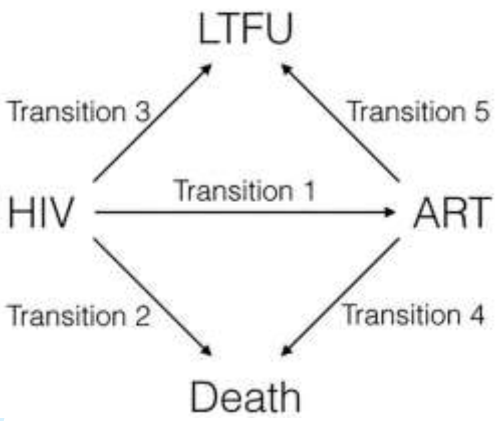

(c) Lee et al 2017

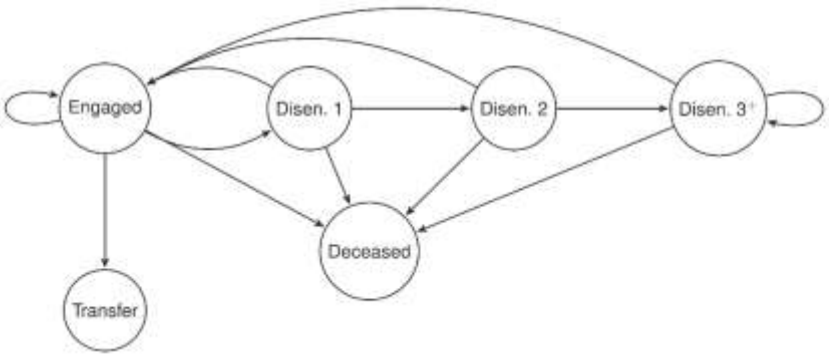

(d) Lee et al 2018

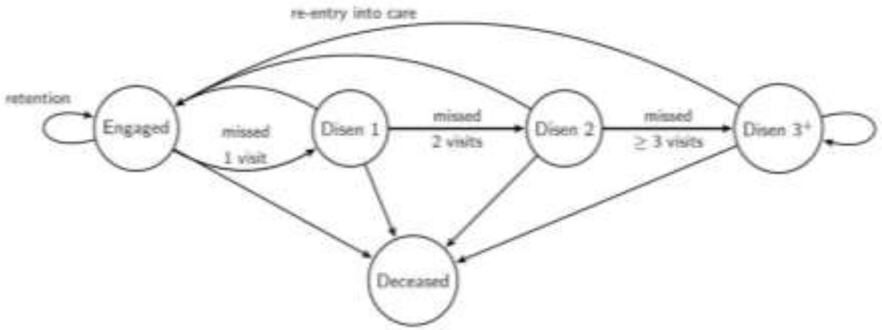

(E) Wang et al 2019

For peer review only - http://bmjopen.bmj.com/site/about/guidelines.xhtml

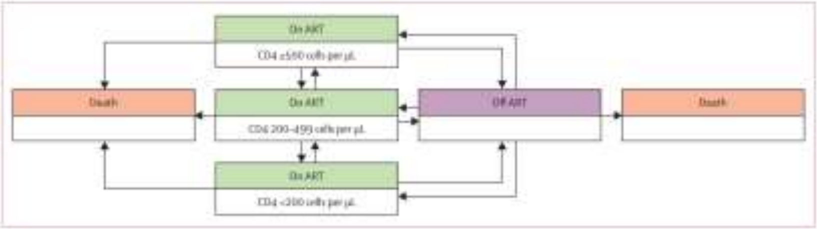

Figure S2: Multistate frameworks of included articles

For peer review only

For peer review only - http://bmjopen.bmj.com/site/about/guidelines.xhtml

ResearchGate

See discussions, stats, and author profiles for this publication at: https://www.researchgate.net/publication/319713049

2017 Guidance for the Conduct of JBI Scoping Reviews

Chapter · September 2017

CITATIONS
29

READS
34,092

6 authors, including:

Micah Peters
University of South Australia
107 PUBLICATIONS **9,825** CITATIONS

Christina M Godfrey
Queen's University
67 PUBLICATIONS **8,557** CITATIONS

Hanan Khalil
La Trobe University
97 PUBLICATIONS **4,104** CITATIONS

Patricia Mcinerney
University of the Witwatersrand
66 PUBLICATIONS **3,526** CITATIONS

SEE PROFILE

SEE PROFILE

SEE PROFILE

SEE PROFILE

Some of the authors of this publication are also working on these related projects:

Project Guidance for the Conduct and Reporting of Scoping Reviews View project

Project Education in Collective Health View project

All content following this page was uploaded by Micah Peters on 21 September 2017.

The user has requested enhancement of the downloaded file.

Chapter 11: Scoping Reviews

For peer review only

Preamble:

The Joanna Briggs Institute Reviewer's Manual^ provides guidance to authors for the conduct and preparation of JBI systematic reviews and evidence syntheses. The JBI Reviewer's Manual has separate chapters devoted synthesis of different types of evidence and to address different types of review questions.

This document is contains Chapter 11: Scoping Reviews from the 4th Edition of the JBI Reviewer's Manual, and is up to date as of September 21, 2017. This Chapter includes changes that correspond to the latest methodological developments determined by the JBI Methodology Groups and JBI Scientific Committee, the latest developments with the JBI SUMARI software (https://www.jbisumari.org/) and also feedback by users.

The online chapter can be accessed at:

https://reviewersmanual.joannabriggs.org/display/MANUAL/Chapter+11%3A+Scoping+reviews

The open-access published journal article can be cited as:

Peters MDJ, Godfrey CM, McInerney, Khalil H, Parker D, and Baldini Soares C. Guidance for conducting systematic scoping reviews. Int J Evid Based Healthc. 2015. 13(3):141-146. Available: http://www.ncbi.nlm.nih.gov/pubmed/26134548

^Aromataris E, Munn Z (Editors). *Joanna Briggs Institute Reviewer's Manual.* The Joanna Briggs Institute, 2017. Available from https://reviewersmanual.joannabriggs.org/

For peer review only - http://bmjopen.bmj.com/site/about/guidelines.xhtml

For peer review only

For peer review only - http://bmjopen.bmj.com/site/about/guidelines.xhtml

Contents

0: Contributors and how to cite ... 5
1: Scoping reviews and evidence-based practice ... 6
1.1 Why a scoping review? ... 6
1.2 JBI scoping reviews ... 7
1.3 The scoping review framework ... 9
2: Development of a JBI scoping review protocol ... 10
2.1 Author information ... 10
2.2 Developing the title, objective, and question ... 10
2.3 Background ... 11
2.4 Inclusion criteria ... 12
2.5 Search strategy ... 13
2.6 Extraction of the results ... 14
2.7 Presentation of the results ... 15
3: The scoping review and summary of the evidence ... 16
3.1 Title of the scoping review ... 16
3.2 Review authors ... 16
3.3 Executive summary ... 16
3.4 Main body of the report ... 18
3.5 Method of the report ... 19
3.6 Presenting the results ... 20
3.7 Discussion, Conclusion, and Implications for research and practice ... 22
3.8 End matter ... 22
3.9 References ... 22
4: Appendices ... 23
4.1 Appendix I: Template study details and characteristics and results extraction instrument ... 23
References ... 24

For peer review only

For peer review only - http://bmjopen.bmj.com/site/about/guidelines.xhtml

Authors:

Micah D J Peters
The Joanna Briggs Institute, Faculty of Health Sciences, The University of Adelaide, Australia

Christina M Godfrey
Queen's Joanna Briggs Collaboration, Queen's University, Kingston, Canada

Patricia McInerney
The Wits - JBI Centre for Evidence-based practice, Faculty of Health Sciences, University of the Witwatersrand, South Africa

Cassia Baldini Soares
The Brazilian Centre for Evidence-based Healthcare, School of Nursing, University of São Paulo, Brazil

Hanan Khalil
Monash University, School of Rural Health, The Centre for Chronic Diseases Management, Australia

Deborah Parker
The University of Queensland, Australian Centre for Evidence Based Community Care

How to Cite the Online Manual:

Peters MDJ, Godfrey C, McInerney P, Baldini Soares C, Khalil H, Parker D. Chapter 11: Scoping Reviews. In: Aromataris E, Munn Z (Editors). Joanna Briggs Institute Reviewer's Manual. The Joanna Briggs Institute, 2017. Available from https://reviewersmanual.joannabriggs.org/

For peer review only - http://bmjopen.bmj.com/site/about/guidelines.xhtml

1: Scoping reviews and evidence-based practice

Evidence-based practice is an expanding field and together with a rapid increase in the availability of primary research, the conduct of reviews has also escalated. Different forms of evidence and different kinds of review objectives and questions call for the development of new approaches that are designed to more effectively and rigorously synthesize the evidence. In 2009 Grant and Booth identified 14 different types of reviews (Grant and Booth, 2009). Scoping reviews, also called "mapping reviews" or "scoping studies" are one of these (Ehrich et al. 2002; Anderson et al. 2008). In 2005 Arksey and O'Malley proposed a framework for conducting them (Arksey and O'Malley, 2005). The Arksey and O'Malley framework was advanced and extended in 2010 by Levac, Colquhoun and O'Brien (2010) and then further refined and corresponding guidance developed by the present authors (Peters et al. 2015).

1.1 Why a scoping review?

There are a number of reasons why a scoping review might be conducted. Unlike other reviews that address relatively precise questions, such as a systematic review of the effectiveness of a particular intervention based on a precise set of outcomes, scoping reviews can be used to map the key concepts underpinning a research area as well as to clarify working definitions, and/or the conceptual boundaries of a topic (Arksey and O'Malley, 2005). A scoping review may focus on one of these aims or all of them as a set.

Scoping reviews can usefully map evidence in a number of ways (Anderson et al. 2008). Scoping reviews undertaken with the objective of providing a map of the range of the available evidence can be undertaken as a preliminary exercise prior to the conduct of a systematic review. Scoping reviews are useful for examining emerging evidence when it is still unclear what other, more specific questions can be posed and valuably addressed. For example, while there are few studies on the sustainability of knowledge translation interventions in the area of chronic disease management, a scoping review has provided the foundation for a future systematic review to investigate the impact of sustainable knowledge translation interventions on health outcomes (Tricco et al. 2016a).

Beyond underpinning future systematic review, scoping reviews can also inform clinical decision making and practice. For example, a scoping review might seek to map the types and details of tools used to assess quality of life following tonsillectomy (Kao et al. 2017a).. In this case, the scoping review could potentially be used both to provide direction for future systematic reviews as well as having value to knowledge users by providing a comprehensive evidence base to aid in the selection of quality of life tools for use in clinical practice.

Scoping reviews can be conducted to examine and clarify broad areas to identify gaps in the evidence, clarify key concepts, and report on the types of evidence that address and inform practice in a topic area. Scoping reviews can be used to map evidence in relation to time (when it was published), location (country), source (peer reviewed or grey literature), approach (how it was studied/researched), and/or origin (healthcare or academic discipline) (Anderson et al. 2008).

Davis, Drey and Gould (2009) explain how, as useful tools for evidence reconnaissance, scoping reviews can be used to provide a broad overview of a topic. For instance, a scoping review that seeks to develop a "concept map" may aim to explore how, by whom and for what

For peer review only - http://bmjopen.bmj.com/site/about/guidelines.xhtml

purpose a particular term is used in a given field (Anderson et al. 2008). Similarly, scoping reviews can be conducted in order to establish a comprehensive understanding of how scoping reviews themselves have been conducted and reported (Pham et al, 2014; Tricco et al. 2016b). Scoping review methodology was used to identify papers and guidelines that had either utilized or described scoping review methods and/or assessed the quality of reporting for scoping reviews (Tricco et al. 2016b). This review showed how the number of scoping reviews had steadily increased since 2012, that there was variation in terms of how they were conducted and reported, and that standardized reporting guidelines were absent.

Scoping reviews may also be used to develop "policy maps" by identifying and mapping evidence from policy documents and reports that guide practice in a particular field (Anderson et al. 2008). For example, a scoping review might include the objective of mapping research papers and policy documents that concern models of transition for young people to adult health services to provide evidence for best practice transitional care for children with complex health needs (Watson et al. 2011).

For peer review only

For peer review only - http://bmjopen.bmj.com/site/about/guidelines.xhtml

For peer review only

The value of scoping reviews to evidence-based practice is the examination of a broader area to identify gaps in the research knowledge base (Crilly, Jashapara and Ferlie, 2010), clarify key concepts (de Chavez et al. 2005), and report on the types of evidence that address and inform practice in the field (Decaria, Sharp and Petrella, 2012).

1.2 JBI scoping reviews

The synthesis of evidence in the form of the systematic review is at the center of evidence-based practice (Pearson et al. 2005). Systematic reviews traditionally bring together evidence from quantitative literature to answer questions on the effectiveness of a specific intervention for a particular condition. Beyond effectiveness, the Joanna Briggs Institute (JBI) is also interested in the context of care delivery, its cost-effectiveness, as well as patient, carer and healthcare provider preferences. These foci are explored in terms of the appropriateness, meaningfulness, and feasibility of healthcare practices and delivery. These sorts of questions are most commonly answered by consideration of other forms of primary evidence found in qualitative and economic research. The results of well-designed research studies of any methodology are regarded by the JBI as potential sources of credible evidence. To match this broader and more inclusive view of evidence, the Institute has developed a number of methodologies and methods for the synthesis of evidence to support healthcare decision-making.

All JBI systematic reviews – including scoping reviews – begin with the development of an *a-priori* protocol with inclusion and exclusion criteria that relate clearly to the objective/s and review question/s. A typical systematic review aims to answer a specific question (or series of questions) based on very precise inclusion criteria, for example, a systematic review may pose the following precise question based upon the PICO (Population, Intervention, Comparator, and Outcome) elements of its inclusion criteria:

> "What is the effectiveness of the Gardasil vaccine compared with the Cervarix vaccine in preventing Human Papillomavirus infection in adolescent and young adult women?"

It is clear from this question that only certain types of quantitative evidence and data would be relevant and that the review will be very specific in terms of the population, intervention, comparator, and kinds of outcomes against which it will measure effectiveness.

A scoping review will have a broader "scope" with correspondingly less restrictive inclusion criteria. The following question based upon the PCC (Population, Concept and Context) elements of the inclusion criteria may be posed:

> "What types of neurological reactions to the Human Papillomavirus vaccination have been reported?"

This question leaves the population rather "open" and implies that both men and women of any age will be suitable for inclusion as long as they have received a HPV vaccination. The intervention in this example is also 'open' to any kind of HPV vaccine and does not stipulate that there will be any kind of measurement of outcomes or comparison involved. The "concept" of this scoping review (neurological reactions) is also broad, and could cover any kind of neurological outcome as long as it is a reaction to HPV vaccination. For this particular question, the 'context' has also been left open, so the evidence may come from any context (e.g. geographical, healthcare setting, sociocultural).

For peer review only - http://bmjopen.bmj.com/site/about/guidelines.xhtml

For peer review only

For peer review only - http://bmjopen.bmj.com/site/about/guidelines.xhtml

An especially important point is that the scoping review question may draw upon data from any type of evidence and research methodology, and is not restricted to quantitative studies (or any other study design) alone. This however is not prescriptive; reviewers may decide that particular study designs would be beyond the scope of their scoping review, or not be appropriate or useful for consideration. For example, in the protocol, this scoping review example may specify that text and opinion literature will not be included. Because of the broad nature of scoping reviews, they are particularly useful for bringing together evidence from disparate or heterogeneous sources. In the example scoping review question regarding HPV vaccination above, reports of neurological side effects such as syncope (fainting) from randomized controlled trials can be considered side by side with qualitative accounts of patients' experiences of paralysis following HPV vaccination.

It is important to highlight the distinction between scoping reviews and "mixed methods" systematic reviews that also rely on evidence from a number of different study designs (Pearson et al. 2015). While in a scoping review the goal is to determine what kind of evidence (quantitative and/or qualitative) is available on the topic and to represent this evidence by mapping or charting the data, mixed methods systematic reviews are designed to answer a question or questions based on the synthesis of evidence from for example qualitative, quantitative and economic research (Reilly et al. 2016). For example:

> "What is the effectiveness, cost effectiveness, acceptability and implementation barriers/enablers for chronic kidney disease management programs for Aboriginal and Torres Strait Islander Australians?"

The goal of this mixed methods systematic review was to: i) report on a the effectiveness of chronic kidney disease management programs from quantitative evidence of effectiveness, ii) report on the relative cost-effectives of chronic kidney disease management programs from economic evidence, and to iii) examine the experiences of Aboriginal and Torres Strait Islander Australians and healthcare providers regarding chronic kidney disease management programs in terms of acceptability as well as barriers and enablers of implementation.. Following separate methodological quality assessment, data extraction and synthesis, the results of each synthesis were then brought together in a comprehensive synthesis using evidence from each research type to answer a specific question. In this example, the knowledge gained from the qualitative and economic evidence can be used to enhance the knowledge gained from the quantitative evidence.

Another important distinction between scoping reviews and systematic reviews is that, unlike systematic reviews, scoping reviews provide an overview of the existing evidence, regardless of quality. This is because scoping reviews aim to provide a map of what evidence has been produced as opposed to seeking only the best available evidence to answer more specific questions related to policy and practice. Hence, unless otherwise specified, a formal assessment of methodological quality of the included studies of a scoping review is generally not performed or congruent with the purpose of scoping reviews.

While implications for research, including for primary research, other scoping reviews, or systematic reviews, may be generated from the results of a scoping review – especially those conducted with the objective of being precursors to systematic reviews, implications for practice are limited by the fact that a formal assessment of methodological quality of the included studies of a scoping review is generally not performed. If implications for practice are

For peer review only - http://bmjopen.bmj.com/site/about/guidelines.xhtml

developed, it is expected that they will clearly flow from the objectives of the scoping review.

For peer review only

For peer review only - http://bmjopen.bmj.com/site/about/guidelines.xhtml

1.3 The scoping review framework

The framework proposed by Arksey and O'Malley (2005) has been influential in the conduct of scoping reviews for some time. Their framework has been further enhanced by the work of Levac, Colquhoun and O'Brien (2010) (see Table 11.1). Levac and colleagues (2010) provide more explicit detail regarding what occurs at each stage of the review process and this enhancement increases both the clarity and rigor of the review process. Both of these frameworks have been drawn on in the development of the JBI approach to the conduct of scoping reviews (Peters et al. 2015).

Table 11.1: Scoping review frameworks

	Arksey and O'Malley framework (2005, p. 22-23)	Enhancements proposed by Levac, Colquhoun and O'Brien. (2010, p. 4-8)	Enhancements proposed by Peters et al (2015).
1.	Identifying the research question	Clarifying and linking the purpose and research question	Defining and aligning the objective/s and question/s
2.	Identifying relevant studies	Balancing feasibility with breadth and comprehensiveness of the scoping process	Developing and aligning the inclusion criteria with the objective/s and question/s
3.	Study selection	Using an iterative team approach to selecting studies and extracting data	Describing the planned approach to evidence searching, selection,
4.	Charting the data	Incorporating a numerical summary and qualitative thematic analysis	Searching for the evidence
5.	Collating, summarizing and reporting the results	Identifying the implications of the study findings for policy, practice or research	Selecting the evidence
6.	Consultation (optional)	Adopting consultation as a required component of scoping study methodology	Extracting the evidence
7.			Charting the evidence
8.			Summarizing the evidence in relation to the objective/s and question/s
9.			Consultation of information scientists, librarians, and/or experts (throughout)

For peer review only - http://bmjopen.bmj.com/site/about/guidelines.xhtml

2: Development of a JBI scoping review protocol

As with all systematic reviews using JBI methodologies, an *a-priori* protocol must be developed before undertaking the scoping review. A scoping review protocol is important as it pre-defines the objectives and methods of the scoping review. It is a systematic approach to the conduct and reporting of the review and allows transparency of process. This in turn allows readers to see how the results of the scoping review were arrived at. The protocol should detail the criteria that the reviewers intend on using to include and exclude studies and to identify what data is relevant, and how the data will be extracted and mapped. The protocol provides the plan for the scoping review and is important in limiting the occurrence of reporting bias. Any deviations of the scoping review report from the protocol should be clearly addressed and explained in the scoping review report. It is also recommended that all scoping reviews should contain the following sentence:

> "The objectives, inclusion criteria and methods for this scoping review were specified in advance and documented in a protocol." (citation)

The citation should be to the corresponding protocol which may be published in the JBI Database of Systematic Reviews and Implementation Reports. Reviewers should also be aware that PROSPERO (the international prospective register of systematic reviews administered by the University of York's Centre for Reviews and Dissemination) states that scoping reviews (and literature reviews) are currently ineligible for registration in the database (Centre for Reviews and Dissemination, n.d. 'inclusion criteria', para. 5) despite appearing to contain a small number of recently registered scoping reviews. In accordance with the recommendations for reporting of systematic reviews detailed in the Preferred Reporting Items for Systematic Reviews and Meta-Analyses (PRISMA) guidelines, this sentence should appear as the final line of the background/introduction section of the review report. Another point prospective scoping reviewers should be aware of is that an extension of the PRISMA statement called the PRISMA-ScR is currently under development and registered with the Enhancing the Quality and Transparency Of health Research (EQUATOR) Network (Tricco, Strauss and Moher, 2015). Led by Tricco, Strauss and Moher, this reporting checklist is being developed with the involvement of numerous international experts on scoping review methodology and evidence synthesis including authors of the JBI methodological guidance described in this chapter. The JBI approach to conducting and reporting scoping reviews described here and in Peters et al. (2015) will be congruent with the PRISMA-ScR checklist which will assist in standardizing the conduct and reporting of scoping reviews. Reviewers are therefore encouraged to use and cite the JBI methodology (Peters et al. 2015) and also indicate their compliance with the PRISMA-ScR checklist when it becomes available.

2.1 Author information

All reviews using JBI methodologies require at least two reviewers in order to minimize reporting bias. The names of all reviewers, institutional affiliations, and JBI center affiliations (if relevant), and email address for the corresponding author must be included.

2.2 Developing the title, objective, and question

Title of the scoping review protocol

The title of the protocol (and the subsequent review) should be informative and give a clear indication of the topic of the scoping review. It is recommended that the title should always include the

For peer review only - http://bmjopen.bmj.com/site/about/guidelines.xhtml

phrase "…: a scoping review" to allow easy identification of the type of document it represents. Correspondingly, protocols should also be identified as such. This is a simple example of a scoping review protocol title by Mordiffi, Peters and Ang, 2016):

"Non-invasive thermometers used in healthcare facilities: A scoping review protocol"

Scoping review titles should not be phrased as questions. For example:

"What types non-invasive thermometers have been used in healthcare facilities?"

The JBI uses a range of mnemonics for different types of review (and research) questions. It is suggested that the "PCC" mnemonic be used as a guide to construct a clear and meaningful title for a scoping review. The PCC mnemonic stands for the Population, Concept, and Context. There is no need for explicit outcomes, interventions or phenomena of interest to be stated for a scoping review; however elements of each of these may be implicit in the Concept under examination.

For peer review only

For peer review only - http://bmjopen.bmj.com/site/about/guidelines.xhtml

For peer review only

The title of the protocol (and subsequent review) should be structured to reflect the core elements of the PCC. Using the PCC mnemonic helps to construct a title that provides potential readers with important information about the focus and scope of the review, and its applicability to their needs. For example, if the review aims to map a range of devices as part of the concept (such as non-invasive thermometers) this should be stated in the title. Including the context in the title helps readers to position the review when they are searching for evidence related to their own particular information needs.

As discussed in further depth below, there should be congruence between the title, review objective/s, question/s and inclusion criteria.

Scoping review objectives

The objective of a scoping review must be clearly stated and be congruent with the title. The objective of the scoping review should indicate what the scoping review project is trying to achieve. The objective may be broad and will guide the scope of the enquiry. For the title example above, the objective has been phrased:

> "The objective of the review is to map the available evidence to provide an overview of the use of non-invasive thermometers in the general context of health care." (Mordiffi, Peters and Ang, 2016, p. 106)

The objective should also clearly underpin the question posed by the scoping review and direct the development of the specific inclusion criteria based on clearly identifiable PCC. Further considerations around the importance of clearly identifying the objective/s and review question/s for scoping reviews has been discussed elsewhere (Peters, 2016).

Scoping review questions

The scoping review question guides and directs the development of the specific inclusion criteria for the scoping review. Clarity in the review question assists in developing the protocol, facilitates effectiveness in the literature search, and provides a clear structure for the development of the scoping review report. As with the title, the question should incorporate the PCC elements. A scoping review will generally have one primary question, e.g.

> "What quality of life questionnaires are available for pediatric patients following tonsillectomies with or without adenoidectomies for chronic infections or sleep-disordered breathing?" (Kao, Peters and Ooi, 2017a, p. 1).

If that question sufficiently addresses the PCC and adequately corresponds with the objective of the review, sub questions will not be needed. However, some scoping review questions benefit from one or more sub questions that delve into particular attributes of Context, Population or Concept. Sub questions can be useful in outlining how the evidence is likely to be mapped. For example, the primary question relates to the broad population; however, the sub questions delve into potential particular issues relate to males or particular age groups of females as distinct sub populations may be relevant. Likewise, a sub question may help to justify mapping the evidence by context, e.g.

> "What nutritional screening instruments have been validated for use for the adult population in primary healthcare settings?" (Håkonsen et al. 2015, p. 92).

2.3 Background

The background section should be comprehensive and cover all the main elements of the topic

For peer review only - http://bmjopen.bmj.com/site/about/guidelines.xhtml

under review. Due to scoping reviews being essentially exploratory, it is not expected that the background covers the extant knowledge in the area under review. The reason for undertaking the scoping review should be clearly stated together with what the scoping review is intended to inform.

For peer review only

For peer review only - http://bmjopen.bmj.com/site/about/guidelines.xhtml

For publication in the JBI Database of Systematic Reviews and Implementation Reports, the suggested length for the background section of the scoping review protocol is approximately 1000 words. The background should detail any definitions important to the topic of interest. The information in the background section must also be sufficient to put the inclusion criteria in context, including an indication of whether or not there are existing scoping reviews, systematic reviews, research syntheses, and/or primary research papers available on the topic, hence supporting the rationale to conduct the scoping review. While the inclusion criteria section of the protocol (explained below) should contain clear details of each of the PCC elements, the background must provide sufficient detail in terms of the rationale for each element. Explaining for example, why only primary care settings are of interest in terms of the context of the review question above.

The background section should conclude with a statement that a preliminary search for existing scoping reviews (and ideally systematic reviews too) on the topic has been conducted. The date of the search/es and databases searched or search platforms utilized must be stated, e.g. J B I Database of Systematic Reviews and Implementation Reports, Cochrane Database of Systematic Reviews, CINAHL, PubMed, EPPI, and Epistemonikos, where relevant. If existing reviews or systematic reviews are available on the topic, a justification that specifies how the proposed review will differ from those already conducted should be detailed. This is so that readers can easily establish what new knowledge or insight the proposed review will contain in relation to existing evidence syntheses.

For publication in the JBI Database of Systematic Reviews and Implementation Reports, The Vancouver style of referencing should be used throughout the protocol with superscript numbers without brackets, used for in-text citations. A guide to the Vancouver style of referencing can be found at: http://openjournals.net/files/Ref/VANCOUVER%20Reference%20guide.pdf

2.4 Inclusion criteria

The "inclusion criteria" of the protocol details the basis on which sources will be considered for inclusion in the scoping review and should be clearly defined. These criteria provide a guide for the reader to clearly understand what is proposed by the reviewers and, more importantly, a guide for the reviewers themselves on which to base decisions about the sources to be included in the scoping review. As explained above, as for other review types, there must be clear congruence between the tile, objective/s, question/s, and inclusion criteria of a scoping review.

Types of participants

Important characteristics of participants should be detailed, including age and other qualifying criteria that make them appropriate for the objectives of the scoping review and for the review question. In the example question above regarding pediatric tonsillectomy quality of life assessment instruments, these characteristics included pediatric patients 16 years of age or younger undergoing tonsillectomy with or without adenoidectomy for chronic tonsillitis or sleep-disordered breathing.. Justification for the inclusion or exclusion of participants should be explained. Confounding population factors, e.g. co-morbidities or co-existing conditions (e.g. congenital heart defects), can also be detailed here as exclusion criteria.

Concept

For peer review only - http://bmjopen.bmj.com/site/about/guidelines.xhtml

The core concept examined by the scoping review should be clearly articulated to guide the scope and breadth of the inquiry. This may include details that pertain to elements that would be detailed in a standard systematic review, such as the “interventions” and/or “phenomena of interest” and/or “outcomes”. For example, quality of life assessment tools – which could be understood to be interventions or approaches to measuring outcomes – was part of the concept of a scoping review designed to map the types and details of existing tools . It would then be necessary to explain any relevant details pertaining to the concept that may be important for the review, for example, whether only particular tools are to be investigated (e.g. validated tools only) or whether any/all tools are eligible for inclusion.

For peer review only - http://bmjopen.bmj.com/site/about/guidelines.xhtml

Outcomes may also be a component of a scoping review's "Concept". If outcomes of interest are to be explained, they should be linked closely to the objective and purpose for undertaking the scoping review. For example, the exemplar review was also concerned with mapping the outcomes of any psychometric assessments that had been used to measure the validity, reliability, and responsiveness to change of included quality of life tools. Details of this were also a part of the "Concept" in terms of defining psychometric testing as well as the different types of validity outcomes being sought (e.g. construct and criterion).

Context

The "Context" element of a scoping review of will vary depending on the objective/s and question/s of the review. The context should be clearly defined and may include, but is not limited to, consideration of cultural factors such as geographic location and/or specific racial or gender-based interests. In some cases, context may also encompass details about the specific setting (such as acute care, primary health care or the community). Reviewers may choose to limit the context of their review to a particular country or health system or healthcare setting, depending on the topic and objectives.

In the scoping review example above, quality of life assessment tools were broadly sought from within the context of pediatric care both pre- and/or postoperatively.

Types of sources

For the purposes of a scoping review, the "source" of information can include any existing literature, e.g. primary research studies, systematic reviews, meta-analyses, letters, guidelines, websites etc. Reviewers may wish to leave the source of information "open" to allow for the inclusion of any and all sources. Otherwise, the reviewers may wish to impose limits on the types of sources they wish to include. This may be done on the basis of having some knowledge of the types of sources that would be most useful and appropriate for a particular topic. For example, the scoping review example on quality of life assessment tools sought only quantitative studies based upon the knowledge that qualitative papers focusing on peoples' experiences were unlikely to contain relevant details regarding the tools themselves or the results of psychometric testing as as such would not be particularly appropriate or useful for meeting the objectives of the review.

2.5 Search strategy

The search strategy for a scoping review should ideally aim to be as comprehensive as possible within the constraints of time and resources in order to identify both published and unpublished (grey literature) primary studies as well as reviews. Any limitations in terms of the breadth and comprehensiveness of the search strategy should be detailed and justified. As recommended in all JBI types of reviews, a three-step search strategy is to be utilized. Each step must be clearly stated in this section of the protocol. The first step is an initial limited search of at least two appropriate online databases relevant to the topic. The databases MEDLINE (PubMed) and CINAHL would be appropriate for a scoping review on quality of life assessment tools. This initial search is then followed by an analysis of the text words contained in the title and abstract of retrieved papers, and of the index terms used to describe the articles. A second search using all identified keywords and index terms should then be undertaken across all included databases. Thirdly, the reference list of identified reports and articles should be searched for

For peer review only - http://bmjopen.bmj.com/site/about/guidelines.xhtml

additional studies. This third stage may examine the reference lists of all identified studies or examine solely the reference lists of the studies that have been selected from full-text and/or included in the review. In any case, it should be clearly stated which group of studies will be examined. A statement should be included of the reviewers' intent to contact authors of primary studies or reviews for further information, if this is relevant. Finally, a full search strategy for at least one database should be included as an appendix to the protocol.

For peer review only

For peer review only - http://bmjopen.bmj.com/site/about/guidelines.xhtml

Reviewers should include the languages that will be considered for inclusion in the review as well as the timeframe, with an appropriate and clear justification for choices.

As the review question might be broad, authors may find that it is appropriate to search for all sources of evidence (e.g. primary studies and text/opinion articles) simultaneously with the one search strategy. This also depends on the relevance of the evidence sources to the topic under review and its objectives. This approach will lead to a greater sensitivity in the search, which is desirable for scoping reviews.

The search for a scoping review may be quite iterative as reviewers become more familiar with the evidence base, additional keywords and sources, and potentially useful search terms may be discovered and incorporated into the search strategy. The input of a research librarian or information scientist can be invaluable in designing and refining the search.

2.6 Extraction of the results

In scoping reviews, the data extraction process may be referred to as "charting the results". This process provides the reader with a logical and descriptive summary of the results that aligns with the objective/s and question/s of the scoping review.

A draft charting table or form should be developed at the protocol stage to record the key information of the source, such as author, reference, and results or findings relevant to the review question/s. This may be further refined at the review stage and the charting table updated accordingly. Some key information that reviewers might choose to chart are:

a. Author(s)
b. Year of publication
c. Origin/country of origin (where the study was published or conducted)
d. Aims/purpose
e. Study population and sample size (if applicable)
f. Methodology / methods
g. Intervention type, comparator and details of these (e.g. duration of the intervention) (if applicable)
h. Duration of the intervention (if applicable)
i. Outcomes and details of these (e.g. how measures) (if applicable)
j. Key findings that relate to the scoping review question/s.

A template data extraction instrument for study details, characteristics and results extraction is provided in Appendix 1 of this chapter which can be adapted by reviewers to use in their own scoping review protocols and reviews with citation to the JBI methodology guidance for scoping reviews.

For ease of reference and tracking, it is suggested that reviewers keep careful records to identity each source. As reviewers chart each study, it may become apparent that additional unforeseen data can be usefully charted. Charting the results can therefore be an iterative process whereby the charting table is continually updated. It is suggested that the review team become familiar with the source results and trial the extraction form on two or three studies to ensure all relevant results are extracted. This approach is favored by other experts on the conduct of scoping

For peer review only - http://bmjopen.bmj.com/site/about/guidelines.xhtml

reviews (Arksey and O'Malley, 2005; Armstrong et al. 2011; Valaitis et al. 2012).

For peer review only

For peer review only - http://bmjopen.bmj.com/site/about/guidelines.xhtml

2.7 Presentation of the results

At the time of protocol development, the reviewers should provide some plan for the presentation of results – for example, a draft chart or table. This would be expected to be further refined toward the end of the review when the reviewers have the greatest awareness of the contents of their included studies.

The results of a scoping review may be presented as a map of the data extracted from the included papers in a diagrammatic or tabular form, and/or in a descriptive format that aligns with the objective/s and scope of the review. The elements of the PCC inclusion criteria may be useful to guide how the data should be mapped most appropriately. In the scoping review example, because the objective was to map the types and details of quality of life assessment tools used in relation to pediatric tonsillectomy, the data may be usefully mapped by a tabular presentation of how the different tools reported in each included paper contain the same or different measurement domains, number of items, and differences in terms of the results of validity, reliability, and/or responsiveness to change testing.

The tables and charts may also show results as: distribution of studies by year or period of publication (depends on each case), countries of origin, area of intervention (clinical, policy, educational, etc.) and research methods. A narrative summary should accompany the tabulated and/or charted results and should describe how the results relate to the review objective/s and question/s.

The results can also be classified under main conceptual categories, such as: “intervention type”, “study population” (and sample size, if it is the case), “duration of intervention”, “aims”, “methodology adopted”, “key findings” (evidence established), and “gaps in the research”. For each category reported, a clear explanation should be provided.

For peer review only - http://bmjopen.bmj.com/site/about/guidelines.xhtml

3: The scoping review and summary of the evidence

This section provides further guidance on the components that should comprise the final report of a scoping review and the information that each component should contain. It illustrates how each component of the review is to be managed in the scoping reviews analytical module of JBI's System for the Unified Management, Assessment and Review of Information (SUMARI) software. This section also provides a brief outline of how the scoping review should be formatted and the stylistic conventions that should be followed to ensure the review meets the criteria for publication in the JBI Database of Systematic Reviews and Implementation Reports. For further information please refer to the Author Guidelines of the journal:

http://journals.lww.com/jbisrir/Pages/informationforauthors.aspx Specifically, guidance is provided on the following components: outline of the report, inclusion criteria (i.e. PCC), search strategy, extraction, presenting and summarizing the results, and any potential implications for research and practice. All scoping reviews published in the JBI Database of Systematic Reviews and Implementation Reports must be based on a peer reviewed, scoping review protocol that has been accepted for publication in the same journal. For a traditional systematic review, while deviations from a published review protocol are rare, due to the more iterative nature of a scoping review, some changes may be necessary. These must still be clearly detailed and justified in the methods section of the scoping review report if and when they occur.

3.1 Title of the scoping review

The title should be clear, explicit and reflect the core elements of the review. Titles should not be phrased as questions or conclusions and there should be congruence between the title, review objective/question/s, and inclusion criteria. The title should include the phrase: "….: a scoping review". The title should not be more than 12-14 words for ease of understanding (see example above in Section 2).

3.2 Review authors

Affiliations for each author need to be stated, including the JBI affiliation of each reviewer (if relevant). A valid email address must be provided as contact details for the corresponding author.

3.3 Executive summary

This section is a structured abstract of the main features of the scoping review. It should be no longer than 500 words and should contain no abbreviations or references. The executive summary must accurately reflect and summarize the review for the reader, in particular the results of the review. The executive summary includes the following required headings:

3.3.1 Background

This section briefly describes the issue under review. While avoiding self-plagiarism, much of the detail in the background section of the scoping review report may be adapted from the background of the protocol.

For peer review only - http://bmjopen.bmj.com/site/about/guidelines.xhtml

3.3.2 Objective/s and Question/s

The review objective/s and question/s should be stated in full, as described in the protocol section.

3.3.3 Inclusion criteria

Types of participants

Important characteristics of participants should be detailed, including age and other qualifying criteria that make them appropriate for the objectives of the scoping review and match the review question.

Concept

The core concept examined by the scoping review should be clearly articulated to guide the scope and breadth of the inquiry should be explained.

Context

The context should be clearly defined and explained.

Types of sources

The source of information can include any existing literature e.g. primary research studies, systematic reviews, meta-analyses, letters, guidelines, etc. should be explained.

3.3.4 Search Strategy

Details of the approach to searching as well as the sources searched should be detailed. Any limits on the search such as dates or languages should also be included.

3.3.5 Extraction of results

The methods/tools used to extract results from the included sources should be described in brief (see Appendix 11.1 for an example).

3.3.6 Presentation of results

Details of results should be described in brief as well as how they have been organized in relation to the objective and question/s of the scoping review. This should be the principle focus of the Executive Summary. Important details of the results, including the number of studies located and included. The results extracted from the literature should be clearly detailed as well as an explanation of how the data has been charted.

3.3.7 Conclusions

Brief overall conclusions based on the scoping review results should be articulated, including a clear answer to the question/s of the scoping review and how the objective was/was not met.

Implications for research

Succinctly detail the key implications for research and further need for primary research and or systematic reviews in the field.

Implications for practice

Succinctly detail the key results that can be used to inform practice. There may be significant limitations on the kinds of implications for practice able to be developed from the results of a

For peer review only - http://bmjopen.bmj.com/site/about/guidelines.xhtml

scoping review as no methodological appraisal of the quality of included studies takes place. This section may be left out if no implications for practice are made.

For peer review only

For peer review only - http://bmjopen.bmj.com/site/about/guidelines.xhtml

3.4 Main body of the scoping review

The main body of the scoping review report follows much the same structure as the protocol; there is a section for the background, objective/s and question/s, the inclusion criteria, methods, and then the presentation of the results of the scoping review, discussion of the results and conclusions. The following sections deal with each in detail.

3.4.1 Background

The background section should be comprehensive and cover all of the main elements of the topic under review, as well as appropriate information important to the review and why the topic or question of interest lends itself to a scoping review. The primary objective of the scoping review should be evident in the background as the background situates the justification and importance of the question/s posed. While many of these details will already have been addressed in the "Background" section of the protocol, reviewers may often find that the background provided with the protocol needs modification or extension following the conduct of the scoping review itself. The background section should conclude with a statement that a preliminary search for previous scoping reviews (and ideally, systematic reviews) on the topic aligning to the same concept was conducted (state the sources searched e.g. JBI Database of Systematic Reviews and Implementation Reports, The Cochrane Database of Systematic Reviews, Campbell Collection, etc.). The background section must include a citation of the original protocol and the following sentence: "The objectives, inclusion criteria and methods of analysis for this review were specified in advance and documented in a protocol." (citation)

For publication in the JBI Database of Systematic Reviews and Implementation Reports , Vancouver style referencing must be used throughout the review with superscript numbers without brackets used for in-text citations.

3.4.2 Objective/s and Question/s

The primary objective/s of the scoping review should be stated. It can be followed by specific objectives that relate to differing conceptual foci contained in the scoping review, such as, participant groups, interventions or outcome measures or a more in depth understanding of a particular phenomenon of interest or concept. (See example above in Section 2.)

The question/s posed by the scoping review should also be included in this section.

3.4.3 Inclusion criteria

This section of the scoping review specifies the basis upon which sources were considered for inclusion in the scoping review. This section should necessarily be as transparent and unambiguous as possible. The inclusion criteria for a scoping review will be contingent on the question/s asked. The PCC should be stipulated (Population, Concept, and Context).

Types of participants

The types of participants in the papers specified sought for inclusion should be related to the objectives of the scoping review. The reasons for the inclusion or exclusion of particular participants detailed in this section should be explained clearly in the background section of the scoping review report.

Concept

For peer review only - http://bmjopen.bmj.com/site/about/guidelines.xhtml

The core concept examined by the scoping review should be clearly articulated to guide the scope and breadth of the inquiry. This may include details that pertain to the “interventions” and/or “phenomena of interest” that would be explained in greater detail in a systematic review.

Outcomes may also be a component of a scoping review’s “Concept”. If outcomes of interest are to be explained, they should be linked closely to the objective and the purpose for undertaking the scoping review.

For peer review only

For peer review only - http://bmjopen.bmj.com/site/about/guidelines.xhtml

Context

Context will vary depending on the objective/s and question/s of the review. The context should be clearly defined and may include, but is not limited to, consideration of cultural factors, such as geographic location and/or specific racial or gender-based interests. In some cases, context may also encompass details about the specific setting (such as acute care, primary health care or the community).

Types of sources

The sources of information for the scoping review should be explained. Sources can include any existing literature, e.g. primary research studies, systematic reviews, meta-analyses, letters, guidelines, etc. The source of information may be left "open" to allow for the inclusion of any, and all sources and rationale for this should be provided. Otherwise, any limits imposed on the types of sources should be detailed and explained. For example, some sources such as text and opinion papers and letters would not be particularly appropriate or useful in order to meet the objectives of particular scoping reviews.

3.4.4 Search strategy

This section documents how the reviewers search for relevant sources of information for inclusion in the scoping review. The search strategy must be comprehensively reported and the detailed search strategy for a minimum of three major bibliographic citation databases that have been searched should be appended to the review. Ideally the individual search strategies for every database searched should be presented in sequence and in a consistent format in an appendix. Clear documentation of the search strategy is a vital component of the scientific validity of any scoping review. A scoping review should ideally consider papers (primary studies, textual papers and reviews) both published and unpublished (grey literature). The timeframe (start and end dates) chosen for the search should be clearly justified and any language restrictions specified (e.g. "only studies published in English were considered for inclusion"). Any hand searching of particular relevant journals should be detailed with the journal names and years examined. Author contact, for example, to request access to known but unavailable articles should also be included along with the outcomes of that contact.

3.4.5 Extraction of results

Extraction of results for a scoping review should include extraction of all data relevant to inform the scoping review objective/s and question/s. Charting table or forms may be used (see Appendix 11.1 for a template tool). A descriptive summary of the main results organized based on the theoretical concept underpinning the review must be included. Examples of extraction fields are identified below.

Author/year

Citation details should be consistent throughout the document. The citation details include the name of the first author (Vancouver referencing style) and year of publication.

Objective/s

A clear description of the objective of the paper should be stated.

For peer review only - http://bmjopen.bmj.com/site/about/guidelines.xhtml

For peer review only

Participants (characteristics/total number)

The defining characteristics of the participants in included sources should be provided. This includes demographic details and total numbers.

Concept

Data from included studies in relation to the concept should be extracted and mapped. The concept examined by the scoping review will vary depending on the review, and should be clearly articulated to guide the scope and breadth of the inquiry. This may include details that pertain to the "interventions" and/or "phenomena of interest" that would be explained in greater detail in a systematic review. Outcomes may also be a component of a scoping review's "Concept". If outcomes of interest are to be explained, they should be linked closely to the objective and the purpose for undertaking the scoping review.

Context

Details of the context, such as location of care (acute, primary health care, community, long term care, etc.) or a particular geographical location, should be described. Cultural, racial or gender factors may be relevant.

3.6 Presenting the results

Results

The presentation of results section should identify how many studies were identified and selected. There should be a narrative description of the search decision process accompanied by the search decision flowchart (see Figure 11.1). This flowchart has been adapted from the PRISMA flowchart developed by Moher et al. (2009), but will be likely to be replaced with a purpose designed flowchart when the PRISMA-ScR is released. The flow chart should clearly detail the review decision process, indicating the results from the search, removal of duplicate citations, study selection, full retrieval and additions from a third search, and final summary presentation.

The narrative summary should logically describe the aims or purposes of the reviewed sources, concepts adopted and results that relate to the review question/s.

The results may be classified under main conceptual categories such as: "intervention type", "study population" (and sample size, if it is the case), "duration of intervention", "aims", "methodology adopted", "key findings" (evidence established) and "gaps in the research". For each category, a clear explanation should be provided.

This section should include an overall description of the included sources with reference to the detailed Table of Included Study Characteristics in the appendices (the template data extraction tool in Appendix 1 can be readily modified by reviewers to suit this purpose) . The aim of this section is to provide detail to support the inclusion of each source (paper, study, report, etc.) in the scoping review. For each source, identify the relevance to the scoping review objective and evidence for the review question. Specific results from sources may be highlighted. A summary table of included studies should be provided in the appendices of the scoping review.

Presentation of the results may map out the reviewed material in logical, diagrammatic or tabular form, and/or in a descriptive format that aligns with the objective and scope of the review. The tables and charts may show results as: distribution of studies by year or period of publication (depends on each case), countries of origin, area of intervention (clinical, policy,

For peer review only - http://bmjopen.bmj.com/site/about/guidelines.xhtml

educational, etc.), and research methods.

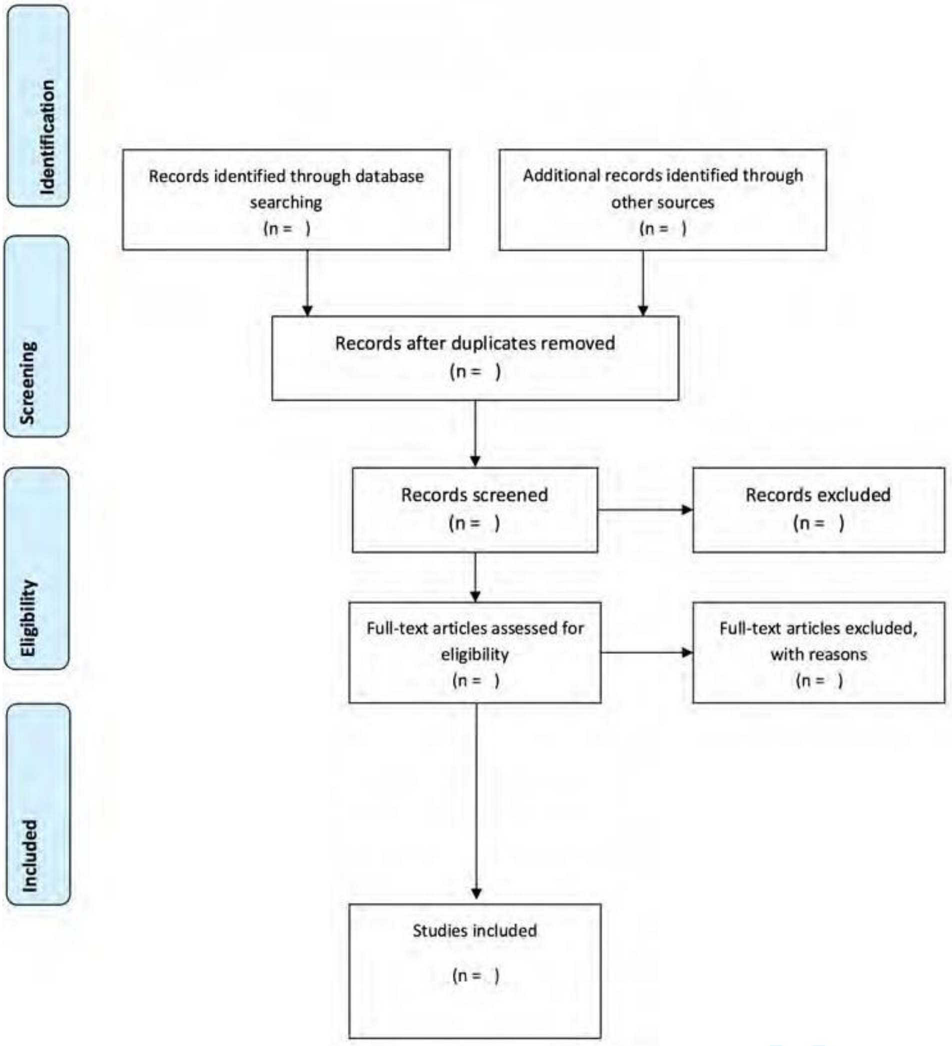

Figure 11.1: Flow Diagram for the scoping review process adapted from the PRISMA statement by Moher and colleagues (2009).

For peer review only - http://bmjopen.bmj.com/site/about/guidelines.xhtml

3.7 Discussion, Conclusion, and Implications for research and practice

3.7.1 Discussion

This section should discuss the results of the review as well as any limitations of the sources included in the scoping review. Results should be discussed in the context of current literature, practice and policy. Scoping reviews are subject to the limitations of any review, relevant sources of information may be omitted and the review is dependent on information on the review question being available. In a scoping review no rating of the quality of evidence is provided, therefore recommendations for practice cannot be graded.

3.7.2 Conclusions

This section should begin with an overall conclusion based on the results. The conclusions drawn should match the review objective/s and question/s.

3.7.3 Implications for research

This section should include clear, specific recommendations for future research based on gaps in knowledge identified from the results of the review. Authors may be able to make comments about the future conduct of systematic reviews that may be appropriate, or primary research in the area of interest.

3.7.4 Implications for practice

This section should include clear results from the scoping review that can be used to inform practice. It may not be possible to develop recommendations for practice from the results of a scoping review as no assessment of methodological quality takes place as part of a scoping review. As such this section may be left out. If implications for practice are included, the JBI Grades of Recommendation must be used (The Joanna Briggs Institute, 2014).

3.5 References

For publication in the JBI Database of Systematic Reviews and Implementation Reports, all references should be listed in full using the Vancouver referencing style, in the order in which they appear in the review. Abbreviated journal titles must be used in accordance with the United States National Library of Medicine (2016).

For peer review only

For peer review only - http://bmjopen.bmj.com/site/about/guidelines.xhtml

For peer review only

4 Review Appendices

Appendices should be numbered using Roman numerals in the order in which they have been referred to in the body of the text. While reviewers may choose to develop additional appendices for details that are unfeasible to present in the main body of the report, there are two required appendices for a JBI scoping review:

Appendix I: Search strategy

A detailed search strategy for at least one of the major databases searched must be appended.

Appendix II: Data extraction instrument

The data extraction instrument used must be appended (see the template in Appendix 11.1)

For peer review only

For peer review only - http://bmjopen.bmj.com/site/about/guidelines.xhtml

5. Chapter References

Anderson, S, Allen, P, Peckham, S & Goodwin, N 2008, 'Asking the right questions: scoping studies in the commissioning of research on the organisation and delivery of health services', *Health Res Policy Syst*, vol. 6, no. 7, viewed 20 March 2017, (online PubMed Central/US National Library of Medicine, National Institutes of Health).

Arksey, H & O'Malley, L 2005, 'Scoping studies: towards a methodological framework', *Int J Soc Res Methodol*, vol. 8, no. 1, pp.19-32.

Armstrong, R, Hall, BJ, Doyle, J, & Waters, E 2011, 'Scoping the scope of a cochrane review', *J Public* Health vol. 33, no. 1, pp. 147-50.

Centre for Reviews and Dissemination n.d., '*PROSPERO International prospective register of systematic reviews'*, CRD The University of York, York, viewed 17 March 2017, https://www.crd.york.ac.uk/PROSPERO/about.php?about=inclusioncriteria

Crilly, T, Jashapara, A & Ferlie, E 2009, *'Research utilisation and knowledge mobilisation: a scoping review of the literature*'. London: Department of Management, King's College London.

Davis, K, Drey, N & Gould, D 2009, 'What are scoping studies? A review of the nursing literature', *Int J Nurs Stud*, vol. 46, no. 10, pp.1386-400.

Decaria, J, Sharp, C & Petrella, R 2012, 'Scoping review report: obesity in older adults', *Int J Obesity*, vol. 36, no. 9, pp. 1141-50.

de Chavez, AC, Backett-Milburn, K, Parry, O & Platt, S 2005, ' *Understanding and researching wellbeing: Its usage in different disciplines and potential for health research and health promotion*', Health Educ J, vol. 64, no.1, pp. 70-87.

Ehrich, K, Freeman, GK, Richards, SC, Robinson, IC, & Shepperd, S 2002, '*How to do a scoping exercise: continuity of care*', Res Pol Plan, vol. 20, no. 1, pp. 25-9.

Grant, MJ & Booth, A 2009, *'A typology of reviews: an analysis of 14 review types and associated methodologies'*, Health Info Libr J, vol.26, no. 2, pp. 91-108.

The Joanna Briggs Institute Levels of Evidence and Grades of Recommendation Working Party 2014, *Supporting Document for the Joanna Briggs Institute Levels of Evidence and Grades of Recommendation*: The Joanna Briggs Institute, Adelaide, viewed 20 March 2017,

For peer review only - http://bmjopen.bmj.com/site/about/guidelines.xhtml

http://joannabriggs.org/jbi-approach.html#tabbed-nav=Levels-of-Evidence

Kao, SS, Peters, MDJ, Dharmawardana, N, Stew, B, & Ooi, EH 2017a, 'Pediatric tonsillectomy quality of life assessment instruments: a scoping review', *Laryngoscope.* 8 March, [Epub ahead of print].

Kao, SS, Peters, MDJ, & Ooi, E 2017b, 'Pediatric tonsillectomy quality of life assessment instruments: a scoping review protocol', *JBI Database System Rev Implement Rep.* [In Press].

Levac, D, Colquhoun, H & O'Brien, KK 2010, 'Scoping studies: advancing the methodology', *Implement Sci*, ed. 5, vol. 1, pp. 1-9.

Moher, D, Liberati, A, Tetzlaff, J & Altman, DG 2009, 'Preferred reporting items for systematic reviews and meta-analyses: the PRISMA statement', *Ann Intern Med*, vol. 151, ed. 4, pp. 264-9.

Mordiffi, SZ, Peters, MDJ & Ang, ENK 2016, 'Non-invasive thermometers used in healthcare facilities: A scoping review protocol'. *JBI Database System Rev Implement Rep'*, vol. 14, ed. 11, pp. 106-12.

Pearson, A, Wiechula, R, Court, A & Lockwood, C 2005, 'The JBI model of evidence–based healthcare', *Int J Evid Based Healthc*, vol. 3 ed. 8, pp. 207-15.

Pearson, A, White, H, Bath-Hextall, F, Salmond, S., Apostolo, J. and Kirkpatrick, P 2015, 'A mixed-methods approach to systematic reviews', *Int J Evid Based Healthc*, vol. 13, ed. 3, pp.121-31.
Peters, MDJ, Godfrey, C, Kahlil, H, McInerney, P, Baldini Soares, C & Parker, D 2015, 'Guidance for conducting systematic scoping reviews'. *Int J Evid Based Healthc,* vol. 13, ed. 3, pp. 141-46.

Peters, MDJ 2016, 'In no uncertain terms: the importance of a defined objective in scoping reviews', *JBI Database System Rev Implement Rep,* vol. 14, ed. 2, pp. 1-4.

Pham, MT, Rajić, A, Greig, JD, Sargeant, JM, Papadopoulos, A & McEwen, SA 2014, 'A scoping review of scoping reviews: advancing the approach and enhancing the consistency'. *Res Synth Methods,* vol. 5, ed. 4, pp. 371-85.

Reilly, R, Evans, K, Gorham, G, Peters, MDJ, Warren, S, O'Shea, R, Brown, A, Cass, A & Gomersall, J 2016, 'Effectiveness, cost effectiveness, acceptability and implementation barriers/enablers of chronic kidney disease management programs for Indigenous people in

For peer review only - http://bmjopen.bmj.com/site/about/guidelines.xhtml

Australia, New Zealand and Canada: a systematic review of mixed evidence', *BMC Health Serv Research,* vol. 16. 6 April, pp.119-33.

Tricco, AC, Ashoor, HM, Cardoso, R, MacDonald, H, Cogo, E, Kastner, M, Perrier, L, McKibbon, A, Grimshaw, JM & Straus, SE 2016a, 'Sustainability of knowledge translation interventions in healthcare decision-making: a scoping review' *Implement Sci*, ed. 11, vol. 1, p.55.

Tricco, AC, Lillie, E, Zarin, W, O'Brien, K, Colquhoun, H, Kastner, M, Levac, D, Ng, C, Pearson Sharpe, J, Wilson, K, Kenny, M, Warren, R, Wilson, C, Stelfox, HT & Straus, SE 2016b, 'A scoping review on the conduct and reporting of scoping reviews', *BMC Med Res Methodol*, vol. 16, ed. 15, viewed 20 March 2017, (online PubMed Central/US National Library of Medicine, National Institutes of Health).

Tricco, AC, Strauss, S & Moher, D 2015, *Preferred Reporting Items for Systematic Reviews and Meta-Analysis: extension for Scoping Reviews (PRISMA-ScR)*, Enhancing the QUAlity and Transparency Of health Research (EQUATOR) Network, Centre for Statistics in Medicine, NDORMS, University of Oxford, London, viewed 17 March 2017, http://www.equator-network.org/library/reporting-guidelines-under-development/#55

The United States National Library of Medicine (U.S. NLM). 2016. *FAQ: NLM® LocatorPlus® -- Database of NLM-Held Journal Titles and Abbreviations*, Rockville Pike, Bethesda, viewed 17 March 2017, https://www.nlm.nih.gov/services/lpabbrev.html

Valaitis, R, Martin-Misener, R, Wong, ST, MacDonald, M, Meagher-Stewart, D, Austin, P, Kaczorowksi, J, O-Mara, L, Savage, R, Strengthening Primary Health Care through Public Health and Primary Care Collaboration Team 2012, 'Methods, strategies and technologies used to conduct a scoping literature review of collaboration between primary care and public health', *Prim Health Care Res Dev*, vol. 13, ed. 3, pp. 219-36.

Watson, R, Parr, JR, Joyce, C, May, C & Le Couteur, AS 2011, 'Models of transitional care for young people with complex health needs: a scoping review', *Child Care Health Dev,* vol. 37, ed. 6, pp.780-91.

For peer review only - http://bmjopen.bmj.com/site/about/guidelines.xhtml

4.1:Appendix 1: JBI Template study details, characteristics and results extraction instrument

**Scoping Review Details**	
Scoping Review title:	
Review objective/s:	
Review question/s:	
**Inclusion/Exclusion Criteria**	
Population	
Concept	
Context	
Types of Study	
**Study Details and Characteristics**	
Study citation details (e.g. author/s, date, title, journal, volume, issue, pages)	
Country	
Context	
Participants (details e.g. age/sex and number)	
**Details/Results extracted from study** (in relation to the concept of the scoping review)	
E.g. Quality of Life Domains assessed	
E.g. Number of items in tool	
E.g. details of psychometric validation of tool	

For peer review only

For peer review only - http://bmjopen.bmj.com/site/about/guidelines.xhtml

For peer review only

For peer review only - http://bmjopen.bmj.com/site/about/guidelines.xhtml

View publication stats
